# Supplementary material for: Mathematical Modelling for Optimal Vaccine Dose Finding: Maximising Efficacy and Minimising Toxicity
Source: Vaccines (Basel). 2022 May 11;10(5):756. doi: 10.3390/vaccines10050756 (PMC9144167; doi:10.3390/vaccines10050756)
Supplement: Supplementary file 1 [file vaccines-10-00756-s001.zip › vaccines-1699875-supplementary.pdf]

Supplementary 1. Models

Saturating - Sigmoid

The following model is a classical dose-response model for modelling dose-efficacy in drugs.

$$Saturating(dose) = \frac{1}{1+e^{(gradient \times (midpoint - dose))}}$$

This may be recognisable as the model for logistic regression. This has an efficacy of 0 as dose tends to negative infinity, and an efficacy of 1 as dose tends to positive infinity. In this work we made the adjustment of multiplying this by a parameter 'maximum' that is between 0 and 1. This was because for a given vaccine candidate assuming that efficacy can become 100% for sufficiently large doses seemed unreasonable. It seemed possible that an otherwise immunogenic vaccine may not be immunogenic in some individuals regardless of dose, for example due to variations in major histocompatibility complexes [1].

Peaking - Latent Quadratic

The latent quadratic model has been commonly used for the purposes of modelling non-monotonically increasing dose response. It is similar to the saturating model, other than it includes quadratic terms to allow qualitative dose-response to vary between small and large doses. By differentiation it can be shown that the maxima point within the parameter bounds used is at:

$$dose = -\frac{gradient1}{2 \times gradient2}$$

Weighted

Please see Supplementary weighted model averaging.

Probit

An excellent description of the probit model for ordinal toxicities can be found in [2]. In short, we consider the probability of observing toxicity less than grade j given dose $P(Y = j|dose)$ by:

$$P(Y = 0|dose) = P(Y < 1|dose) - P(Y < 0|dose) = P(Y < 1|dose) - 0$$
$$P(Y = 1|dose) = P(Y < 2|dose) - P(Y < 1|dose)$$
$$P(Y = 2|dose) = P(Y < 3|dose) - P(Y < 2|dose)$$
$$P(Y = 3|dose) = P(Y < 4|dose) - P(Y < 3|dose) = 1 - P(Y < 3|dose)$$

$P(Y < 0|dose)$ and $P(Y < 4|dose)$ are respectively 0 and one (0 and 3 are assumed to be the lowest and highest adverse event gradings possible). Otherwise $P(Y < j|dose)$ is given by $\phi\left(threshold_j - gradient \times dose\right)$, where ϕ() is the cumulative density function with $\sigma = 1$. Hence at dose = $threshold_j$/gradient, $P(Y < j|dose)$ = 0.5.

Parameters and bounding

Within the calibration of these models, certain bounds were used to aim the calibration and for biological plausibility.

Parameter	Bounds	Notes
	Saturating - Sigmoid	
gradient	0 to 6	It seems biologically unlikely that a vaccine dose-efficacy curve is discontinuous or that a relatively small change in dose could be responsible for a massive change in vaccine efficacy. Therefore the gradient was bounded to prevent an overly steep curve. The maximum gradient of 6 would mean that a single log increase in dose (only 1/10th of the dosing space) could be responsible for 90% of the change in vaccine efficacy. None of the scenarios had a gradient steeper than this, so if it is believed that 1/10th of

		the dosing space could be responsible for >90% of the change in vaccine efficacy a higher value should be used.<br><br>A lower bound of 0 prevents efficacy from being decreasing w.r.t. increasing dose, which is one of the assumptions of saturating dose-response.
midpoint	0 to infinity	This ensures that the model cannot predict that efficacy is saturating below the lowest dose in the dosing space. This assumption was true for all scenarios, but could be relaxed if it is believed that efficacy may have already begun saturating at the lowest dose in the dosing space.
maximum	0 to 1	Having a greater than 1 or less than 0 probability of efficacy is biologically and probabilistically impossible.
Peaking - Latent Quadratic		
base	-infinity to infinity	Unbounded to allow other parameters to well define the model.
gradient1	0 to 6	See notes for the gradient parameter of the saturation sigmoid model. Both used the same bounds to ensure fairness in comparison.
gradient2	-infinity to 0	Bounding to be less than zero is to ensure that an increasing dose will eventually lead to a

		decreasing efficacy, an assumption of the peaking model.
Toxicity Probit		
gradient	0 to 6	See notes for the gradient parameter of the saturation sigmoid model.
threshold1	-infinity to infinity	Unbounded to allow high or low levels of this toxicity grade at either the lowest or highest doses in the dosing space.
threshold2	-infinity to infinity	As above.
threshold3	-infinity to infinity	As above.

Supplementary 2. Weighted model averaging

Model averaging can be a useful method for inference when the current model form is uncertain. We use the method of model weighting via the Akaike Information Criterion (AIC) model averaging [3]. We outline this here.

Suppose that we have two competing models M_1 and M_2. After gathering data and calibrating models we have respective likelihoods L_1 and L_2 for the two models. Then AIC_1 and AIC_2 can be calculated as

$$AIC_i = -2ln(L_i) + 2k_i$$

Where k_i is the number of parameters for model i. Let AIC_{best} be the minimum of AIC_1 and AIC_2. Then $\nabla_i = AIC_i - AIC_{best}$ for each model.

The Akaike weights w_1 and w_2 can be calculated as

$$w_i = \frac{exp(-\nabla_i)}{exp(-\nabla_1) + exp(-\nabla_2)}$$

Finally the predictions of the weighted model at some input value x are hence given by

$$M_w(x) = w_1 M_1(x) + w_2 M_2(x)$$

Where $M_1(x)$ and $M_2(x)$ are respective predictions for M_1 and M_2 at input value x.

Supplementary 3. Pareto Optimality

In multi-objective optimisation problems, multiple objectives are required to be maximised, minimised, or kept within some thresholds. In our work, this is shown by the requirements of maximising efficacy and minimising some level of toxicity (in this section we consider toxicity as binary rather than the ordinal perspective of the main work, this is for simplicity in explanation).

Consider table S.Pareto.1. Here two vaccines have been trialed and found to have respective efficacy and toxicity percentages. Clearly vaccine B is optimal compared to vaccine A, as both efficacy is higher and toxicity is lower.

Vaccine	Efficacy (%)	Toxicity (%)
A	50	40
B	70	30

Table S.Pareto.1.

Vaccine	Efficacy (%)	Toxicity (%)
A	60	20
B	80	50

Table S.Pareto.2.

Now instead consider tables S.Pareto.2. Here neither vaccine can be considered truly 'optimal' relative to the other, as A better minimises toxicity and B better maximises toxicity. Hence the concept of 'optimal' is poorly defined in this case, and we can only consider 'pareto optimality' and 'domination'.

In the first of these examples we say that 'B dominates A'. This means that, for all objectives, B is at least as good as A, and that there is at least one objective for which B is preferable to A. An option (be that a vaccine in this case or a dose in the main body of work) is 'pareto optimal' if it is not dominated by any other option. So in table S.Pareto.1, only vaccine B is pareto optimal. In

table S.Pareto.2, both vaccine A and vaccine B are pareto optimal. In table S.Pareto.3, vaccine A is dominated by vaccine B and vaccine C, vaccine B is not dominated by any other vaccine, vaccineC is not dominated by any other vaccine, and vaccine D is dominated by all other options. Hence here only vaccine B and vaccine C are pareto optimal.

Vaccine	Efficacy (%)	Toxicity (%)
A	50	40
B	60	40
C	70	50
D	20	90

Table S.Pareto.1.

This is important when considering dose optimisation, and informs why a utility function is needed. Considering figure S.Pareto.1, we see that when dose-efficacy and dose-toxicity are saturating, all doses are pareto optimal, and so defining optimal dose as the dose which maximises efficacy and minimises toxicity is effectively meaningless in this case. When does-efficacy is peaking, all doses greater than that which maximises efficacy are dominated by the dose that maximises efficacy, and hence all doses less than this dose are pareto optimal [figure S.Pareto.2].

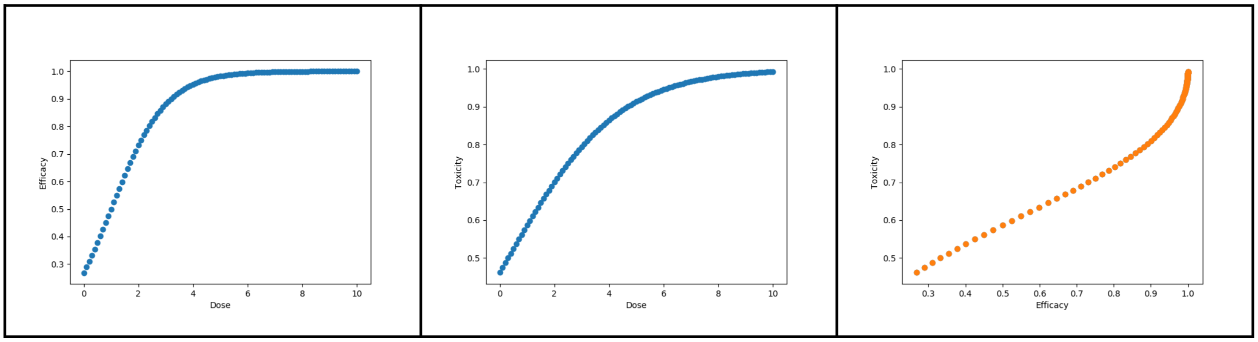

Figure S.Pareto.1. Example saturating dose-efficacy (left), dose-toxicity(middle), and resultant efficacy-toxicity (right) plots. For the efficacy-toxicity plot, pareto optimal doses are in orange (here all doses are pareto optimal).

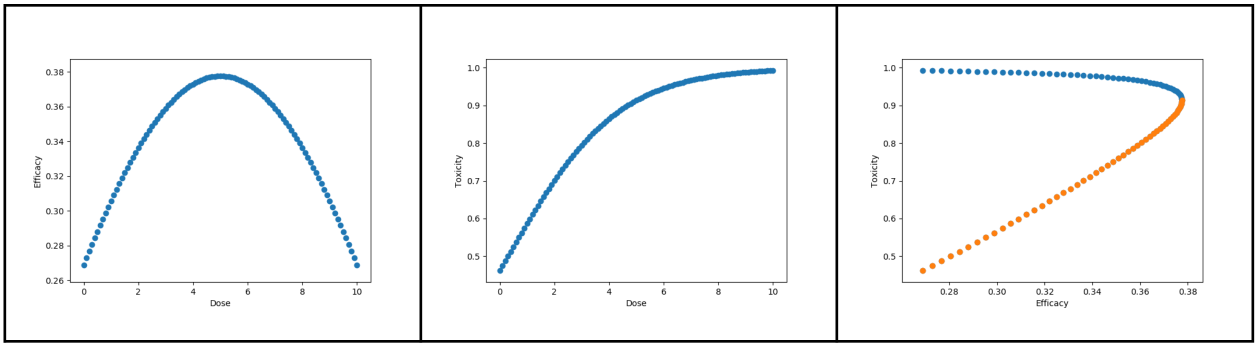

Figure S.Pareto.2. Example peaking dose-efficacy (left), dose-toxicity(middle), and resultant efficacy-toxicity (right) plots. For the efficacy-toxicity plot, pareto optimal doses are in orange and non-pareto optimal doses are in blue (here all doses less than that which maximises efficacy are pareto optimal).

This should highlight why explicitly defining a utility function is key to meaningfully determining 'optimal' dose.

Supplementary 4. Scenarios

We aimed for the scenarios to be qualitatively different in both shape and optimal dose.

Scenario Saturating 1

Qualitatively this scenario had a saturating efficacy curve and a high optimal dose, with a relatively steep utility curve.

	Parameter	Value
Efficacy	gradient	1.000
	midpoint	6.000
	maximum	0.900
Toxicity	gradient	1.000
	threshold1	3.000
	threshold2	9.000
	threshold3	10.500
Utility Weights	$\text{Weight}_{\text{Efficacy}}$	0.133

Table.S.Scenarios.Saturating 1. Parameters for the scenario Saturating 1

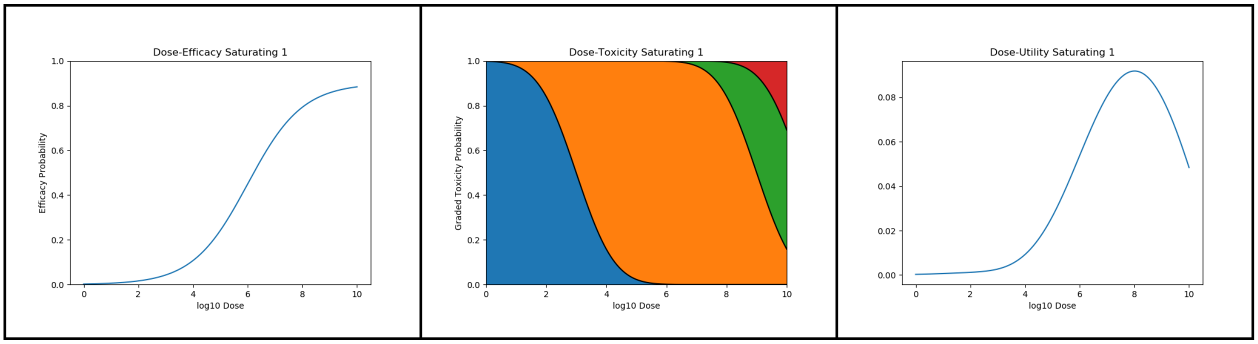

Figure.S.Scenarios.Saturating 1. Dose-efficacy, dose-toxicity, and dose-utility plots for the scenario Saturating 1

Scenario Saturating 2

Qualitatively this scenario had a saturating efficacy curve and a middling optimal dose, with a relatively broad utility curve.

	Parameter	Value
Efficacy	gradient	1.800
	midpoint	2.500
	maximum	0.900
Toxicity	gradient	0.500
	threshold1	1.000
	threshold2	4.000
	threshold3	5.000
Utility Weights	$\text{Weight}_{\text{Efficacy}}$	0.133

Table.S.Scenarios.Saturating 2. Parameters for the scenario Saturating 2

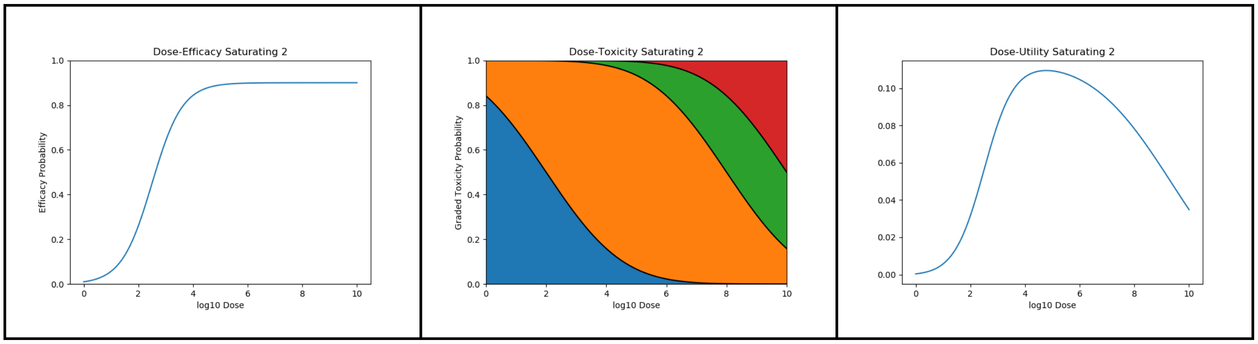

Figure.S.Scenarios.Saturating 2. Dose-efficacy, dose-toxicity, and dose-utility plots for the scenario Saturating 2

Scenario Saturating 3

Qualitatively this scenario had a saturating efficacy curve and a low optimal dose, with a relatively steep utility curve.

	Parameter	Value

Efficacy	gradient	2.500
	midpoint	2.000
	maximum	0.900
Toxicity	gradient	0.500
	threshold1	0.100
	threshold2	2.500
	threshold3	3.000
Utility Weights	$\text{Weight}_{\text{Efficacy}}$	0.133

Table.S.Scenarios.Saturating 3. Parameters for the scenario Saturating 3

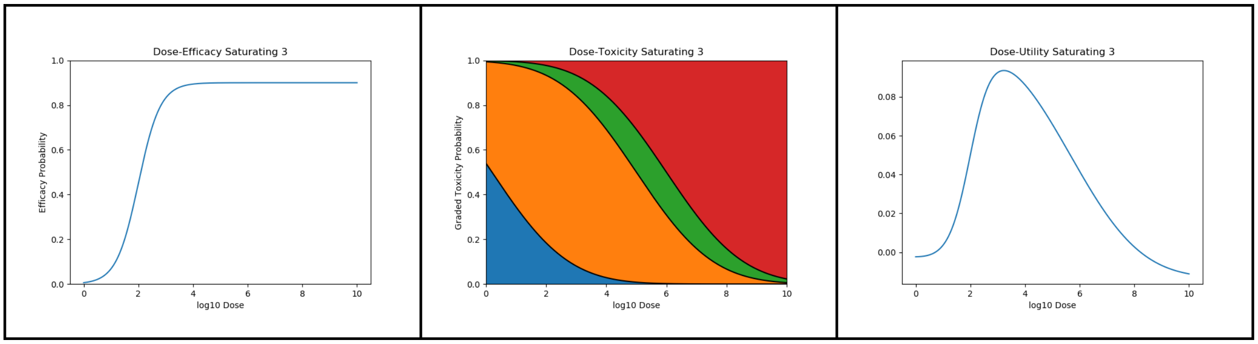

Figure.S.Scenarios.Saturating 3. Dose-efficacy, dose-toxicity, and dose-utility plots for the scenario Saturating 3

Scenario Saturating 4

Qualitatively this scenario had a saturating efficacy curve and a high optimal dose at 10, representing the case where the 'true optimal' is not within the dosing space.

	Parameter	Value
Efficacy	gradient	0.700
	midpoint	7.500
	maximum	0.900
Toxicity	gradient	0.500

	threshold1	1.000
	threshold2	2.000
	threshold3	5.000
Utility Weights	$Weight_{Efficacy}$	.266

Table.S.Scenarios.Saturating 4. Parameters for the scenario Saturating 4

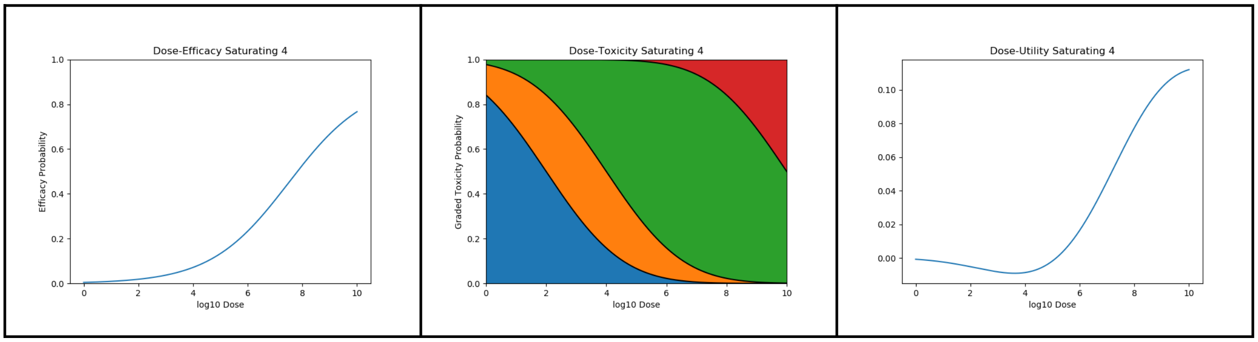

Figure.S.Scenarios.Saturating 4. Dose-efficacy, dose-toxicity, and dose-utility plots for the scenario Saturating 4

Scenario Saturating 5

Qualitatively this scenario had a saturating efficacy curve, which changed only gradually over the dosing space.

	Parameter	Value
Efficacy	gradient	0.100
	midpoint	8.000
	maximum	1.000
Toxicity	gradient	0.500
	threshold1	1.000
	threshold2	4.000
	threshold3	5.000
Utility Weights	$Weight_{Efficacy}$	.266

Table.S.Scenarios.Saturating 5. Parameters for the scenario Saturating 5

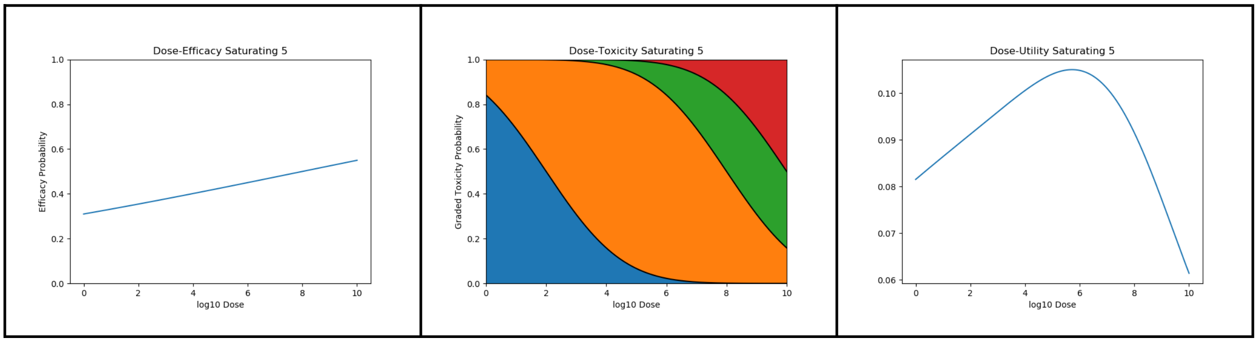

Figure.S.Scenarios.Saturating 5. Dose-efficacy, dose-toxicity, and dose-utility plots for the scenario Saturating 5

Scenario Peaking 1

Qualitatively this scenario had a peaking efficacy curve and a high optimal dose, with a relatively steep utility curve.

	Parameter	Value
Efficacy	base	-9.000
	gradient1	3.000
	gradient2	-0.214
Toxicity	gradient	1.000
	threshold1	3.000
	threshold2	9.000
	threshold3	10.500
Utility Weights	$Weight_{Efficacy}$	0.133

Table.S.Scenarios.Peaking 1. Parameters for the scenario Peaking 1

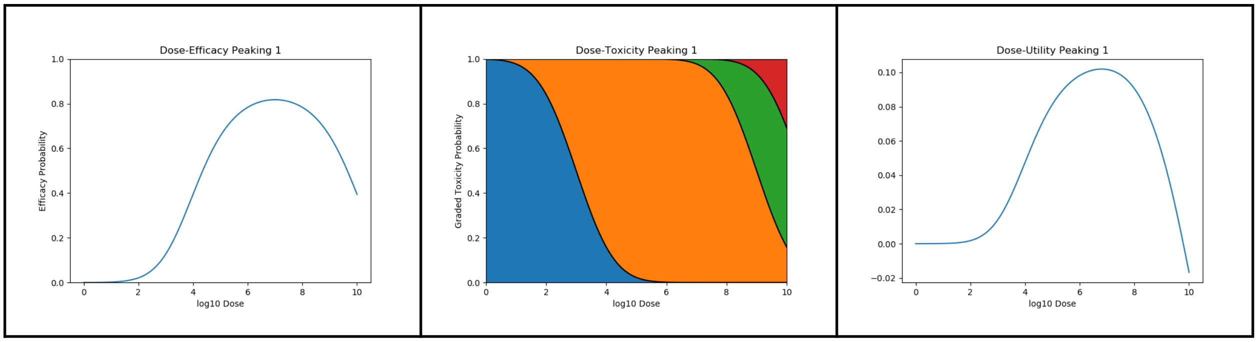

Figure.S.Scenarios.Peaking 1. Dose-efficacy, dose-toxicity, and dose-utility plots for the scenario Peaking 1

Scenario Peaking 2

Qualitatively this scenario had a peaking efficacy curve and a middling optimal dose, with a relatively broad utility curve.

	Parameter	Value
Efficacy	base	-4.000
	gradient1	2.000
	gradient2	-0.166
Toxicity	gradient	0.100
	threshold1	0.100
	threshold2	0.400
	threshold3	1.500
Utility Weights	$\text{Weight}_{\text{Efficacy}}$	0.133

Table.S.Scenarios.Peaking 2. Parameters for the scenario Peaking 2

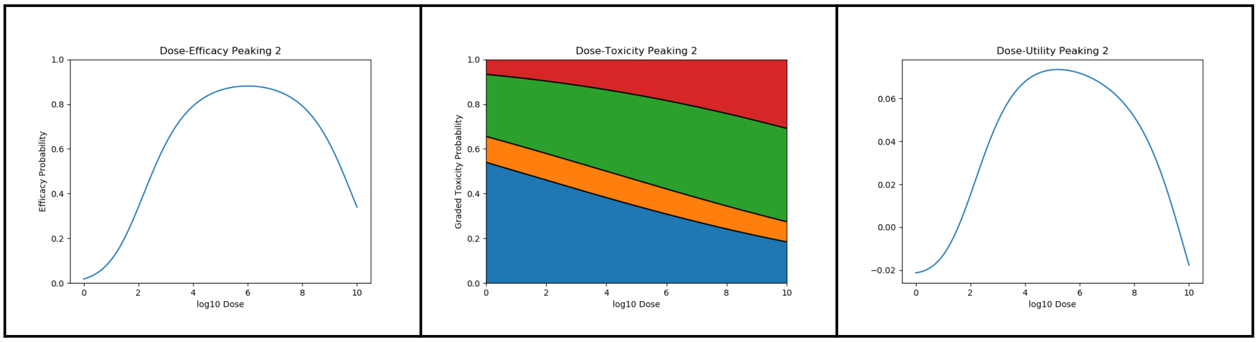

Figure.S.Scenarios.Peaking 2. Dose-efficacy, dose-toxicity, and dose-utility plots for the scenario Peaking 2

Scenario Peaking 3

Qualitatively this scenario had a peaking efficacy curve and a low optimal dose, with a relatively steep utility curve.

	Parameter	Value
Efficacy	base	-6.000
	gradient1	5.000
	gradient2	-0.750
Toxicity	gradient	0.500
	threshold1	1.000
	threshold2	3.000
	threshold3	5.000
Utility Weights	$Weight_{Efficacy}$	0.133

Table.S.Scenarios.Peaking 3. Parameters for the scenario Peaking 3

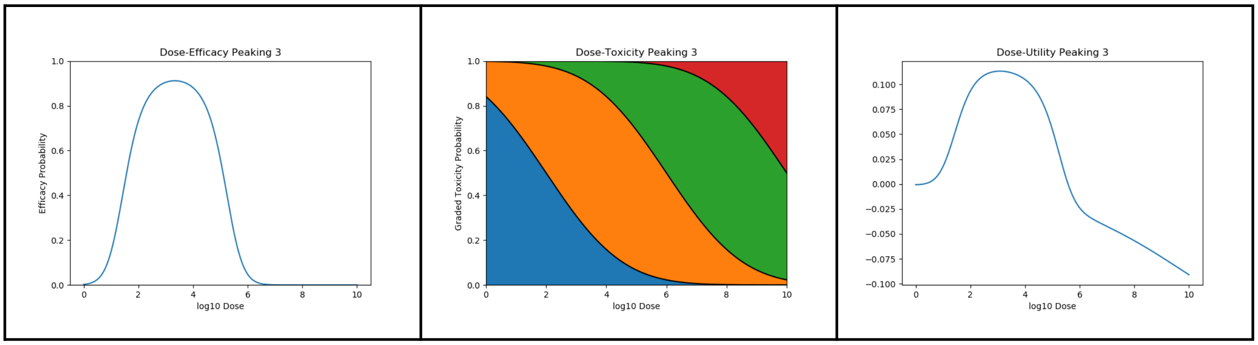

Figure.S.Scenarios.Peaking 3. Dose-efficacy, dose-toxicity, and dose-utility plots for the scenario Peaking 3

Scenario Peaking 4

Qualitatively this scenario had a peaking efficacy curve and a high optimal dose at 10, representing the case where the 'true optimal' is not within the dosing space.

Peaking 4 uses a peaking model, but efficacy is still increasing at the maximum dose. Thus this is effectively a saturating scenario, but represents the potential case where dose-efficacy is peaking but this is unimportant within the feasible dosing space.

	Parameter	Value
Efficacy	base	-12.000
	gradient1	2.500
	gradient2	-0.114
Toxicity	gradient	.300
	threshold1	1.000
	threshold2	1.500
	threshold3	2.000
Utility Weights	$Weight_{Efficacy}$	0.266

Table.S.Scenarios.Peaking 4. Parameters for the scenario Peaking 4

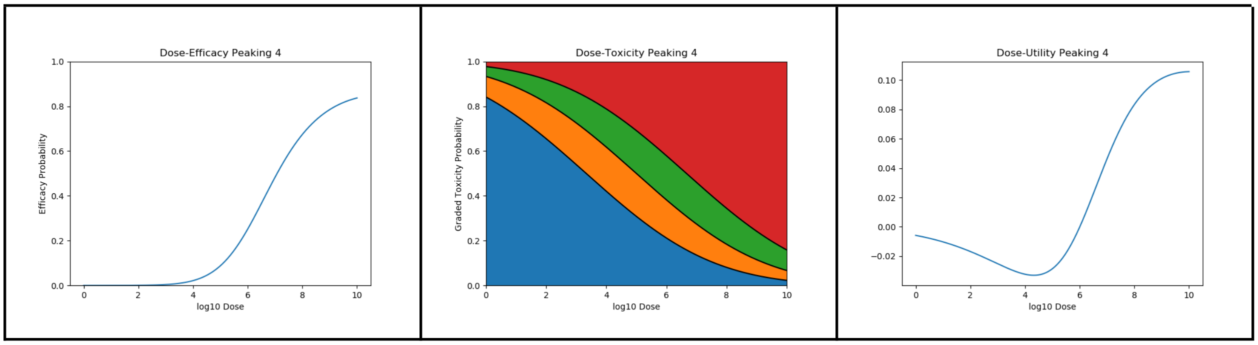

Figure.S.Scenarios.Peaking 4. Dose-efficacy, dose-toxicity, and dose-utility plots for the scenario Peaking 4

Scenario Peaking 5

Qualitatively this scenario had a peaking efficacy curve, which changed only gradually over the dosing space.

	Parameter	Value

Efficacy	base	0.000
	gradient1	0.800
	gradient2	0.067
Toxicity	gradient	0.100
	threshold1	0.100
	threshold2	0.400
	threshold3	1.500
Utility Weights	$Weight_{Efficacy}$	0.133

Table.S.Scenarios.Peaking 5. Parameters for the scenario Peaking 5

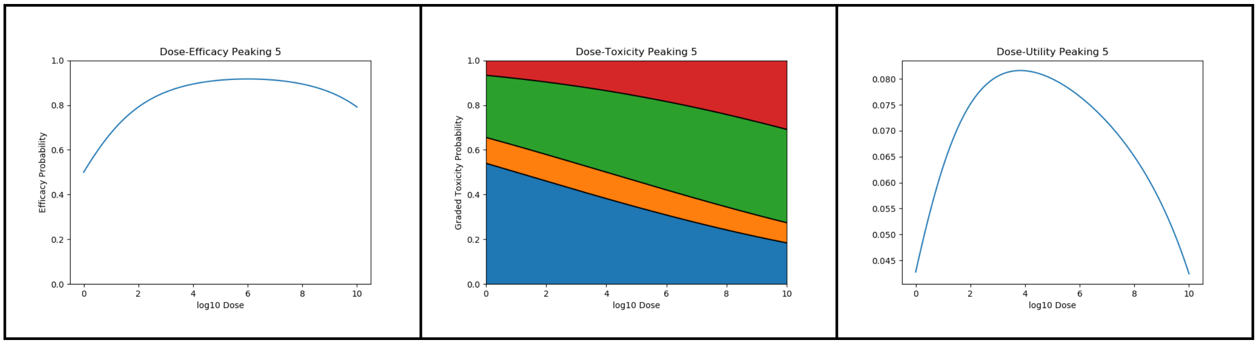

Figure.S.Scenarios.Peaking 5. Dose-efficacy, dose-toxicity, and dose-utility plots for the scenario Peaking 5

Scenario Other 1

Other 1 represents a vaccine for which nearly zero efficacy is observed for all doses. The efficacy model is given as

$$Flat(dose) \ = \ base$$

	Parameter	Value
Efficacy	base	0.020
Toxicity	gradient	0.500
	threshold1	0.000

	threshold2	3.000
	threshold3	5.000
Utility Weights	$\text{Weight}_{\text{Efficacy}}$	0.133

Table.S.Scenarios.Other 1. Parameters for the scenario Other 1

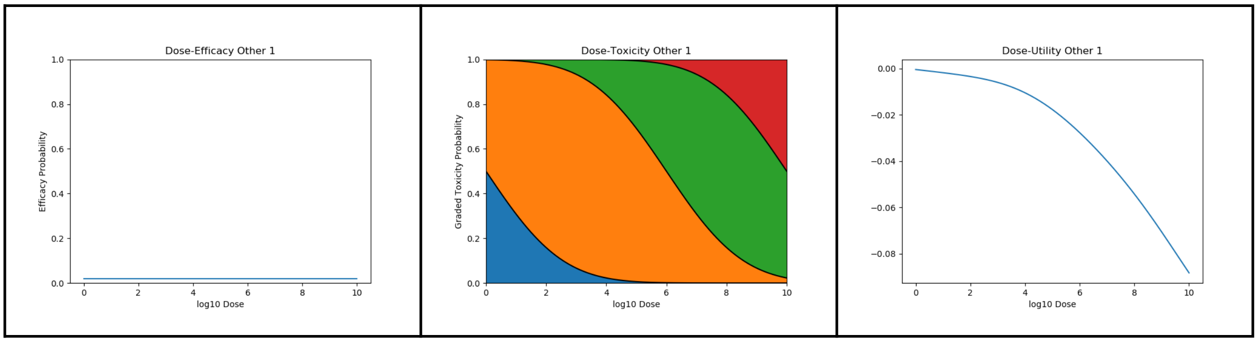

Figure.S.Scenarios.Other 1. Dose-efficacy, dose-toxicity, and dose-utility plots for the scenario Other 1

Scenario Other 2

Other 2 represents a vaccine for which dose-efficacy is fundamentally saturating, but follows a different and more complicated bishasic parametric form to the sigmoid saturating model assumed elsewhere in this paper. The efficacy model is given as

$$Biphasic(dose) \;=\; \frac{maximum \times fraction}{1+e^{(gradient1 \times (midpoint1\,-\,dose))}} \;+\; \frac{maximum \times (1 - fraction)}{1+e^{(gradient2 \times (midpoint2\,-\,dose))}}$$

	Parameter	Value
Efficacy	gradient1	0.500
	gradient2	3.000
	midpoint1	4.000
	midpoint2	6.000
	maximum	0.900
	fraction	0.500
Toxicity	gradient	0.500

	threshold1	1.000
	threshold2	3.000
	threshold3	5.000
Utility Weights	$\text{Weight}_{\text{Efficacy}}$	0.133

Table.S.Scenarios.Other 2. Parameters for the scenario Other 2

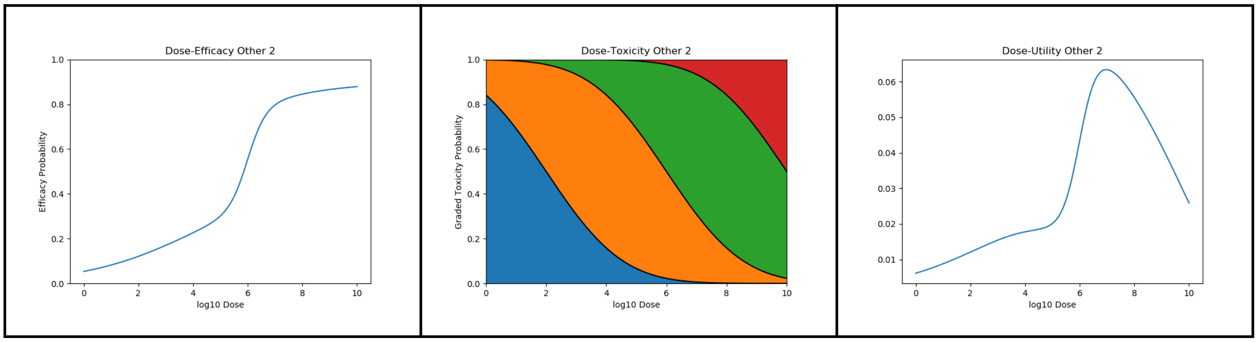

Figure.S.Scenarios.Other 2. Dose-efficacy, dose-toxicity, and dose-utility plots for the scenario Other 2

Scenario Other 3

Other 3 represents a vaccine for which dose-efficacy is fundamentally peaking, but follows a different and more complicated biphasic parametric form to the latent quadratic saturating model assumed elsewhere in this paper. The efficacy model is given as

$$Biphasic(dose) = \frac{maximum \times fraction}{1+e^{(gradient1 \times (midpoint1 - dose))}} + \frac{maximum \times (1-fraction)}{1+e^{(gradient2 \times (midpoint2 - dose))}}$$

	Parameter	Value
Efficacy	gradient1	1.000
	gradient2	2.000
	midpoint1	4.000
	midpoint2	7.000
	maximum	0.500
	fraction	2.000

Toxicity	gradient	0.500
	threshold1	1.000
	threshold2	3.000
	threshold3	5.000
Utility Weights	$\text{Weight}_{\text{Efficacy}}$	0.133

Table.S.Scenarios.Other 3. Parameters for the scenario Other 3

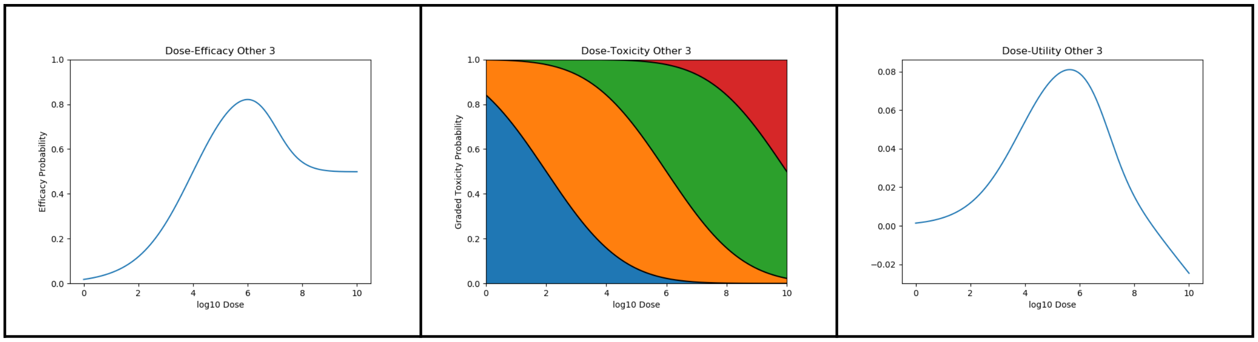

Figure.S.Scenarios.Other 3. Dose-efficacy, dose-toxicity, and dose-utility plots for the scenario Other 3

Scenario Other 4

Other 4 represents a vaccine for which dose-efficacy is fundamentally saturating, but follows yet another different parametric saturating model, the linear model. The efficacy model is given as

$$linear(dose) = \frac{maximum \times dose}{gradient + dose}$$

	Parameter	Value
Efficacy	gradient	3.000
	maximum	1.200
Toxicity	gradient	0.200
	threshold1	1.000
	threshold2	1.200
	threshold3	2.000

Utility Weights	$Weight_{Efficacy}$	0.133

Table.S.Scenarios.Other 4. Parameters for the scenario Other 4

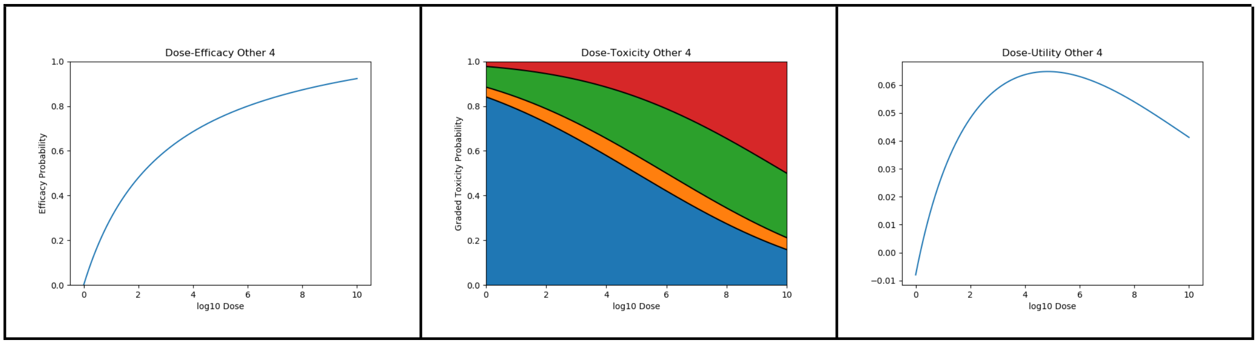

Figure.S.Scenarios.Other 4. Dose-efficacy, dose-toxicity, and dose-utility plots for the scenario Other 4.

Supplementary 5. Softmax

Method

Softmax selection is a method of action selection used commonly in both multi-armed bandit problems and reinforcement learning. We provide a description of action/dose selection under this method. Let A1, A2, …., An be the n possible actions available to be taken, each with respective predicted utility U1, U2,..., Un. Then an action Ai is selected to test (dose selected to trial) with probability

$$Probability\ of\ selecting\ action\ A_i = \frac{exp(inverse\_temperature \times U_i)}{\sum_{j=1}^{n} exp(inverse\_temperature * \times)}$$

where inverse_temperature is a hyperparameter which controls the degree of exploration. An increased inverse temperature leads to actions with lower predicted utility having lower probability of selection. For inverse_temperature = 0, which is the lowest possible inverse_temperature, all actions are selected with equal probability (1/n). A random number generator is used to select an action with these probabilities.

In the case of the 3 stage softmax, where multiple doses are selected at once, actions/doses are sampled with these probabilities independently of the other actions selected.

inverse_temperature values

For the balanced fully continuous trial dose selection method we used inverse_temperature = 69. This was chosen such that a utility difference of 0.01 would have a doubled probability of selection. This is shown

$$2 \times exp(inverse\_temperature \times U_i) = exp(inverse\_temperature \times (U_i + .01))$$
$$= exp(inverse\_temperature \times U_i) \times exp(inverse\_temperature \times 0.01)$$
$$2 = exp(inverse\_temperature \times 0.01)$$
$$ln(2) = 0.69 = inverse\_temperature \times 0.01$$
$$69 = inverse\_temperature$$

For the three stage softmax dose selection method we used inverse_temperature = (58.9,294) for selecting the (2nd, 3rd) stage of doses. This was chosen such that for utility difference of (0.05, 0.01) would lead to the dose that was predicted better being selected 95% of the time . This is shown in the 0.05 case by

$$95 \times exp(inverse\_temperature \times U_i) = 5 \times exp(inverse\_temperature \times (U_i + .05))$$
$$= 5 \times exp(inverse\_temperature \times U_i) \times exp(inverse\_temperature \times 0.05)$$
$$19 = exp(inverse\_temperature \times 0.05)$$
$$ln(19) = 2.94 = inverse\_temperature \times 0.05$$
$$58.9 = inverse\_temperature$$

And similarly for 0.01.

These may not have been optimal values, but optimal values are likely to vary depending on the scenario.

Supplementary 6. Pseudodata

Psuedodata or anchor points are used to stabilise and inform models for which little real data is available. In short, we pretend that there exist data which does not actually exist, but have these data points being less important than real data, and further downweight or ignore these data as more data is gathered. We aim to use minimally informative psuedodata. For all approaches pseudodata is fully ignored for final dose selection.

Efficacy Models

For efficacy modelling, pseudo data were of the form in table S.Pseudodata.1. Thus there were 300 pseudo individuals divided evenly over 3 doses.

For the standard and balanced fully continual approaches the weight of a pseudodata-point was 0.01 of regular datapoint. Thus the effective sample size of the pseudodata was 3 (=300x0.01), which is quickly minimal relative to the amount of real data.

For the 3 stage softmax approaches the weights of a pseudodata-point were 0.01 and 0.001 for the second and third trial dose selections respectively. Thus the effective sample size of the pseudodata were 3 and 0.3. Thus for the third dose selection the pseudodata represented only 1.47% (=0.3/ (20+0.3)) of the data.

Dose	Non-efficacy response	Efficacy Response
1	90	10
5	50	50
9	10	90

Table S.Pseudodata.1. Efficacy pseudodata

Toxicity Model

For toxicity modelling, pseudo data were of the form in table S/Pseudodata.2. Thus there were 200 pseudo individuals divided evenly over 2 doses.

Again, for the standard and balanced fully continual approaches the weight of a pseudodatapoint was 0.01 of regular datapoint. Thus the effective sample size of the pseudodata was 2, which is quickly minimal relative to the amount of real data.

For the 3 stage softmax approaches the weights of a pseudodatapoint were 0.01 and 0.001 at the second and third trial dose selections respectively. Thus the effective sample size of the pseudodata were 2 and 0.2. Thus for the third dose selection the psuedodata represented only 0.99% (=0.2/20.2) of the data.

Dose	Grade 0 Response	Grade 1 Response	Grade 2 Response	Grade 3 Response
1	45	35	10	10
9	2	3	5	90

Table S.Pseudodata.2. Toxicity pseudodata

Supplementary 7. Copeland

Copeland's method is a method of ranking that effectively asks the question "how often would we have preferred to have used this option over a different option". The process is to compare the metrics of each simulation, and see which approach did 'best'.
In a comparison between the ith simulation for approach A and and the jth simulation for approach B:

If A_i was better that B_j: A scores +1, B scores +0

If A_i was worse that B_j: A scores +0, B scores +1

If A_i and B_j were the same: Both score +0.5

In any case the count of A comparisons (n_A) and count of B comparisons (n_B) also increase by 1
Comparisons are conducted for all i and j between all approaches, then divided by the count of comparisons for that approach

It is well discussed, but here we show a concrete example for a tiny dataset. Recall that a lower PSR is preferable.

	Approach A	Approach B	Approach C
Respective PSR from the first and second simulation of each approach.	5	9	7
	6	1	5

Table S.Copeland.1. Scenario 1 Data

The first simulation of approach A got a PSR of 5.

- The first simulation of approach B got a PSR of 9. As this is greater (and therefore less preferable) than the PSR for that simulation of approach A, score A increases by 1 and score B says the same.

- The second simulation of approach B got a PSR of 1. As this is lower (and therefore preferable) than the PSR for that simulation of approach A, score B increases by 1 and score A says the same.
- The first simulation of approach C got a PSR of 7. As this is greater (and therefore less preferable) than the PSR for that simulation of approach A, score A increases by 1 and score C says the same.
- The second simulation of approach C got a PSR of 5. As this is equal to the PSR for that simulation of approach A, score A and score C increase by 0.5.

Thus after making comparisons for simulation 1 of approach A we have score A = 2.5 and n_A = 4.

The second simulation of approach A got a PSR of 6. Repeating the steps leads to score A increasing to 4.5(=2.5+2) and n_A=8(=4+4). Thus the total copeland score is 0.5625(=4.5/8).

This can be repeated for all 3 approaches, and approaches ranked by their Copeland Score to give the following table.

	Approach A	Approach B	Approach C
Copeland Score	0.5625	0.5	0.4375
Copeland Rank	1	2	3

Table S.Copeland.2. Scenario 1 Copeland Table

Suppose that there was a second scenario that these approaches were tested on, with data as below.

	Approach A	Approach B	Approach C
Respective simple regret from the first and second simulation of each approach.	8	3	6
	10	5	4

Table S.Copeland.3. Scenario 2 Data

This could give the table below, representing the Copelands scores and ranking for that scenario.

	Approach A	Approach B	Approach C
Copeland Score	0	0.875	0.625
Copeland Rank	3	1	2

Table S.Copeland.4. Scenario 2 Copeland Table

We could combine these by summing the scores and comparisons for both scenarios. So for example, in scenario 1 we had $score_A = 4.5$ and $n_A = 8$. In scenario 2 we had $score_A = 0$ and $n_A = 8$. Therefore we have a total $score_A = 4.5$ and $n_A = 16$ for a total Copeland score of 0.2815. Repeating this gives aggregate copelands metrics in the below table.

	Approach A	Approach B	Approach C
Copeland Score	0.28125	0.6875	0.53125
Copeland Rank	3	1	2

Table S.Copeland.5. Aggregate Copeland table for both scenarios.

Note that this can be read as approach B 'winning' 68.75% of comparisons across both scenarios. From this we could say that Approach B seems to be most effective for minimising PSR across both of these scenarios.

Supplementary 8. Exceptions

These exceptions were (n=60,100, scenario = Saturating 3) , and (n=60,100, scenario = Other 2) [supplementary obj 1 plots Saturating 3, Other 2]. For all other scenarios the peaking latent-quadratic performed similarly or better than the sigmoid saturating curve. We suggest that, for the Saturating 3 scenario, the peaking curve was unable to approximate well the efficacy curve saturating at a low dose and remaining high across the rest of the dosing-space. We suggest that, for the Other 2 scenario, the steady increase in efficacy with a large jump in efficacy near the middle in Other 2 could have been the feature of the scenario that inhibited the peaking latent-quadratic model.

Supplementary 9. Optimistic Bias

In this work we found that predicted utility of the dose that was predicted optimal was often higher than the true utility at that dose. This was shown by the inaccuracy metric typically being greater than zero, and is referred to as an ‘optimistic bias’. This was true for all of the dose-optimisation approaches, please consider the problems of ‘regression to the mean’ and the ‘Stein Paradox’ for examples of why this should be expected even in direct comparison approaches to dose optimisation. The amount of optimism was decreased by increasing trial size. This ‘optimistic bias’ is clearly undesirable. However, we believe that this is neither an issue with the models or calibration, nor unique to modelling-based optimisation. In this supplementary section we show that

- Similar issues are observed in binomial direct comparison dose-optimsation approaches.
- Similar issues are observed in continuous direct comparison dose-optimsation approaches.
- Similar issues are observed in a heavily simplified modelling setting.
- Similar issues are observed in a simple physical modelling setting.

We also note that similar problems have previously been highlighted both in over modelling based optimisation problems, and in the continuous self-optimisation problems referred to as reinforcement learning. Methods for solving these problems involve either double-q learning [4], or using only half the data for optimisation and half for prediction, neither of which seem entirely reasonable given the small amount of data and parametric model forms involved in vaccine dose-optimisation.

Direct comparison: Binomial

Consider testing k vaccine doses and attempting to choose that which maximises a binary measure of vaccine efficacy. For each of doses (d1, d2,...,dk) n individuals receive that dose and a binary efficacy outcome is recorded depending on the true probability of efficacy for that dose, $\bar{p}=(p_1, p_2,..., p_k)$.

These are used to estimate the probability of efficacy for each dose, ep_1, ep2, …, ep_k. Predicted optimal dose is then d_i such that $ep_i >= ep_j$ for all j in 1 to k, breaking ties at random. This is the basic direct comparison approach.

After conducting this procedure, one of 3 things can happen.

- $p_i < ep_i$ (overestimation)
- $p_i = ep_i$ (accurate estimation)

- $p_i > ep_i$ (under estimation)

We show that typically the first ($p_i < ep_i$) can be most common, which is to say that optimistic bias is observed when simulated. As an explicit example, consider k = 2, $\bar{p}$=(0.5,0.5), n = 10. A reasonable example efficacy observation for these doses respectively given these probabilities could be (6/10, 4/10). The dose with 6/10 efficacies would be predicted to be most likely to be optimal, and the best estimate of efficacy probability would be 0.6, an overestimation. Hence, whilst these observations were unbiased, the estimate for the optimal dose was optimistically biassed.

See below tables for different simulation observations. Note that this bias is made worse by increasing the number of possible doses, and by the query doses having more similar true probabilities

k	$\bar{p}$	n	Overestimation	Accurate Estimation	Underestimation
2	(0.5, 0.5)	10	61999	24774	14027
2	(0.5, 0.5)	100	70910	8017	21073
2	(0.6, 0.7)	10	59076	25649	15275
2	(0.6, 0.7)	100	53007	8579	38414
2	(0.1, 0.9)	10	34975	38748	26277
2	(0.1, 0.9)	100	45254	13238	41508
3	(0.5, 0.5, 0.5)	10	75665	18956	5379
3	(0.5, 0.5, 0.5)	100	75665	18956	5379
3	(0.5,0.6, 0.7)	20	65322	17433	17245
3	(0.1, 0.1, 0.5)	10	40845	24764	34391
3	(0.0, 0.0, 0.5)	10	37552	24683	37765

Table.S.OptimisticBias.1. Overestimation/Underestimation results from 100,000 simulated clinical trials.

This can also be considered a similar phenomenon to that of the multiple comparison problem.

Direct comparison: Continuous

Consider testing k vaccine doses and attempting to choose that which maximises a continuous, normally distributed measure of vaccine efficacy. For each of doses d1, d2,..., dk n individuals receive that dose and the continuous efficacy outcome is recorded from the true p.d.f of the efficacy response. This is defined by normal distributions with respective means and standards deviations, m =(m1, m2,..., mk) and s =(s1, s2,..., sk).
These are used to estimate the mean value of the true efficacy variable for each dose, em1, em2, …, emk. Predicted optimal dose is then di such that emi >=epm for all j in 1 to k, breaking ties at random. This is the basic direct comparison approach.

After conducting this procedure, one of 3 things can happen.

- mi<emi (overestimation)
- mi=emi (accurate estimation) - effectively impossible unless censored to a number of significant places.
- mi>emi (under estimation)

Again we see that the first is most likely. This is because we have for each dose di,

$$em_i = m_i + \epsilon_i,\ with\ \epsilon_i \sim N(0, \sigma)$$

Where ϵ_i is the ith error term.
Hence for the selection process (maximising em_i) we are not only maximising m_i, but also ϵ_i, hence we are more likely to select doses which are overestimated.

See below tables for different observations. Note again that this bias is made worse by these doses having more similar true probabilities. We only show k=2 examples here, but again increasing k exacerbates the issue.

k	m	s	n	Overestimation	Underestimation
2	(10,10)	(2,2)	10	75113	24887
2	(10,10)	(2,2)	100	75126	24874
2	(10,9)	(2,2)	10	61422	38578
2	(10,9)	(2,2)	100	50177	49823

2	(10,10)	(4,4)	10	75113	24887
2	(10,10)	(4,4)	100	75126	24874
2	(10,9)	(4,4)	10	70636	29364
2	(10,9)	(4,4)	100	53819	46181
2	(50,20)	(2,2)	10	49919	50081
2	(50,20)	(2,2)	100	50152	49848

Table.S.OptimisticBias.2. Overestimation/Underestimation results from 100,000 simulated clinical trials. Figures of these results in Figure.S.OptimisticBias.1

n = 10	n = 100
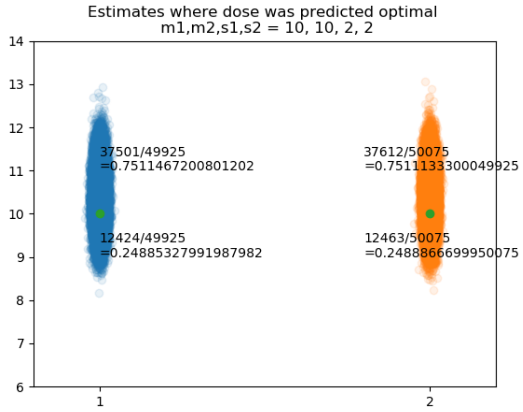	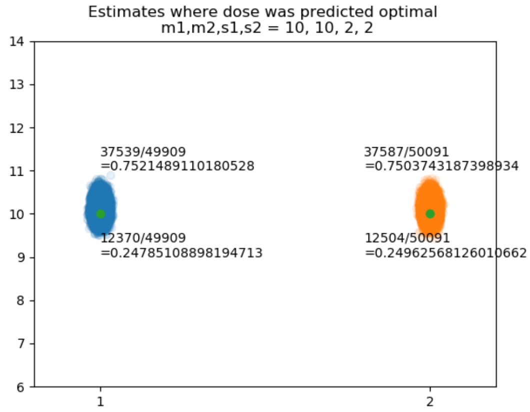
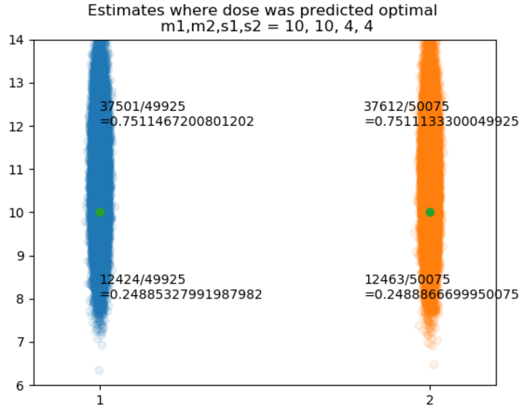	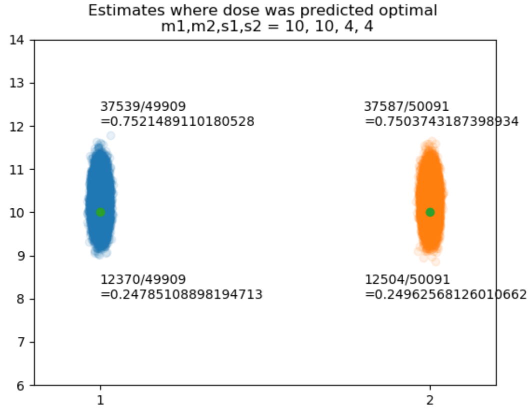

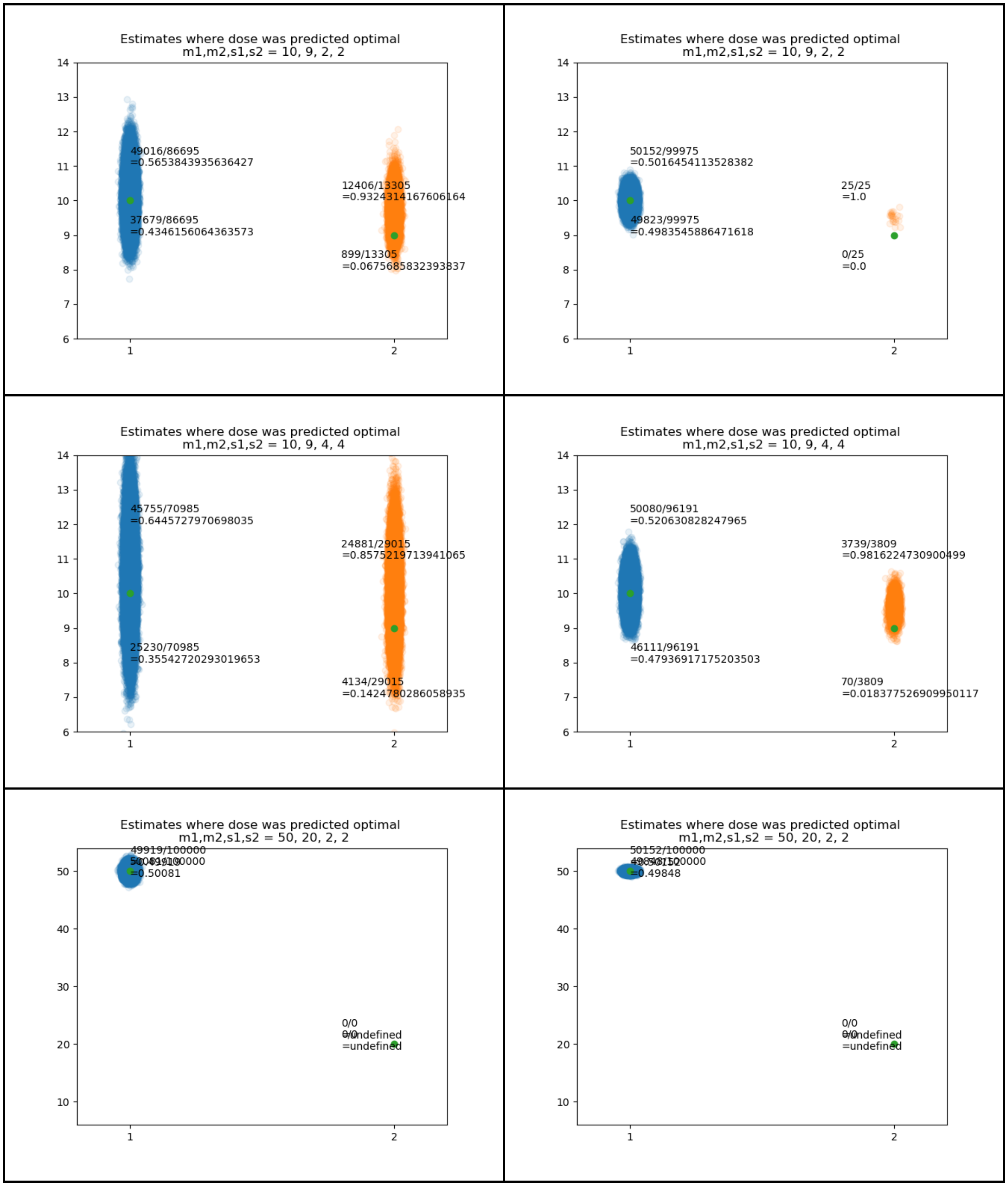

Figure.S.OptimisticBias.1. Each point represents a clinical trial where that dose (1 or 2) was selected, and em_i for that trial. The green dots are the true m_1/m_2 values. Note that typically the clinical trial dots are likely to be greater than the respective true value dots.

Simplified modelling

We show that it is intuitive that overestimation may occur in an example simplified model case. Consider again an example of trying to optimise some continuous function of dose-utility. We use a modified bell curve as the parameters are more interpretable, and assume that both the true function and assumed function follow this. The parameters of this function are midpoint, maximum, and scale. Midpoint is the point where the peak of the dose-utility curve occurs, maximum is the value of utility for this dose, and scale widens or shrinks the bell curve around this point. Say the true curve is defined by:

$Midpoint_{true} = 5$
$Maximum_{true} = 3$
$Scale_{true} = 1$

And thus looks like:

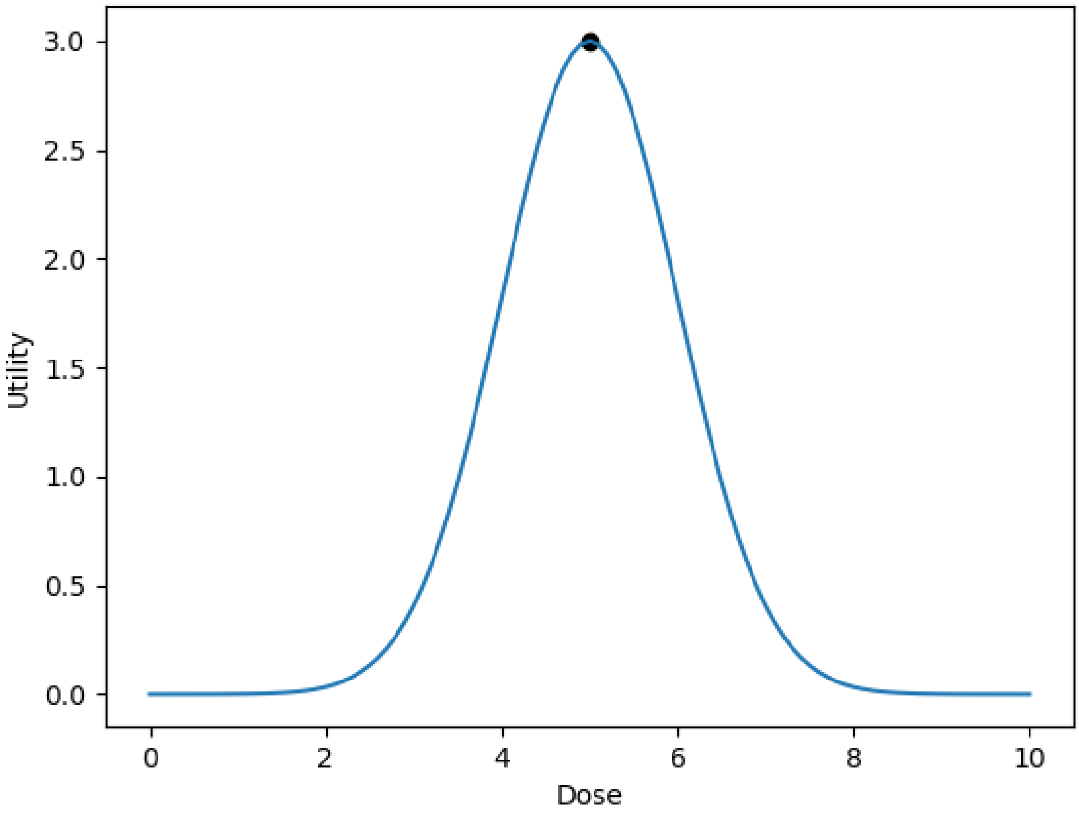

Figure.S.OptimisticBias.2. True Dose Utility Curve in toy example. Black point represents optimal dose and response.

An experiment is conducted, and estimates are calculated for these model parameters

$Midpoint_{estimate} = Midpoint_{true} + error_{midpoint}$

$Maximum_{estimate} = Maximum_{true} + error_{maximum}$

$Scale_{estimate}$ = Scaletrue + $error_{scale}$

Where each estimate is normally distributed around the true value (eg $error_i \sim N(0,\sigma_i)$)

Results of underestimating/overestimating each of these values individually are shown in the figure below. An overestimation would occur in 3 of the 6 cases, and an underestimation in only 1 of the 6. See

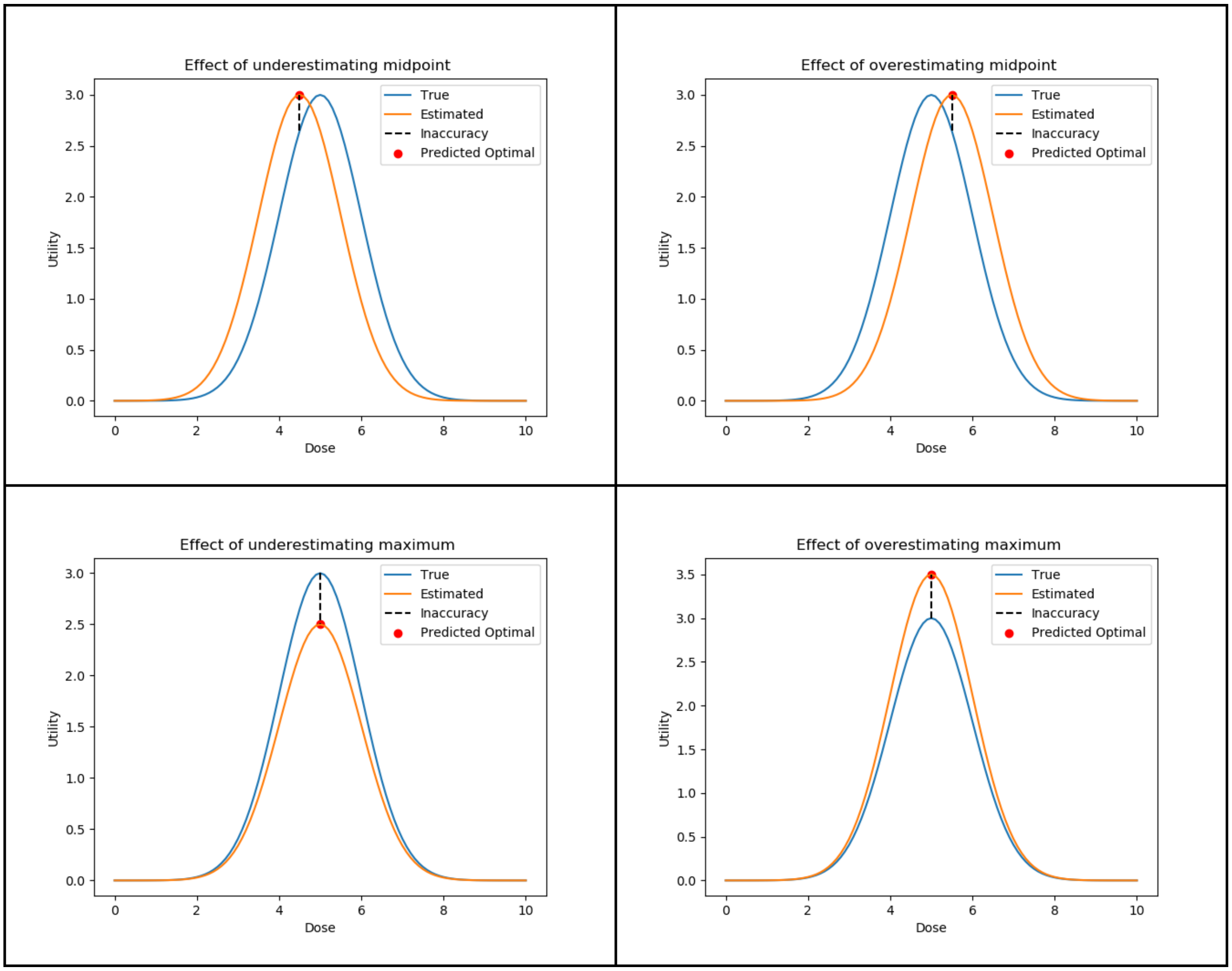

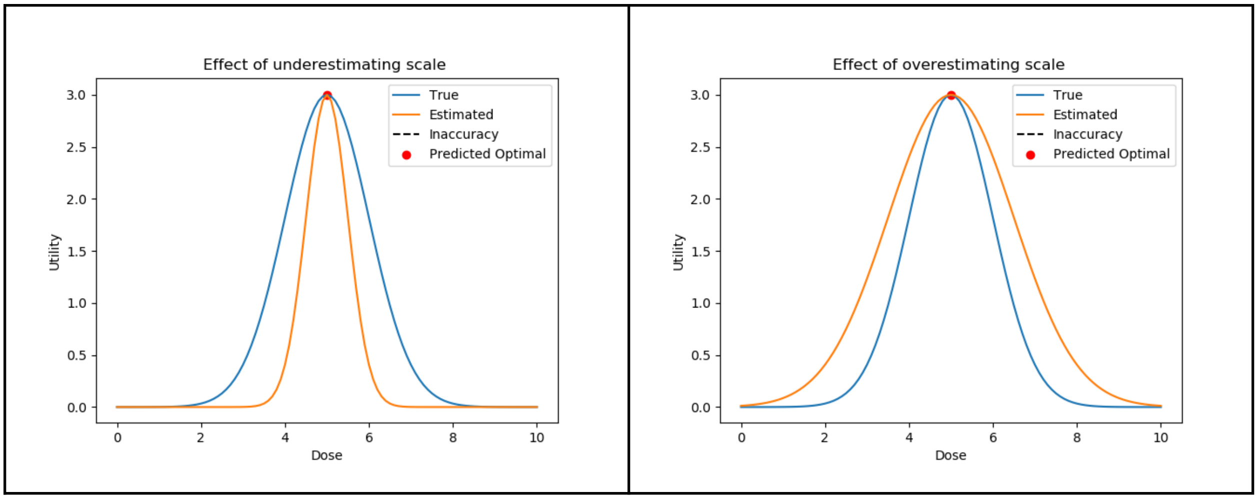

Figure.S.OptimisticBias.3. Demonstrations for how for some models overestimation can arise from parameter uncertainty.

Running simulations of 10000 clinicals trials with σ_i=0.5 for all parameters, we found that in 68.63% of simulation there was overestimation. The figure below should suggest why the quasi-convex nature of the dose-utility curve might lead to overestimation even if the parameter errors are normally distributed around the true value (unbiased) and the distribution of predicted optimal dose/response is a multivariate normal around the true value.

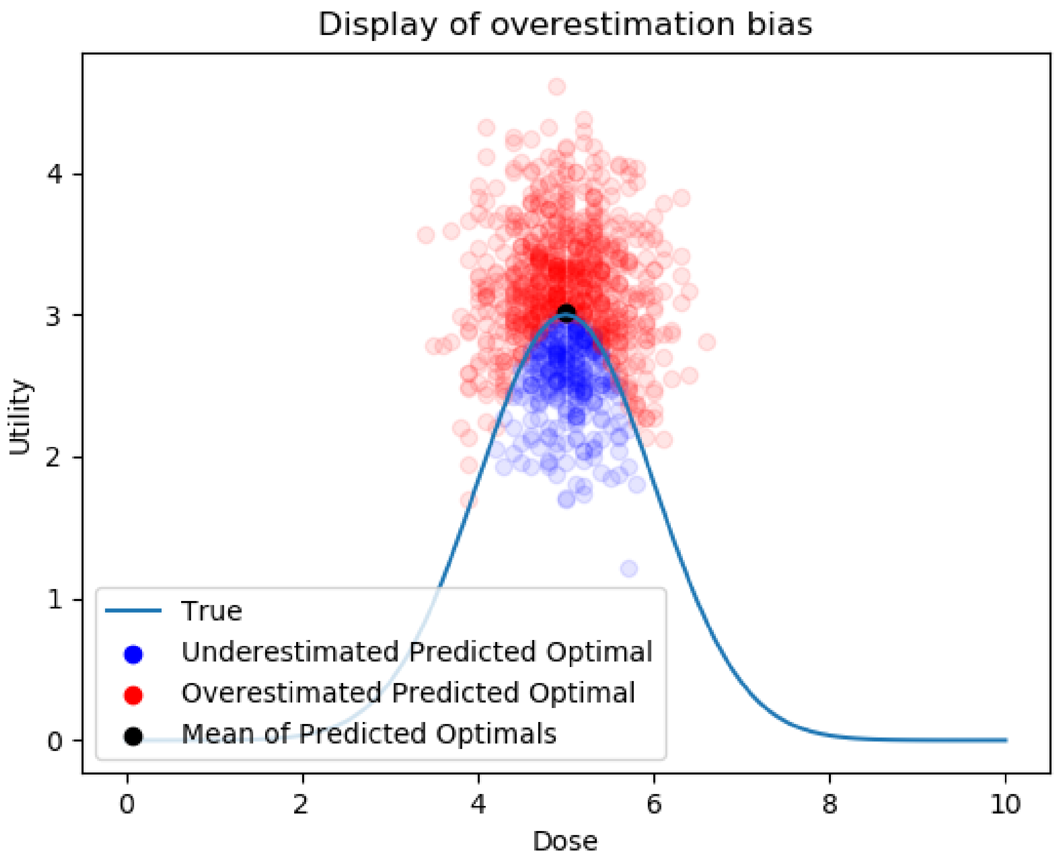

Figure.S.OptimisticBias.4. Each point represents a 'clinical trial' where instead of considering uncertainty in parameters we just assume that the predicted optimal dose and predicted optimal response are normally distributed around the true optimal. In this case the quasi-convex shape of the utility curve ensures that there is an optimistic bias.

We repeated the simulation of clinical trials with parameter uncertainty for σ_i between 0 and 0.5, and we found that increasing the σ_i increases the overestimation bias, but that at least some bias is observed for all σ_i>0.

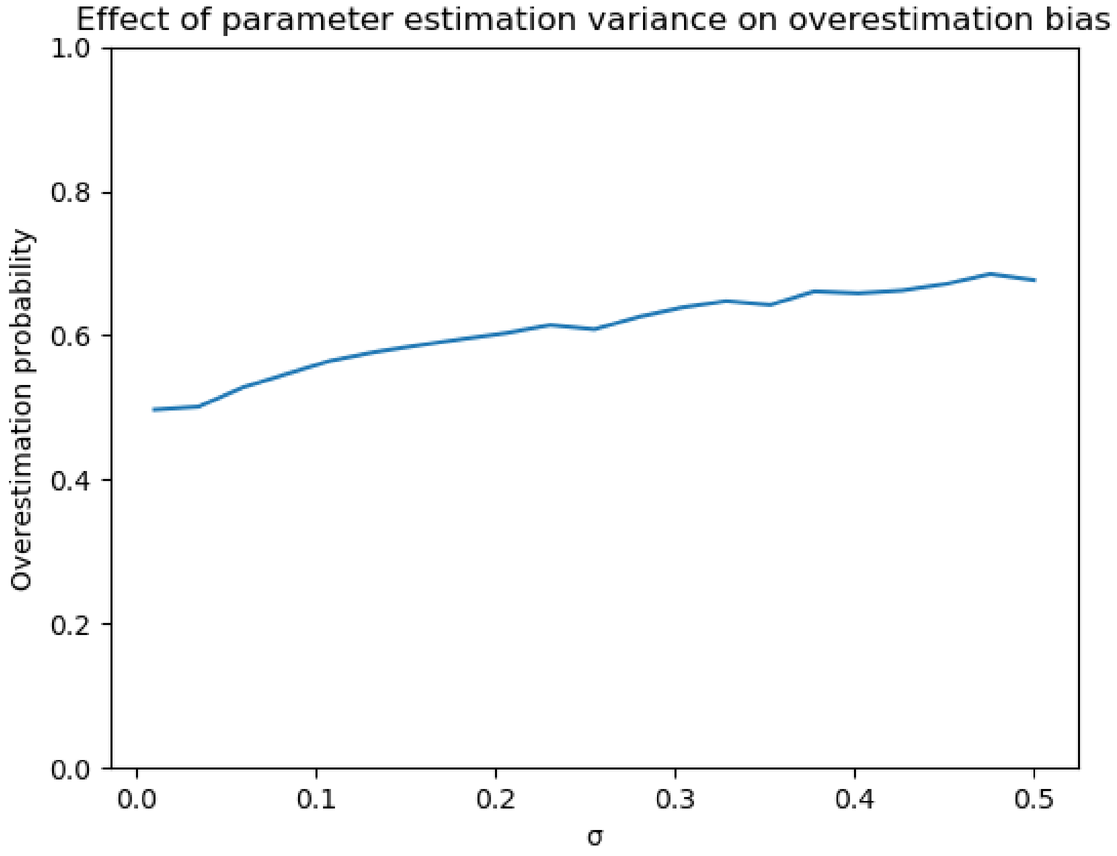

Physical Modelling

To take a break from questions of dose to touch on a different example of a non-trivial optimisation problem. Consider the toy problem of attempting to choose optimal battery size for an unmanned aerial vehicle ('drone'), where 'optimal' means the battery such that the drone can fly furthest. A heavily simplified physical model is built, see figure below.

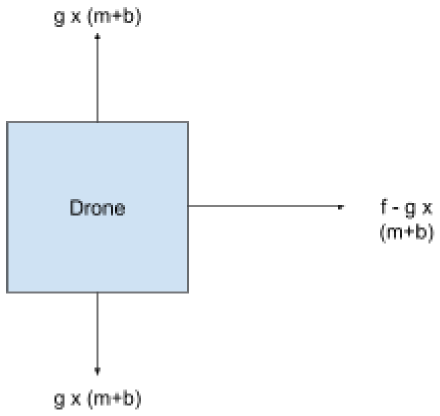

Figure S.OverestimationBias.Drone2. A visual depiction of the model forces of drone flight used in this section.

The distance the drone flies is determined by the following 5 parameters.

- g: the acceleration due to gravity
- m: The base mass of the drone
- a: drain rate, the rate at which the battery drains
- f: The force that the drone is capable of generating to fly (will be divided between maintaining height and moving forward)
- b: the size of the battery, non-dimensionalised to reflect both mass and capacity of the battery.

Increasing b increases the time taken to drain the battery, but also increases the weight of the drone, reducing the amount of force that can be used to move forward.

It can be shown that the distance travelled s given g,m,a,f, and b is $s = \frac{(f-g(m+b))b}{(m+b)a}$ and hence that optimal b is found for $\frac{ds}{db} = 0$ and hence the optimal b is equal to $max(-m - \sqrt{f * m/g}, -m + \sqrt{f * m/g})$.

A researcher estimates g, m, a, and f from available data, the results of which are normally distributed around the true values with some error scale. That is to say, with $error_i$ ~ N(0, error_scale)

$g_{estimate} = g_{true} + error_g$
$m_{estimate} = m_{true} + error_m$
$a_{estimate} = a_{true} + error_a$
$f_{estimate} = f_{true} + error_f$

These estimations are then used to predict optimal battery size and hence the predicted maximal distance the drone should travel with that battery size. We again show that there is an overestimation bias observed that again depends on the variance of the parameters estimates from the true values.

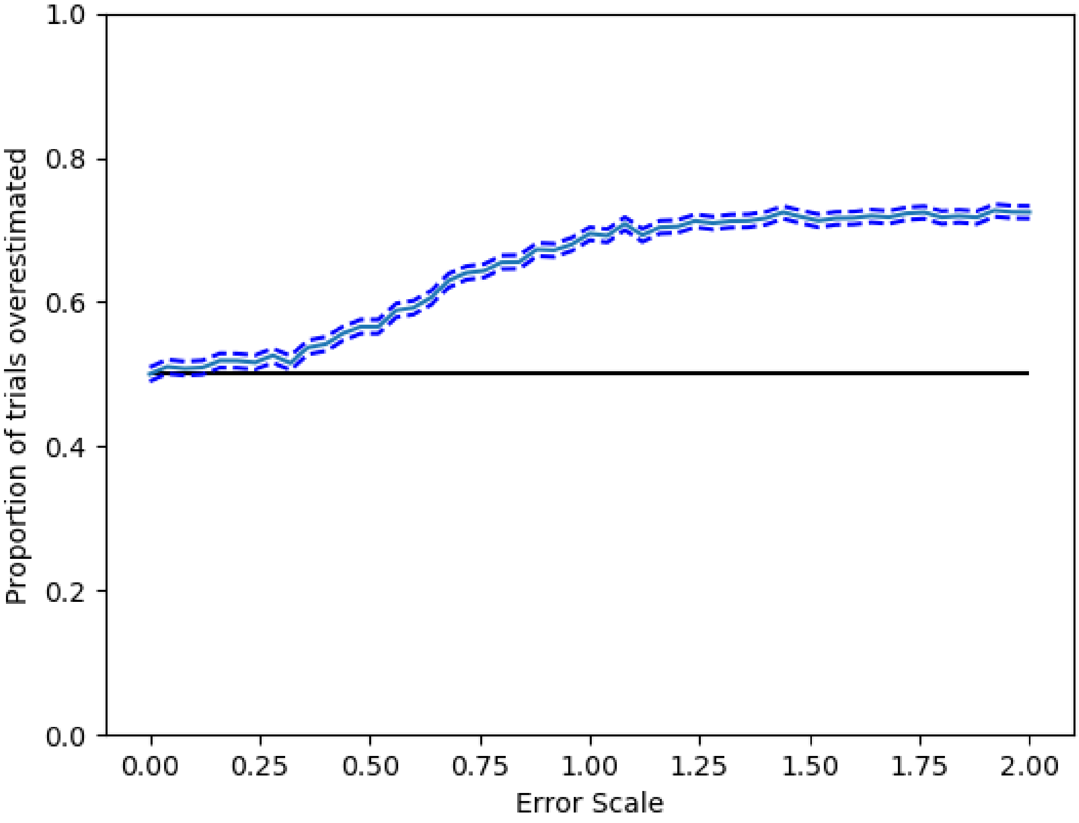

Figure S.OverestimationBias.Drone2. The effect of increasing the variance of normally distributed errors in parameters for the drone optimisation problem. 10,000 simulations of parameter estimation, optimisation, and comparison to true flight distance for the chosen battery size were done for each error scale value. 95% confidence bounds on the overestimation proportion are in dashed blue.

Supplementary 10. Plotted Clinicals Trial Results

Scenario Saturating 1

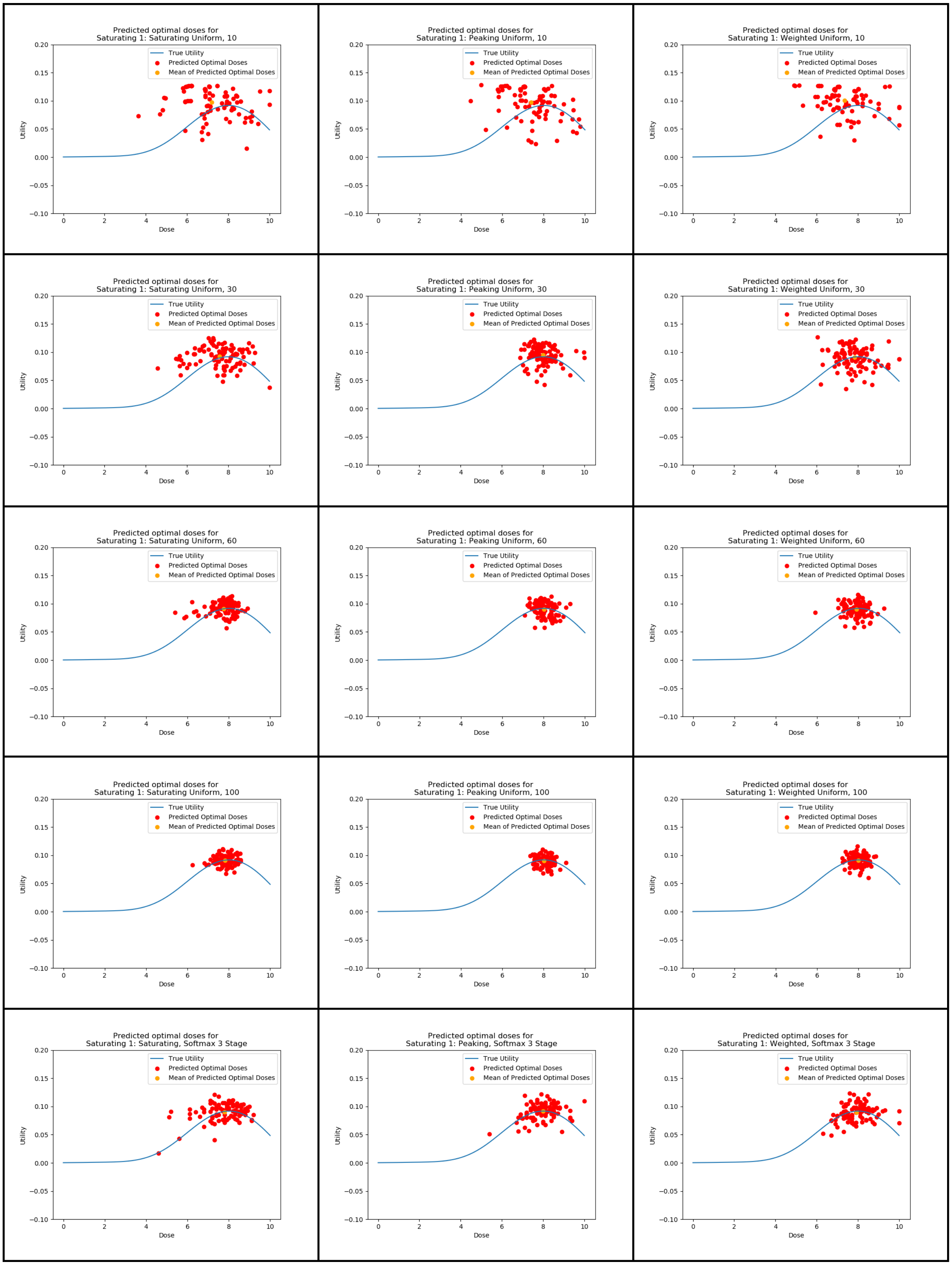

Predicted optimal doses for
Saturating 1: Saturating Uniform, 10
Predicted optimal doses for
Saturating 1: Peaking Uniform, 10
Predicted optimal doses for
Saturating 1: Weighted Uniform, 10
Predicted optimal doses for
Saturating 1: Saturating Uniform, 30
Predicted optimal doses for
Saturating 1: Peaking Uniform, 30
Predicted optimal doses for
Saturating 1: Weighted Uniform, 30
Predicted optimal doses for
Saturating 1: Saturating Uniform, 60
Predicted optimal doses for
Saturating 1: Peaking Uniform, 60
Predicted optimal doses for
Saturating 1: Weighted Uniform, 60
Predicted optimal doses for
Saturating 1: Saturating Uniform, 100
Predicted optimal doses for
Saturating 1: Peaking Uniform, 100
Predicted optimal doses for
Saturating 1: Weighted Uniform, 100
Predicted optimal doses for
Saturating 1: Saturating, Softmax 3 Stage
Predicted optimal doses for
Saturating 1: Peaking, Softmax 3 Stage
Predicted optimal doses for
Saturating 1: Weighted, Softmax 3 Stage
True Utility
Predicted Optimal Doses
Mean of Predicted Optimal Doses
Utility
Dose

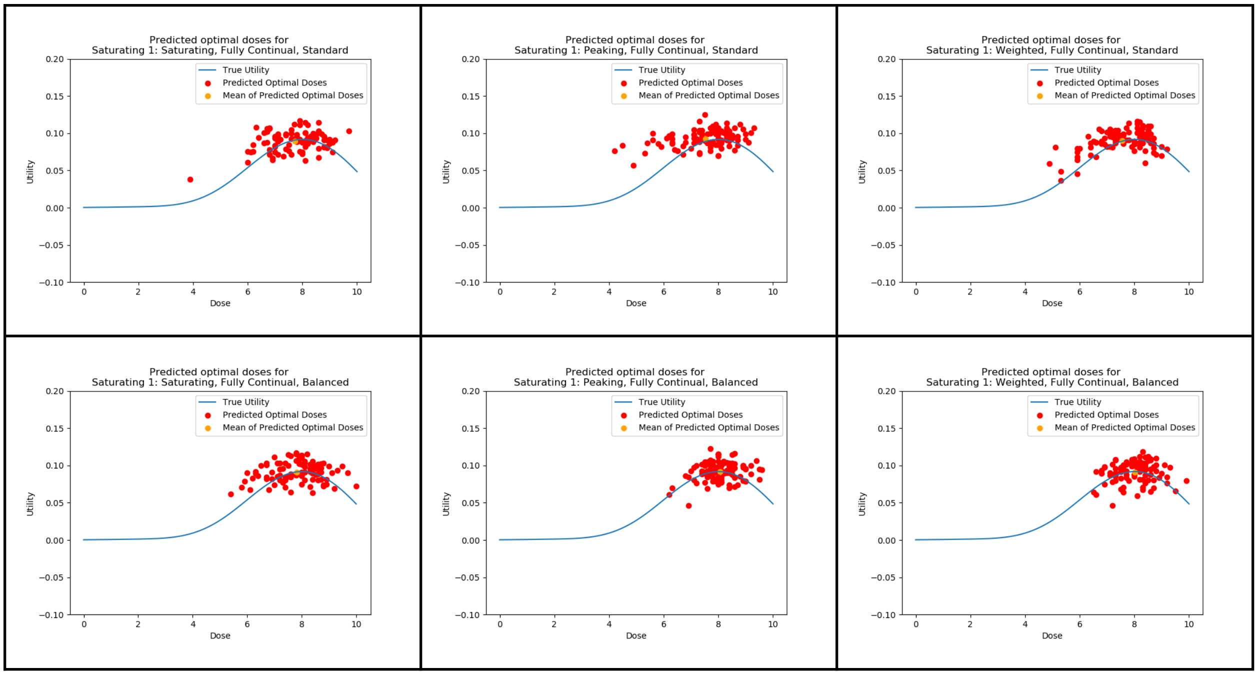

Fig.S.ClinTrialPlots.S1. Plots of true utility curves and predicted optimal/dose response for 100 simulations of each approach for this scenario. Approaches use a saturating (left), peaking(middle), or weighted (right) efficacy curve. From top to bottom; trial size is 10, 30, 60, 100, 30, 30, 30. Method of trial dose selection from top to bottom in uniform, uniform, uniform, uniform, softmax 3 stage. Standard fully continual, and balanced fully continual.

Scenario Saturating 2

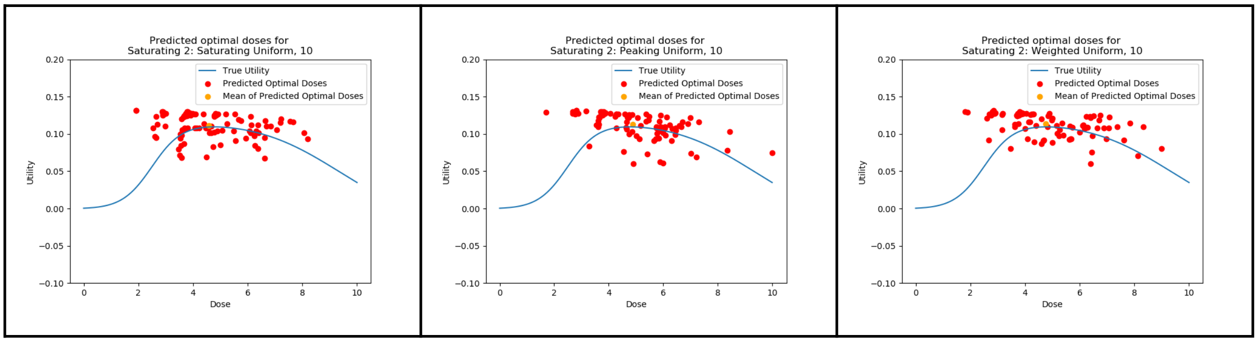

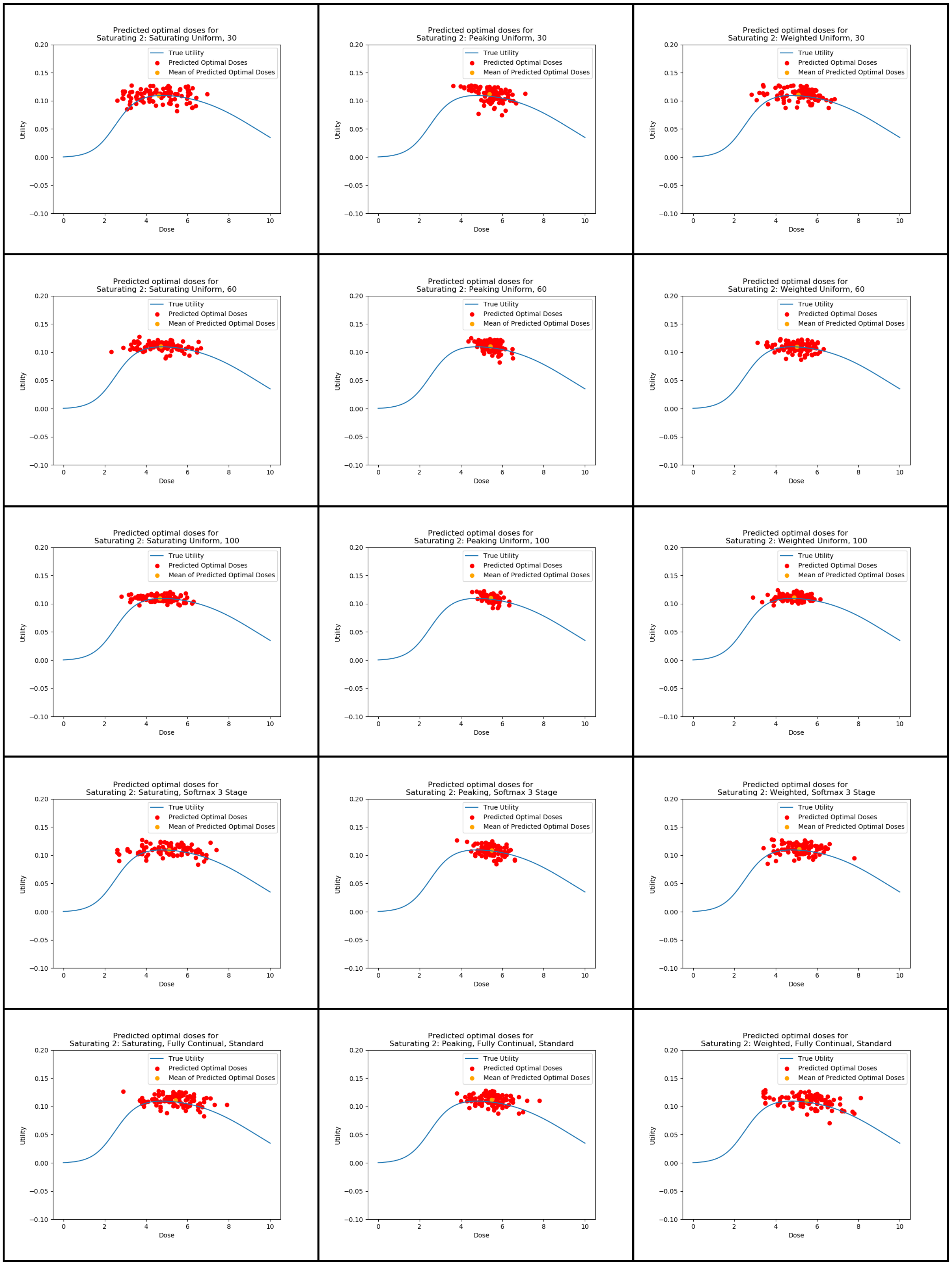
Predicted optimal doses for
Saturating 2: Saturating Uniform, 30
Predicted optimal doses for
Saturating 2: Peaking Uniform, 30
Predicted optimal doses for
Saturating 2: Weighted Uniform, 30
Predicted optimal doses for
Saturating 2: Saturating Uniform, 60
Predicted optimal doses for
Saturating 2: Peaking Uniform, 60
Predicted optimal doses for
Saturating 2: Weighted Uniform, 60
Predicted optimal doses for
Saturating 2: Saturating Uniform, 100
Predicted optimal doses for
Saturating 2: Peaking Uniform, 100
Predicted optimal doses for
Saturating 2: Weighted Uniform, 100
Predicted optimal doses for
Saturating 2: Saturating, Softmax 3 Stage
Predicted optimal doses for
Saturating 2: Peaking, Softmax 3 Stage
Predicted optimal doses for
Saturating 2: Weighted, Softmax 3 Stage
Predicted optimal doses for
Saturating 2: Saturating, Fully Continual, Standard
Predicted optimal doses for
Saturating 2: Peaking, Fully Continual, Standard
Predicted optimal doses for
Saturating 2: Weighted, Fully Continual, Standard
True Utility
Predicted Optimal Doses
Mean of Predicted Optimal Doses
Utility
Dose

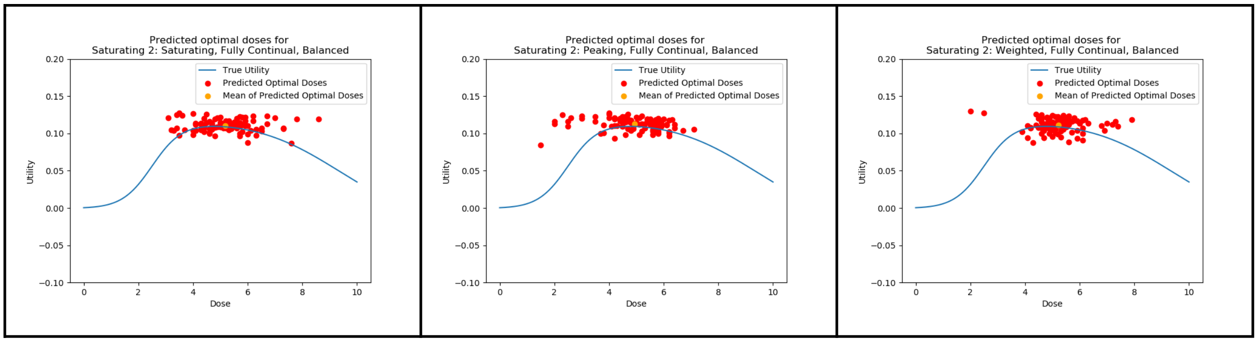

Fig.S.ClinTrialPlots.S2. Plots of true utility curves and predicted optimal/dose response for 100 simulations of each approach for this scenario. Approaches use a saturating (left), peaking(middle), or weighted (right) efficacy curve. From top to bottom; trial size is 10, 30, 60, 100, 30, 30, 30. Method of trial dose selection from top to bottom in uniform, uniform, uniform, uniform, softmax 3 stage. Standard fully continual, and balanced fully continual.

Scenario Saturating 3

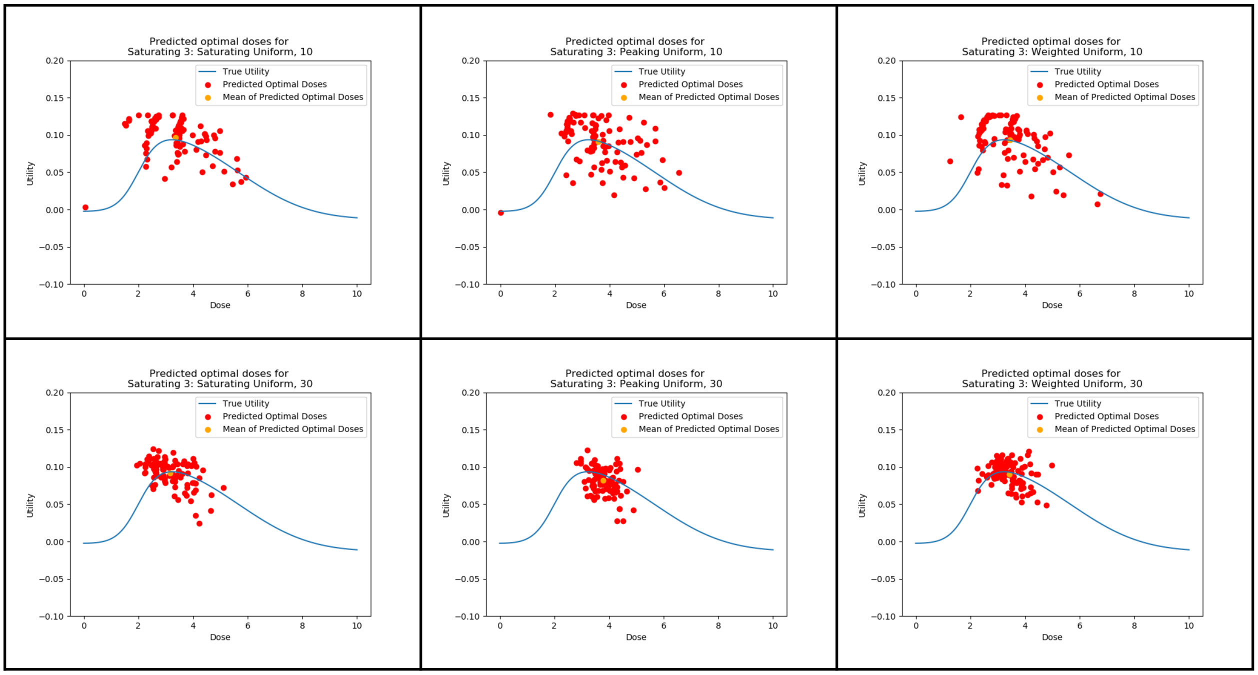

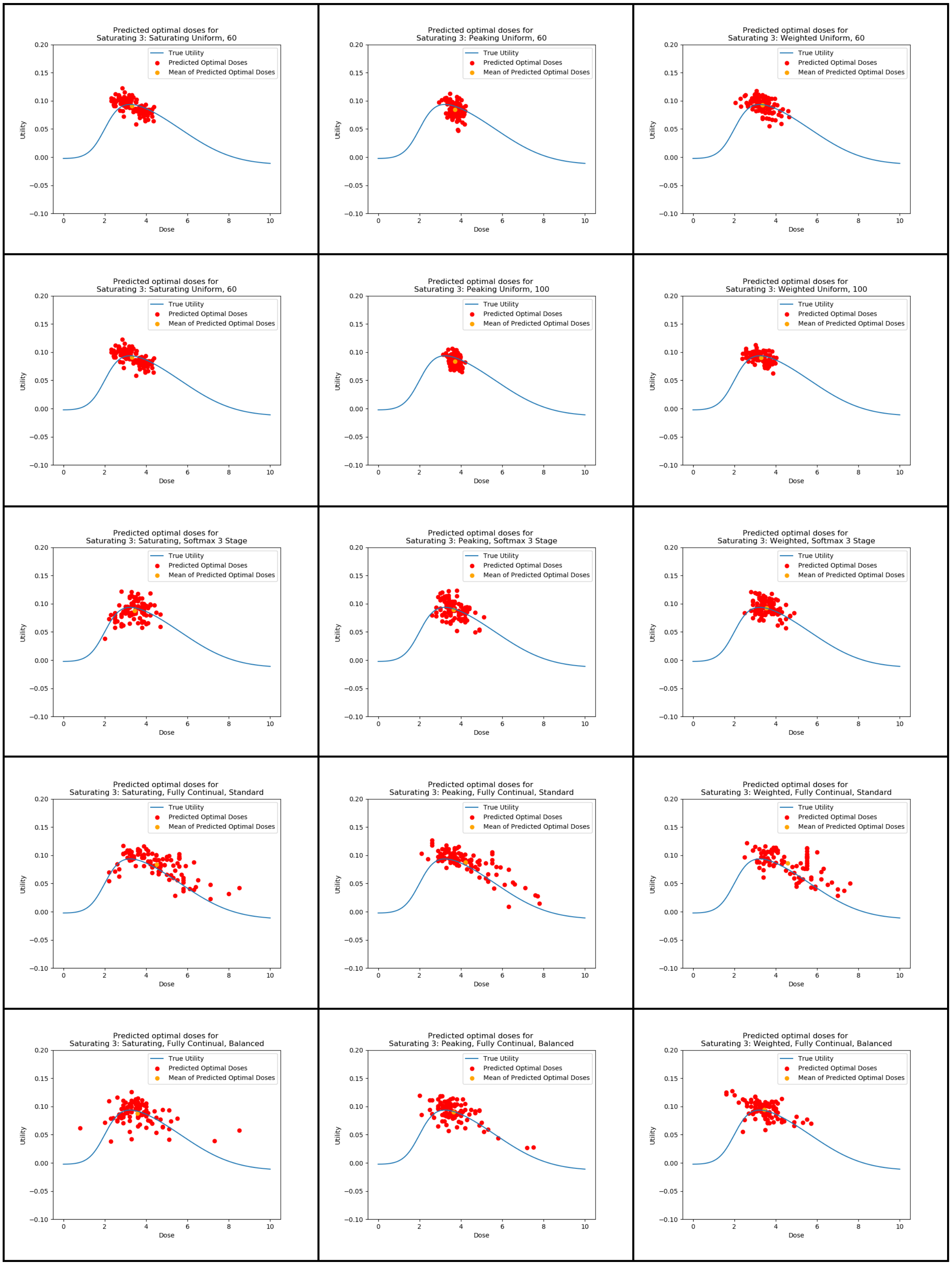
Predicted optimal doses for Saturating 3: Saturating Uniform, 60
Predicted optimal doses for Saturating 3: Peaking Uniform, 60
Predicted optimal doses for Saturating 3: Weighted Uniform, 60
Predicted optimal doses for Saturating 3: Saturating Uniform, 60
Predicted optimal doses for Saturating 3: Peaking Uniform, 100
Predicted optimal doses for Saturating 3: Weighted Uniform, 100
Predicted optimal doses for Saturating 3: Saturating, Softmax 3 Stage
Predicted optimal doses for Saturating 3: Peaking, Softmax 3 Stage
Predicted optimal doses for Saturating 3: Weighted, Softmax 3 Stage
Predicted optimal doses for Saturating 3: Saturating, Fully Continual, Standard
Predicted optimal doses for Saturating 3: Peaking, Fully Continual, Standard
Predicted optimal doses for Saturating 3: Weighted, Fully Continual, Standard
Predicted optimal doses for Saturating 3: Saturating, Fully Continual, Balanced
Predicted optimal doses for Saturating 3: Peaking, Fully Continual, Balanced
Predicted optimal doses for Saturating 3: Weighted, Fully Continual, Balanced
True Utility
Predicted Optimal Doses
Mean of Predicted Optimal Doses
Utility
Dose

Fig.S.ClinTrialPlots.S3. Plots of true utility curves and predicted optimal/dose response for 100 simulations of each approach for this scenario. Approaches use a saturating (left), peaking(middle), or weighted (right) efficacy curve. From top to bottom; trial size is 10, 30, 60, 100, 30, 30, 30. Method of trial dose selection from top to bottom in uniform, uniform, uniform, uniform, softmax 3 stage. Standard fully continual, and balanced fully continual.

Scenario Saturating 4

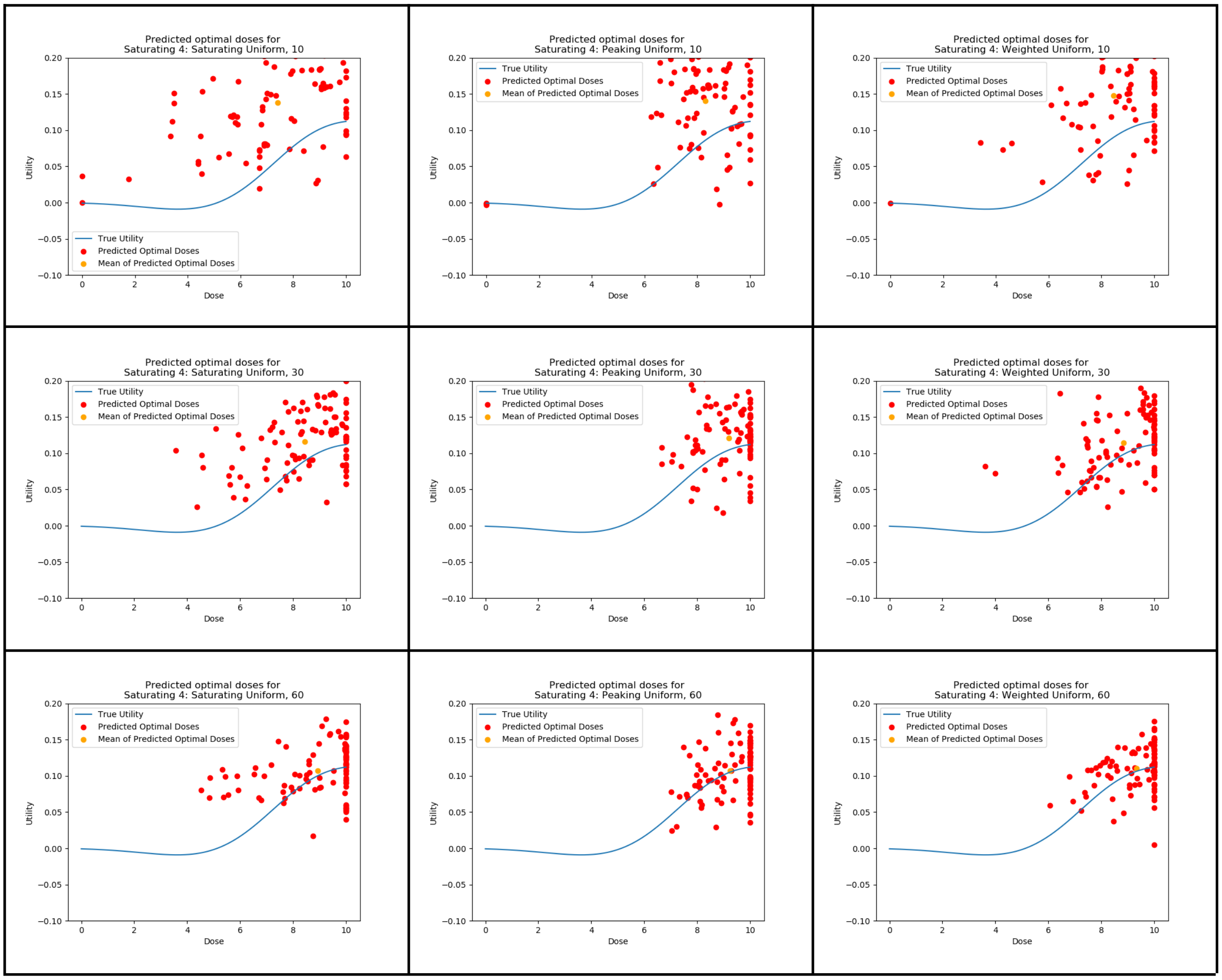

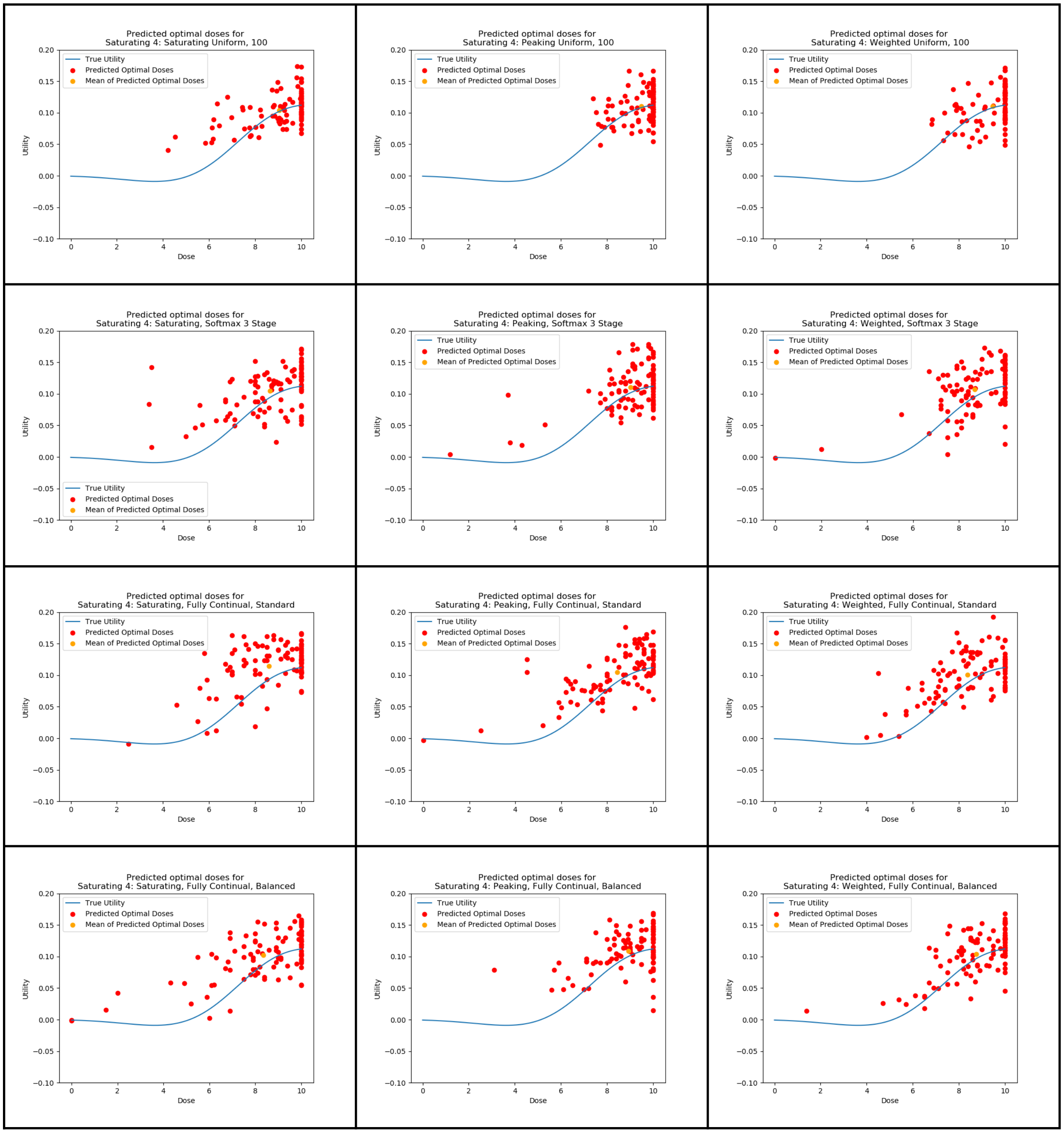

Fig.S.ClinTrialPlots.S4. Plots of true utility curves and predicted optimal/dose response for 100 simulations of each approach for this scenario. Approaches use a saturating (left), peaking(middle), or weighted (right) efficacy curve. From top to bottom; trial size is 10, 30, 60, 100, 30, 30, 30. Method of trial dose selection from top to bottom in uniform, uniform, uniform, uniform, softmax 3 stage. Standard fully continual, and balanced fully continual.

Scenario Saturating 5

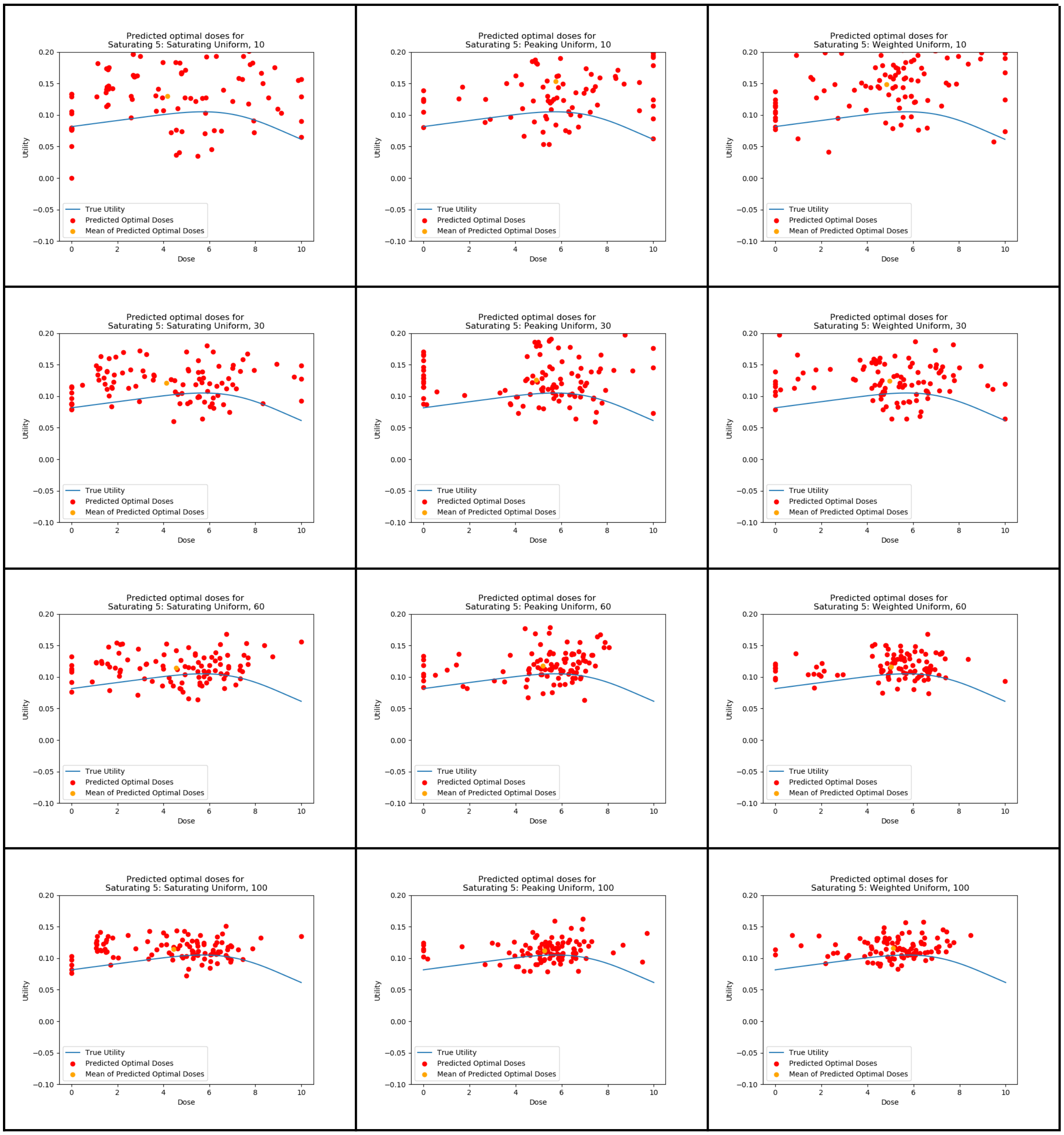

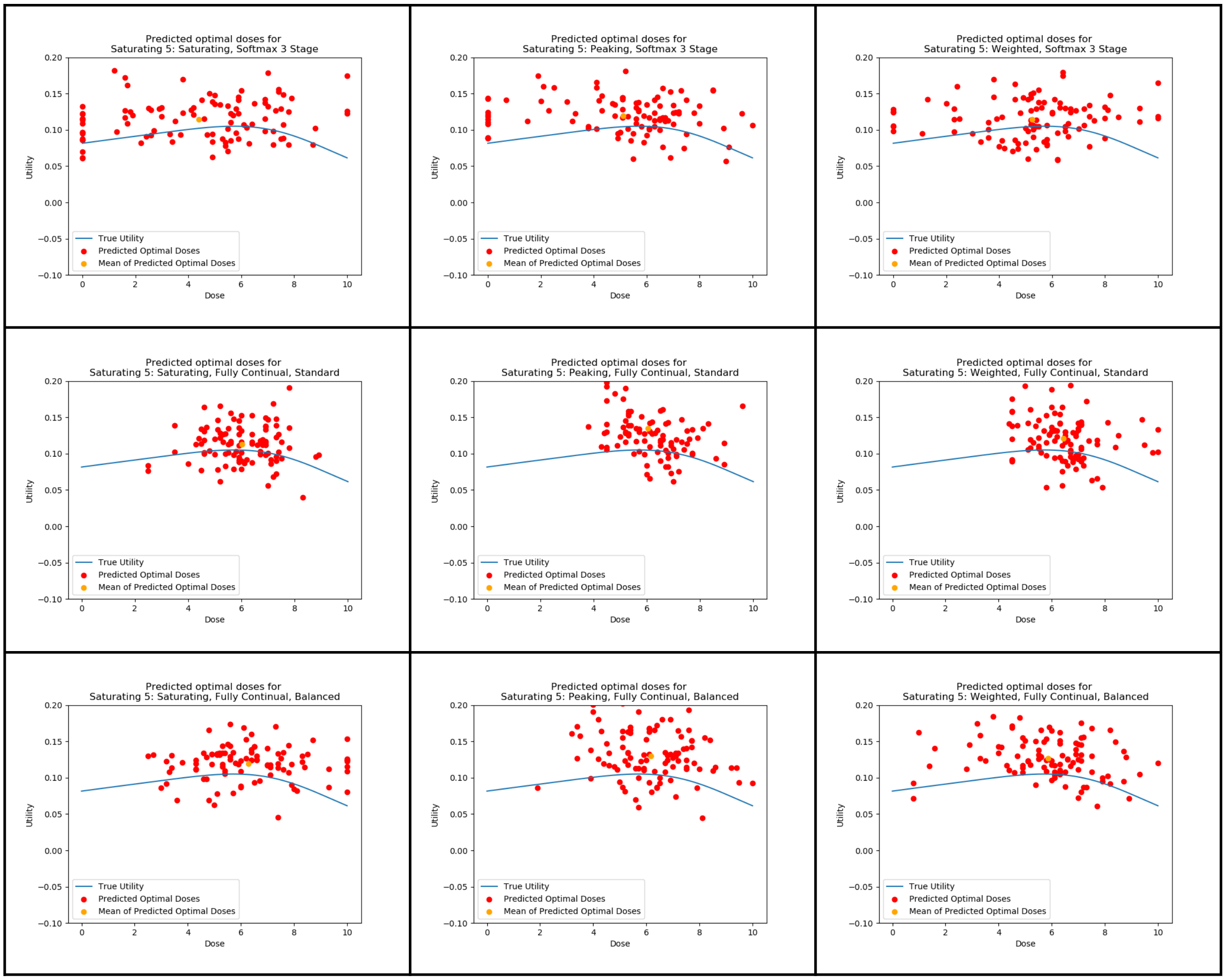

Fig.S.ClinTrialPlots.S5. Plots of true utility curves and predicted optimal/dose response for 100 simulations of each approach for this scenario. Approaches use a saturating (left), peaking(middle), or weighted (right) efficacy curve. From top to bottom; trial size is 10, 30, 60, 100, 30, 30, 30. Method of trial dose selection from top to bottom in uniform, uniform, uniform, uniform, softmax 3 stage. Standard fully continual, and balanced fully continual.

Scenario Peaking 1

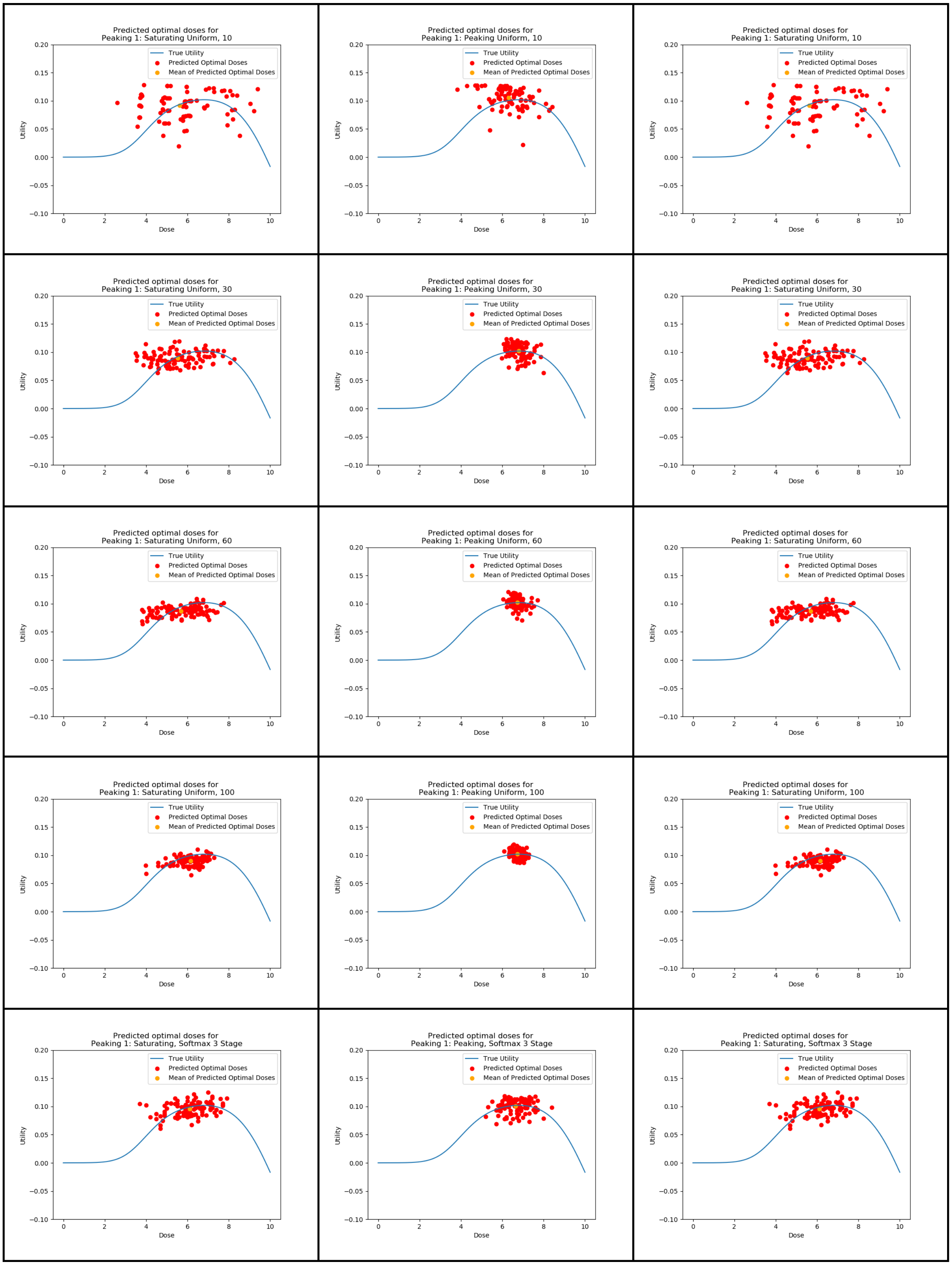
Predicted optimal doses for
Peaking 1: Saturating Uniform, 10
Predicted optimal doses for
Peaking 1: Peaking Uniform, 10
Predicted optimal doses for
Peaking 1: Saturating Uniform, 10
Predicted optimal doses for
Peaking 1: Saturating Uniform, 30
Predicted optimal doses for
Peaking 1: Peaking Uniform, 30
Predicted optimal doses for
Peaking 1: Saturating Uniform, 30
Predicted optimal doses for
Peaking 1: Saturating Uniform, 60
Predicted optimal doses for
Peaking 1: Peaking Uniform, 60
Predicted optimal doses for
Peaking 1: Saturating Uniform, 60
Predicted optimal doses for
Peaking 1: Saturating Uniform, 100
Predicted optimal doses for
Peaking 1: Peaking Uniform, 100
Predicted optimal doses for
Peaking 1: Saturating Uniform, 100
Predicted optimal doses for
Peaking 1: Saturating, Softmax 3 Stage
Predicted optimal doses for
Peaking 1: Peaking, Softmax 3 Stage
Predicted optimal doses for
Peaking 1: Saturating, Softmax 3 Stage
True Utility
Predicted Optimal Doses
Mean of Predicted Optimal Doses
Utility
Dose

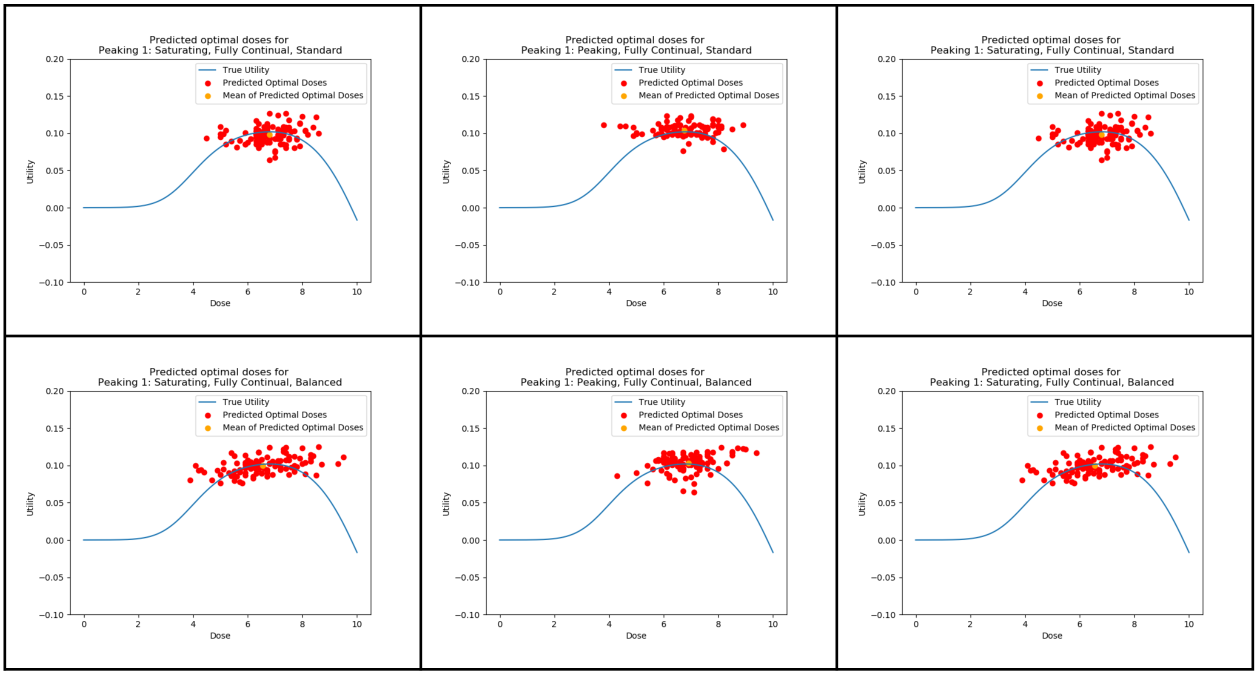

Fig.S.ClinTrialPlots.P1. Plots of true utility curves and predicted optimal/dose response for 100 simulations of each approach for this scenario. Approaches use a saturating (left), peaking(middle), or weighted (right) efficacy curve. From top to bottom; trial size is 10, 30, 60, 100, 30, 30, 30. Method of trial dose selection from top to bottom in uniform, uniform, uniform, uniform, softmax 3 stage. Standard fully continual, and balanced fully continual

Scenario Peaking 2

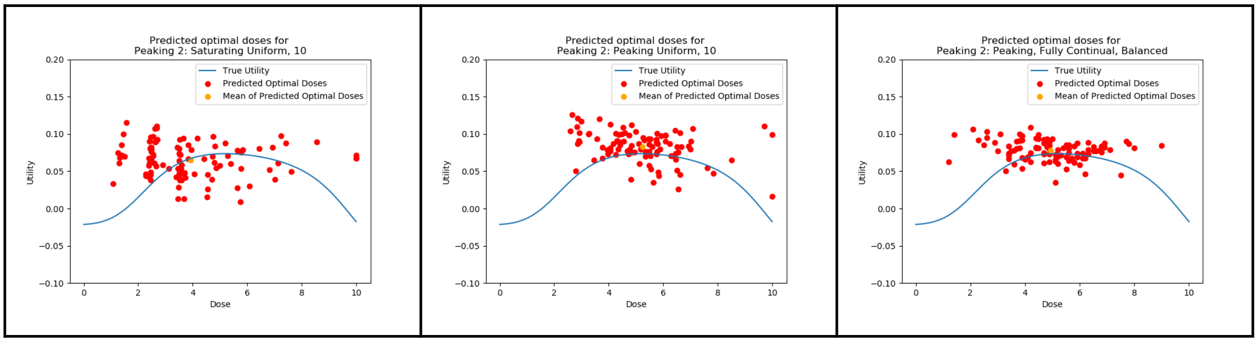

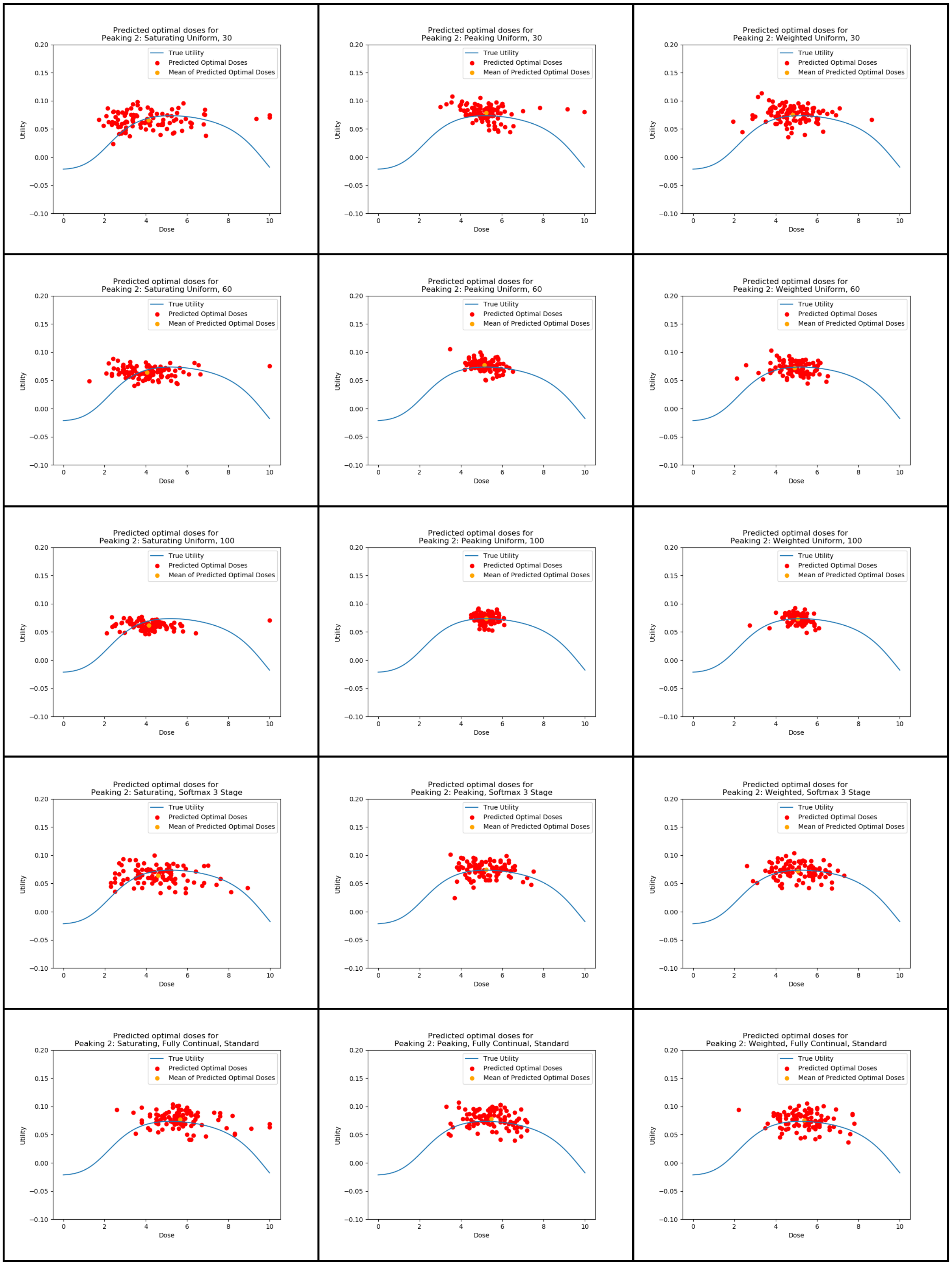
Predicted optimal doses for Peaking 2: Saturating Uniform, 30
Predicted optimal doses for Peaking 2: Peaking Uniform, 30
Predicted optimal doses for Peaking 2: Weighted Uniform, 30
Predicted optimal doses for Peaking 2: Saturating Uniform, 60
Predicted optimal doses for Peaking 2: Peaking Uniform, 60
Predicted optimal doses for Peaking 2: Weighted Uniform, 60
Predicted optimal doses for Peaking 2: Saturating Uniform, 100
Predicted optimal doses for Peaking 2: Peaking Uniform, 100
Predicted optimal doses for Peaking 2: Weighted Uniform, 100
Predicted optimal doses for Peaking 2: Saturating, Softmax 3 Stage
Predicted optimal doses for Peaking 2: Peaking, Softmax 3 Stage
Predicted optimal doses for Peaking 2: Weighted, Softmax 3 Stage
Predicted optimal doses for Peaking 2: Saturating, Fully Continual, Standard
Predicted optimal doses for Peaking 2: Peaking, Fully Continual, Standard
Predicted optimal doses for Peaking 2: Weighted, Fully Continual, Standard
True Utility
Predicted Optimal Doses
Mean of Predicted Optimal Doses
Utility
Dose

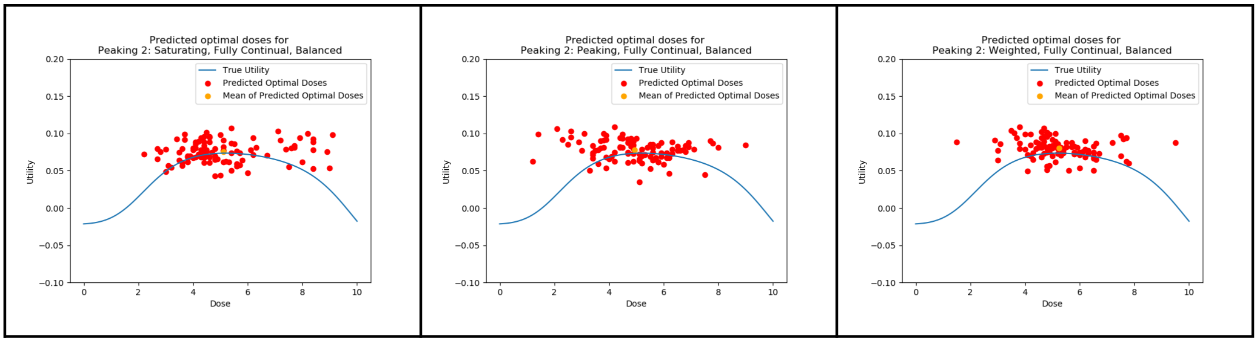

Fig.S.ClinTrialPlots.P2. Plots of true utility curves and predicted optimal/dose response for 100 simulations of each approach for this scenario. Approaches use a saturating (left), peaking(middle), or weighted (right) efficacy curve. From top to bottom; trial size is 10, 30, 60, 100, 30, 30, 30. Method of trial dose selection from top to bottom in uniform, uniform, uniform, uniform, softmax 3 stage. Standard fully continual, and balanced fully continual.

Scenario Peaking 3

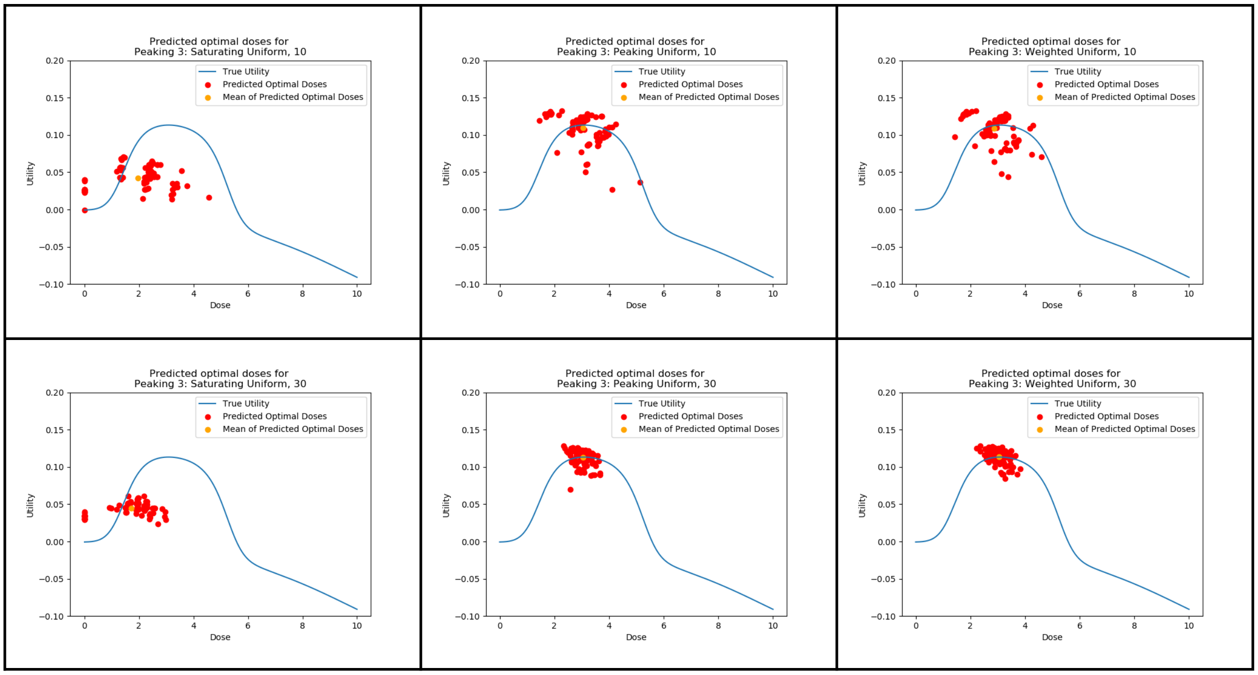

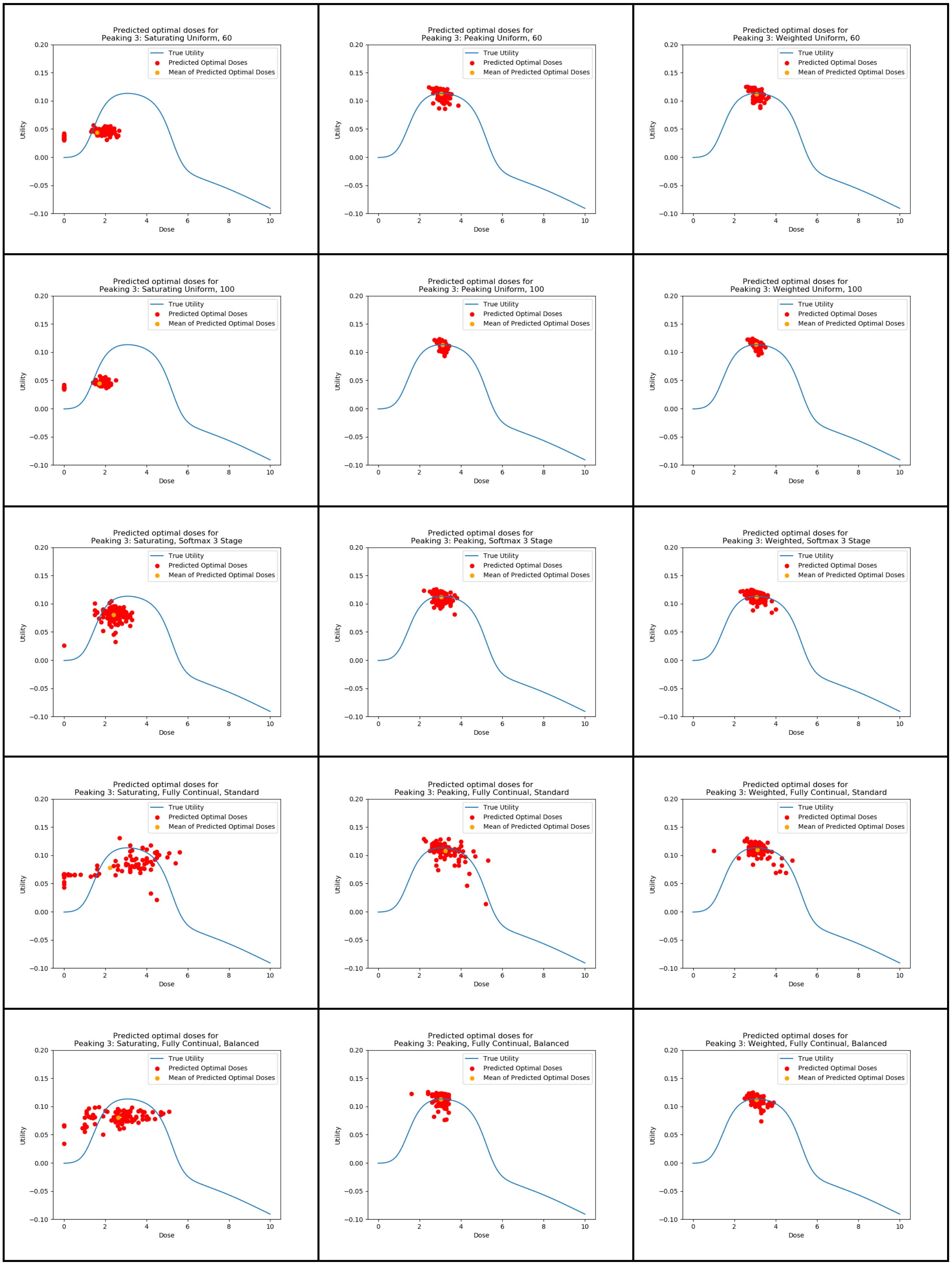
Predicted optimal doses for
Peaking 3: Saturating Uniform, 60
Predicted optimal doses for
Peaking 3: Peaking Uniform, 60
Predicted optimal doses for
Peaking 3: Weighted Uniform, 60
Predicted optimal doses for
Peaking 3: Saturating Uniform, 100
Predicted optimal doses for
Peaking 3: Peaking Uniform, 100
Predicted optimal doses for
Peaking 3: Weighted Uniform, 100
Predicted optimal doses for
Peaking 3: Saturating, Softmax 3 Stage
Predicted optimal doses for
Peaking 3: Peaking, Softmax 3 Stage
Predicted optimal doses for
Peaking 3: Weighted, Softmax 3 Stage
Predicted optimal doses for
Peaking 3: Saturating, Fully Continual, Standard
Predicted optimal doses for
Peaking 3: Peaking, Fully Continual, Standard
Predicted optimal doses for
Peaking 3: Weighted, Fully Continual, Standard
Predicted optimal doses for
Peaking 3: Saturating, Fully Continual, Balanced
Predicted optimal doses for
Peaking 3: Peaking, Fully Continual, Balanced
Predicted optimal doses for
Peaking 3: Weighted, Fully Continual, Balanced
True Utility
Predicted Optimal Doses
Mean of Predicted Optimal Doses
Dose
Utility

Fig.S.ClinTrialPlots.P3. Plots of true utility curves and predicted optimal/dose response for 100 simulations of each approach for this scenario. Approaches use a saturating (left), peaking(middle), or weighted (right) efficacy curve. From top to bottom; trial size is 10, 30, 60, 100, 30, 30, 30. Method of trial dose selection from top to bottom in uniform, uniform, uniform, uniform, softmax 3 stage. Standard fully continual, and balanced fully continual.

Scenario Peaking 4

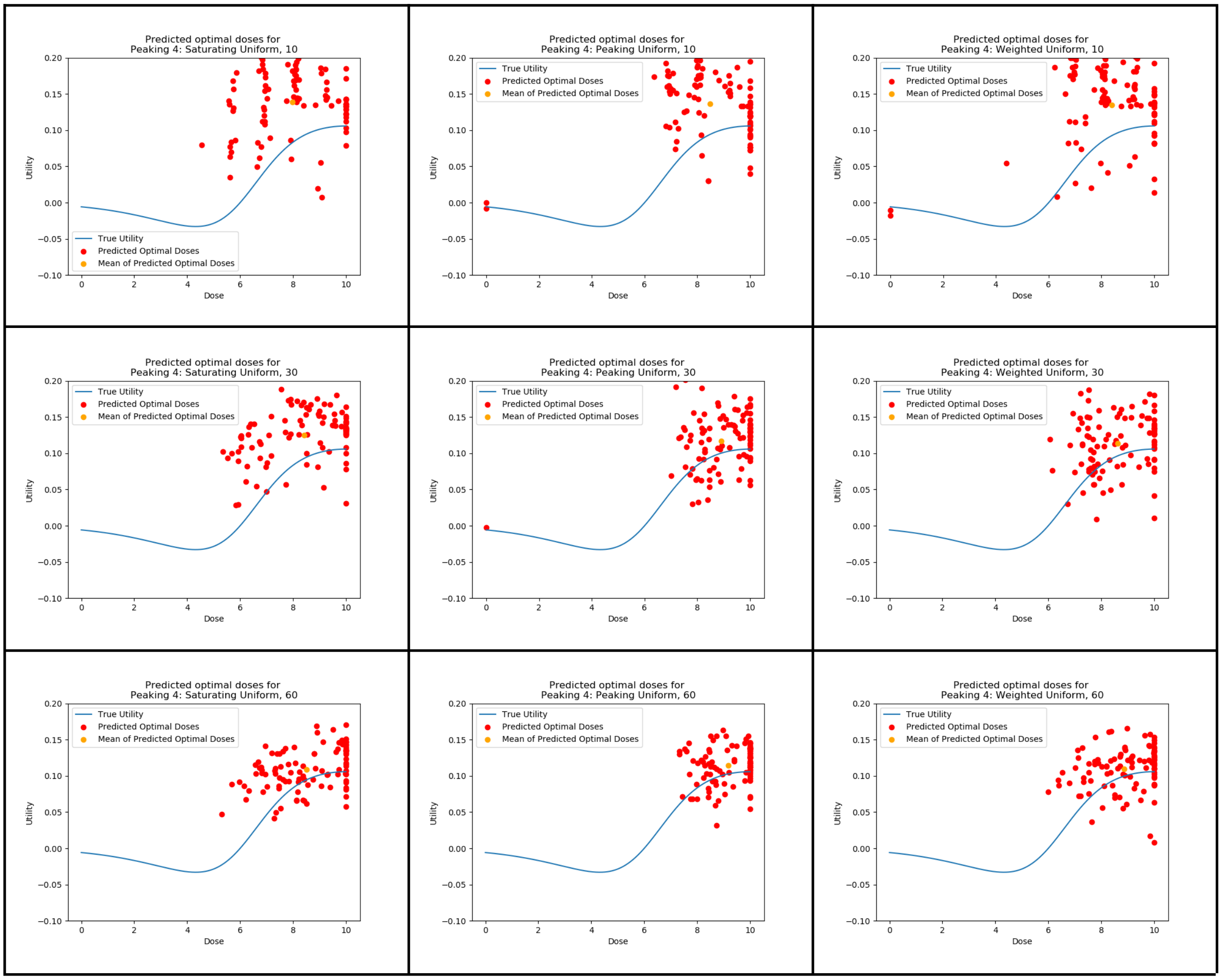

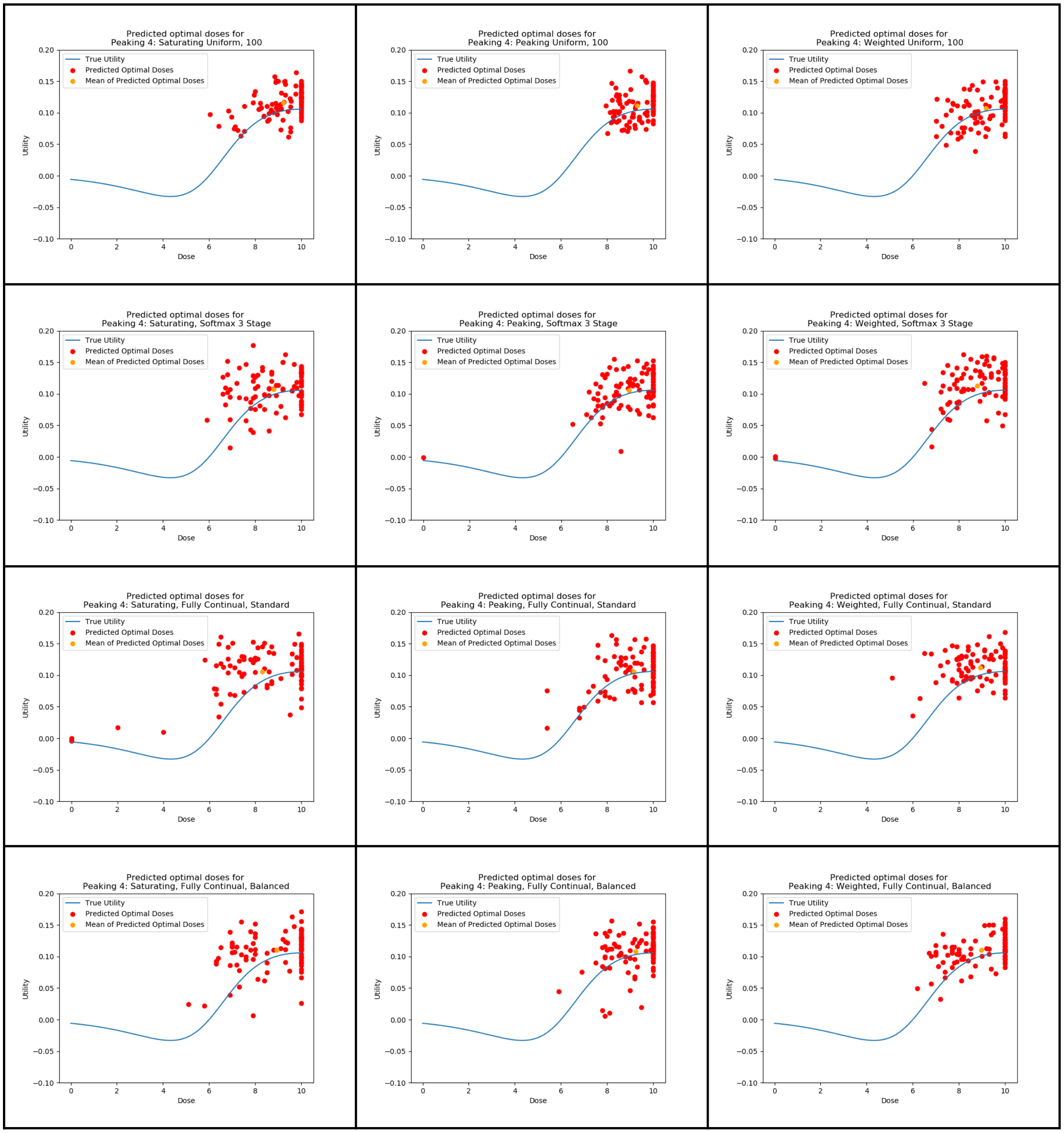

Fig.S.ClinTrialPlots.P4. Plots of true utility curves and predicted optimal/dose response for 100 simulations of each approach for this scenario. Approaches use a saturating (left), peaking(middle), or weighted (right) efficacy curve. From top to bottom; trial size is 10, 30, 60, 100, 30, 30, 30. Method of trial dose selection from top to bottom in uniform, uniform, uniform, uniform, softmax 3 stage. Standard fully continual, and balanced fully continual.

Scenario Peaking 5

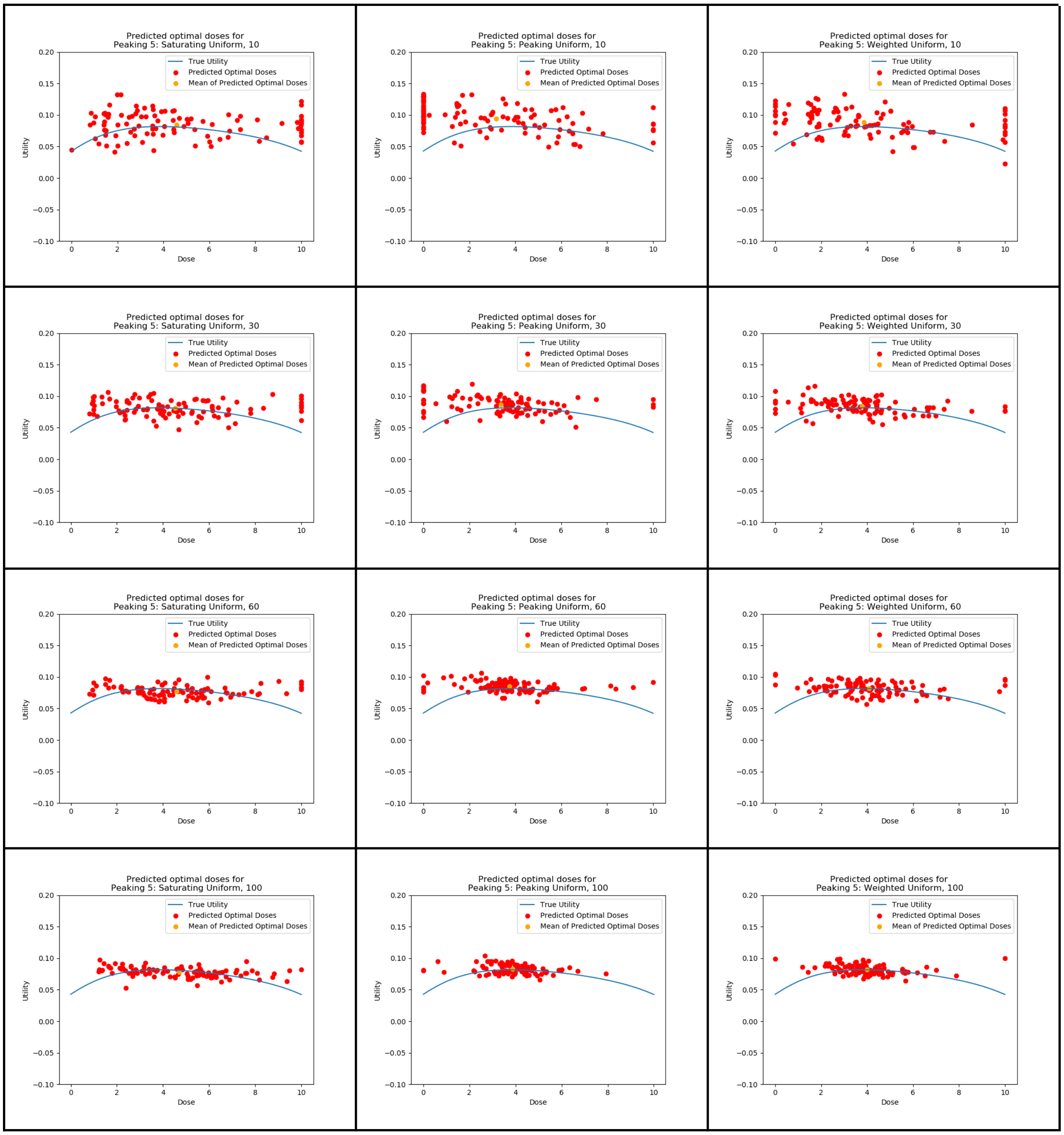

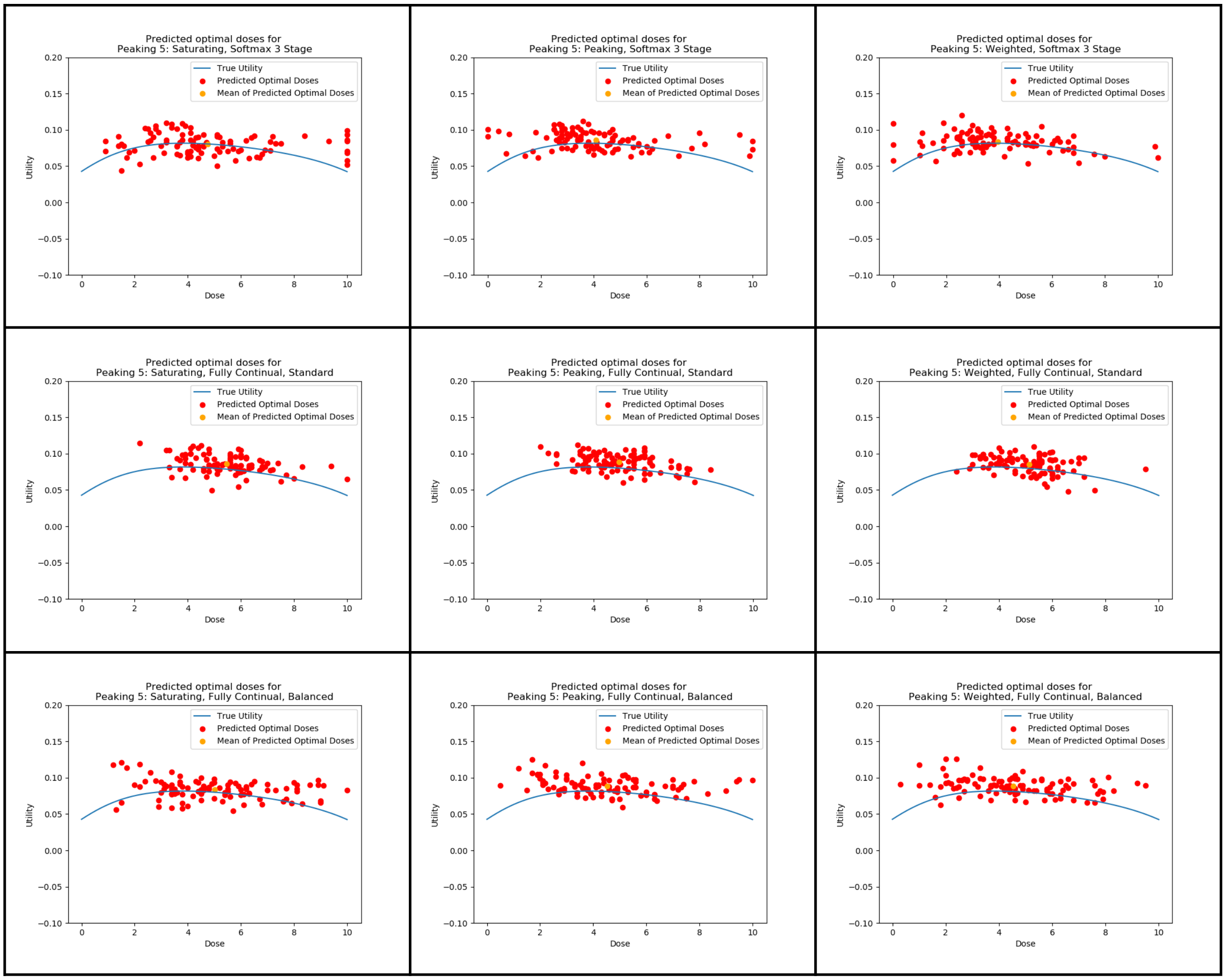

Fig.S.ClinTrialPlots.P5. Plots of true utility curves and predicted optimal/dose response for 100 simulations of each approach for this scenario. Approaches use a saturating (left), peaking(middle), or weighted (right) efficacy curve. From top to bottom; trial size is 10, 30, 60, 100, 30, 30, 30. Method of trial dose selection from top to bottom in uniform, uniform, uniform, uniform, softmax 3 stage. Standard fully continual, and balanced fully continual.

Scenario Other 1

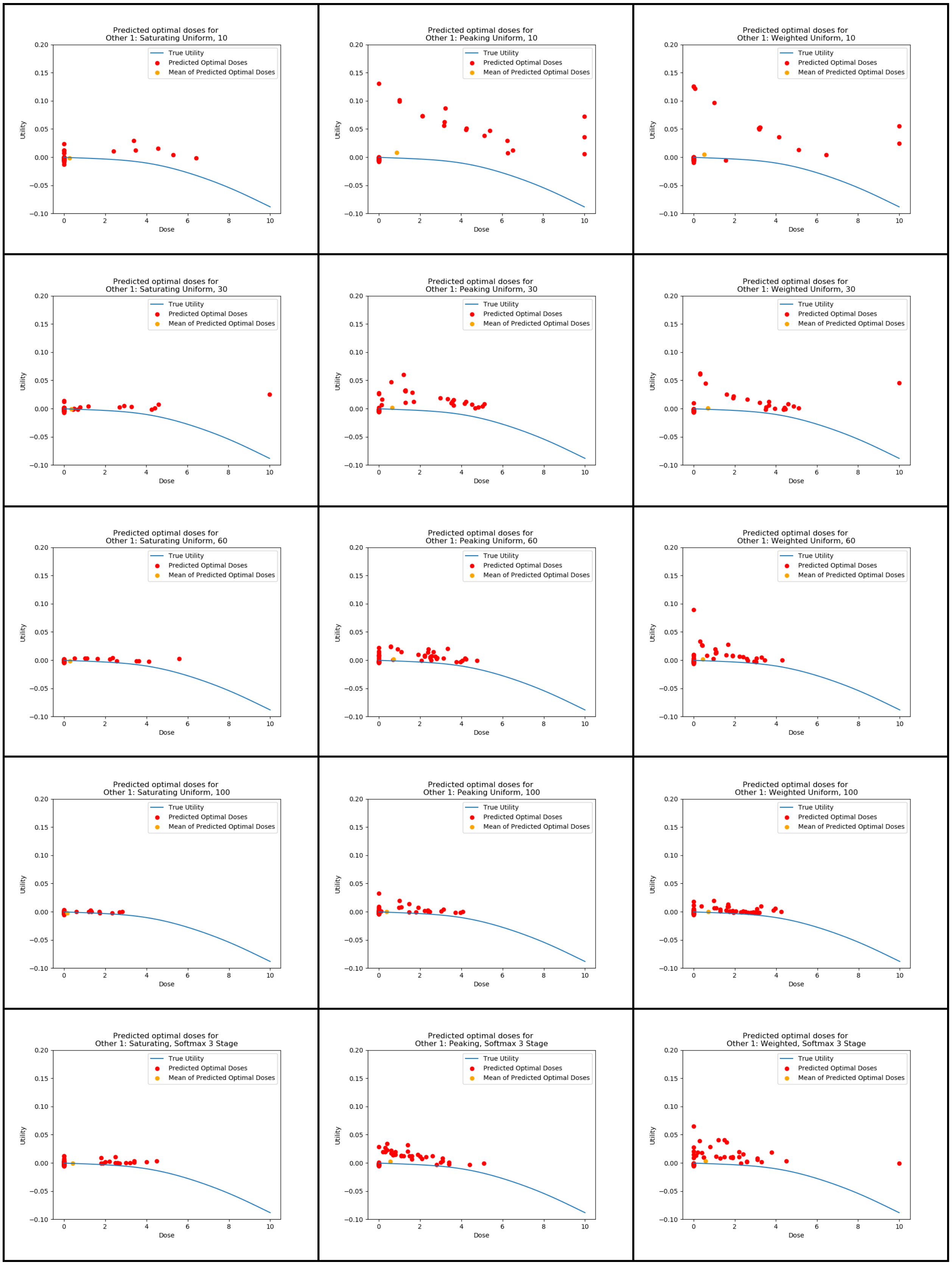
Predicted optimal doses for
Other 1: Saturating Uniform, 10
Predicted optimal doses for
Other 1: Peaking Uniform, 10
Predicted optimal doses for
Other 1: Weighted Uniform, 10
Predicted optimal doses for
Other 1: Saturating Uniform, 30
Predicted optimal doses for
Other 1: Peaking Uniform, 30
Predicted optimal doses for
Other 1: Weighted Uniform, 30
Predicted optimal doses for
Other 1: Saturating Uniform, 60
Predicted optimal doses for
Other 1: Peaking Uniform, 60
Predicted optimal doses for
Other 1: Weighted Uniform, 60
Predicted optimal doses for
Other 1: Saturating Uniform, 100
Predicted optimal doses for
Other 1: Peaking Uniform, 100
Predicted optimal doses for
Other 1: Weighted Uniform, 100
Predicted optimal doses for
Other 1: Saturating, Softmax 3 Stage
Predicted optimal doses for
Other 1: Peaking, Softmax 3 Stage
Predicted optimal doses for
Other 1: Weighted, Softmax 3 Stage
True Utility
Predicted Optimal Doses
Mean of Predicted Optimal Doses
Utility
Dose
0.20
0.15
0.10
0.05
0.00
−0.05
−0.10
0
2
4
6
8
10

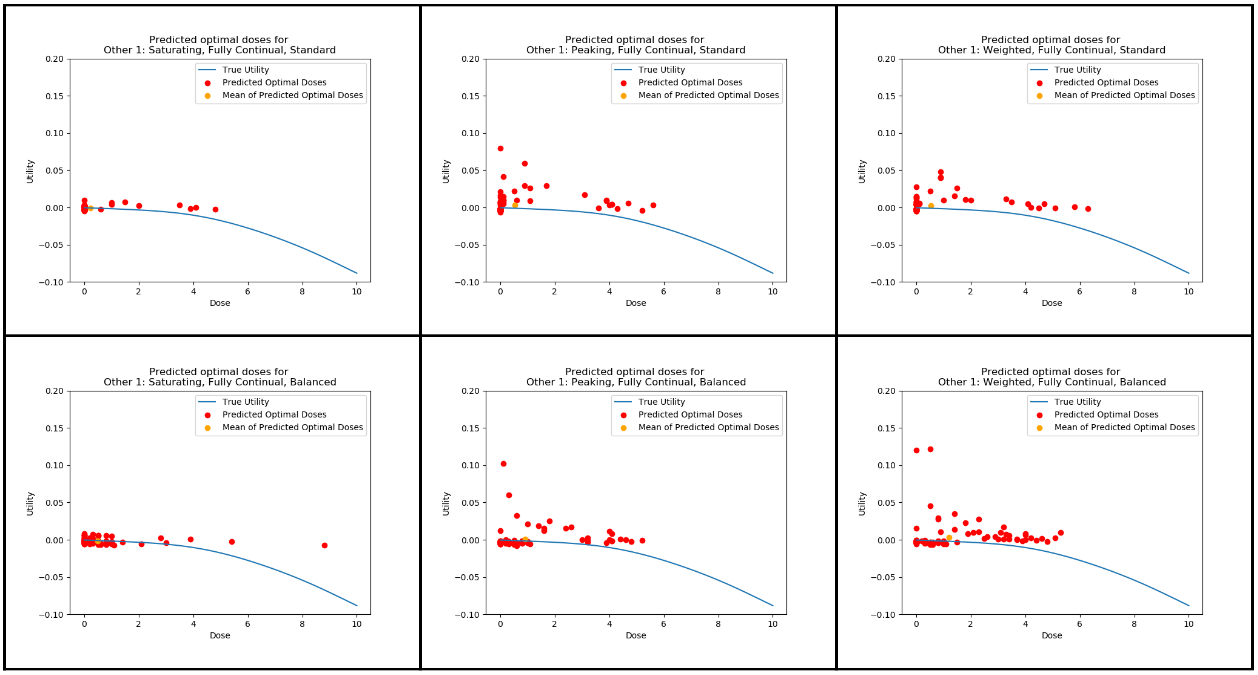

Fig.S.ClinTrialPlots.X1. Plots of true utility curves and predicted optimal/dose response for 100 simulations of each approach for this scenario. Approaches use a saturating (left), peaking(middle), or weighted (right) efficacy curve. From top to bottom; trial size is 10, 30, 60, 100, 30, 30, 30. Method of trial dose selection from top to bottom in uniform, uniform, uniform, uniform, softmax 3 stage. Standard fully continual, and balanced fully continual.

Scenario Other 2

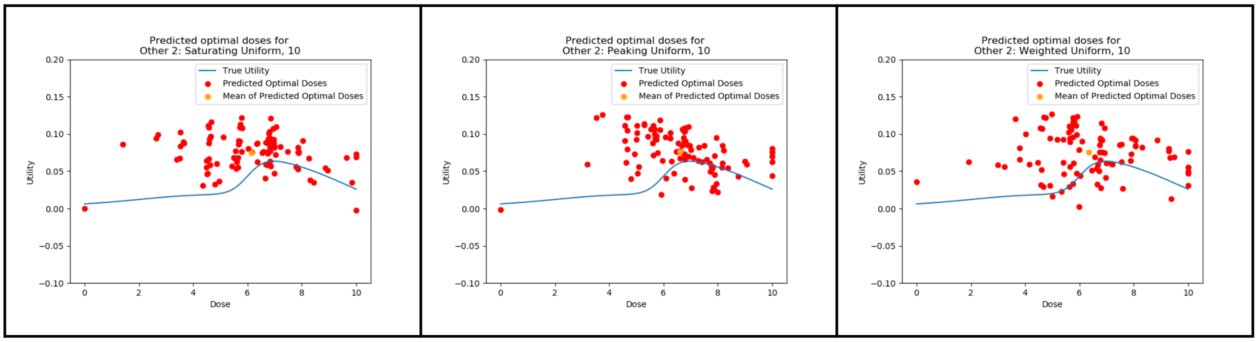

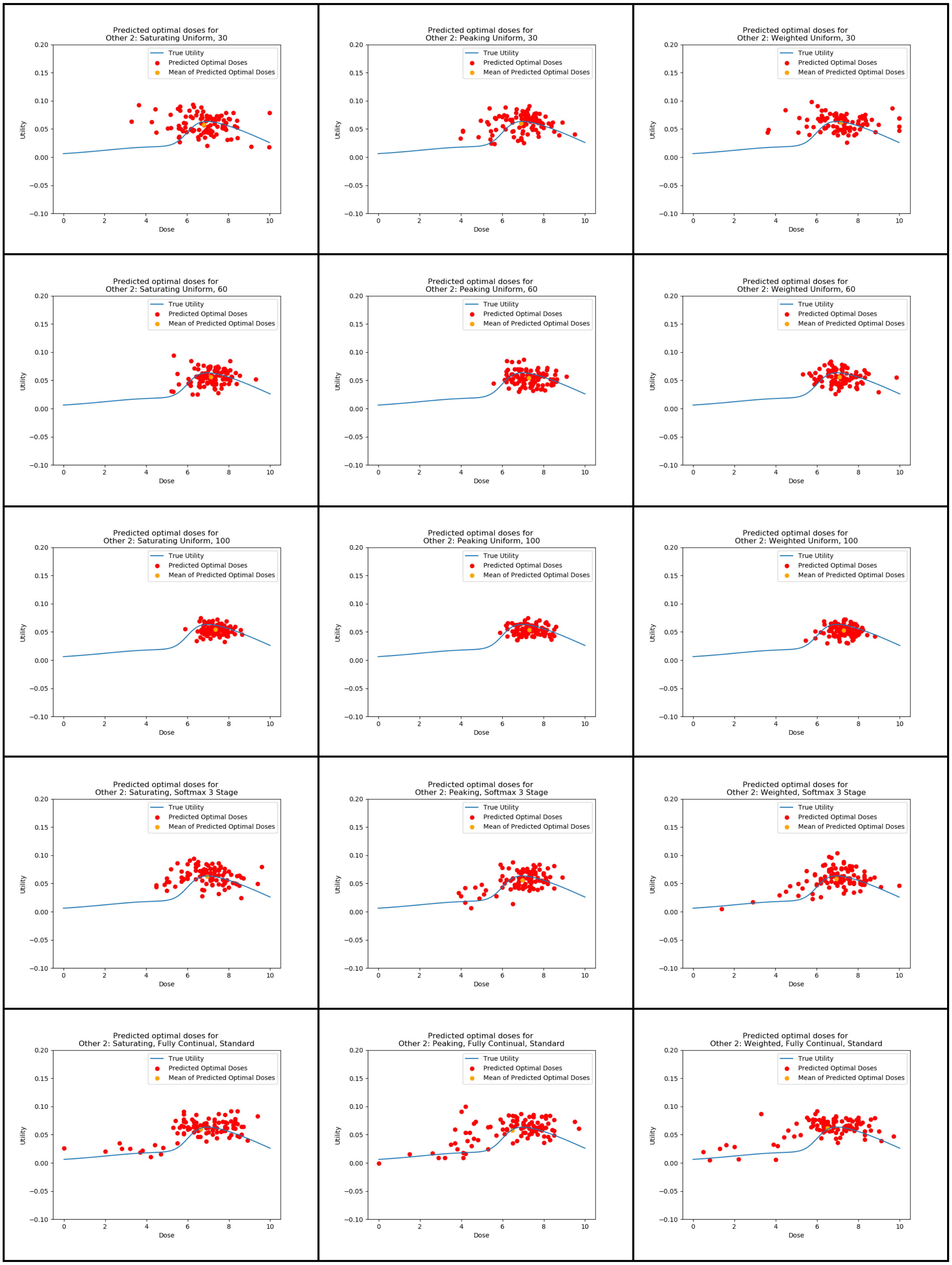
Predicted optimal doses for Other 2: Saturating Uniform, 30
Predicted optimal doses for Other 2: Peaking Uniform, 30
Predicted optimal doses for Other 2: Weighted Uniform, 30
Predicted optimal doses for Other 2: Saturating Uniform, 60
Predicted optimal doses for Other 2: Peaking Uniform, 60
Predicted optimal doses for Other 2: Weighted Uniform, 60
Predicted optimal doses for Other 2: Saturating Uniform, 100
Predicted optimal doses for Other 2: Peaking Uniform, 100
Predicted optimal doses for Other 2: Weighted Uniform, 100
Predicted optimal doses for Other 2: Saturating, Softmax 3 Stage
Predicted optimal doses for Other 2: Peaking, Softmax 3 Stage
Predicted optimal doses for Other 2: Weighted, Softmax 3 Stage
Predicted optimal doses for Other 2: Saturating, Fully Continual, Standard
Predicted optimal doses for Other 2: Peaking, Fully Continual, Standard
Predicted optimal doses for Other 2: Weighted, Fully Continual, Standard
True Utility
Predicted Optimal Doses
Mean of Predicted Optimal Doses
Utility
Dose

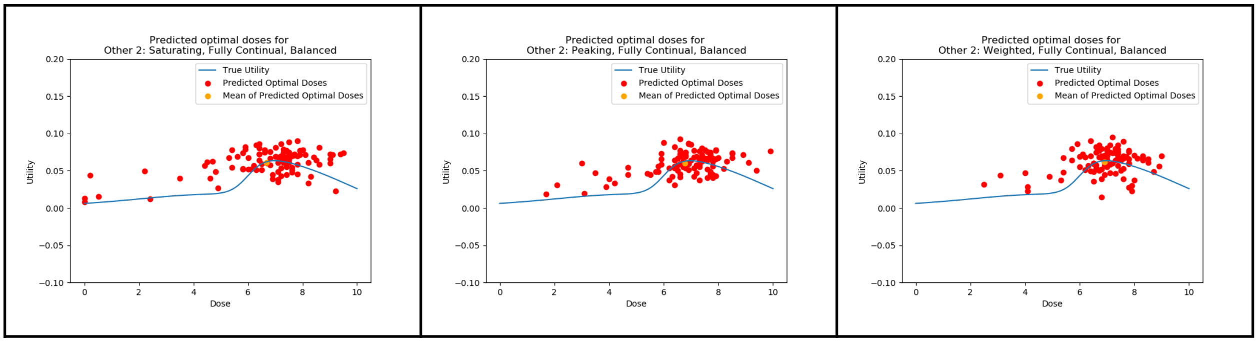

Fig.S.ClinTrialPlots.X2. Plots of true utility curves and predicted optimal/dose response for 100 simulations of each approach for this scenario. Approaches use a saturating (left), peaking(middle), or weighted (right) efficacy curve. From top to bottom; trial size is 10, 30, 60, 100, 30, 30, 30. Method of trial dose selection from top to bottom in uniform, uniform, uniform, uniform, softmax 3 stage. Standard fully continual, and balanced fully continual.

Scenario Other 3

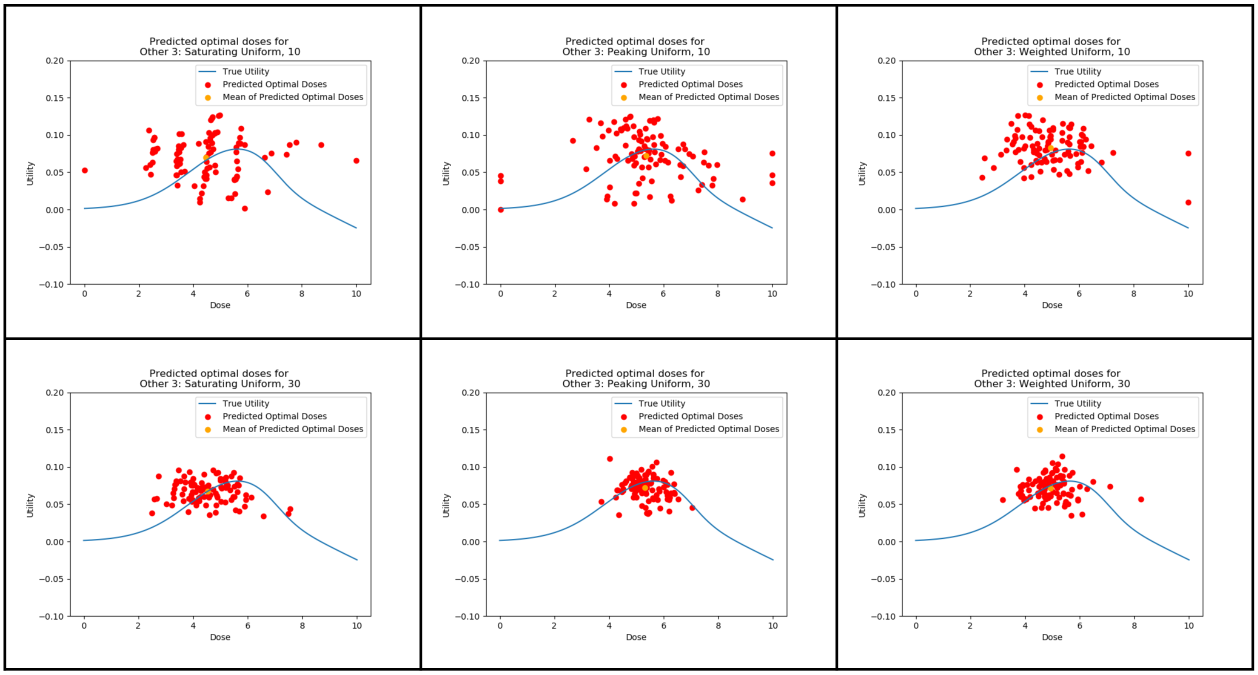

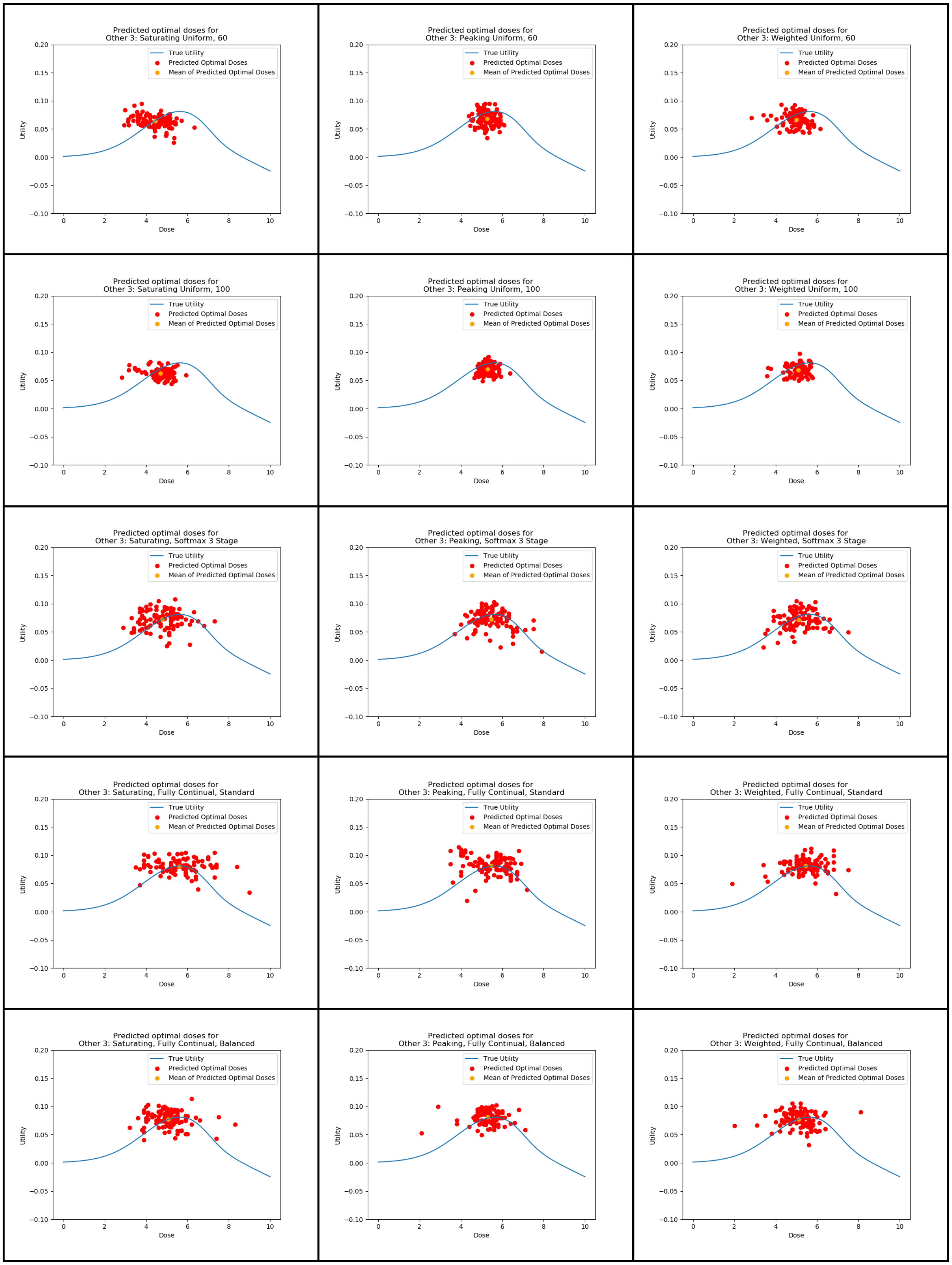
Predicted optimal doses for
Other 3: Saturating Uniform, 60
Predicted optimal doses for
Other 3: Peaking Uniform, 60
Predicted optimal doses for
Other 3: Weighted Uniform, 60
Predicted optimal doses for
Other 3: Saturating Uniform, 100
Predicted optimal doses for
Other 3: Peaking Uniform, 100
Predicted optimal doses for
Other 3: Weighted Uniform, 100
Predicted optimal doses for
Other 3: Saturating, Softmax 3 Stage
Predicted optimal doses for
Other 3: Peaking, Softmax 3 Stage
Predicted optimal doses for
Other 3: Weighted, Softmax 3 Stage
Predicted optimal doses for
Other 3: Saturating, Fully Continual, Standard
Predicted optimal doses for
Other 3: Peaking, Fully Continual, Standard
Predicted optimal doses for
Other 3: Weighted, Fully Continual, Standard
Predicted optimal doses for
Other 3: Saturating, Fully Continual, Balanced
Predicted optimal doses for
Other 3: Peaking, Fully Continual, Balanced
Predicted optimal doses for
Other 3: Weighted, Fully Continual, Balanced
True Utility
Predicted Optimal Doses
Mean of Predicted Optimal Doses
Utility
Dose
0.20
0.15
0.10
0.05
0.00
−0.05
−0.10
0
2
4
6
8
10

Fig.S.ClinTrialPlots.X3. Plots of true utility curves and predicted optimal/dose response for 100 simulations of each approach for this scenario. Approaches use a saturating (left), peaking(middle), or weighted (right) efficacy curve. From top to bottom; trial size is 10, 30, 60, 100, 30, 30, 30. Method of trial dose selection from top to bottom in uniform, uniform, uniform, uniform, softmax 3 stage. Standard fully continual, and balanced fully continual.

Scenario Other 4

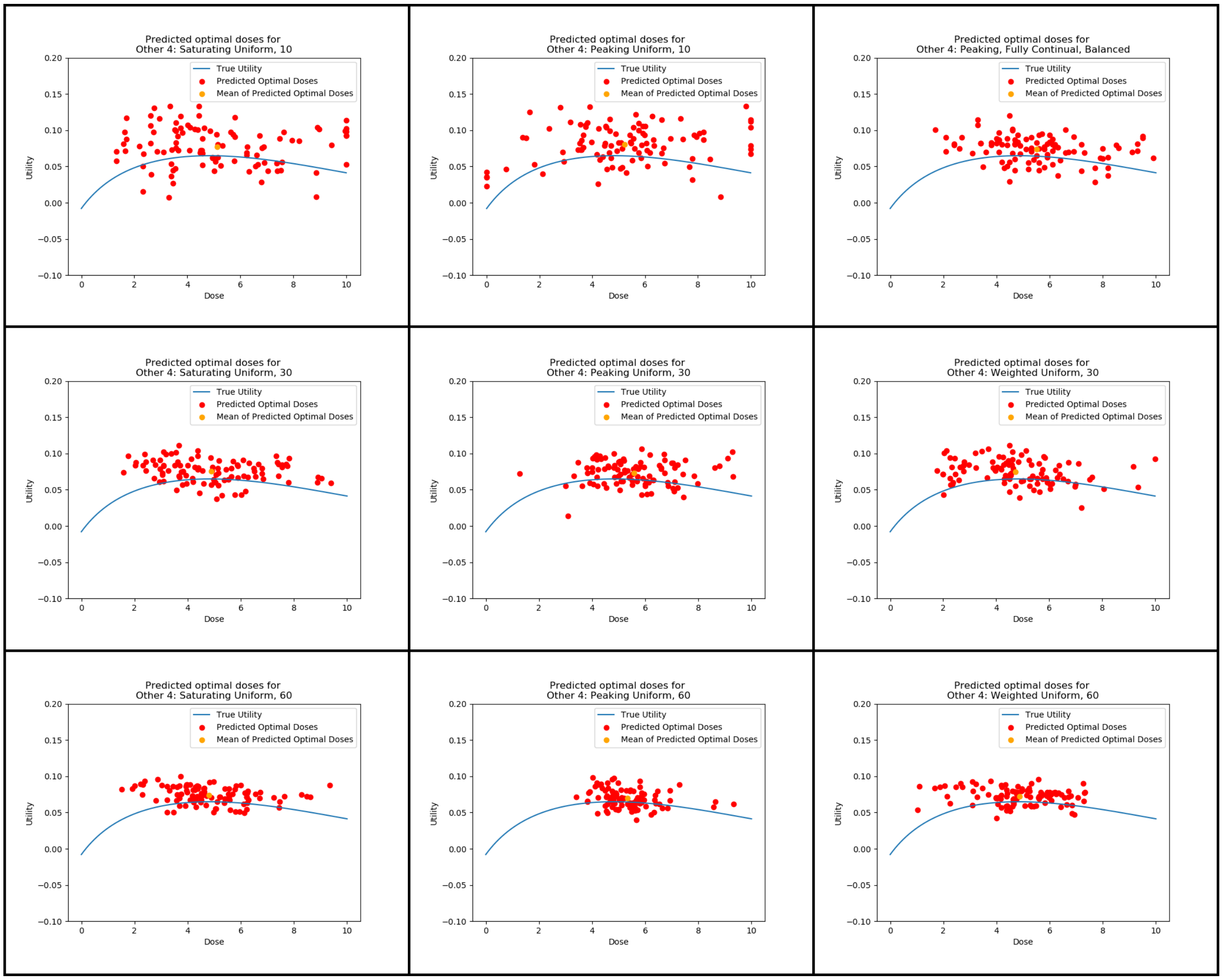

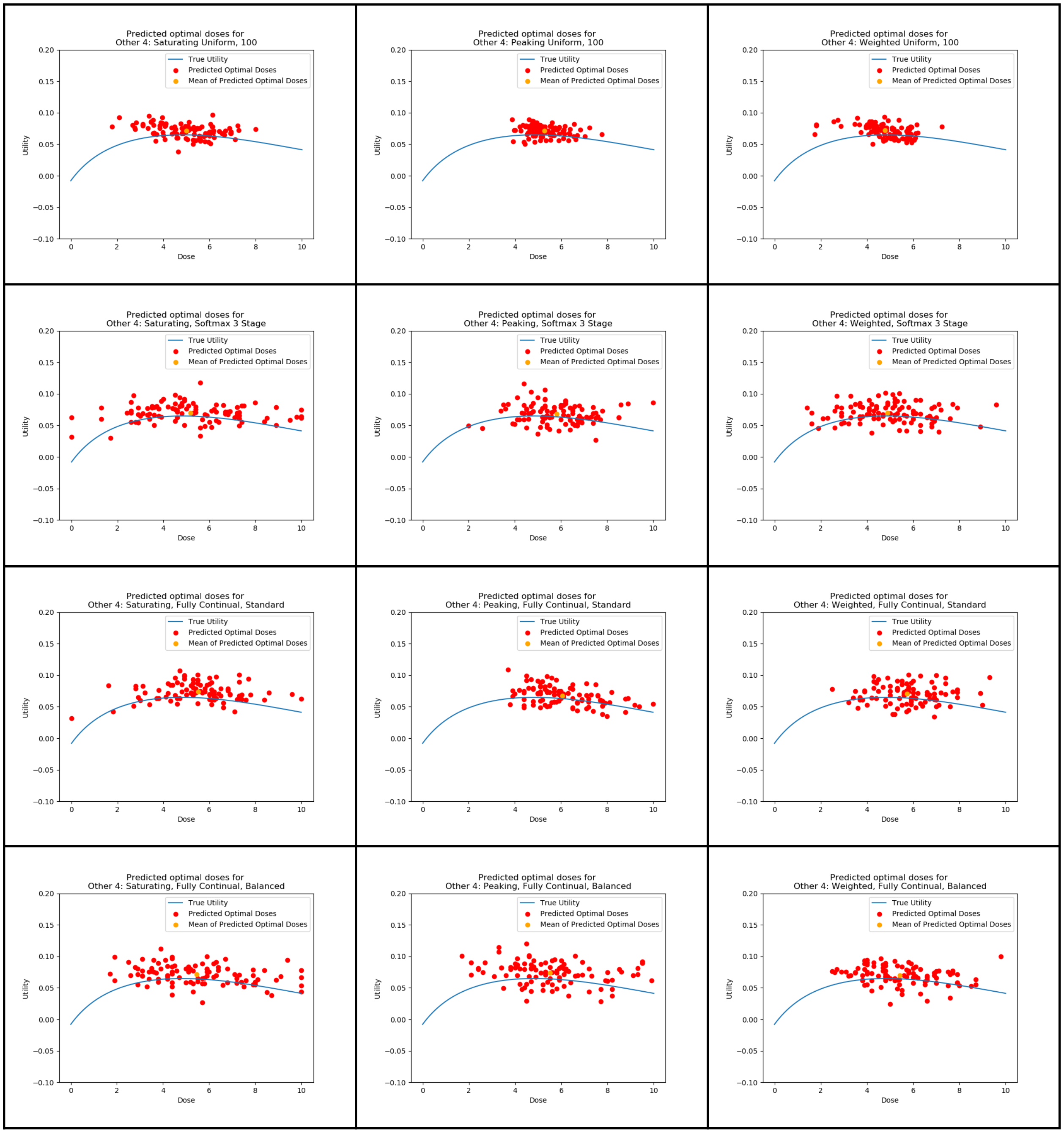

Fig.S.ClinTrialPlots.X4. Plots of true utility curves and predicted optimal/dose response for 100 simulations of each approach for this scenario. Approaches use a saturating (left), peaking(middle), or weighted (right) efficacy curve. From top to bottom; trial size is 10, 30, 60, 100, 30, 30, 30. Method of trial dose selection from top to bottom in uniform, uniform, uniform, uniform, softmax 3 stage. Standard fully continual, and balanced fully continual.

Supplementary 11. Objective 1 Plots

Scenario Saturating 1

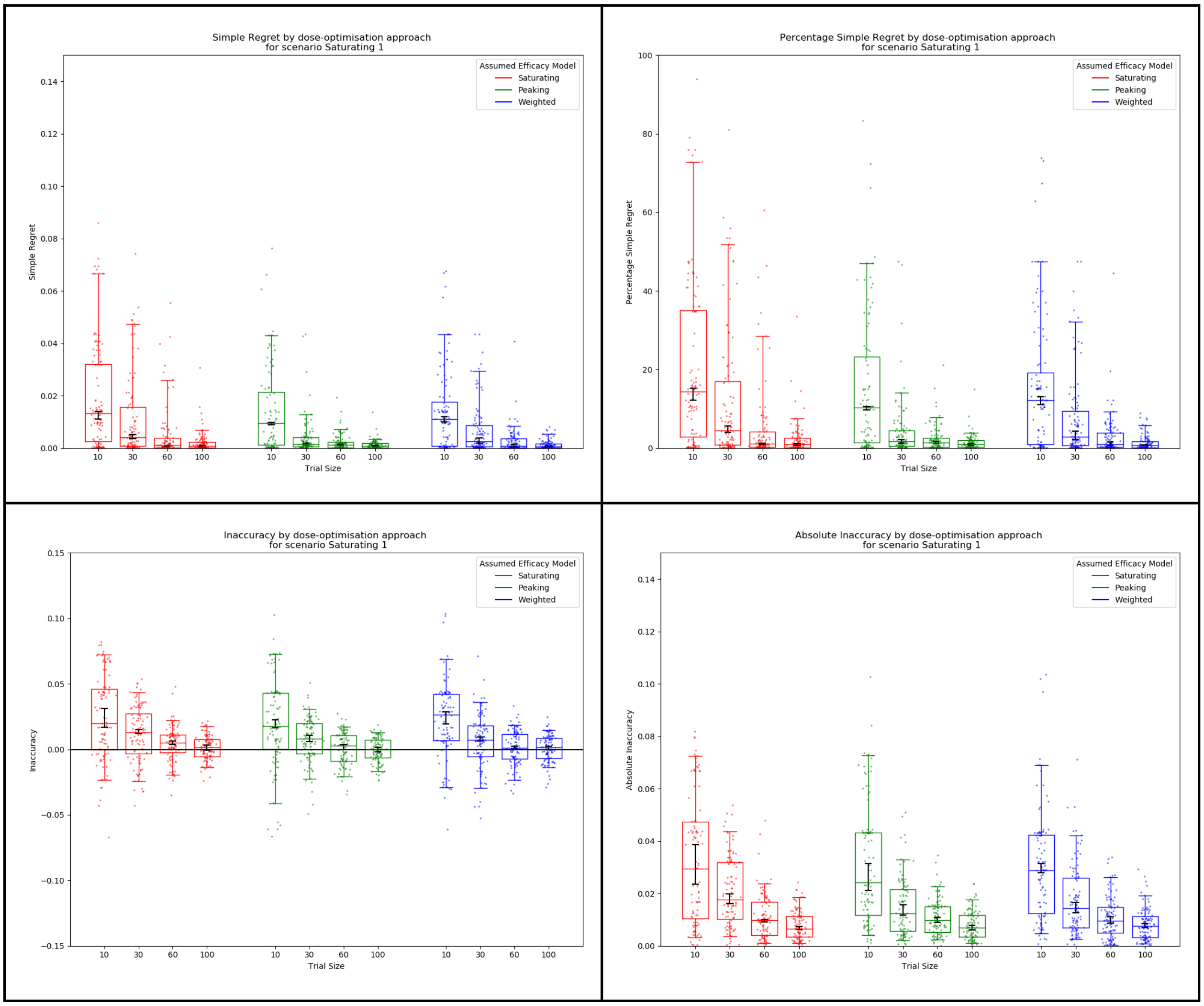

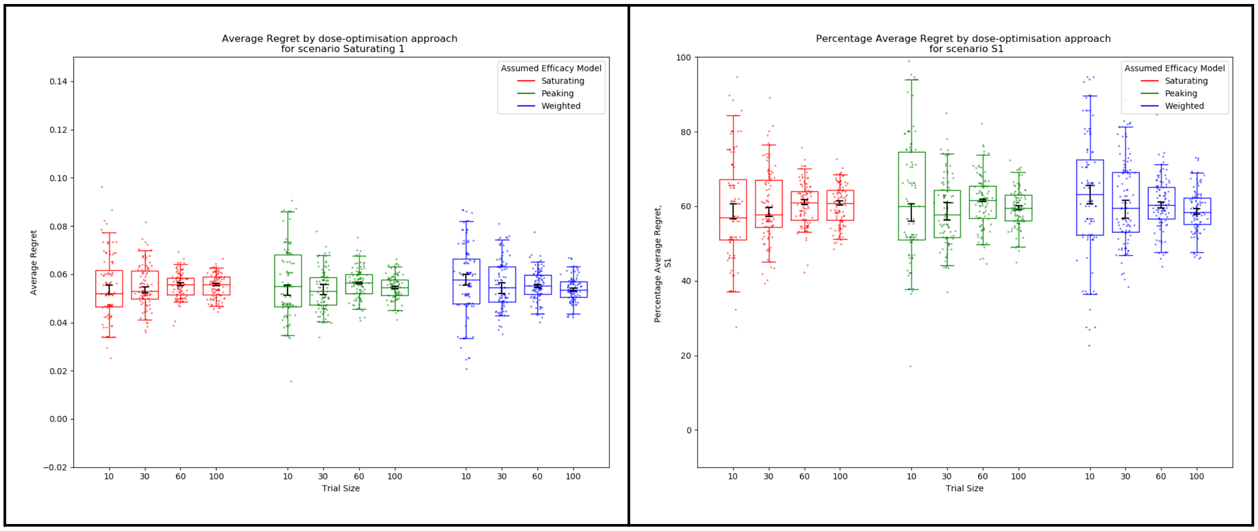

Figure Supplementary.Obj1_Plots.Saturating 1. Plots of the metrics from simulations for dose-optimisation approaches in objective 1 for scenario Saturating 1. The shown metrics are simple regret (top left), percentage simple regret (top right), inaccuracy (middle left), absolute inaccuracy (middle right), average regret (bottom left), and percentage average regret (bottom right).

Scenario Saturating 2

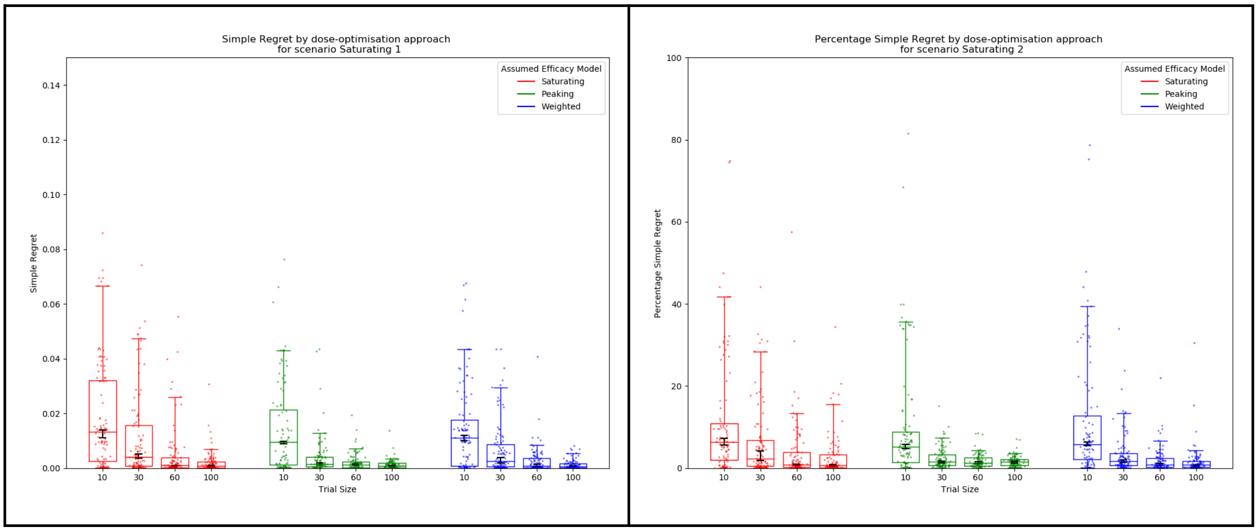

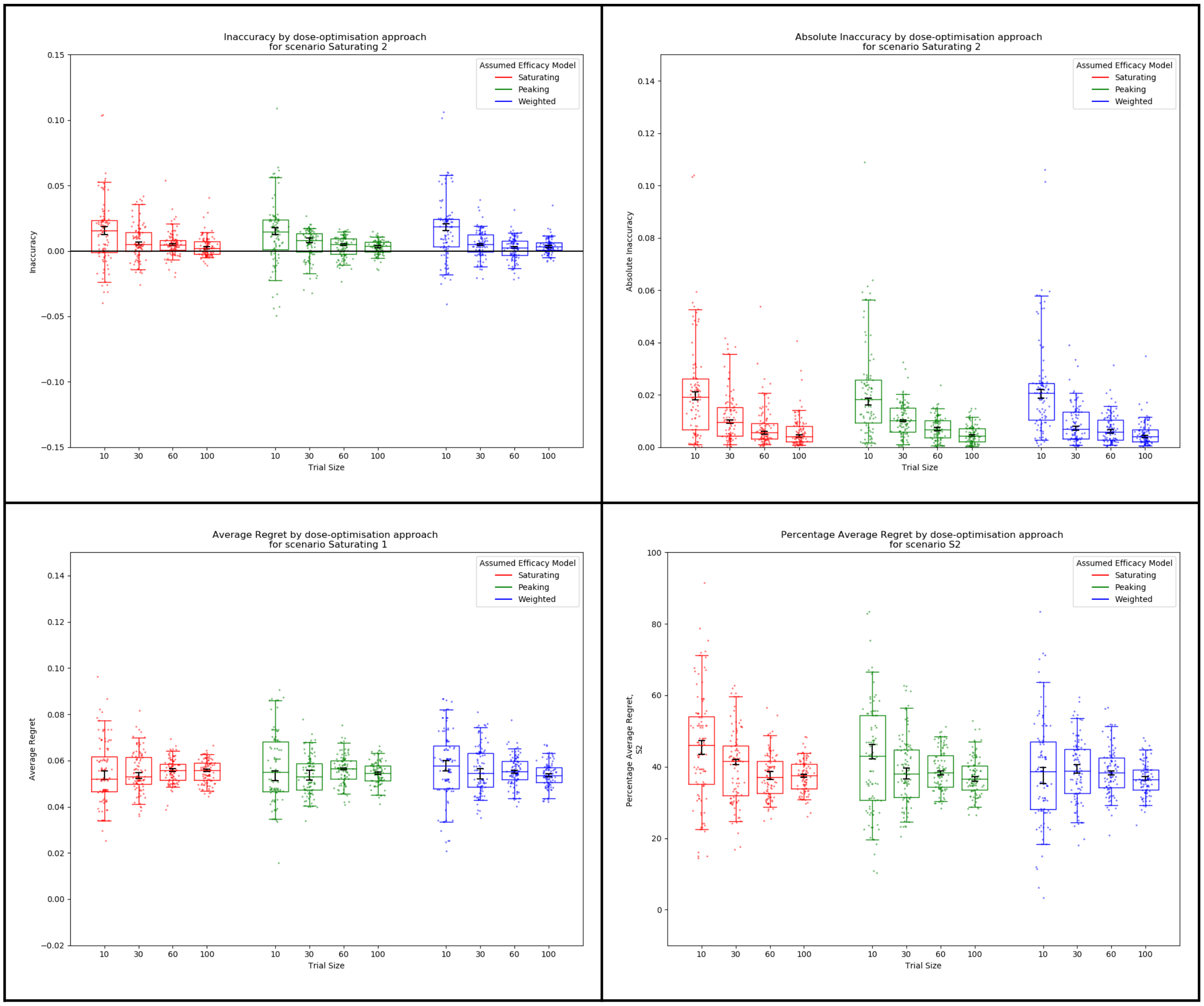

Figure Supplementary.Obj1_Plots.Saturating 2. Plots of the metrics from simulations for dose-optimisation approaches in objective 1 for scenario Saturating 2. The shown metrics are simple regret (top left), percentage simple regret (top right), inaccuracy (middle left), absolute inaccuracy (middle right), average regret (bottom left), and percentage average regret (bottom right).

Scenario Saturating 3

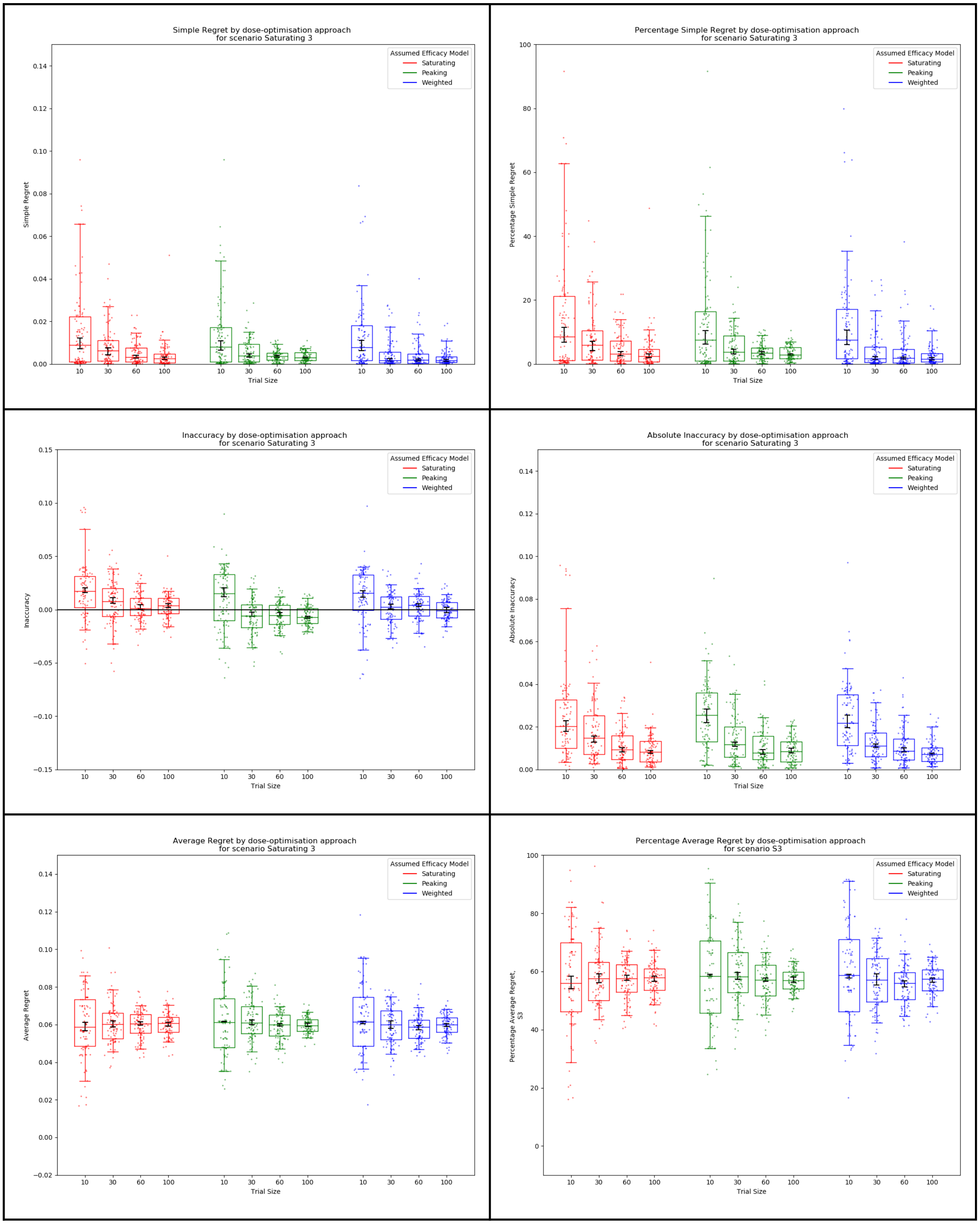

Figure Supplementary.Obj1_Plots.Saturating 3. Plots of the metrics from simulations for dose-optimisation approaches in objective 1 for scenario Saturating 3. The shown metrics are simple regret (top left), percentage simple regret (top right), inaccuracy (middle left), absolute

inaccuracy (middle right), average regret (bottom left), and percentage average regret (bottom right).

Scenario Saturating 4

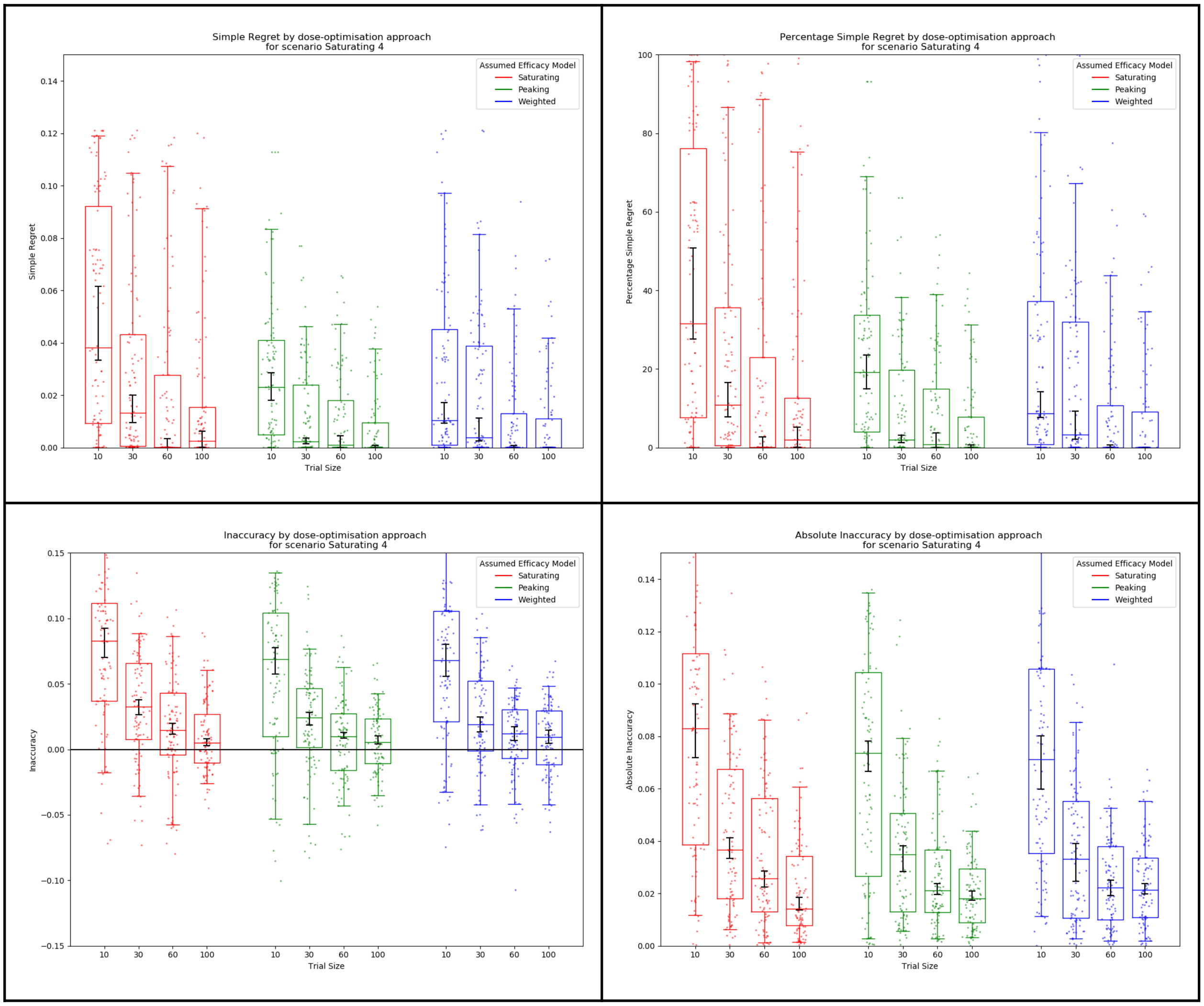

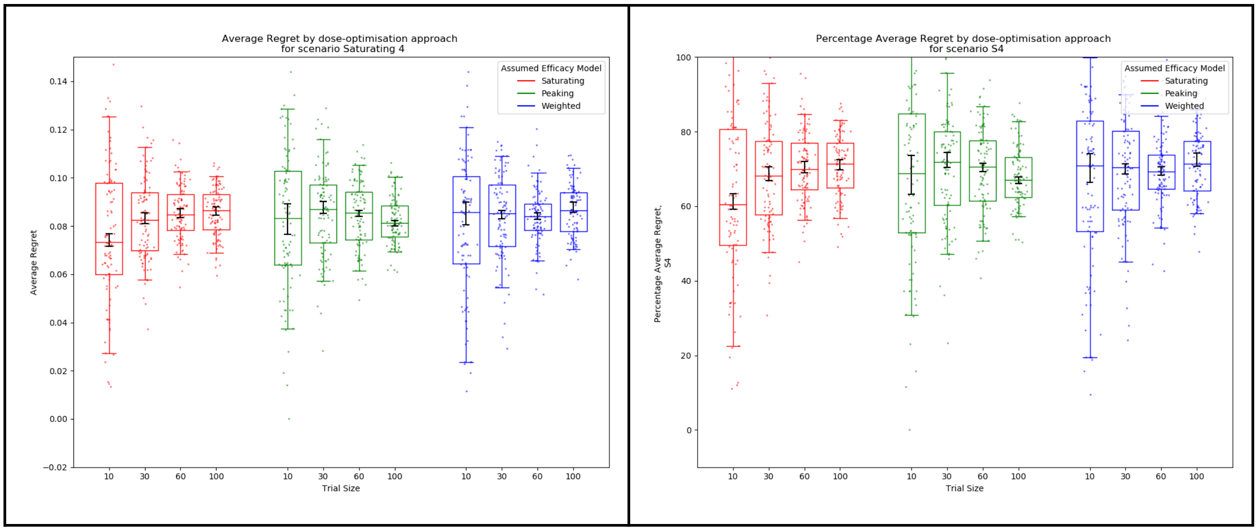

Figure Supplementary.Obj1_Plots.Saturating 4. Plots of the metrics from simulations for dose-optimisation approaches in objective 1 for scenario Saturating 4. The shown metrics are simple regret (top left), percentage simple regret (top right), inaccuracy (middle left), absolute inaccuracy (middle right), average regret (bottom left), and percentage average regret (bottom right).

Scenario Saturating 5

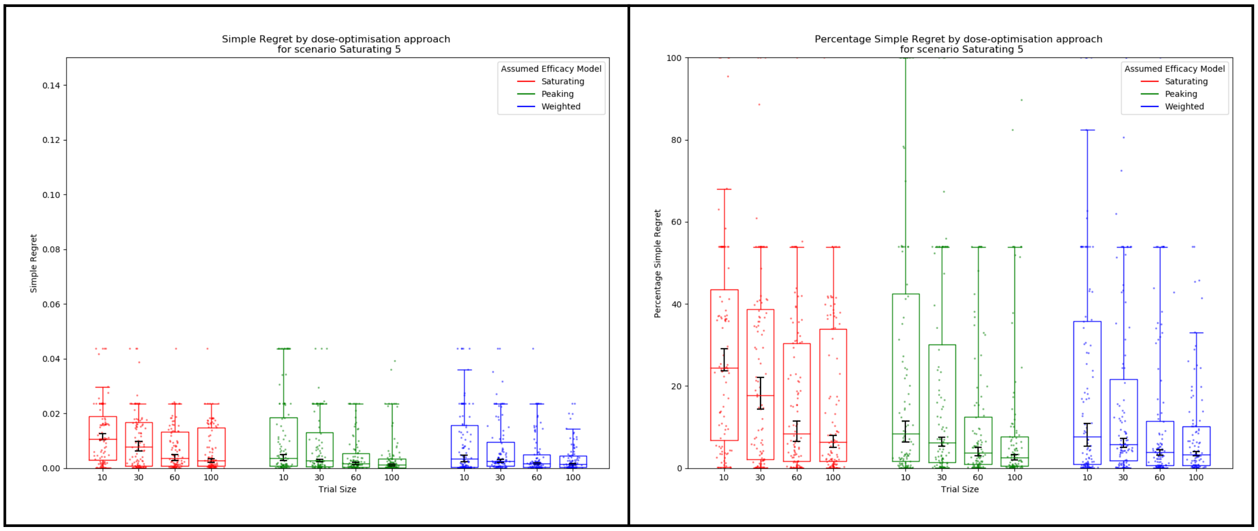

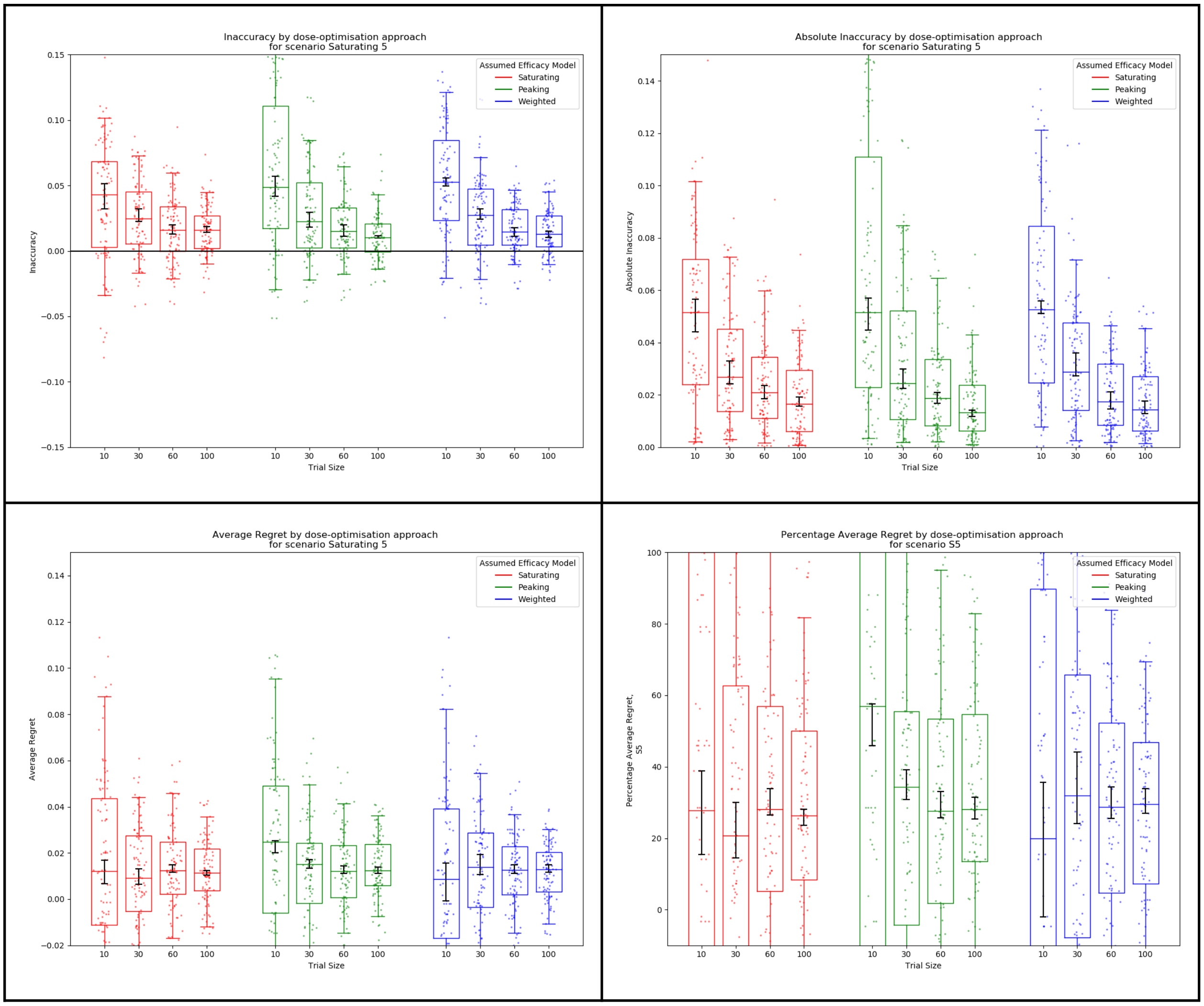

Figure Supplementary.Obj1_Plots.Saturating 5. Plots of the metrics from simulations for dose-optimisation approaches in objective 1 for scenario Saturating 5. The shown metrics are simple regret (top left), percentage simple regret (top right), inaccuracy (middle left), absolute inaccuracy (middle right), average regret (bottom left), and percentage average regret (bottom right).

Scenario Peaking 1

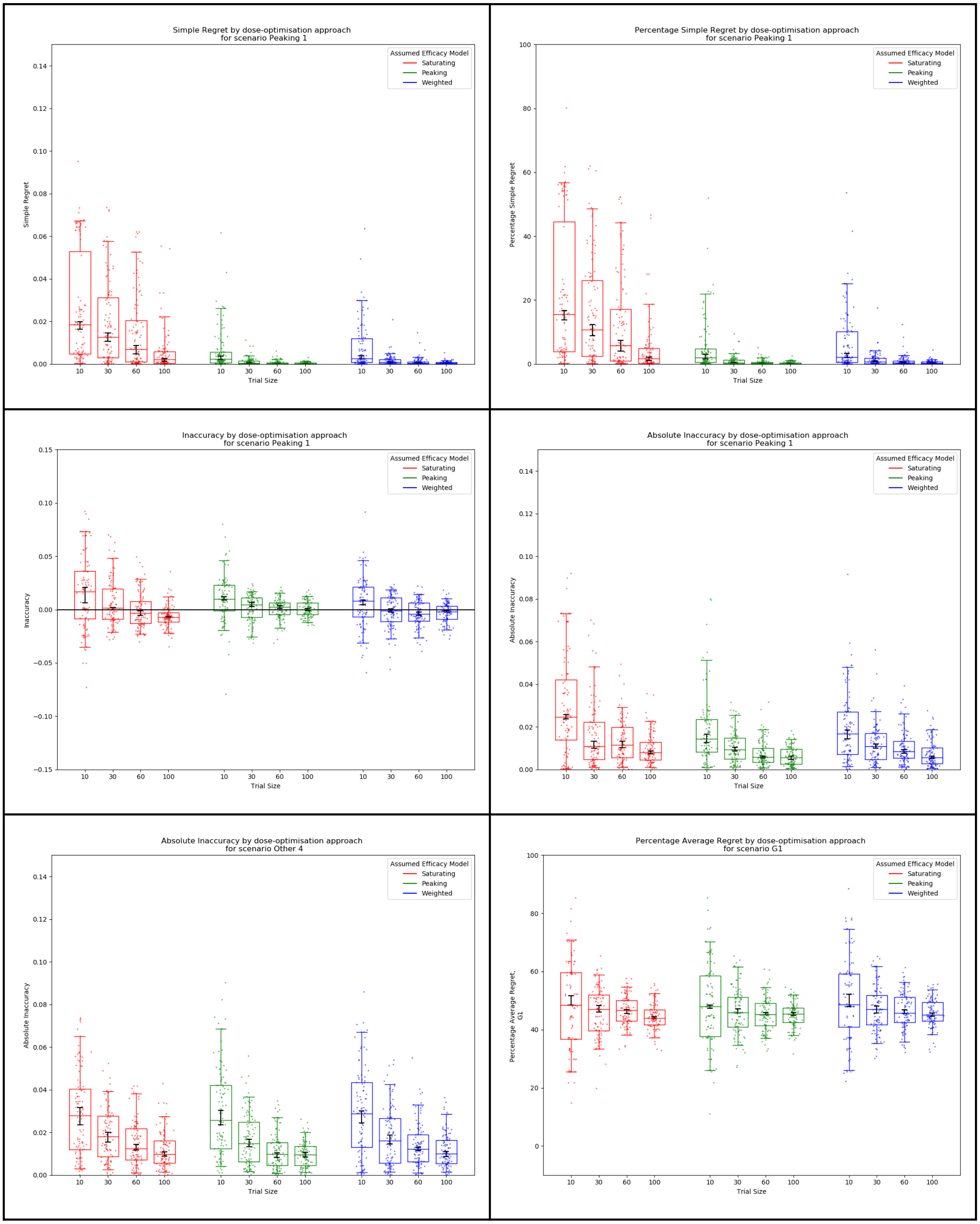

Figure Supplementary.Obj1_Plots.Peaking 1. Plots of the metrics from simulations for dose-optimisation approaches in objective 1 for scenario Peaking 1. The shown metrics are simple regret (top left), percentage simple regret (top right), inaccuracy (middle left), absolute

inaccuracy (middle right), average regret (bottom left), and percentage average regret (bottom right).

Scenario Peaking 2

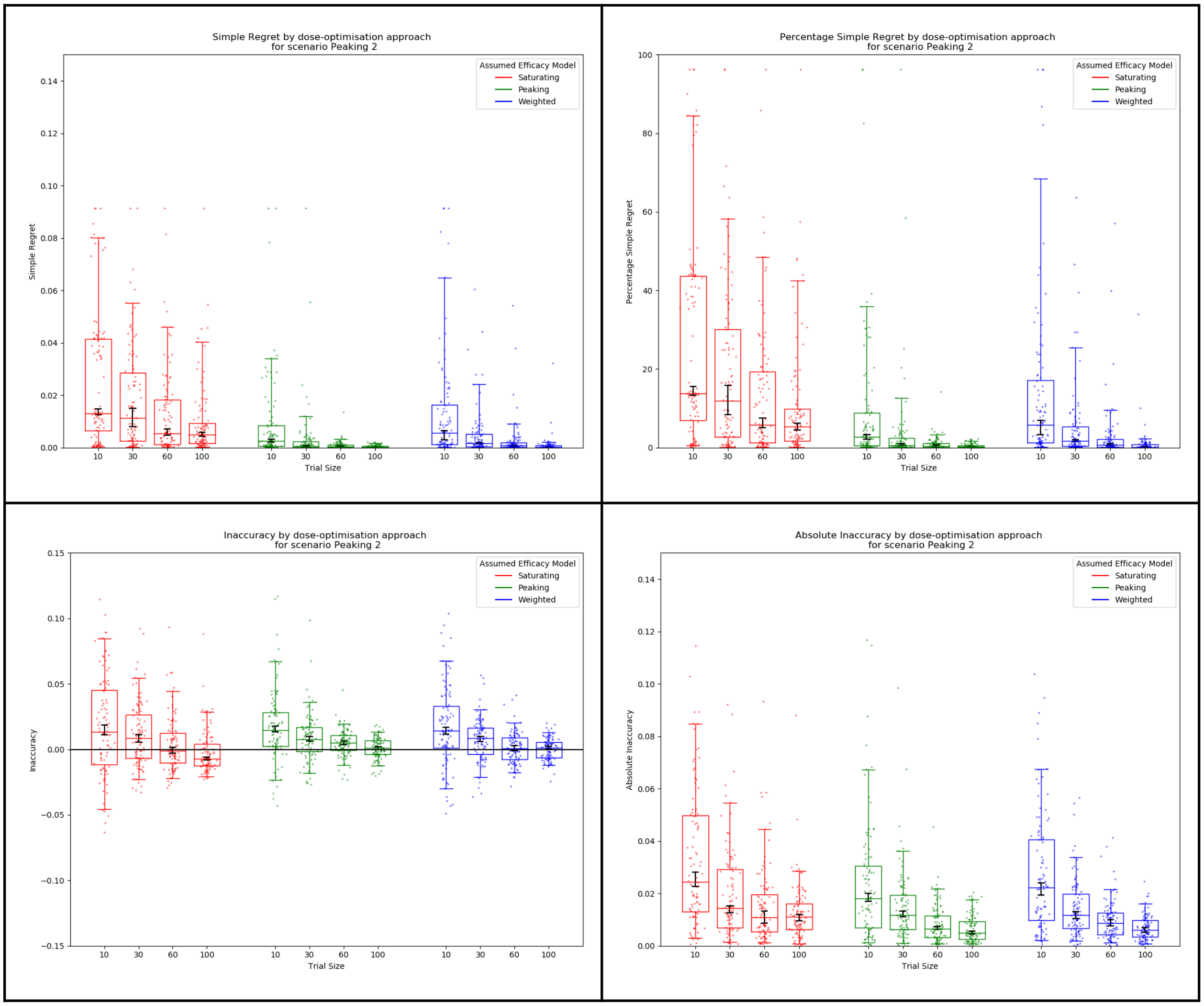

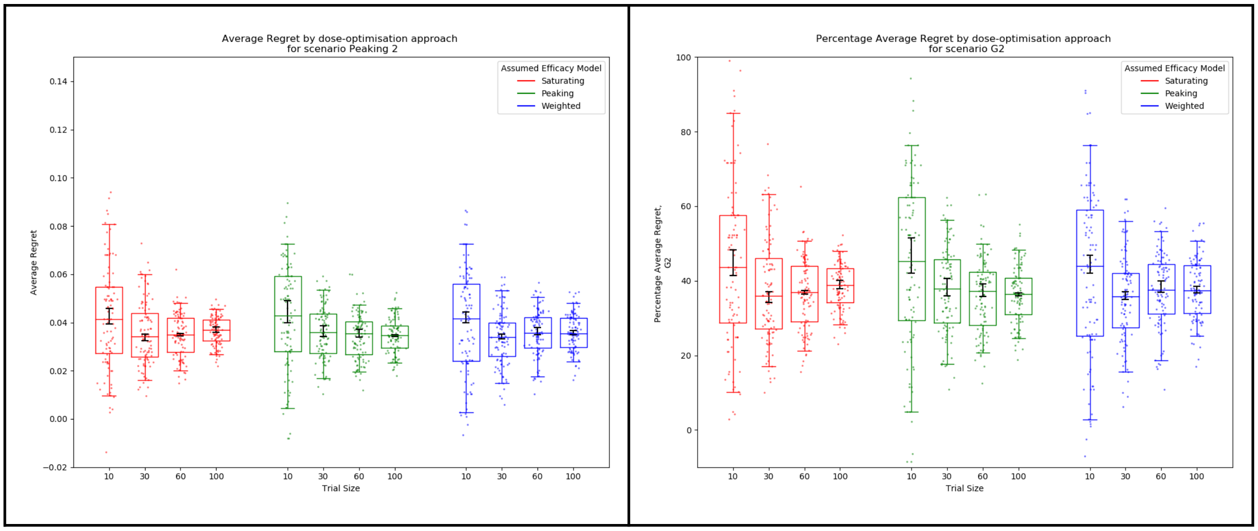

Figure Supplementary.Obj1_Plots.Peaking 2. Plots of the metrics from simulations for dose-optimisation approaches in objective 1 for scenario Peaking 2. The shown metrics are simple regret (top left), percentage simple regret (top right), inaccuracy (middle left), absolute inaccuracy (middle right), average regret (bottom left), and percentage average regret (bottom right).

Scenario Peaking 3

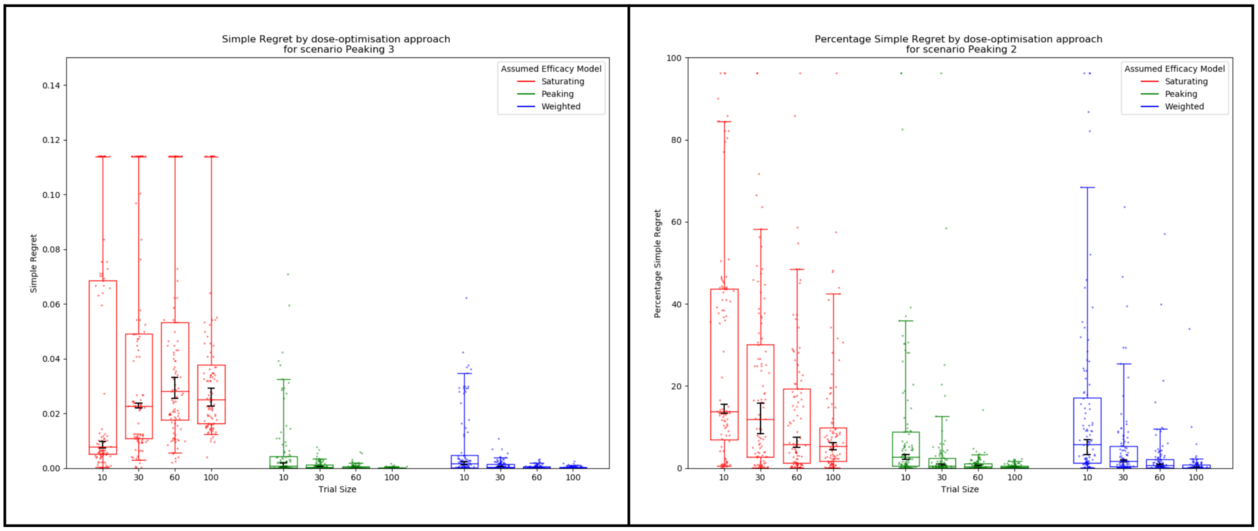

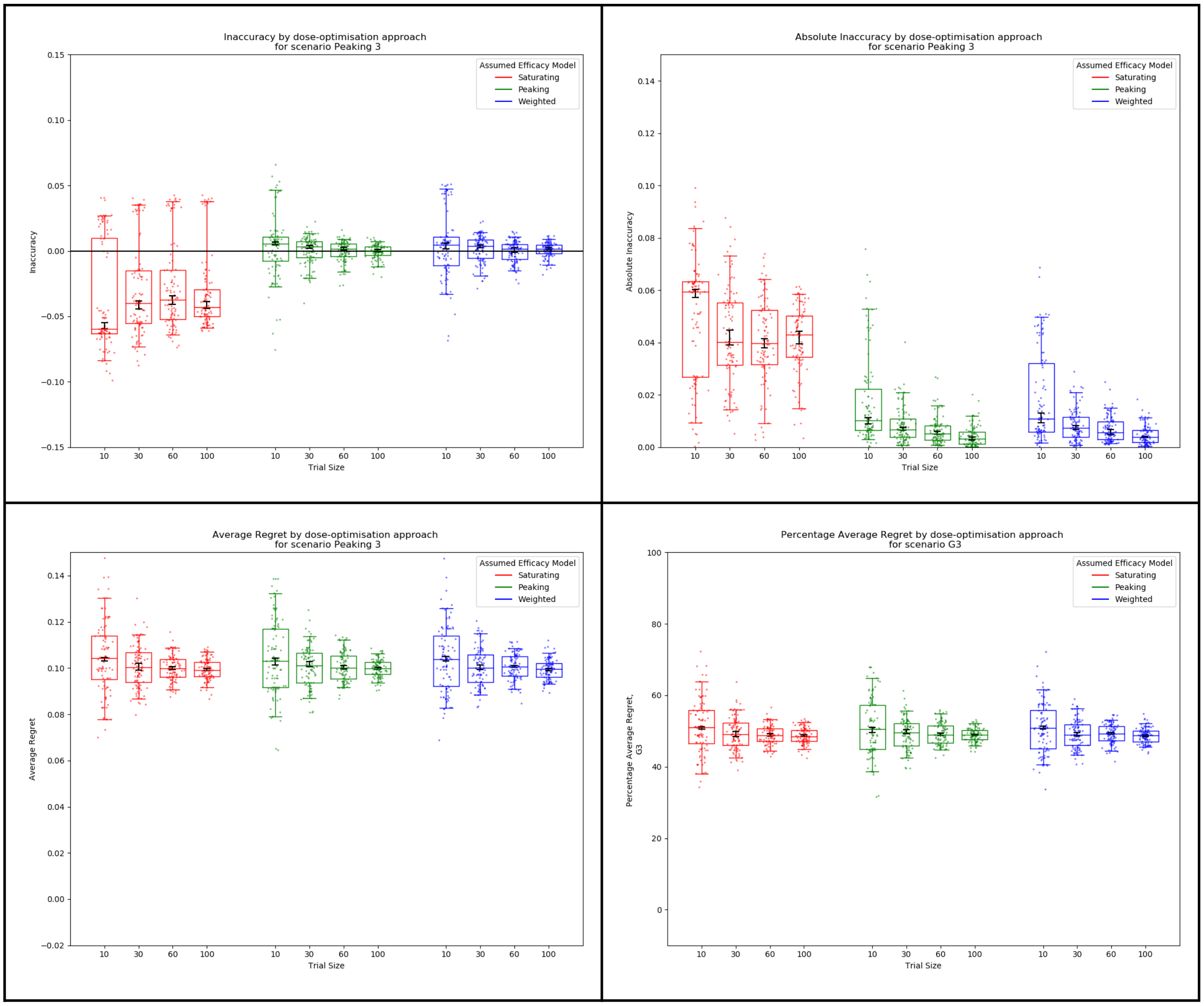

Figure Supplementary.Obj1_Plots.Peaking 3. Plots of the metrics from simulations for dose-optimisation approaches in objective 1 for scenario Peaking 3. The shown metrics are simple regret (top left), percentage simple regret (top right), inaccuracy (middle left), absolute inaccuracy (middle right), average regret (bottom left), and percentage average regret (bottom right).

Scenario Peaking 4

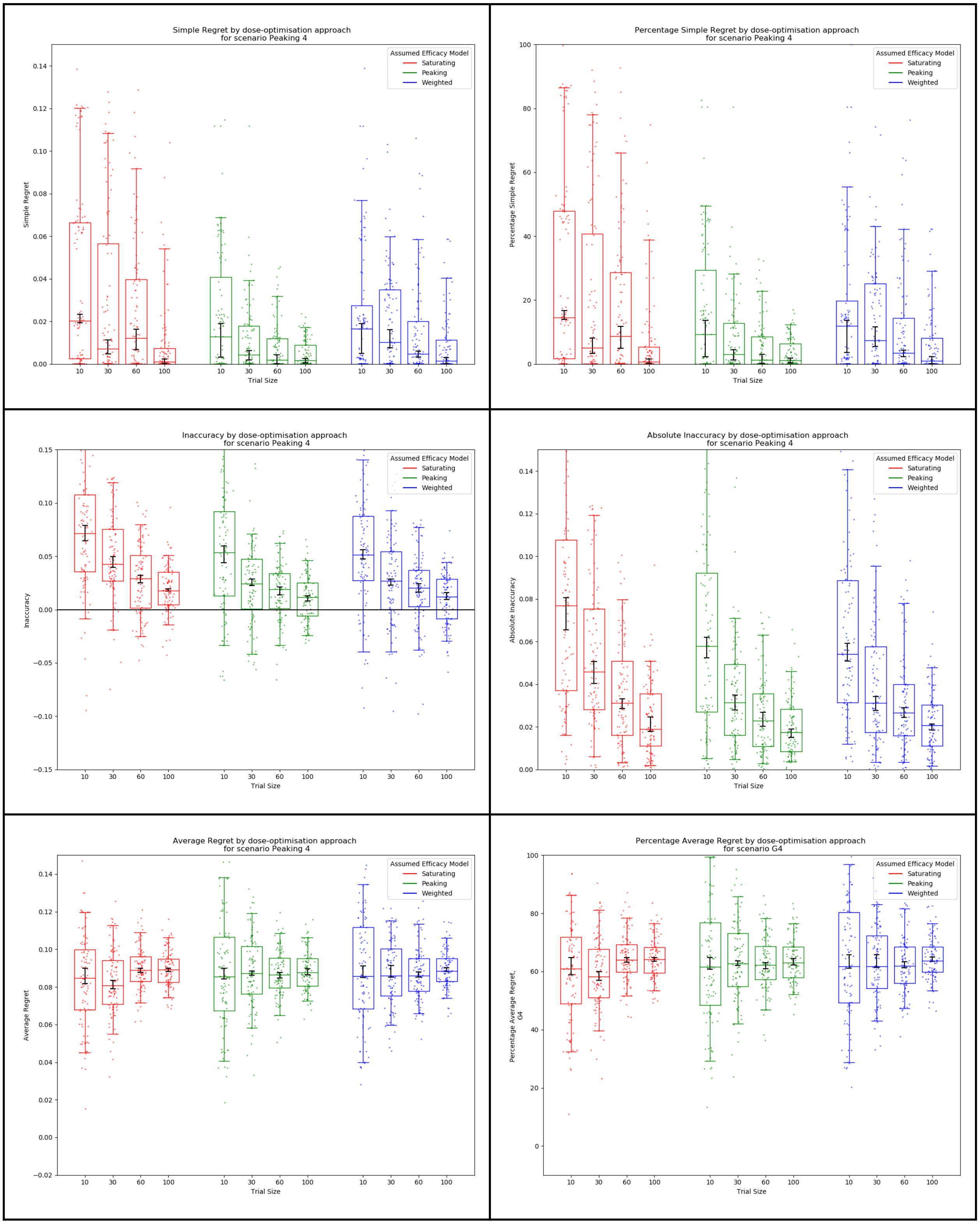

Figure Supplementary.Obj1_Plots.Peaking 4. Plots of the metrics from simulations for dose-optimisation approaches in objective 1 for scenario Peaking 4. The shown metrics are simple regret (top left), percentage simple regret (top right), inaccuracy (middle left), absolute

inaccuracy (middle right), average regret (bottom left), and percentage average regret (bottom right).

Scenario Peaking 5

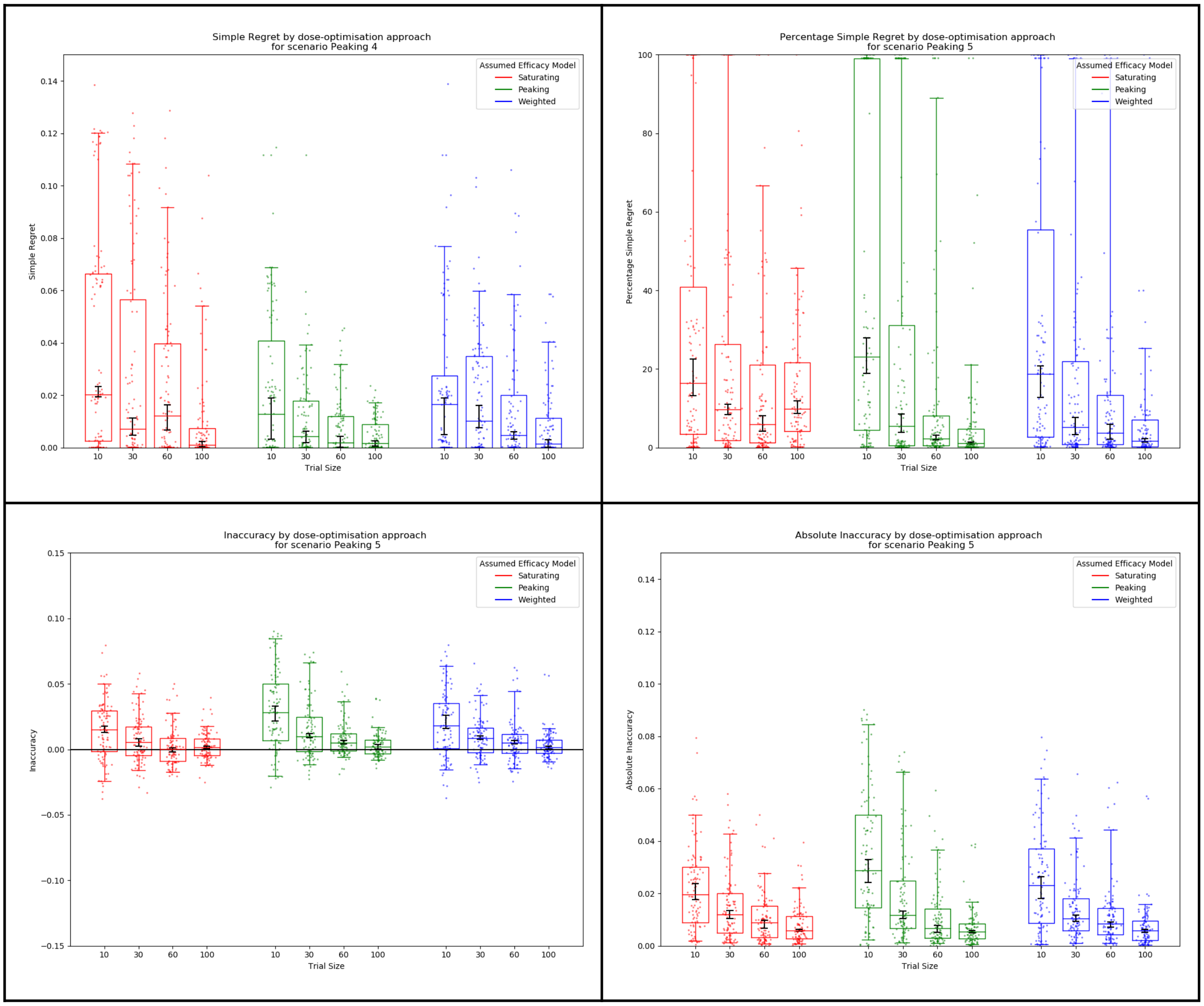

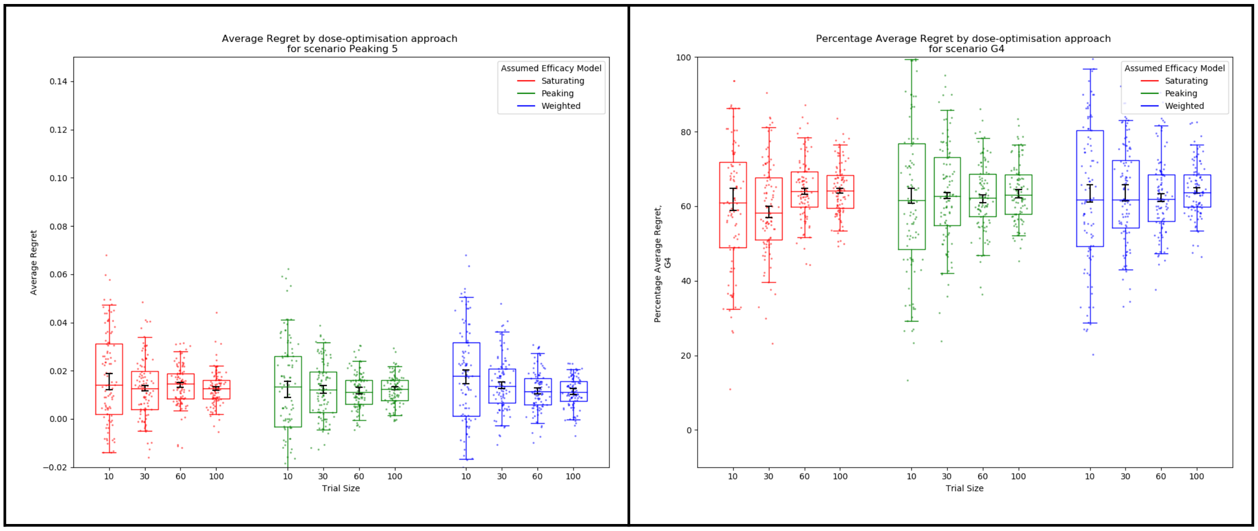

Figure Supplementary.Obj1_Plots.Peaking 5. Plots of the metrics from simulations for dose-optimisation approaches in objective 1 for scenario Peaking 5. The shown metrics are simple regret (top left), percentage simple regret (top right), inaccuracy (middle left), absolute inaccuracy (middle right), average regret (bottom left), and percentage average regret (bottom right).

Scenario Other 1

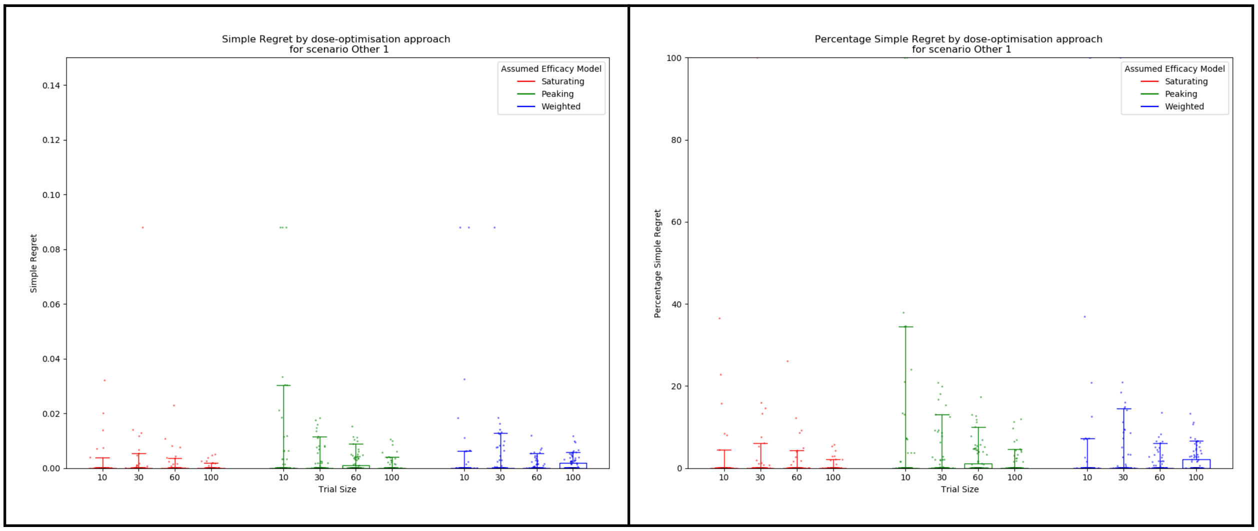

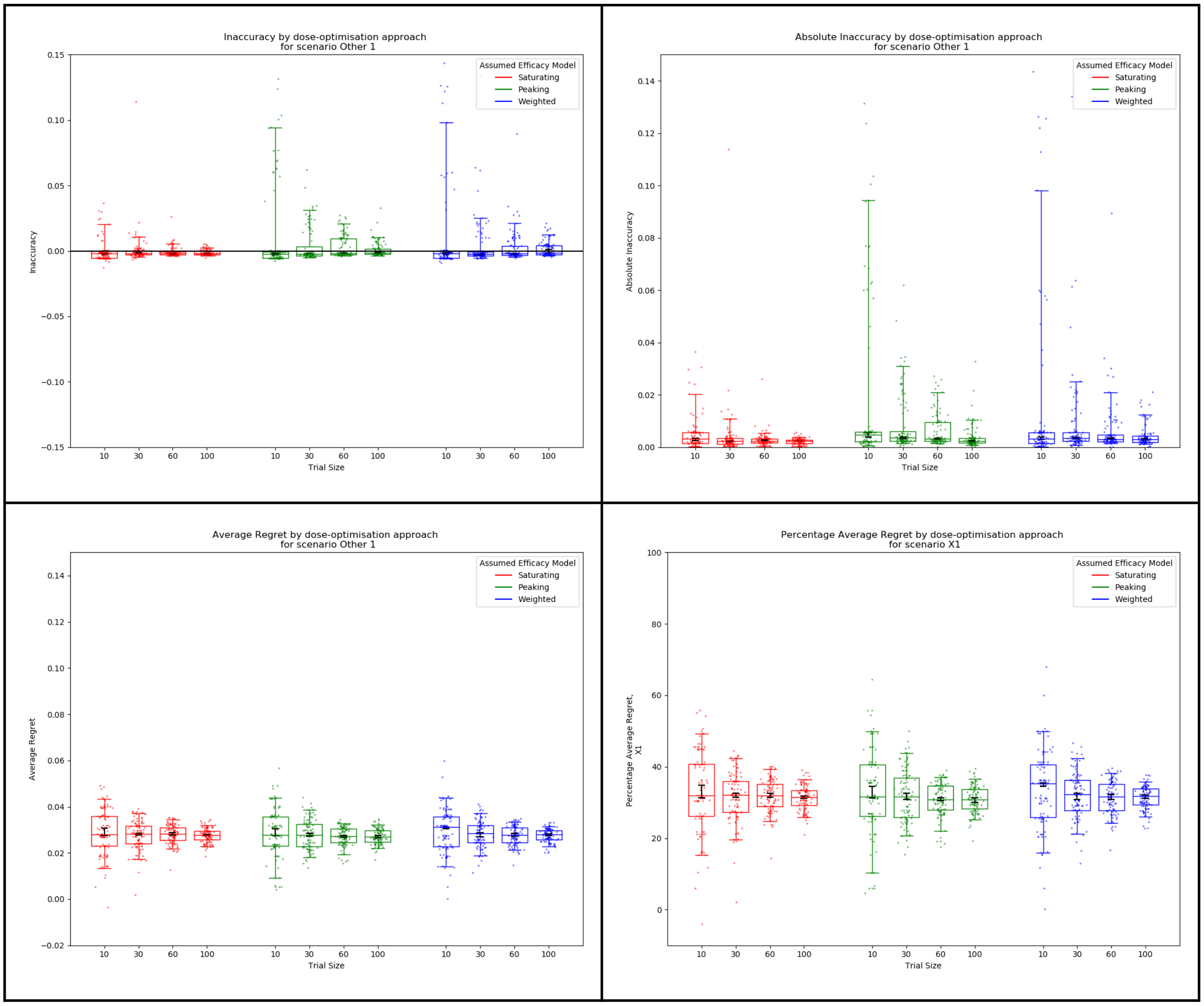

Figure Supplementary.Obj1_Plots.Other 1. Plots of the metrics from simulations for dose-optimisation approaches in objective 1 for scenario Other 1. The shown metrics are simple regret (top left), percentage simple regret (top right), inaccuracy (middle left), absolute inaccuracy (middle right), average regret (bottom left), and percentage average regret (bottom right).

Scenario Other 2

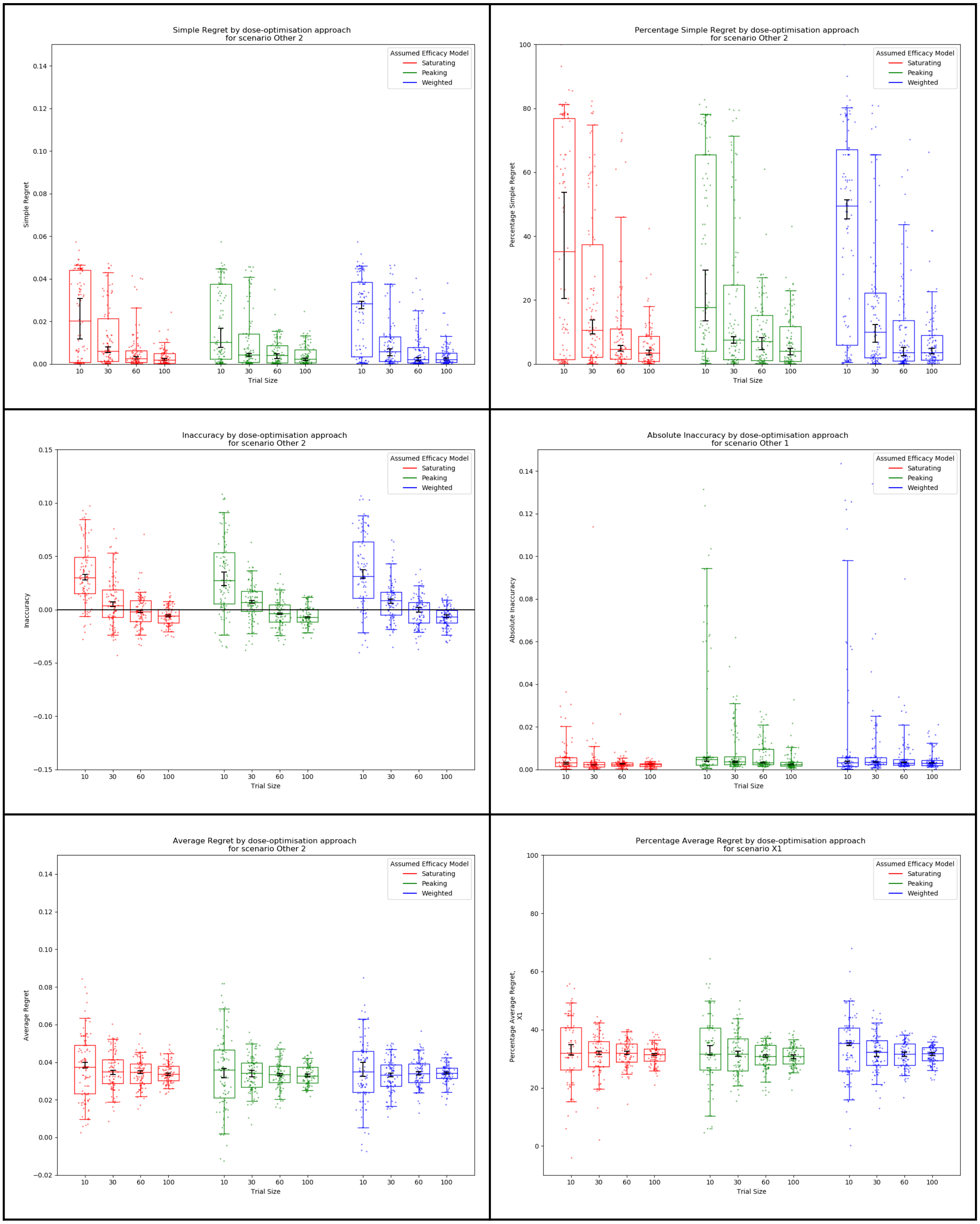

Figure Supplementary.Obj1_Plots.Other 2. Plots of the metrics from simulations for dose-optimisation approaches in objective 1 for scenario Other 2. The shown metrics are simple regret (top left), percentage simple regret (top right), inaccuracy (middle left), absolute

inaccuracy (middle right), average regret (bottom left), and percentage average regret (bottom right).

Scenario Other 3

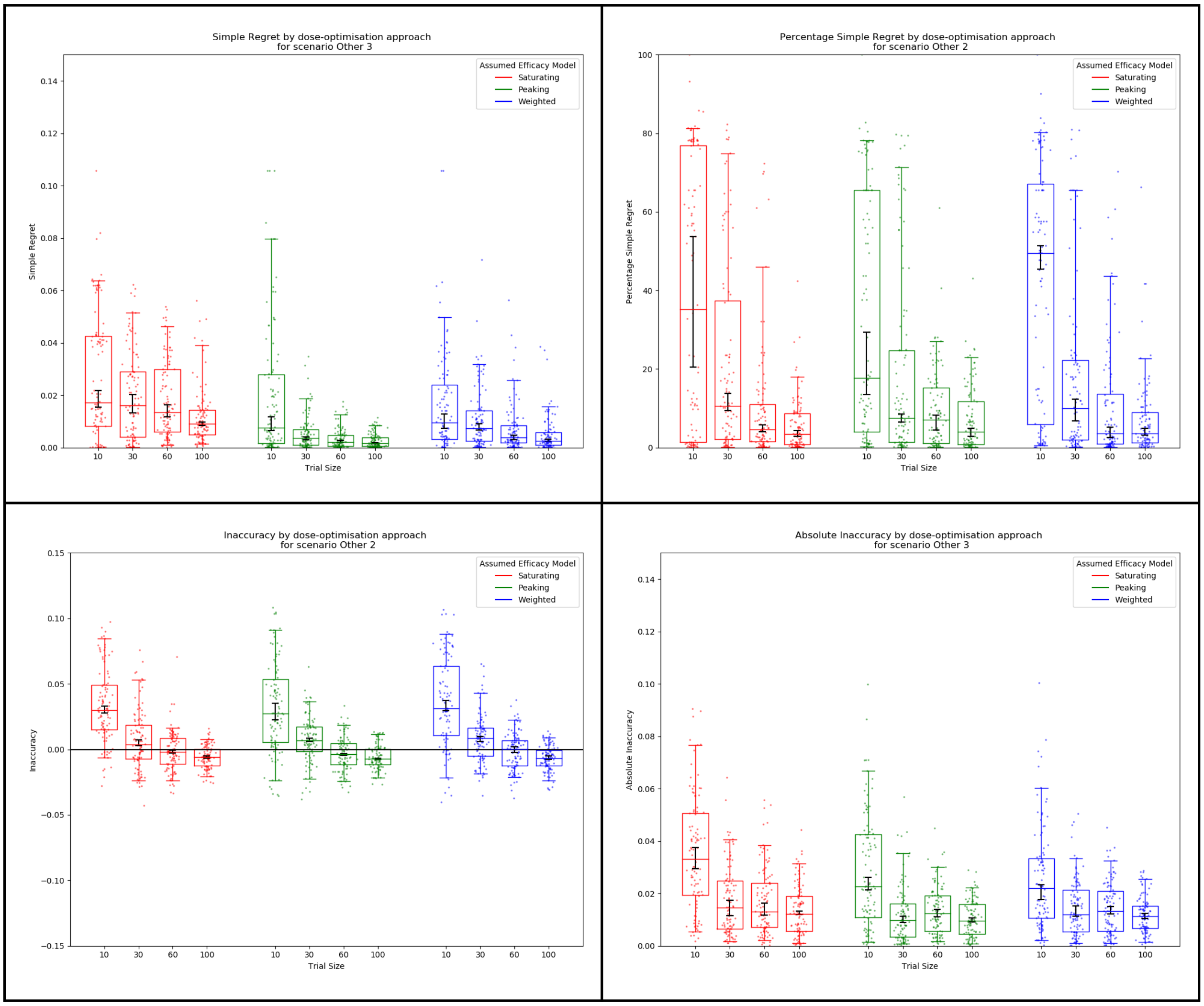

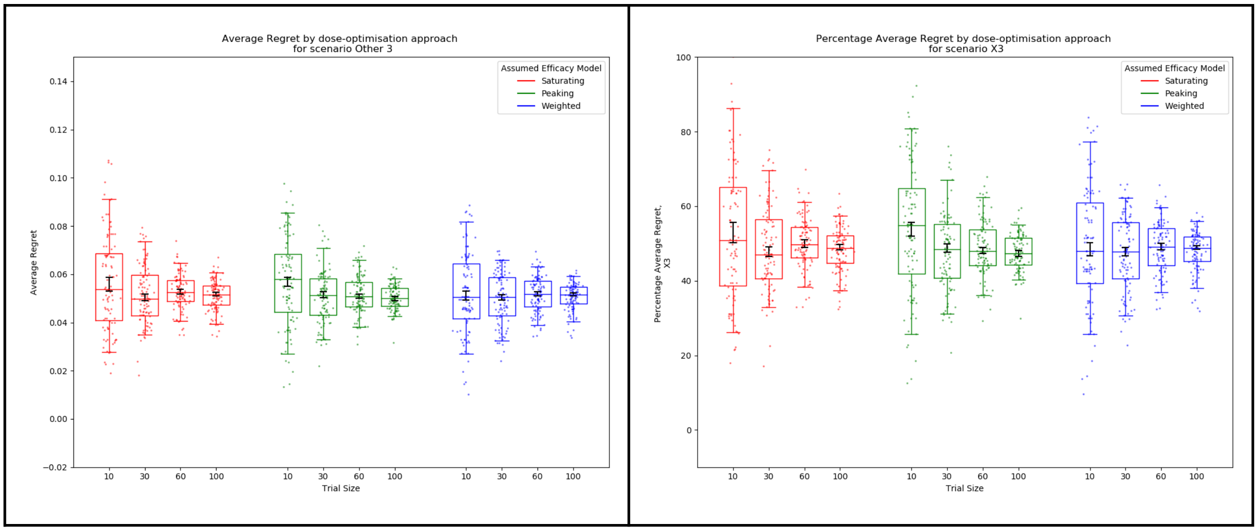

Figure Supplementary.Obj1_Plots.Other 3. Plots of the metrics from simulations for dose-optimisation approaches in objective 1 for scenario Other 3. The shown metrics are simple regret (top left), percentage simple regret (top right), inaccuracy (middle left), absolute inaccuracy (middle right), average regret (bottom left), and percentage average regret (bottom right).

Scenario Other 4

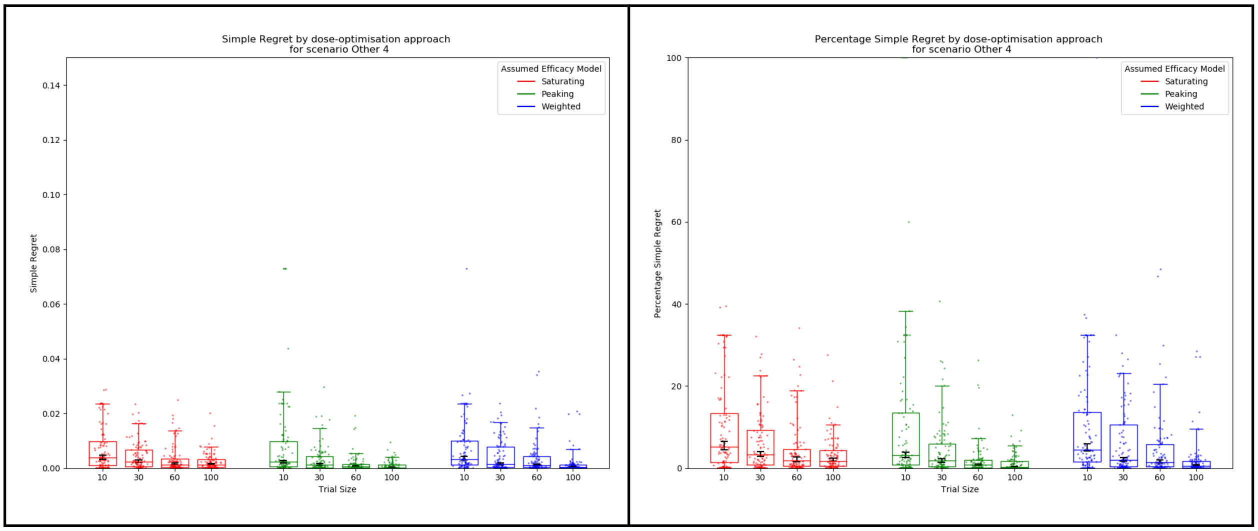

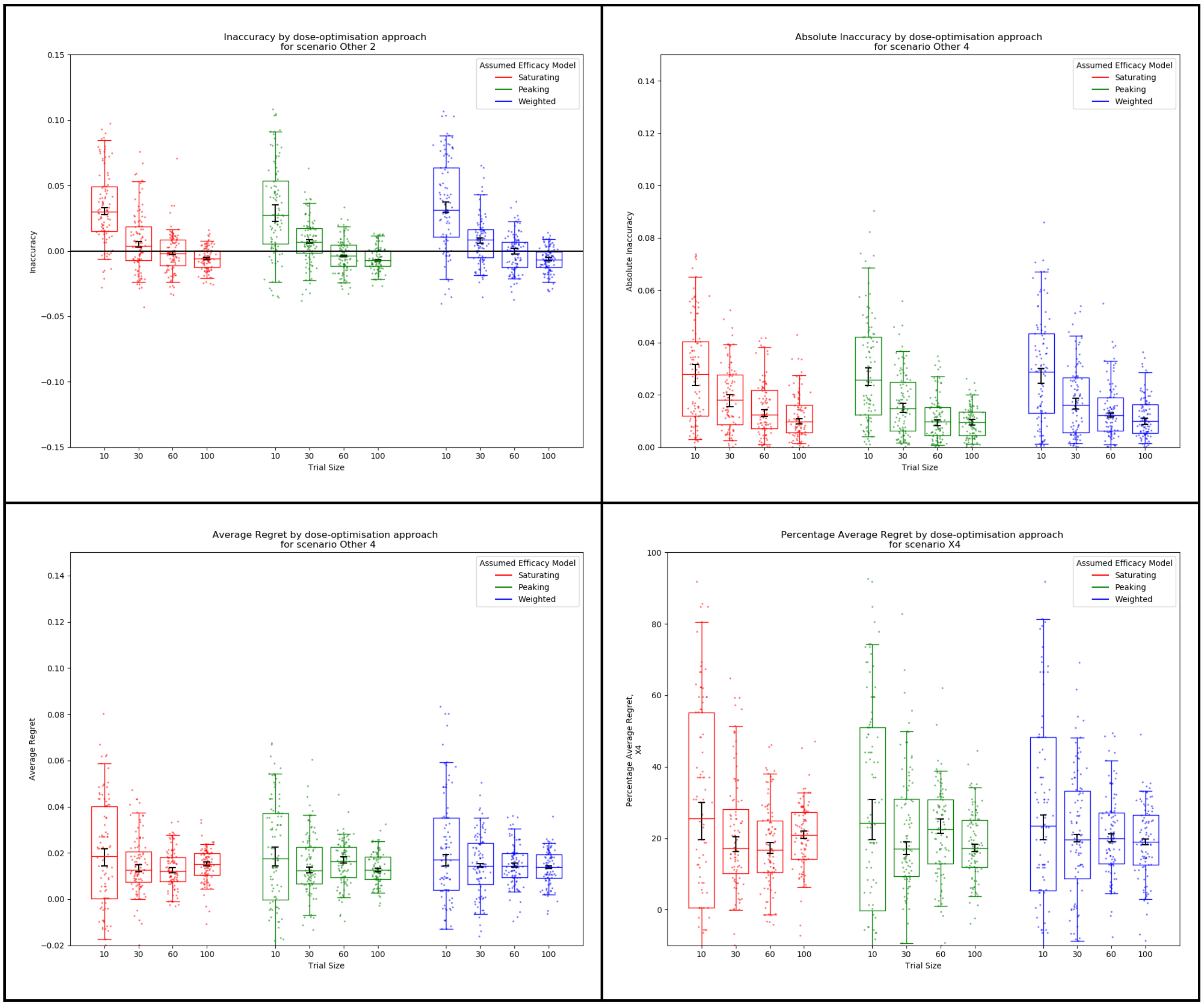

Figure Supplementary.Obj1_Plots.Other 4. Plots of the metrics from simulations for dose-optimisation approaches in objective 1 for scenario Other 4. The shown metrics are simple regret (top left), percentage simple regret (top right), inaccuracy (middle left), absolute inaccuracy (middle right), average regret (bottom left), and percentage average regret (bottom right).

Supplementary 12. Statistical Analysis

Here we provide a statistical analysis of the data presented in the main body and supplementary sections of this work. We provide analysis in two ways.

1. The Kolmogorov–Smirnov Test is used to determine whether there is evidence to support two samples being drawn from the same distribution or from different distributions [5].

2. The One-sided Mann-Whitney U Test is used to test whether values in one sample tend to be larger/smaller than another [6].

For each test we present a heatmap of p-values, with each cell in the heat map containing the p-value for that test for the comparison between the sample metrics for the dose-optimisation approach in the respective row/column. Cells in the table with a light pink hue represent the test statistic for that comparison would be significant under the threshold $p<0.05$, cells with a red hue represent the test statistic for that comparison would be significant under the threshold $p<0.05$ with Bonferri multiple comparison correction [7]. This threshold was $p<0.00076 = 0.05/66$ (66 = 12C2 numbers of unique pairings of 12 approaches in each objective)

We believe that the qualitative analysis and Copeland metrics presented in the body of the work and Supplementary sections 10-13 are more relevant for showing practical differences between the dose-optimisation approaches than the statistical analysis presented in this section. These analyses are only presented for interest.

Objective 1 Total Analysis

Here we show the p-values for objective 1 for the metrics of PSR, Absolute Inaccuracy, and PAR. These are the metrics for the combined data of all scenarios. This is the data in figures 7, 8b and 9 respectively.

For interpretation, the Kolmogorov–Smirnov heatmaps are symmetric, with significance representing evidence that the true distribution for the approach-scenario test metrics of PSR, Absolute Inaccuracy, and PAR differ between the two dose-optimisation approaches across all scenarios. The One-sided Mann-Whitney U test heatmaps are not symmetric, with significance for the cell in row A and column B representing 'statistically significant' evidence that approach A was preferable to approach B with regards to that metric (eg. lower PSR, lower Absolute Inaccuracy, Lower PAR).

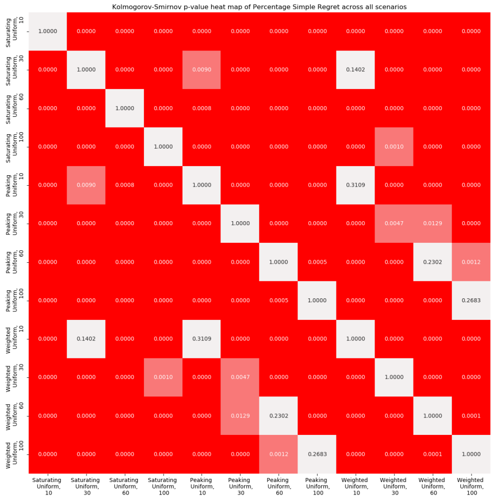
Kolmogorov-Smirnov p-value heat map of Percentage Simple Regret across all scenarios
Saturating Uniform, 10
Saturating Uniform, 30
Saturating Uniform, 60
Saturating Uniform, 100
Peaking Uniform, 10
Peaking Uniform, 30
Peaking Uniform, 60
Peaking Uniform, 100
Weighted Uniform, 10
Weighted Uniform, 30
Weighted Uniform, 60
Weighted Uniform, 100

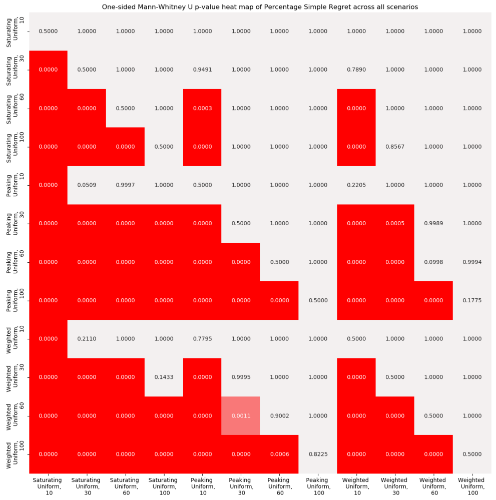
One-sided Mann-Whitney U p-value heat map of Percentage Simple Regret across all scenarios
Saturating Uniform, 10
Saturating Uniform, 30
Saturating Uniform, 60
Saturating Uniform, 100
Peaking Uniform, 10
Peaking Uniform, 30
Peaking Uniform, 60
Peaking Uniform, 100
Weighted Uniform, 10
Weighted Uniform, 30
Weighted Uniform, 60
Weighted Uniform, 100

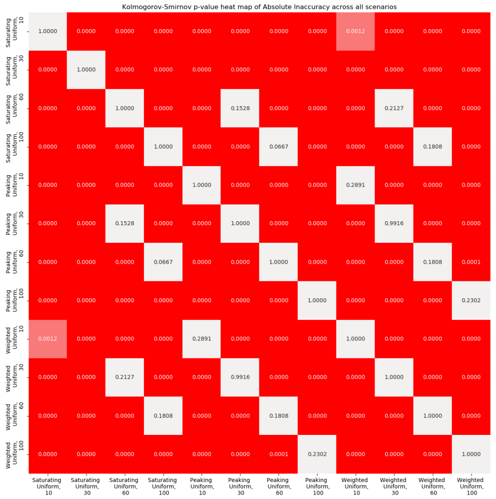
Kolmogorov-Smirnov p-value heat map of Absolute Inaccuracy across all scenarios
Saturating Uniform, 10
Saturating Uniform, 30
Saturating Uniform, 60
Saturating Uniform, 100
Peaking Uniform, 10
Peaking Uniform, 30
Peaking Uniform, 60
Peaking Uniform, 100
Weighted Uniform, 10
Weighted Uniform, 30
Weighted Uniform, 60
Weighted Uniform, 100

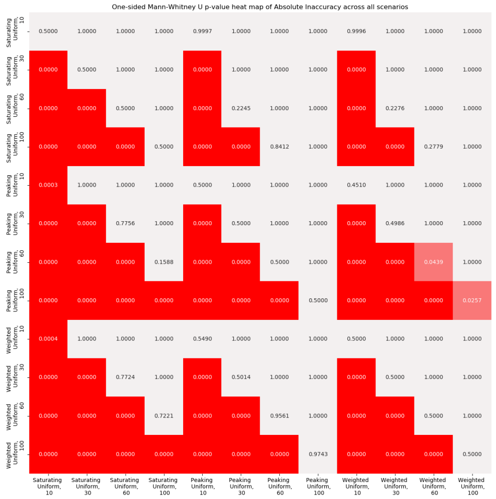
One-sided Mann-Whitney U p-value heat map of Absolute Inaccuracy across all scenarios
Saturating Uniform, 10
Saturating Uniform, 30
Saturating Uniform, 60
Saturating Uniform, 100
Peaking Uniform, 10
Peaking Uniform, 30
Peaking Uniform, 60
Peaking Uniform, 100
Weighted Uniform, 10
Weighted Uniform, 30
Weighted Uniform, 60
Weighted Uniform, 100

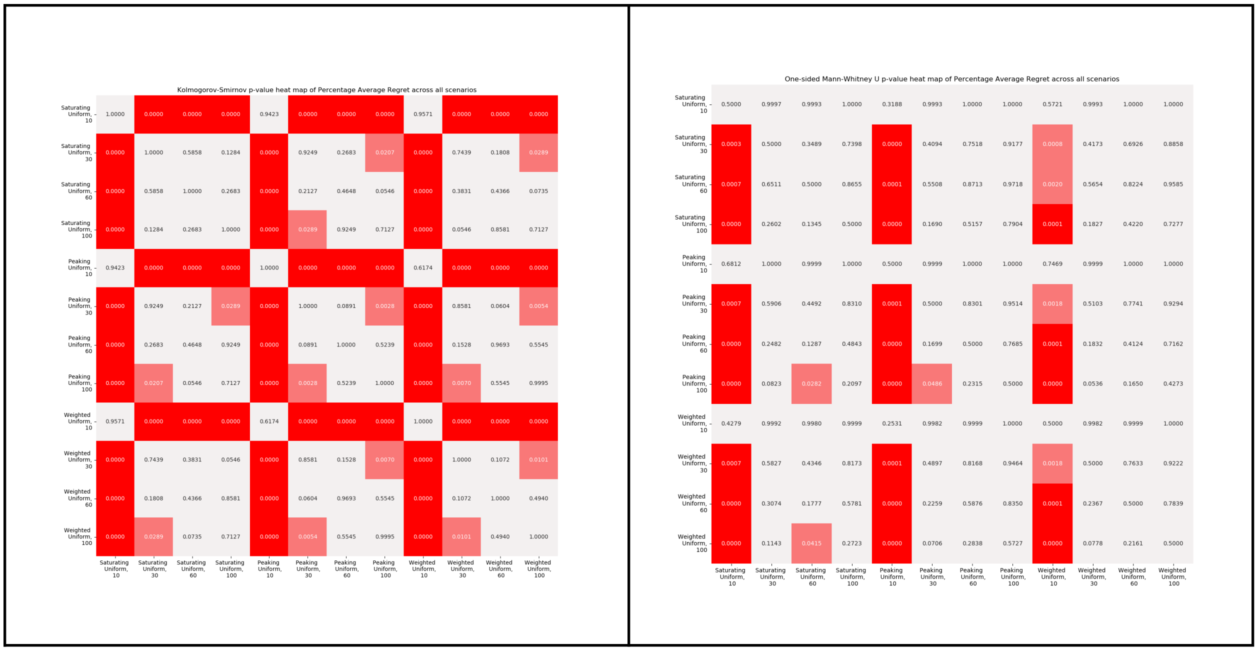

Figure Supplementary.Statistical.Obj1.Total. Kolmogorov–Smirnov (left) and Mann-Whitney U (right) heatmaps of p-values for objective 1 across all scenarios. These are for the metrics of PSR (top), Absolute Inaccuracy (middle) and PAR (bottom). Cells with a light pink hue represent the test statistic for that comparison would be significant under the threshold p<0.05, cells with a red hue represent the test statistic for that comparison would be significant under the threshold p<0.05 with Bonferri multiple comparison correction.

Objective 1 Scenario Specific

Here we show the p-values for objective 1 for the metrics of PSR, Absolute Inaccuracy, and PAR. These are the metrics for the data stratified on scenario. This is the data in Supplementary 11.

For interpretation, the Kolmogorov–Smirnov heatmaps are symmetric, with significance representing evidence that the true distribution for the approach-scenario test metrics of PSR, Absolute Inaccuracy, and PAR differ between the two dose-optimisation approaches across all scenarios. The One-sided Mann-Whitney U test heatmaps are not symmetric, with significance for the cell in row A and column B representing ‘statistically significant’ evidence that approach A was preferable to approach B with regards to that metric(eg. lower PSR, lower Absolute Inaccuracy, Lower PAR).

Scenario Saturating 1

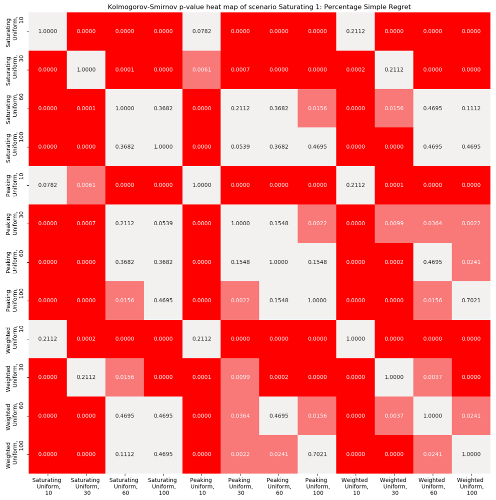

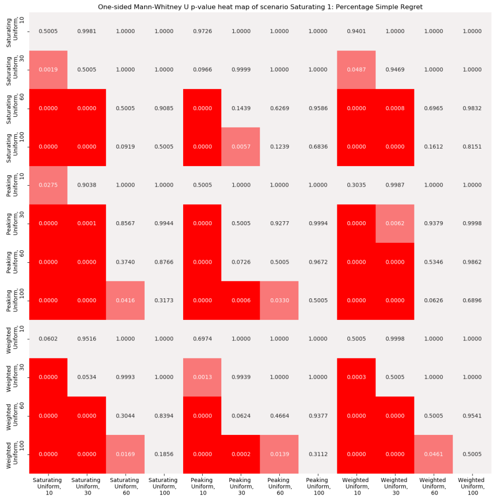

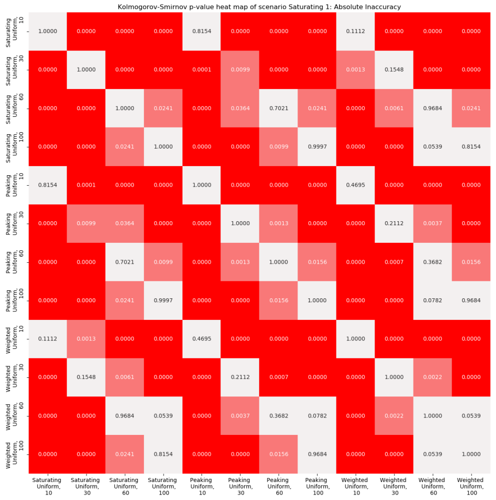

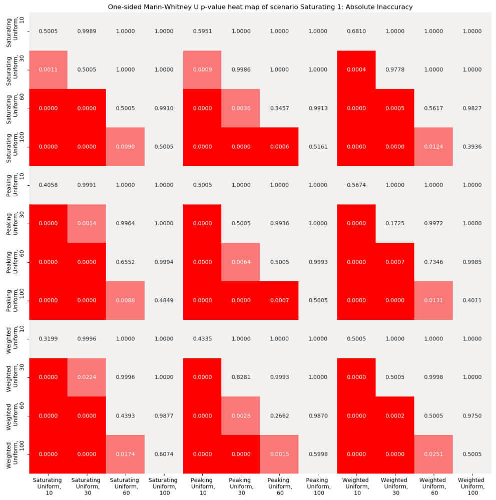

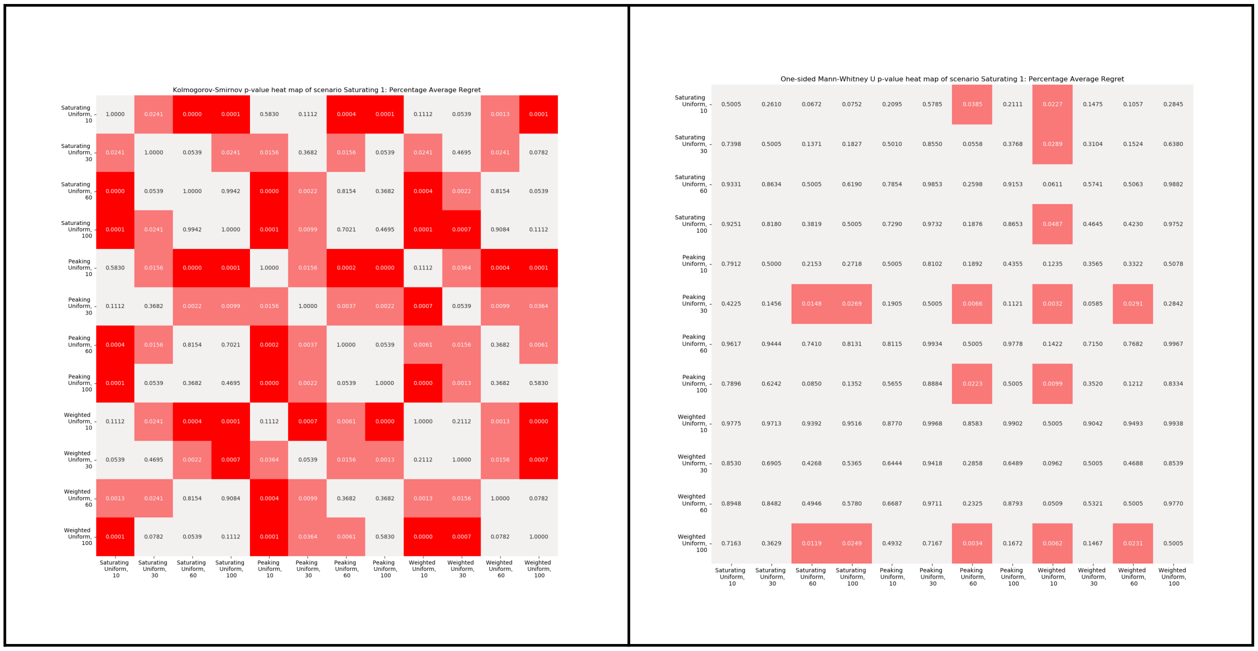

Figure Supplementary.Statistical.Obj1.S1. Kolmogorov–Smirnov (left) and Mann-Whitney U (right) heatmaps of p-values for objective 1, Scenario Saturating 1. These are for the metrics of PSR (top), Absolute Inaccuracy (middle) and PAR (bottom). Cells with a light pink hue represent the test statistic for that comparison would be significant under the threshold p<0.05, cells with a red hue represent the test statistic for that comparison would be significant under the threshold p<0.05 with Bonferri multiple comparison correction.

Scenario Saturating 2

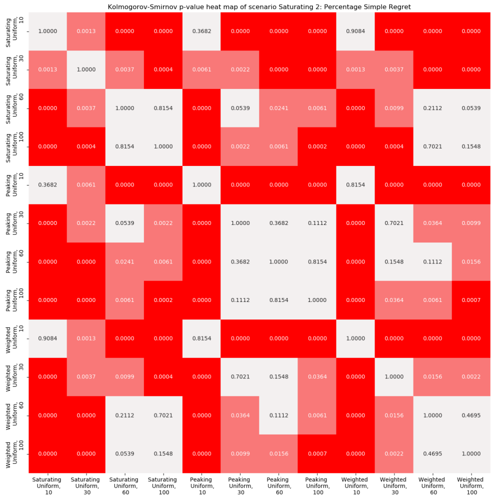
Kolmogorov-Smirnov p-value heat map of scenario Saturating 2: Percentage Simple Regret

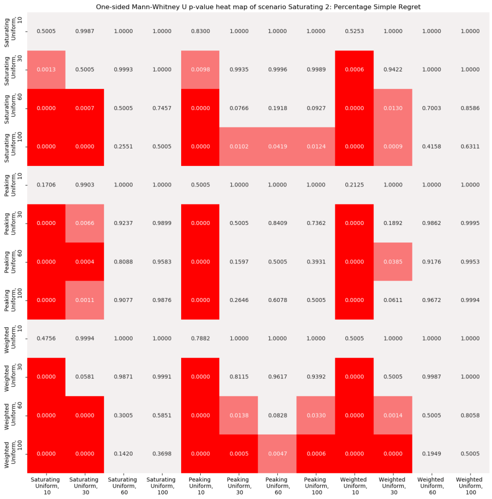
One-sided Mann-Whitney U p-value heat map of scenario Saturating 2: Percentage Simple Regret

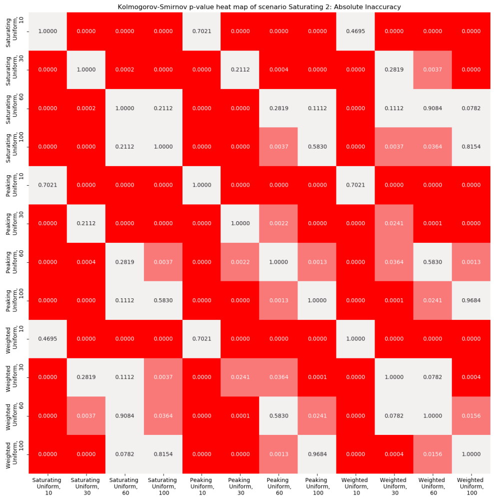
Kolmogorov-Smirnov p-value heat map of scenario Saturating 2: Absolute Inaccuracy

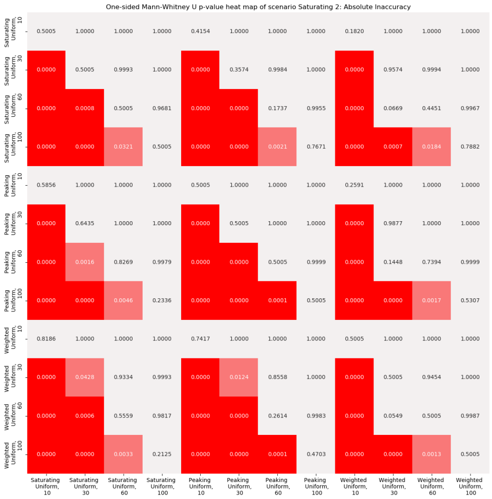
One-sided Mann-Whitney U p-value heat map of scenario Saturating 2: Absolute Inaccuracy

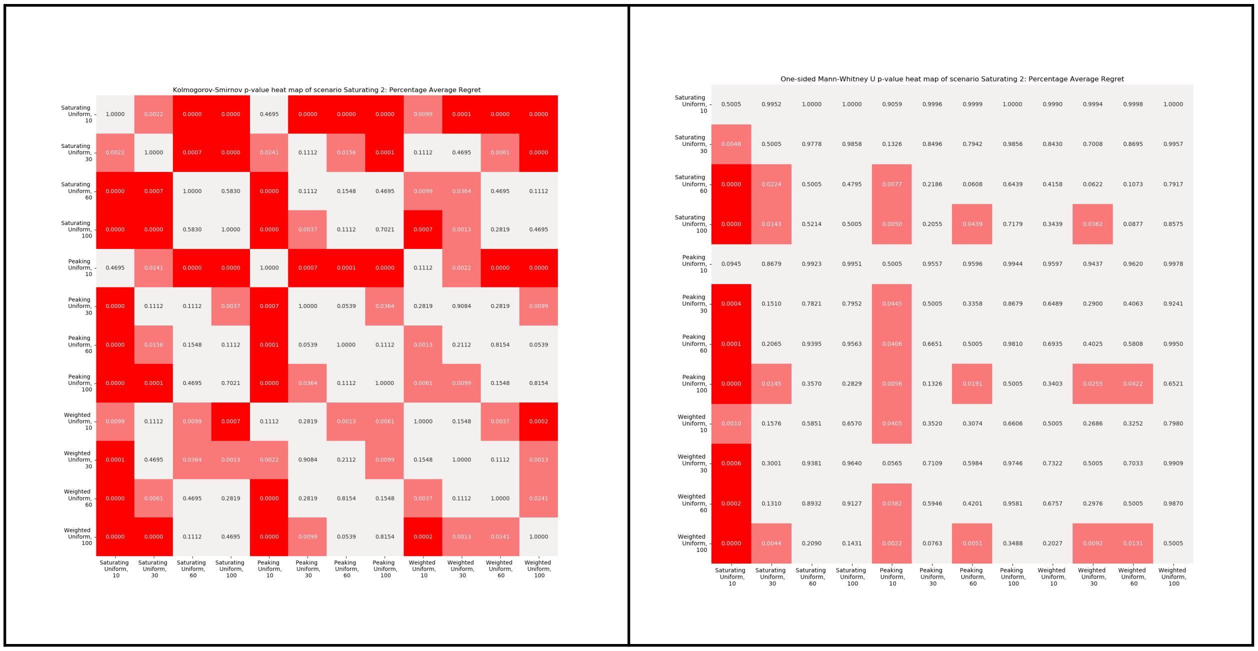

Figure Supplementary.Statistical.Obj1.S2. Kolmogorov–Smirnov (left) and Mann-Whitney U (right) heatmaps of p-values for objective 1, Scenario Saturating 2. These are for the metrics of PSR (top), Absolute Inaccuracy (middle) and PAR (bottom). Cells with a light pink hue represent the test statistic for that comparison would be significant under the threshold p<0.05, cells with a red hue represent the test statistic for that comparison would be significant under the threshold p<0.05 with Bonferri multiple comparison correction.

Scenario Saturating 3

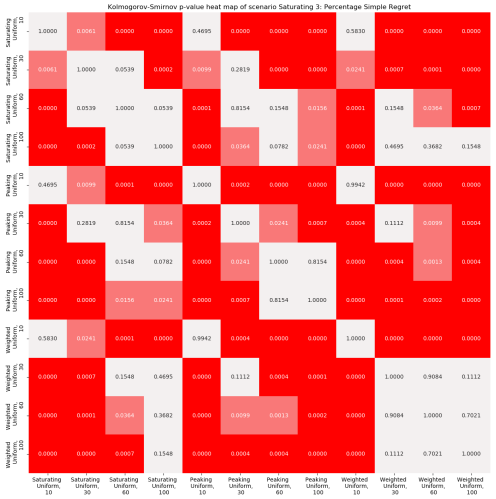
Kolmogorov-Smirnov p-value heat map of scenario Saturating 3: Percentage Simple Regret
Saturating Uniform, 10
Saturating Uniform, 30
Saturating Uniform, 60
Saturating Uniform, 100
Peaking Uniform, 10
Peaking Uniform, 30
Peaking Uniform, 60
Peaking Uniform, 100
Weighted Uniform, 10
Weighted Uniform, 30
Weighted Uniform, 60
Weighted Uniform, 100

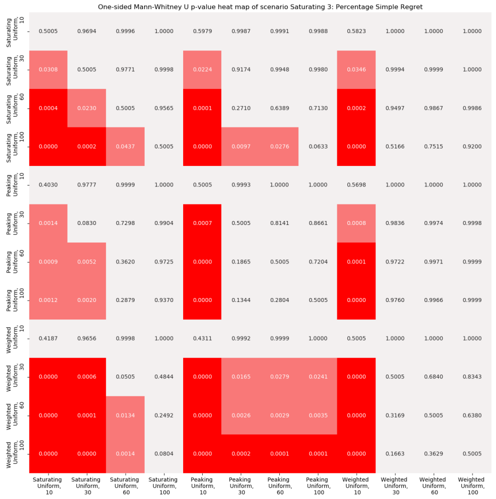
One-sided Mann-Whitney U p-value heat map of scenario Saturating 3: Percentage Simple Regret
Saturating Uniform, 10
Saturating Uniform, 30
Saturating Uniform, 60
Saturating Uniform, 100
Peaking Uniform, 10
Peaking Uniform, 30
Peaking Uniform, 60
Peaking Uniform, 100
Weighted Uniform, 10
Weighted Uniform, 30
Weighted Uniform, 60
Weighted Uniform, 100

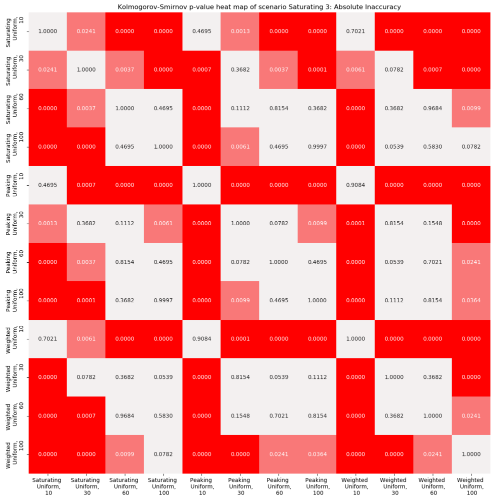
Kolmogorov-Smirnov p-value heat map of scenario Saturating 3: Absolute Inaccuracy
Saturating Uniform, 10
Saturating Uniform, 30
Saturating Uniform, 60
Saturating Uniform, 100
Peaking Uniform, 10
Peaking Uniform, 30
Peaking Uniform, 60
Peaking Uniform, 100
Weighted Uniform, 10
Weighted Uniform, 30
Weighted Uniform, 60
Weighted Uniform, 100

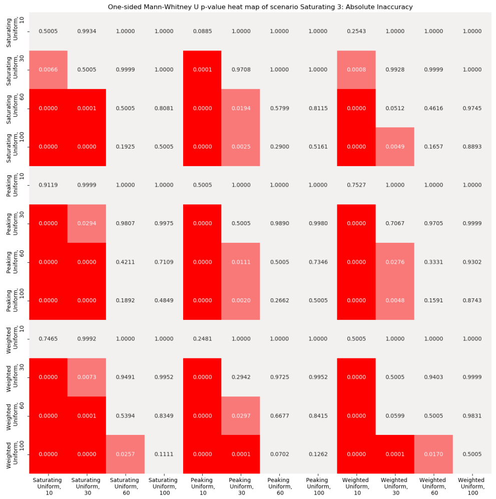
One-sided Mann-Whitney U p-value heat map of scenario Saturating 3: Absolute Inaccuracy
Saturating Uniform, 10
Saturating Uniform, 30
Saturating Uniform, 60
Saturating Uniform, 100
Peaking Uniform, 10
Peaking Uniform, 30
Peaking Uniform, 60
Peaking Uniform, 100
Weighted Uniform, 10
Weighted Uniform, 30
Weighted Uniform, 60
Weighted Uniform, 100

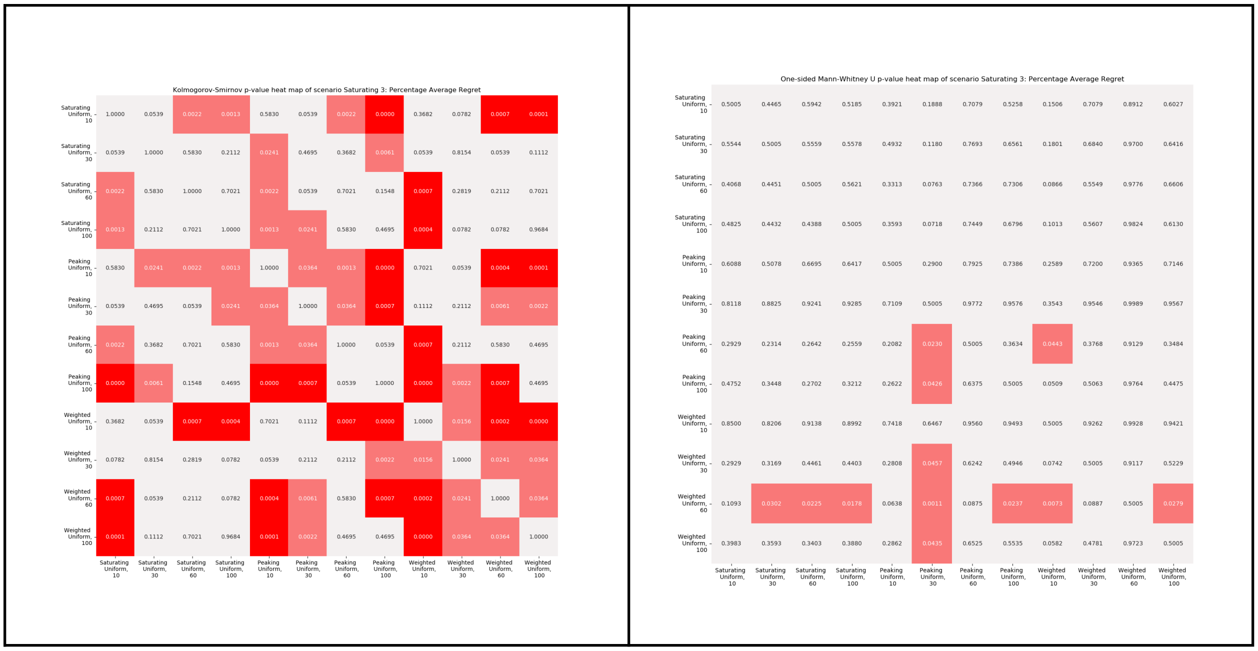

Figure Supplementary.Statistical.Obj1.S3. Kolmogorov–Smirnov (left) and Mann-Whitney U (right) heatmaps of p-values for objective 1, Scenario Saturating 3. These are for the metrics of PSR (top), Absolute Inaccuracy (middle) and PAR (bottom). Cells with a light pink hue represent the test statistic for that comparison would be significant under the threshold p<0.05, cells with a red hue represent the test statistic for that comparison would be significant under the threshold p<0.05 with Bonferri multiple comparison correction.

Scenario Saturating 4

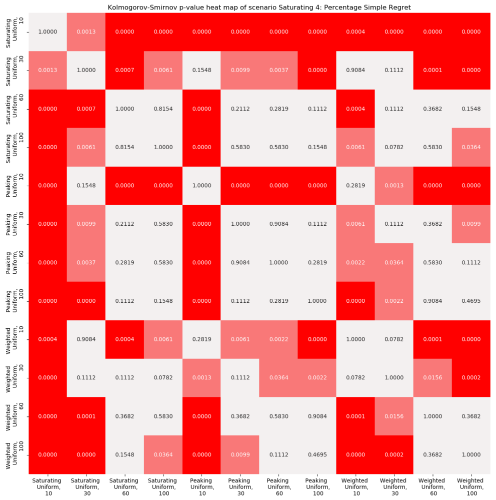
Kolmogorov-Smirnov p-value heat map of scenario Saturating 4: Percentage Simple Regret
Saturating Uniform, 10
Saturating Uniform, 30
Saturating Uniform, 60
Saturating Uniform, 100
Peaking Uniform, 10
Peaking Uniform, 30
Peaking Uniform, 60
Peaking Uniform, 100
Weighted Uniform, 10
Weighted Uniform, 30
Weighted Uniform, 60
Weighted Uniform, 100

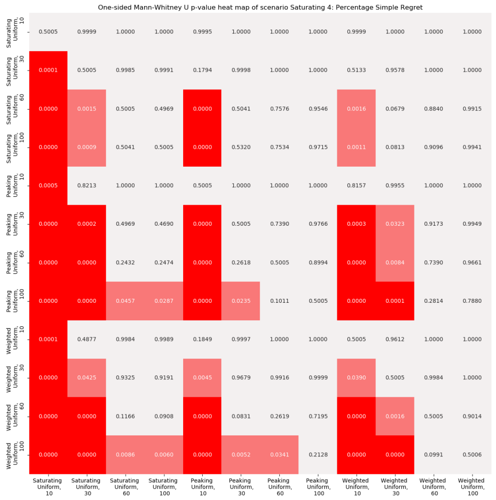
One-sided Mann-Whitney U p-value heat map of scenario Saturating 4: Percentage Simple Regret
Saturating Uniform, 10
Saturating Uniform, 30
Saturating Uniform, 60
Saturating Uniform, 100
Peaking Uniform, 10
Peaking Uniform, 30
Peaking Uniform, 60
Peaking Uniform, 100
Weighted Uniform, 10
Weighted Uniform, 30
Weighted Uniform, 60
Weighted Uniform, 100

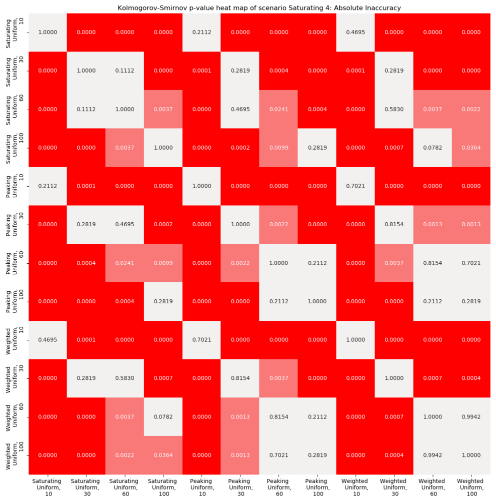
Kolmogorov-Smirnov p-value heat map of scenario Saturating 4: Absolute Inaccuracy
Saturating Uniform, 10
Saturating Uniform, 30
Saturating Uniform, 60
Saturating Uniform, 100
Peaking Uniform, 10
Peaking Uniform, 30
Peaking Uniform, 60
Peaking Uniform, 100
Weighted Uniform, 10
Weighted Uniform, 30
Weighted Uniform, 60
Weighted Uniform, 100

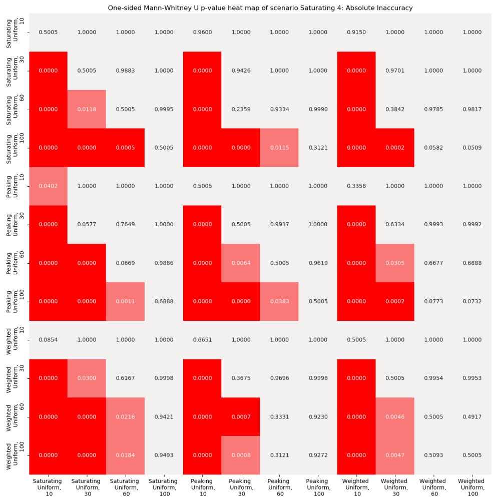
One-sided Mann-Whitney U p-value heat map of scenario Saturating 4: Absolute Inaccuracy
Saturating Uniform, 10
Saturating Uniform, 30
Saturating Uniform, 60
Saturating Uniform, 100
Peaking Uniform, 10
Peaking Uniform, 30
Peaking Uniform, 60
Peaking Uniform, 100
Weighted Uniform, 10
Weighted Uniform, 30
Weighted Uniform, 60
Weighted Uniform, 100

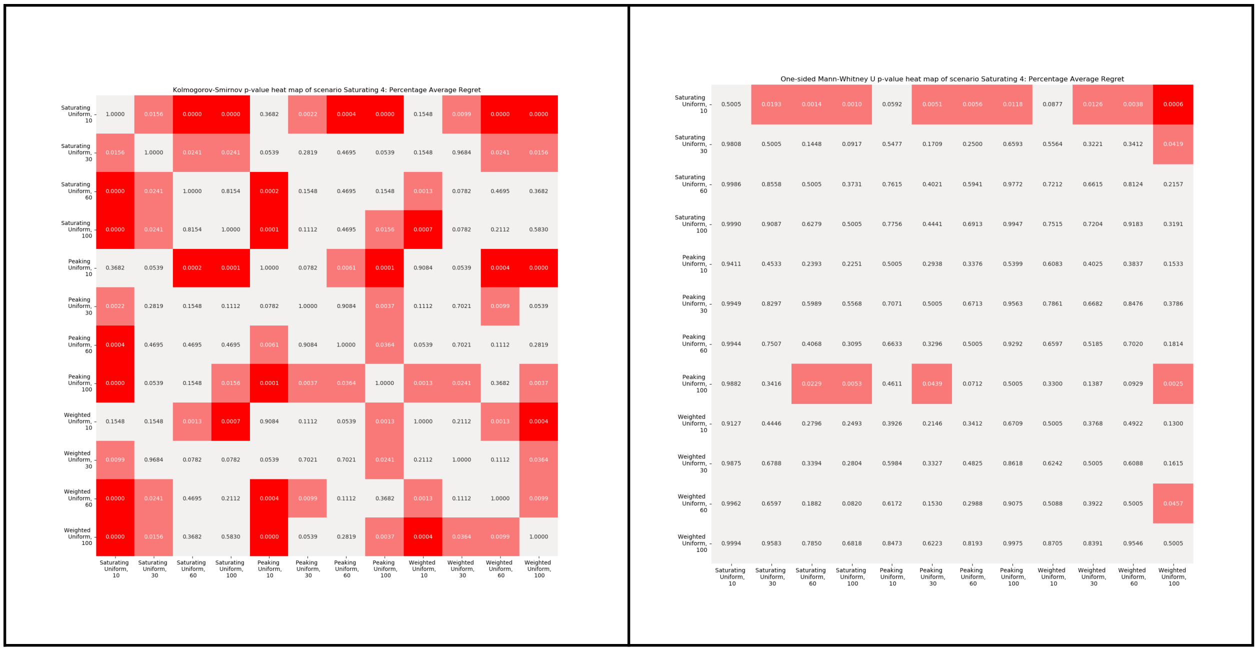

Figure Supplementary.Statistical.Obj1.S4. Kolmogorov–Smirnov (left) and Mann-Whitney U (right) heatmaps of p-values for objective 1, Scenario Saturating 4. These are for the metrics of PSR (top), Absolute Inaccuracy (middle) and PAR (bottom). Cells with a light pink hue represent the test statistic for that comparison would be significant under the threshold p<0.05, cells with a red hue represent the test statistic for that comparison would be significant under the threshold p<0.05 with Bonferri multiple comparison correction.

Scenario Saturating 5

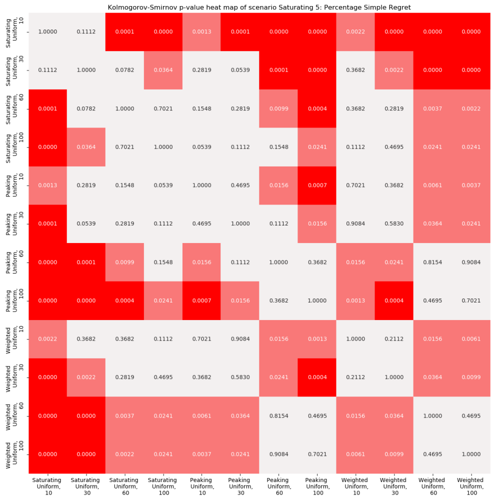
Kolmogorov-Smirnov p-value heat map of scenario Saturating 5: Percentage Simple Regret
Saturating Uniform, 10
Saturating Uniform, 30
Saturating Uniform, 60
Saturating Uniform, 100
Peaking Uniform, 10
Peaking Uniform, 30
Peaking Uniform, 60
Peaking Uniform, 100
Weighted Uniform, 10
Weighted Uniform, 30
Weighted Uniform, 60
Weighted Uniform, 100

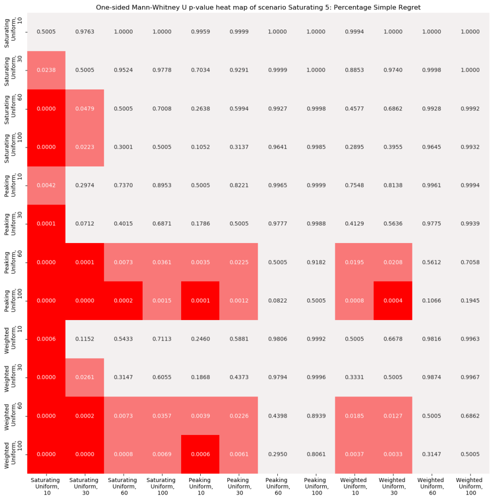
One-sided Mann-Whitney U p-value heat map of scenario Saturating 5: Percentage Simple Regret
Saturating Uniform, 10
Saturating Uniform, 30
Saturating Uniform, 60
Saturating Uniform, 100
Peaking Uniform, 10
Peaking Uniform, 30
Peaking Uniform, 60
Peaking Uniform, 100
Weighted Uniform, 10
Weighted Uniform, 30
Weighted Uniform, 60
Weighted Uniform, 100

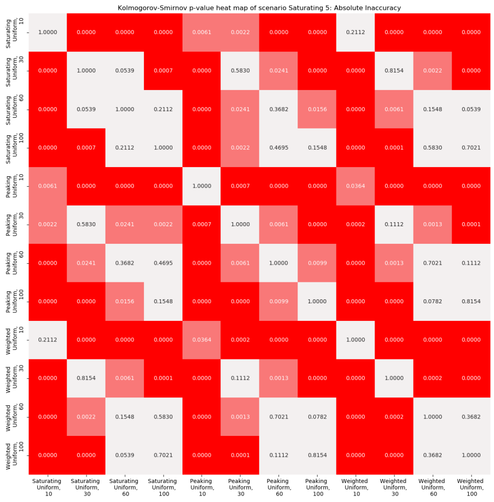
Kolmogorov-Smirnov p-value heat map of scenario Saturating 5: Absolute Inaccuracy

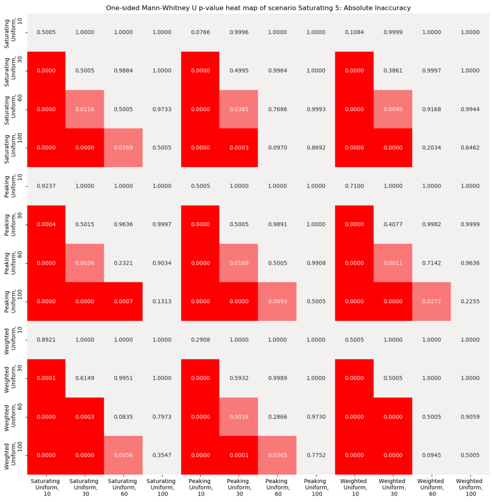
One-sided Mann-Whitney U p-value heat map of scenario Saturating 5: Absolute Inaccuracy

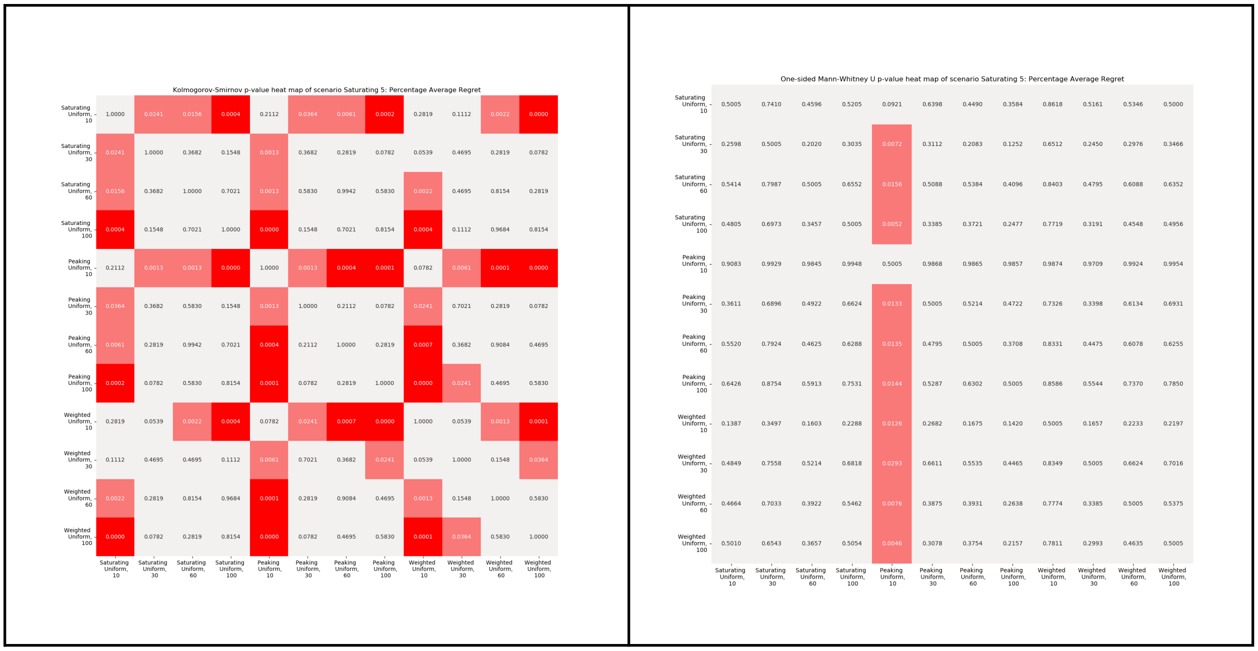

Figure Supplementary.Statistical.Obj1.S5. Kolmogorov–Smirnov (left) and Mann-Whitney U (right) heatmaps of p-values for objective 1, Scenario Saturating 5. These are for the metrics of PSR (top), Absolute Inaccuracy (middle) and PAR (bottom). Cells with a light pink hue represent the test statistic for that comparison would be significant under the threshold $p<0.05$, cells with a red hue represent the test statistic for that comparison would be significant under the threshold $p<0.05$ with Bonferri multiple comparison correction.

<u>Scenario Peaking 1</u>

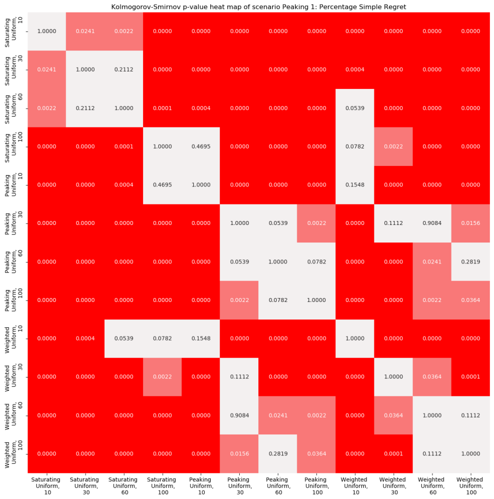
Kolmogorov-Smirnov p-value heat map of scenario Peaking 1: Percentage Simple Regret
Saturating Uniform, 10: 1.0000 0.0241 0.0022 0.0000 0.0000 0.0000 0.0000 0.0000 0.0000 0.0000 0.0000 0.0000
Saturating Uniform, 30: 0.0241 1.0000 0.2112 0.0000 0.0000 0.0000 0.0000 0.0000 0.0004 0.0000 0.0000 0.0000
Saturating Uniform, 60: 0.0022 0.2112 1.0000 0.0001 0.0004 0.0000 0.0000 0.0000 0.0539 0.0000 0.0000 0.0000
Saturating Uniform, 100: 0.0000 0.0000 0.0001 1.0000 0.4695 0.0000 0.0000 0.0000 0.0782 0.0022 0.0000 0.0000
Peaking Uniform, 10: 0.0000 0.0000 0.0004 0.4695 1.0000 0.0000 0.0000 0.0000 0.1548 0.0000 0.0000 0.0000
Peaking Uniform, 30: 0.0000 0.0000 0.0000 0.0000 0.0000 1.0000 0.0539 0.0022 0.0000 0.1112 0.9084 0.0156
Peaking Uniform, 60: 0.0000 0.0000 0.0000 0.0000 0.0000 0.0539 1.0000 0.0782 0.0000 0.0000 0.0241 0.2819
Peaking Uniform, 100: 0.0000 0.0000 0.0000 0.0000 0.0000 0.0022 0.0782 1.0000 0.0000 0.0000 0.0022 0.0364
Weighted Uniform, 10: 0.0000 0.0004 0.0539 0.0782 0.1548 0.0000 0.0000 0.0000 1.0000 0.0000 0.0000 0.0000
Weighted Uniform, 30: 0.0000 0.0000 0.0000 0.0022 0.0000 0.1112 0.0000 0.0000 0.0000 1.0000 0.0364 0.0001
Weighted Uniform, 60: 0.0000 0.0000 0.0000 0.0000 0.0000 0.9084 0.0241 0.0022 0.0000 0.0364 1.0000 0.1112
Weighted Uniform, 100: 0.0000 0.0000 0.0000 0.0000 0.0000 0.0156 0.2819 0.0364 0.0000 0.0001 0.1112 1.0000
Saturating Uniform, 10
Saturating Uniform, 30
Saturating Uniform, 60
Saturating Uniform, 100
Peaking Uniform, 10
Peaking Uniform, 30
Peaking Uniform, 60
Peaking Uniform, 100
Weighted Uniform, 10
Weighted Uniform, 30
Weighted Uniform, 60
Weighted Uniform, 100

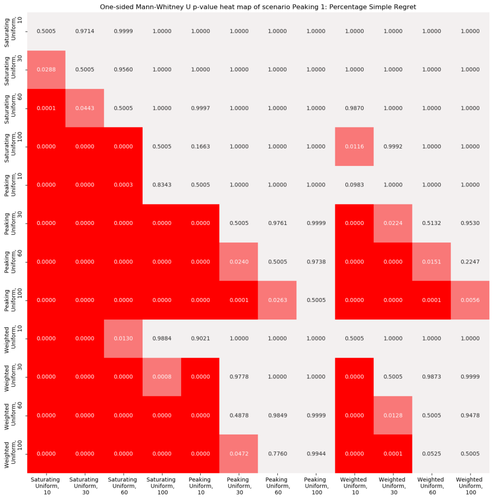
One-sided Mann-Whitney U p-value heat map of scenario Peaking 1: Percentage Simple Regret
Saturating Uniform, 10: 0.5005 0.9714 0.9999 1.0000 1.0000 1.0000 1.0000 1.0000 1.0000 1.0000 1.0000 1.0000
Saturating Uniform, 30: 0.0288 0.5005 0.9560 1.0000 1.0000 1.0000 1.0000 1.0000 1.0000 1.0000 1.0000 1.0000
Saturating Uniform, 60: 0.0001 0.0443 0.5005 1.0000 0.9997 1.0000 1.0000 1.0000 0.9870 1.0000 1.0000 1.0000
Saturating Uniform, 100: 0.0000 0.0000 0.0000 0.5005 0.1663 1.0000 1.0000 1.0000 0.0116 0.9992 1.0000 1.0000
Peaking Uniform, 10: 0.0000 0.0000 0.0003 0.8343 0.5005 1.0000 1.0000 1.0000 0.0983 1.0000 1.0000 1.0000
Peaking Uniform, 30: 0.0000 0.0000 0.0000 0.0000 0.0000 0.5005 0.9761 0.9999 0.0000 0.0224 0.5132 0.9530
Peaking Uniform, 60: 0.0000 0.0000 0.0000 0.0000 0.0000 0.0240 0.5005 0.9738 0.0000 0.0000 0.0151 0.2247
Peaking Uniform, 100: 0.0000 0.0000 0.0000 0.0000 0.0000 0.0001 0.0263 0.5005 0.0000 0.0000 0.0001 0.0056
Weighted Uniform, 10: 0.0000 0.0000 0.0130 0.9884 0.9021 1.0000 1.0000 1.0000 0.5005 1.0000 1.0000 1.0000
Weighted Uniform, 30: 0.0000 0.0000 0.0000 0.0008 0.0000 0.9778 1.0000 1.0000 0.0000 0.5005 0.9873 0.9999
Weighted Uniform, 60: 0.0000 0.0000 0.0000 0.0000 0.0000 0.4878 0.9849 0.9999 0.0000 0.0128 0.5005 0.9478
Weighted Uniform, 100: 0.0000 0.0000 0.0000 0.0000 0.0000 0.0472 0.7760 0.9944 0.0000 0.0001 0.0525 0.5005
Saturating Uniform, 10
Saturating Uniform, 30
Saturating Uniform, 60
Saturating Uniform, 100
Peaking Uniform, 10
Peaking Uniform, 30
Peaking Uniform, 60
Peaking Uniform, 100
Weighted Uniform, 10
Weighted Uniform, 30
Weighted Uniform, 60
Weighted Uniform, 100

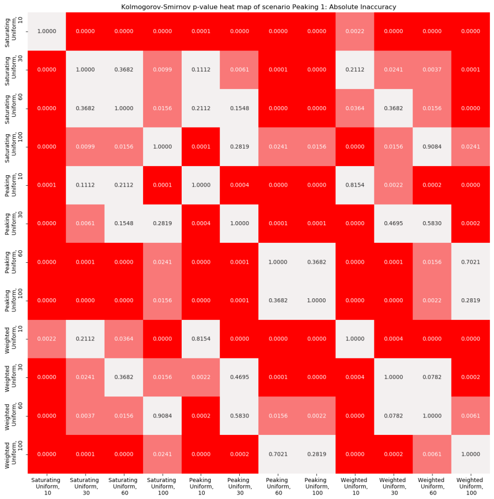
Kolmogorov-Smirnov p-value heat map of scenario Peaking 1: Absolute Inaccuracy
Saturating Uniform, 10: 1.0000 0.0000 0.0000 0.0000 0.0001 0.0000 0.0000 0.0000 0.0022 0.0000 0.0000 0.0000
Saturating Uniform, 30: 0.0000 1.0000 0.3682 0.0099 0.1112 0.0061 0.0001 0.0000 0.2112 0.0241 0.0037 0.0001
Saturating Uniform, 60: 0.0000 0.3682 1.0000 0.0156 0.2112 0.1548 0.0000 0.0000 0.0364 0.3682 0.0156 0.0000
Saturating Uniform, 100: 0.0000 0.0099 0.0156 1.0000 0.0001 0.2819 0.0241 0.0156 0.0000 0.0156 0.9084 0.0241
Peaking Uniform, 10: 0.0001 0.1112 0.2112 0.0001 1.0000 0.0004 0.0000 0.0000 0.8154 0.0022 0.0002 0.0000
Peaking Uniform, 30: 0.0000 0.0061 0.1548 0.2819 0.0004 1.0000 0.0001 0.0001 0.0000 0.4695 0.5830 0.0002
Peaking Uniform, 60: 0.0000 0.0001 0.0000 0.0241 0.0000 0.0001 1.0000 0.3682 0.0000 0.0001 0.0156 0.7021
Peaking Uniform, 100: 0.0000 0.0000 0.0000 0.0156 0.0000 0.0001 0.3682 1.0000 0.0000 0.0000 0.0022 0.2819
Weighted Uniform, 10: 0.0022 0.2112 0.0364 0.0000 0.8154 0.0000 0.0000 0.0000 1.0000 0.0004 0.0000 0.0000
Weighted Uniform, 30: 0.0000 0.0241 0.3682 0.0156 0.0022 0.4695 0.0001 0.0000 0.0004 1.0000 0.0782 0.0002
Weighted Uniform, 60: 0.0000 0.0037 0.0156 0.9084 0.0002 0.5830 0.0156 0.0022 0.0000 0.0782 1.0000 0.0061
Weighted Uniform, 100: 0.0000 0.0001 0.0000 0.0241 0.0000 0.0002 0.7021 0.2819 0.0000 0.0002 0.0061 1.0000
Saturating Uniform, 10
Saturating Uniform, 30
Saturating Uniform, 60
Saturating Uniform, 100
Peaking Uniform, 10
Peaking Uniform, 30
Peaking Uniform, 60
Peaking Uniform, 100
Weighted Uniform, 10
Weighted Uniform, 30
Weighted Uniform, 60
Weighted Uniform, 100

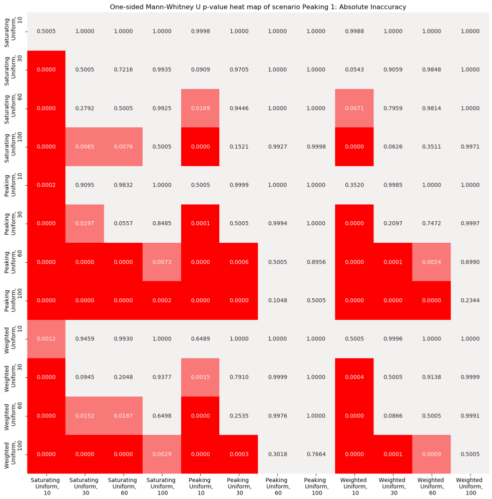
One-sided Mann-Whitney U p-value heat map of scenario Peaking 1: Absolute Inaccuracy
Saturating Uniform, 10: 0.5005 1.0000 1.0000 1.0000 0.9998 1.0000 1.0000 1.0000 0.9988 1.0000 1.0000 1.0000
Saturating Uniform, 30: 0.0000 0.5005 0.7216 0.9935 0.0909 0.9705 1.0000 1.0000 0.0543 0.9059 0.9848 1.0000
Saturating Uniform, 60: 0.0000 0.2792 0.5005 0.9925 0.0169 0.9446 1.0000 1.0000 0.0071 0.7959 0.9814 1.0000
Saturating Uniform, 100: 0.0000 0.0065 0.0076 0.5005 0.0000 0.1521 0.9927 0.9998 0.0000 0.0626 0.3511 0.9971
Peaking Uniform, 10: 0.0002 0.9095 0.9832 1.0000 0.5005 0.9999 1.0000 1.0000 0.3520 0.9985 1.0000 1.0000
Peaking Uniform, 30: 0.0000 0.0297 0.0557 0.8485 0.0001 0.5005 0.9994 1.0000 0.0000 0.2097 0.7472 0.9997
Peaking Uniform, 60: 0.0000 0.0000 0.0000 0.0073 0.0000 0.0006 0.5005 0.8956 0.0000 0.0001 0.0024 0.6990
Peaking Uniform, 100: 0.0000 0.0000 0.0000 0.0002 0.0000 0.0000 0.1048 0.5005 0.0000 0.0000 0.0000 0.2344
Weighted Uniform, 10: 0.0012 0.9459 0.9930 1.0000 0.6489 1.0000 1.0000 1.0000 0.5005 0.9996 1.0000 1.0000
Weighted Uniform, 30: 0.0000 0.0945 0.2048 0.9377 0.0015 0.7910 0.9999 1.0000 0.0004 0.5005 0.9138 0.9999
Weighted Uniform, 60: 0.0000 0.0152 0.0187 0.6498 0.0000 0.2535 0.9976 1.0000 0.0000 0.0866 0.5005 0.9991
Weighted Uniform, 100: 0.0000 0.0000 0.0000 0.0029 0.0000 0.0003 0.3018 0.7664 0.0000 0.0001 0.0009 0.5005
Saturating Uniform, 10
Saturating Uniform, 30
Saturating Uniform, 60
Saturating Uniform, 100
Peaking Uniform, 10
Peaking Uniform, 30
Peaking Uniform, 60
Peaking Uniform, 100
Weighted Uniform, 10
Weighted Uniform, 30
Weighted Uniform, 60
Weighted Uniform, 100

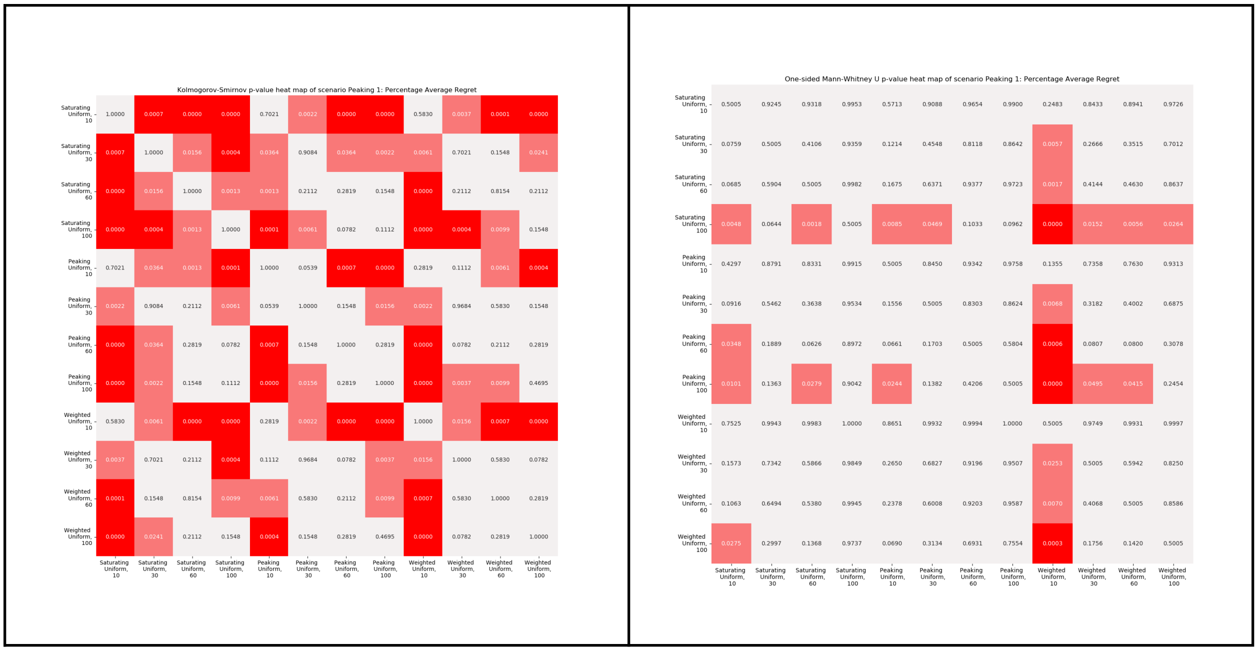

Figure Supplementary.Statistical.Obj1.P1. Kolmogorov–Smirnov (left) and Mann-Whitney U (right) heatmaps of p-values for objective 1, Scenario Peaking 1. These are for the metrics of PSR (top), Absolute Inaccuracy (middle) and PAR (bottom). Cells with a light pink hue represent the test statistic for that comparison would be significant under the threshold p<0.05, cells with a red hue represent the test statistic for that comparison would be significant under the threshold p<0.05 with Bonferri multiple comparison correction.

<u>Scenario Peaking 2</u>

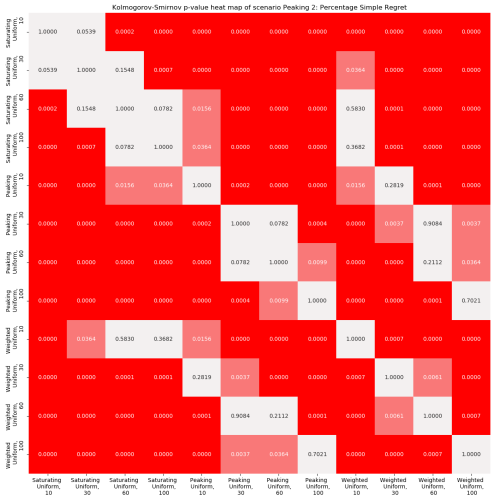
Kolmogorov-Smirnov p-value heat map of scenario Peaking 2: Percentage Simple Regret
Saturating Uniform, 10
Saturating Uniform, 30
Saturating Uniform, 60
Saturating Uniform, 100
Peaking Uniform, 10
Peaking Uniform, 30
Peaking Uniform, 60
Peaking Uniform, 100
Weighted Uniform, 10
Weighted Uniform, 30
Weighted Uniform, 60
Weighted Uniform, 100

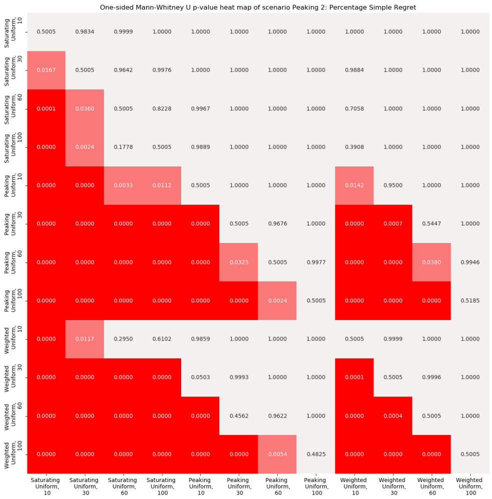
One-sided Mann-Whitney U p-value heat map of scenario Peaking 2: Percentage Simple Regret
Saturating Uniform, 10
Saturating Uniform, 30
Saturating Uniform, 60
Saturating Uniform, 100
Peaking Uniform, 10
Peaking Uniform, 30
Peaking Uniform, 60
Peaking Uniform, 100
Weighted Uniform, 10
Weighted Uniform, 30
Weighted Uniform, 60
Weighted Uniform, 100

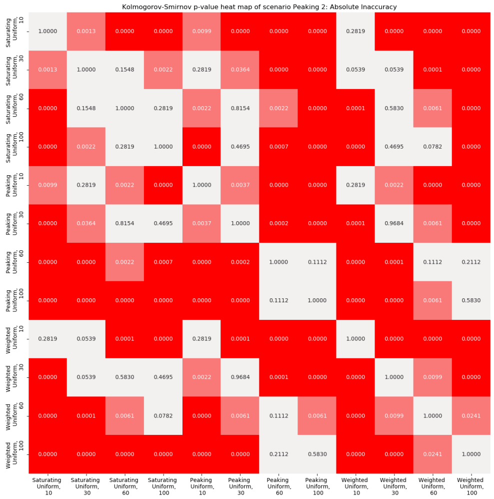
Kolmogorov-Smirnov p-value heat map of scenario Peaking 2: Absolute Inaccuracy
Saturating Uniform, 10
Saturating Uniform, 30
Saturating Uniform, 60
Saturating Uniform, 100
Peaking Uniform, 10
Peaking Uniform, 30
Peaking Uniform, 60
Peaking Uniform, 100
Weighted Uniform, 10
Weighted Uniform, 30
Weighted Uniform, 60
Weighted Uniform, 100

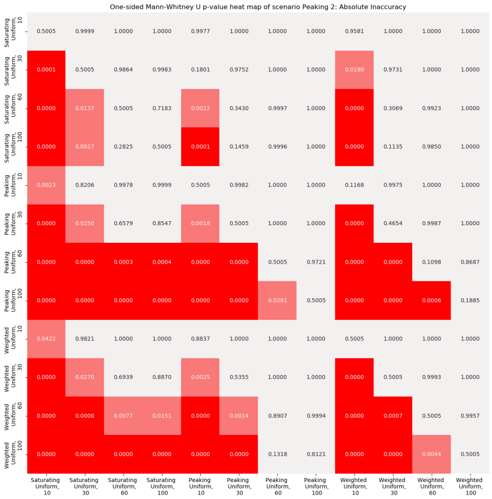
One-sided Mann-Whitney U p-value heat map of scenario Peaking 2: Absolute Inaccuracy
Saturating Uniform, 10
Saturating Uniform, 30
Saturating Uniform, 60
Saturating Uniform, 100
Peaking Uniform, 10
Peaking Uniform, 30
Peaking Uniform, 60
Peaking Uniform, 100
Weighted Uniform, 10
Weighted Uniform, 30
Weighted Uniform, 60
Weighted Uniform, 100

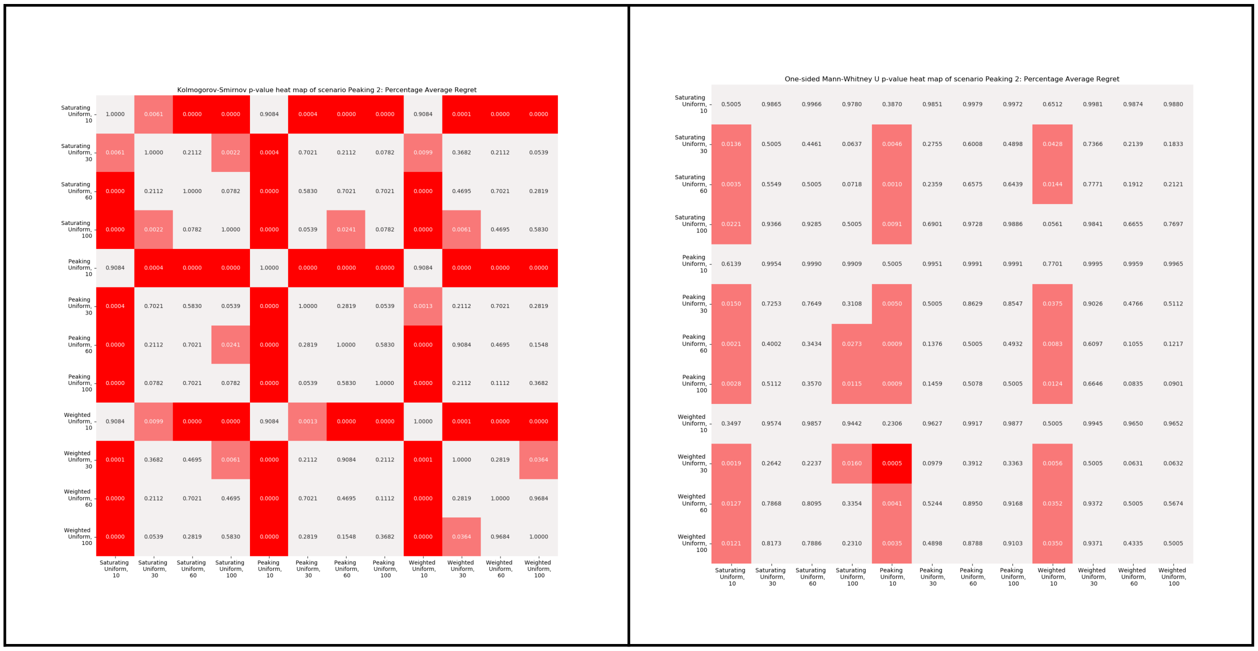

Figure Supplementary.Statistical.Obj1.P2. Kolmogorov–Smirnov (left) and Mann-Whitney U (right) heatmaps of p-values for objective 1, Scenario Peaking 2. These are for the metrics of PSR (top), Absolute Inaccuracy (middle) and PAR (bottom). Cells with a light pink hue represent the test statistic for that comparison would be significant under the threshold p<0.05, cells with a red hue represent the test statistic for that comparison would be significant under the threshold p<0.05 with Bonferri multiple comparison correction.

Scenario Peaking 3

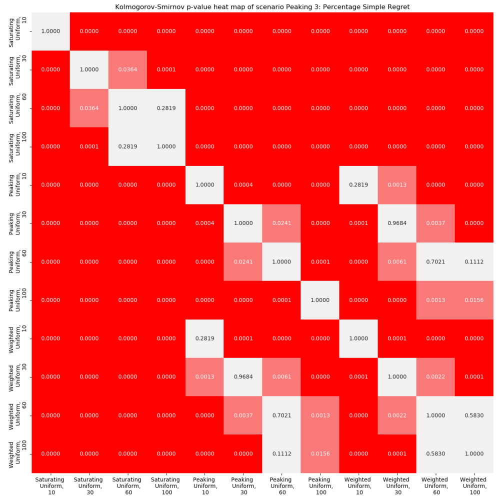
Kolmogorov-Smirnov p-value heat map of scenario Peaking 3: Percentage Simple Regret
Saturating Uniform, 10: 1.0000 0.0000 0.0000 0.0000 0.0000 0.0000 0.0000 0.0000 0.0000 0.0000 0.0000 0.0000
Saturating Uniform, 30: 0.0000 1.0000 0.0364 0.0001 0.0000 0.0000 0.0000 0.0000 0.0000 0.0000 0.0000 0.0000
Saturating Uniform, 60: 0.0000 0.0364 1.0000 0.2819 0.0000 0.0000 0.0000 0.0000 0.0000 0.0000 0.0000 0.0000
Saturating Uniform, 100: 0.0000 0.0001 0.2819 1.0000 0.0000 0.0000 0.0000 0.0000 0.0000 0.0000 0.0000 0.0000
Peaking Uniform, 10: 0.0000 0.0000 0.0000 0.0000 1.0000 0.0004 0.0000 0.0000 0.2819 0.0013 0.0000 0.0000
Peaking Uniform, 30: 0.0000 0.0000 0.0000 0.0000 0.0004 1.0000 0.0241 0.0000 0.0001 0.9684 0.0037 0.0000
Peaking Uniform, 60: 0.0000 0.0000 0.0000 0.0000 0.0000 0.0241 1.0000 0.0001 0.0000 0.0061 0.7021 0.1112
Peaking Uniform, 100: 0.0000 0.0000 0.0000 0.0000 0.0000 0.0000 0.0001 1.0000 0.0000 0.0000 0.0013 0.0156
Weighted Uniform, 10: 0.0000 0.0000 0.0000 0.0000 0.2819 0.0001 0.0000 0.0000 1.0000 0.0001 0.0000 0.0000
Weighted Uniform, 30: 0.0000 0.0000 0.0000 0.0000 0.0013 0.9684 0.0061 0.0000 0.0001 1.0000 0.0022 0.0001
Weighted Uniform, 60: 0.0000 0.0000 0.0000 0.0000 0.0000 0.0037 0.7021 0.0013 0.0000 0.0022 1.0000 0.5830
Weighted Uniform, 100: 0.0000 0.0000 0.0000 0.0000 0.0000 0.0000 0.1112 0.0156 0.0000 0.0001 0.5830 1.0000
Saturating Uniform, 10 | Saturating Uniform, 30 | Saturating Uniform, 60 | Saturating Uniform, 100 | Peaking Uniform, 10 | Peaking Uniform, 30 | Peaking Uniform, 60 | Peaking Uniform, 100 | Weighted Uniform, 10 | Weighted Uniform, 30 | Weighted Uniform, 60 | Weighted Uniform, 100

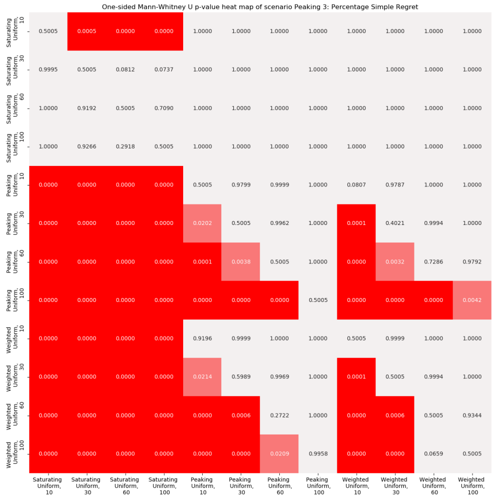
One-sided Mann-Whitney U p-value heat map of scenario Peaking 3: Percentage Simple Regret
0.5005 0.0005 0.0000 0.0000 1.0000 1.0000 1.0000 1.0000 1.0000 1.0000 1.0000 1.0000
0.9995 0.5005 0.0812 0.0737 1.0000 1.0000 1.0000 1.0000 1.0000 1.0000 1.0000 1.0000
1.0000 0.9192 0.5005 0.7090 1.0000 1.0000 1.0000 1.0000 1.0000 1.0000 1.0000 1.0000
1.0000 0.9266 0.2918 0.5005 1.0000 1.0000 1.0000 1.0000 1.0000 1.0000 1.0000 1.0000
0.0000 0.0000 0.0000 0.0000 0.5005 0.9799 0.9999 1.0000 0.0807 0.9787 1.0000 1.0000
0.0000 0.0000 0.0000 0.0000 0.0202 0.5005 0.9962 1.0000 0.0001 0.4021 0.9994 1.0000
0.0000 0.0000 0.0000 0.0000 0.0001 0.0038 0.5005 1.0000 0.0000 0.0032 0.7286 0.9792
0.0000 0.0000 0.0000 0.0000 0.0000 0.0000 0.0000 0.5005 0.0000 0.0000 0.0000 0.0042
0.0000 0.0000 0.0000 0.0000 0.9196 0.9999 1.0000 1.0000 0.5005 0.9999 1.0000 1.0000
0.0000 0.0000 0.0000 0.0000 0.0214 0.5989 0.9969 1.0000 0.0001 0.5005 0.9994 1.0000
0.0000 0.0000 0.0000 0.0000 0.0000 0.0006 0.2722 1.0000 0.0000 0.0006 0.5005 0.9344
0.0000 0.0000 0.0000 0.0000 0.0000 0.0000 0.0209 0.9958 0.0000 0.0000 0.0659 0.5005
Saturating Uniform, 10 | Saturating Uniform, 30 | Saturating Uniform, 60 | Saturating Uniform, 100 | Peaking Uniform, 10 | Peaking Uniform, 30 | Peaking Uniform, 60 | Peaking Uniform, 100 | Weighted Uniform, 10 | Weighted Uniform, 30 | Weighted Uniform, 60 | Weighted Uniform, 100

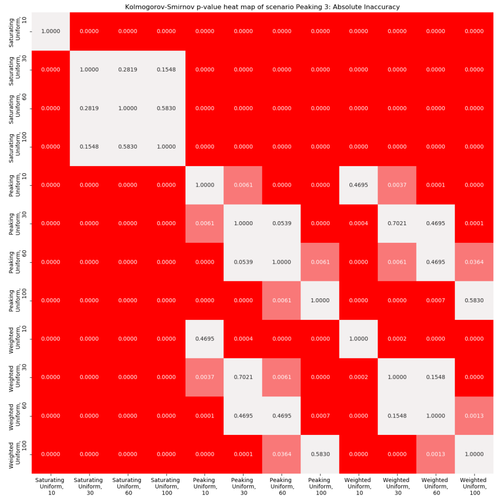
Kolmogorov-Smirnov p-value heat map of scenario Peaking 3: Absolute Inaccuracy
1.0000 0.0000 0.0000 0.0000 0.0000 0.0000 0.0000 0.0000 0.0000 0.0000 0.0000 0.0000
0.0000 1.0000 0.2819 0.1548 0.0000 0.0000 0.0000 0.0000 0.0000 0.0000 0.0000 0.0000
0.0000 0.2819 1.0000 0.5830 0.0000 0.0000 0.0000 0.0000 0.0000 0.0000 0.0000 0.0000
0.0000 0.1548 0.5830 1.0000 0.0000 0.0000 0.0000 0.0000 0.0000 0.0000 0.0000 0.0000
0.0000 0.0000 0.0000 0.0000 1.0000 0.0061 0.0000 0.0000 0.4695 0.0037 0.0001 0.0000
0.0000 0.0000 0.0000 0.0000 0.0061 1.0000 0.0539 0.0000 0.0004 0.7021 0.4695 0.0001
0.0000 0.0000 0.0000 0.0000 0.0000 0.0539 1.0000 0.0061 0.0000 0.0061 0.4695 0.0364
0.0000 0.0000 0.0000 0.0000 0.0000 0.0000 0.0061 1.0000 0.0000 0.0000 0.0007 0.5830
0.0000 0.0000 0.0000 0.0000 0.4695 0.0004 0.0000 0.0000 1.0000 0.0002 0.0000 0.0000
0.0000 0.0000 0.0000 0.0000 0.0037 0.7021 0.0061 0.0000 0.0002 1.0000 0.1548 0.0000
0.0000 0.0000 0.0000 0.0000 0.0001 0.4695 0.4695 0.0007 0.0000 0.1548 1.0000 0.0013
0.0000 0.0000 0.0000 0.0000 0.0000 0.0001 0.0364 0.5830 0.0000 0.0000 0.0013 1.0000
Saturating Uniform, 10 | Saturating Uniform, 30 | Saturating Uniform, 60 | Saturating Uniform, 100 | Peaking Uniform, 10 | Peaking Uniform, 30 | Peaking Uniform, 60 | Peaking Uniform, 100 | Weighted Uniform, 10 | Weighted Uniform, 30 | Weighted Uniform, 60 | Weighted Uniform, 100

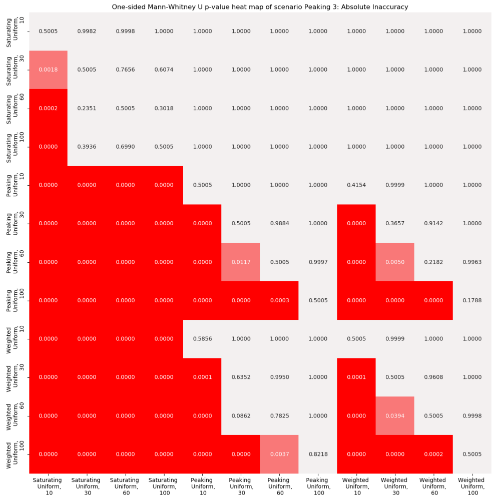
One-sided Mann-Whitney U p-value heat map of scenario Peaking 3: Absolute Inaccuracy
0.5005 0.9982 0.9998 1.0000 1.0000 1.0000 1.0000 1.0000 1.0000 1.0000 1.0000 1.0000
0.0018 0.5005 0.7656 0.6074 1.0000 1.0000 1.0000 1.0000 1.0000 1.0000 1.0000 1.0000
0.0002 0.2351 0.5005 0.3018 1.0000 1.0000 1.0000 1.0000 1.0000 1.0000 1.0000 1.0000
0.0000 0.3936 0.6990 0.5005 1.0000 1.0000 1.0000 1.0000 1.0000 1.0000 1.0000 1.0000
0.0000 0.0000 0.0000 0.0000 0.5005 1.0000 1.0000 1.0000 0.4154 0.9999 1.0000 1.0000
0.0000 0.0000 0.0000 0.0000 0.0000 0.5005 0.9884 1.0000 0.0000 0.3657 0.9142 1.0000
0.0000 0.0000 0.0000 0.0000 0.0000 0.0117 0.5005 0.9997 0.0000 0.0050 0.2182 0.9963
0.0000 0.0000 0.0000 0.0000 0.0000 0.0000 0.0003 0.5005 0.0000 0.0000 0.0000 0.1788
0.0000 0.0000 0.0000 0.0000 0.5856 1.0000 1.0000 1.0000 0.5005 0.9999 1.0000 1.0000
0.0000 0.0000 0.0000 0.0000 0.0001 0.6352 0.9950 1.0000 0.0001 0.5005 0.9608 1.0000
0.0000 0.0000 0.0000 0.0000 0.0000 0.0862 0.7825 1.0000 0.0000 0.0394 0.5005 0.9998
0.0000 0.0000 0.0000 0.0000 0.0000 0.0000 0.0037 0.8218 0.0000 0.0000 0.0002 0.5005
Saturating Uniform, 10 | Saturating Uniform, 30 | Saturating Uniform, 60 | Saturating Uniform, 100 | Peaking Uniform, 10 | Peaking Uniform, 30 | Peaking Uniform, 60 | Peaking Uniform, 100 | Weighted Uniform, 10 | Weighted Uniform, 30 | Weighted Uniform, 60 | Weighted Uniform, 100

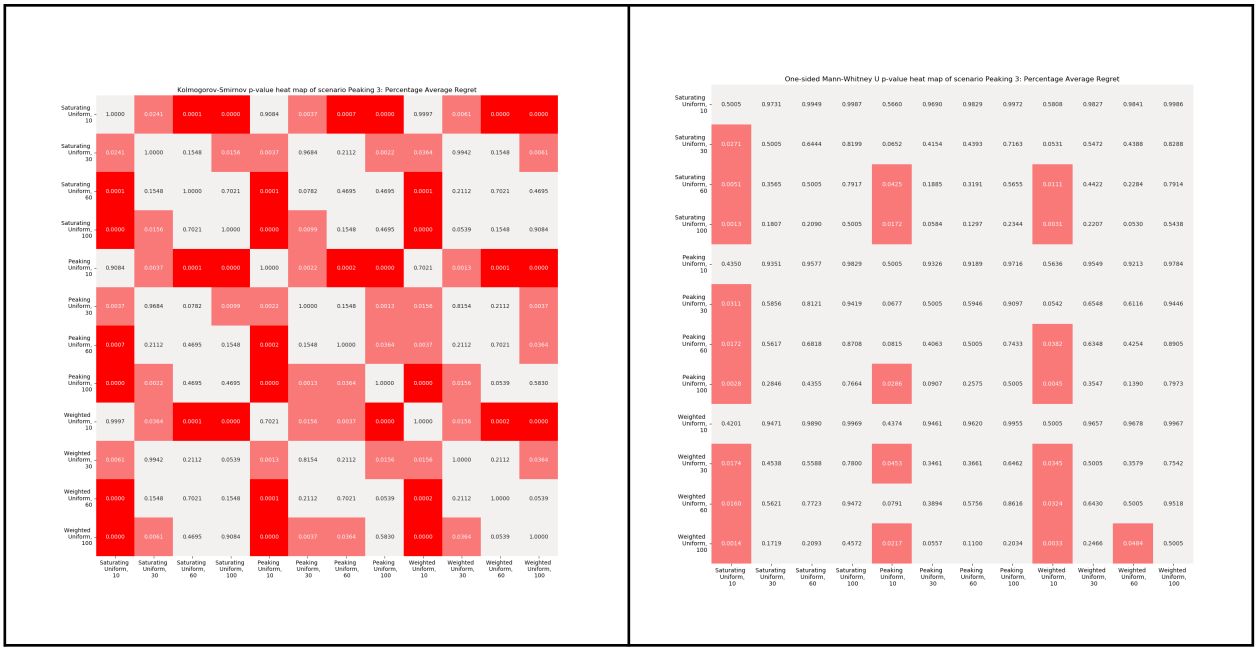

Figure Supplementary.Statistical.Obj1.P3. Kolmogorov–Smirnov (left) and Mann-Whitney U (right) heatmaps of p-values for objective 1, Scenario Peaking 3. These are for the metrics of PSR (top), Absolute Inaccuracy (middle) and PAR (bottom). Cells with a light pink hue represent the test statistic for that comparison would be significant under the threshold p<0.05, cells with a red hue represent the test statistic for that comparison would be significant under the threshold p<0.05 with Bonferri multiple comparison correction.

Scenario Peaking 4

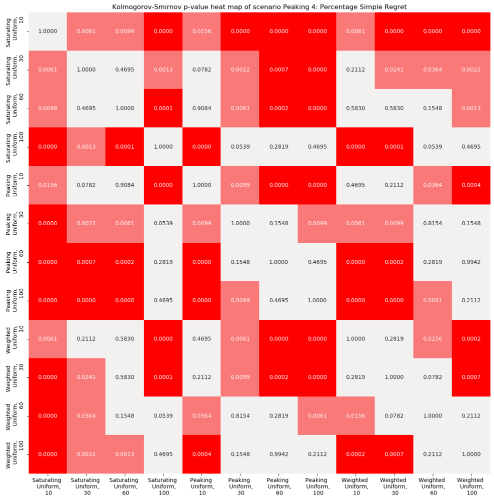
Kolmogorov-Smirnov p-value heat map of scenario Peaking 4: Percentage Simple Regret
Saturating Uniform, 10
Saturating Uniform, 30
Saturating Uniform, 60
Saturating Uniform, 100
Peaking Uniform, 10
Peaking Uniform, 30
Peaking Uniform, 60
Peaking Uniform, 100
Weighted Uniform, 10
Weighted Uniform, 30
Weighted Uniform, 60
Weighted Uniform, 100

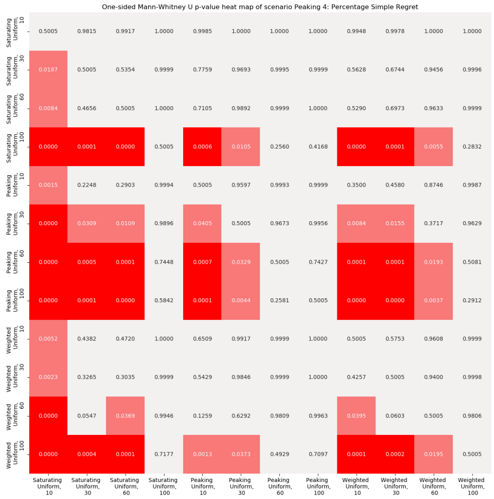
One-sided Mann-Whitney U p-value heat map of scenario Peaking 4: Percentage Simple Regret
Saturating Uniform, 10
Saturating Uniform, 30
Saturating Uniform, 60
Saturating Uniform, 100
Peaking Uniform, 10
Peaking Uniform, 30
Peaking Uniform, 60
Peaking Uniform, 100
Weighted Uniform, 10
Weighted Uniform, 30
Weighted Uniform, 60
Weighted Uniform, 100

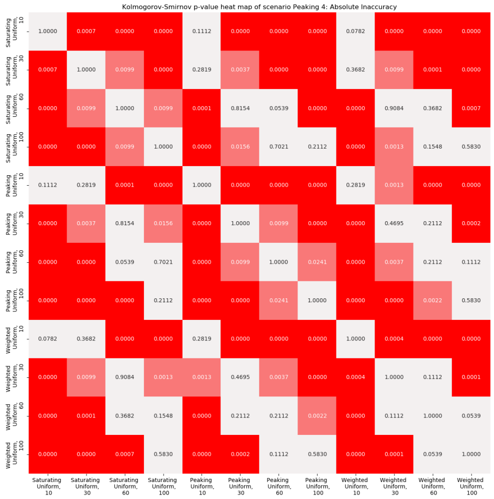
Kolmogorov-Smirnov p-value heat map of scenario Peaking 4: Absolute Inaccuracy
Saturating Uniform, 10
Saturating Uniform, 30
Saturating Uniform, 60
Saturating Uniform, 100
Peaking Uniform, 10
Peaking Uniform, 30
Peaking Uniform, 60
Peaking Uniform, 100
Weighted Uniform, 10
Weighted Uniform, 30
Weighted Uniform, 60
Weighted Uniform, 100

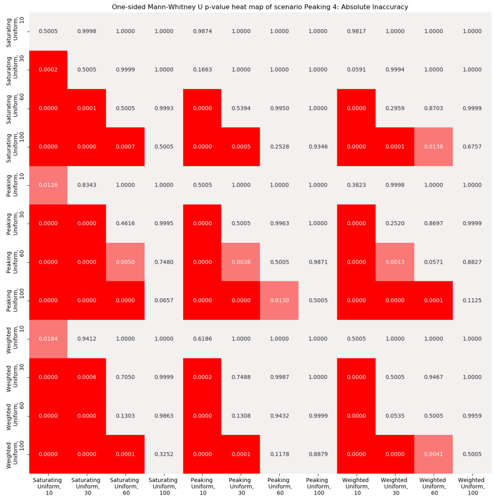
One-sided Mann-Whitney U p-value heat map of scenario Peaking 4: Absolute Inaccuracy
Saturating Uniform, 10
Saturating Uniform, 30
Saturating Uniform, 60
Saturating Uniform, 100
Peaking Uniform, 10
Peaking Uniform, 30
Peaking Uniform, 60
Peaking Uniform, 100
Weighted Uniform, 10
Weighted Uniform, 30
Weighted Uniform, 60
Weighted Uniform, 100

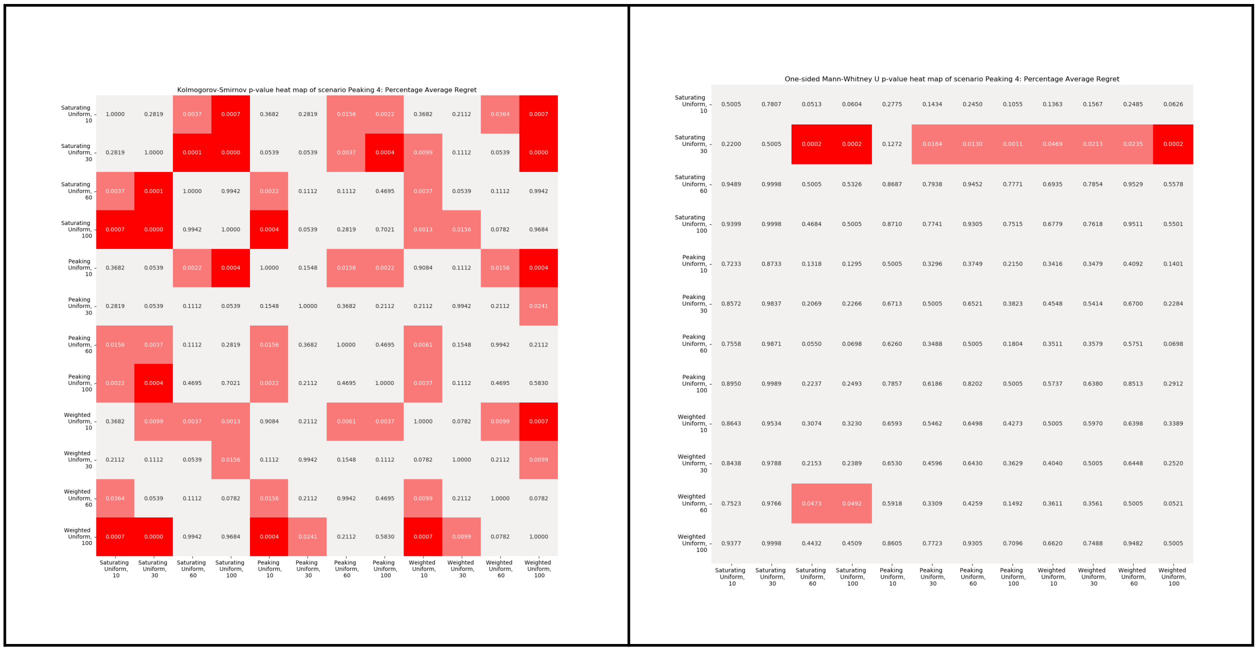

Figure Supplementary.Statistical.Obj1.P4. Kolmogorov–Smirnov (left) and Mann-Whitney U (right) heatmaps of p-values for objective 1, Scenario Peaking 4. These are for the metrics of PSR (top), Absolute Inaccuracy (middle) and PAR (bottom). Cells with a light pink hue represent the test statistic for that comparison would be significant under the threshold p<0.05, cells with a red hue represent the test statistic for that comparison would be significant under the threshold p<0.05 with Bonferri multiple comparison correction.

Scenario Peaking 5

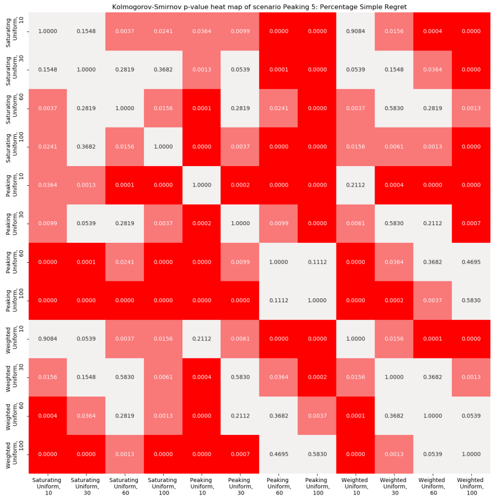
Kolmogorov-Smirnov p-value heat map of scenario Peaking 5: Percentage Simple Regret
Saturating Uniform, 10
Saturating Uniform, 30
Saturating Uniform, 60
Saturating Uniform, 100
Peaking Uniform, 10
Peaking Uniform, 30
Peaking Uniform, 60
Peaking Uniform, 100
Weighted Uniform, 10
Weighted Uniform, 30
Weighted Uniform, 60
Weighted Uniform, 100

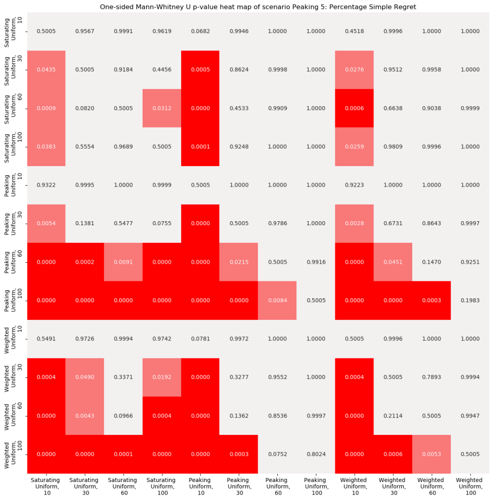
One-sided Mann-Whitney U p-value heat map of scenario Peaking 5: Percentage Simple Regret
Saturating Uniform, 10
Saturating Uniform, 30
Saturating Uniform, 60
Saturating Uniform, 100
Peaking Uniform, 10
Peaking Uniform, 30
Peaking Uniform, 60
Peaking Uniform, 100
Weighted Uniform, 10
Weighted Uniform, 30
Weighted Uniform, 60
Weighted Uniform, 100

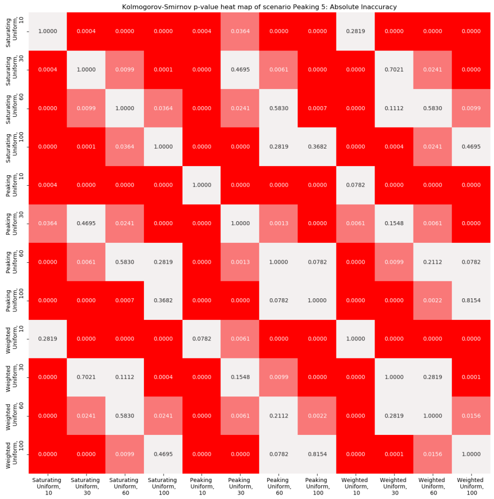
Kolmogorov-Smirnov p-value heat map of scenario Peaking 5: Absolute Inaccuracy
Saturating Uniform, 10
Saturating Uniform, 30
Saturating Uniform, 60
Saturating Uniform, 100
Peaking Uniform, 10
Peaking Uniform, 30
Peaking Uniform, 60
Peaking Uniform, 100
Weighted Uniform, 10
Weighted Uniform, 30
Weighted Uniform, 60
Weighted Uniform, 100

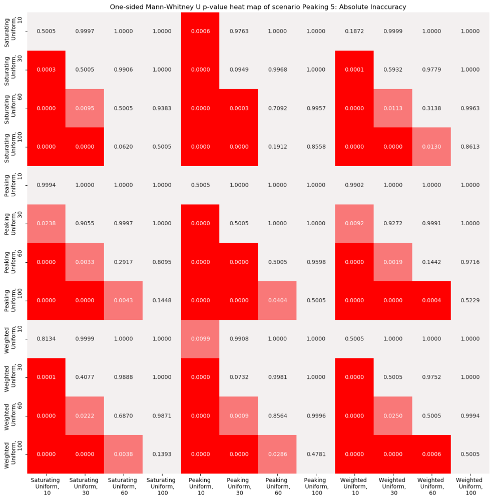
One-sided Mann-Whitney U p-value heat map of scenario Peaking 5: Absolute Inaccuracy
Saturating Uniform, 10
Saturating Uniform, 30
Saturating Uniform, 60
Saturating Uniform, 100
Peaking Uniform, 10
Peaking Uniform, 30
Peaking Uniform, 60
Peaking Uniform, 100
Weighted Uniform, 10
Weighted Uniform, 30
Weighted Uniform, 60
Weighted Uniform, 100

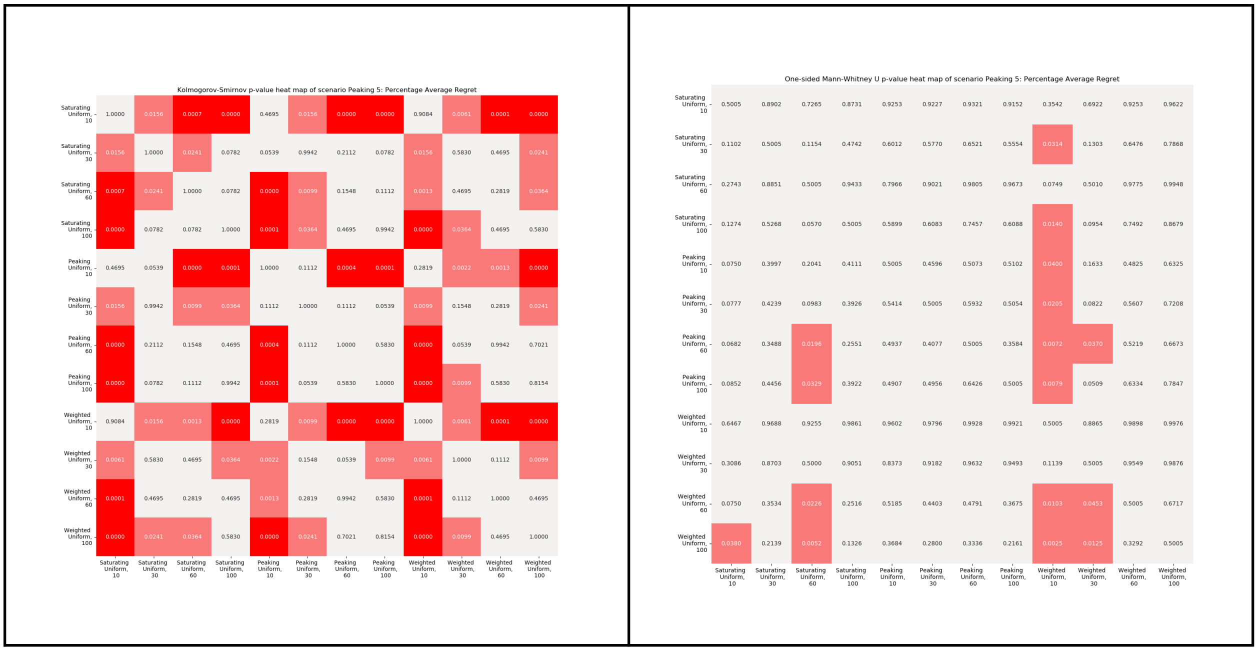

Figure Supplementary.Statistical.Obj1.P5. Kolmogorov–Smirnov (left) and Mann-Whitney U (right) heatmaps of p-values for objective 1, Scenario Peaking 5. These are for the metrics of PSR (top), Absolute Inaccuracy (middle) and PAR (bottom). Cells with a light pink hue represent the test statistic for that comparison would be significant under the threshold p<0.05, cells with a red hue represent the test statistic for that comparison would be significant under the threshold p<0.05 with Bonferri multiple comparison correction.

Scenario Other 1

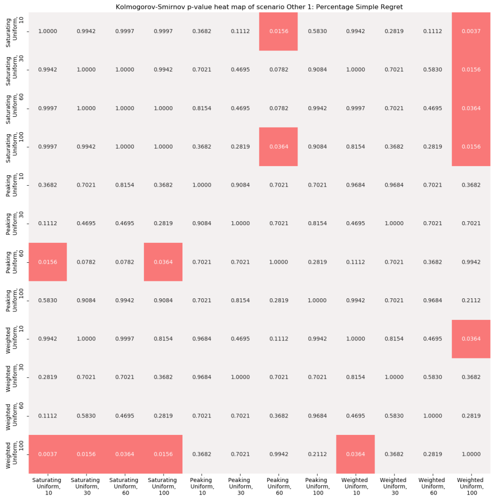
Kolmogorov-Smirnov p-value heat map of scenario Other 1: Percentage Simple Regret
Saturating Uniform, 10
Saturating Uniform, 30
Saturating Uniform, 60
Saturating Uniform, 100
Peaking Uniform, 10
Peaking Uniform, 30
Peaking Uniform, 60
Peaking Uniform, 100
Weighted Uniform, 10
Weighted Uniform, 30
Weighted Uniform, 60
Weighted Uniform, 100
1.0000 0.9942 0.9997 0.9997 0.3682 0.1112 0.0156 0.5830 0.9942 0.2819 0.1112 0.0037
0.9942 1.0000 1.0000 0.9942 0.7021 0.4695 0.0782 0.9084 1.0000 0.7021 0.5830 0.0156
0.9997 1.0000 1.0000 1.0000 0.8154 0.4695 0.0782 0.9942 0.9997 0.7021 0.4695 0.0364
0.9997 0.9942 1.0000 1.0000 0.3682 0.2819 0.0364 0.9084 0.8154 0.3682 0.2819 0.0156
0.3682 0.7021 0.8154 0.3682 1.0000 0.9084 0.7021 0.7021 0.9684 0.9684 0.7021 0.3682
0.1112 0.4695 0.4695 0.2819 0.9084 1.0000 0.7021 0.8154 0.4695 1.0000 0.7021 0.7021
0.0156 0.0782 0.0782 0.0364 0.7021 0.7021 1.0000 0.2819 0.1112 0.7021 0.3682 0.9942
0.5830 0.9084 0.9942 0.9084 0.7021 0.8154 0.2819 1.0000 0.9942 0.7021 0.9684 0.2112
0.9942 1.0000 0.9997 0.8154 0.9684 0.4695 0.1112 0.9942 1.0000 0.8154 0.4695 0.0364
0.2819 0.7021 0.7021 0.3682 0.9684 1.0000 0.7021 0.7021 0.8154 1.0000 0.5830 0.3682
0.1112 0.5830 0.4695 0.2819 0.7021 0.7021 0.3682 0.9684 0.4695 0.5830 1.0000 0.2819
0.0037 0.0156 0.0364 0.0156 0.3682 0.7021 0.9942 0.2112 0.0364 0.3682 0.2819 1.0000
Saturating Uniform, 10
Saturating Uniform, 30
Saturating Uniform, 60
Saturating Uniform, 100
Peaking Uniform, 10
Peaking Uniform, 30
Peaking Uniform, 60
Peaking Uniform, 100
Weighted Uniform, 10
Weighted Uniform, 30
Weighted Uniform, 60
Weighted Uniform, 100

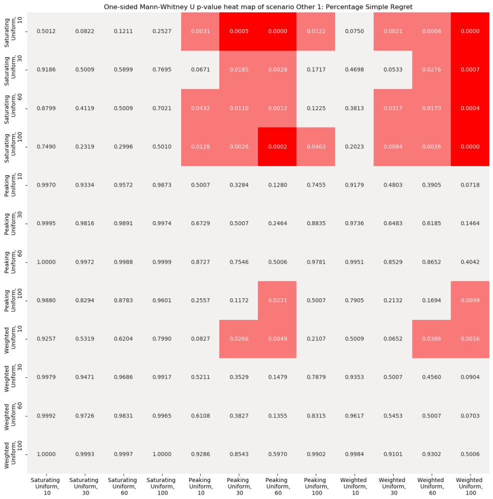
One-sided Mann-Whitney U p-value heat map of scenario Other 1: Percentage Simple Regret
Saturating Uniform, 10
Saturating Uniform, 30
Saturating Uniform, 60
Saturating Uniform, 100
Peaking Uniform, 10
Peaking Uniform, 30
Peaking Uniform, 60
Peaking Uniform, 100
Weighted Uniform, 10
Weighted Uniform, 30
Weighted Uniform, 60
Weighted Uniform, 100
0.5012 0.0822 0.1211 0.2527 0.0031 0.0005 0.0000 0.0122 0.0750 0.0021 0.0008 0.0000
0.9186 0.5009 0.5899 0.7695 0.0671 0.0185 0.0028 0.1717 0.4698 0.0533 0.0276 0.0007
0.8799 0.4119 0.5009 0.7021 0.0432 0.0110 0.0012 0.1225 0.3813 0.0317 0.0170 0.0004
0.7490 0.2319 0.2996 0.5010 0.0128 0.0026 0.0002 0.0403 0.2023 0.0084 0.0036 0.0000
0.9970 0.9334 0.9572 0.9873 0.5007 0.3284 0.1280 0.7455 0.9179 0.4803 0.3905 0.0718
0.9995 0.9816 0.9891 0.9974 0.6729 0.5007 0.2464 0.8835 0.9736 0.6483 0.6185 0.1464
1.0000 0.9972 0.9988 0.9999 0.8727 0.7546 0.5006 0.9781 0.9951 0.8529 0.8652 0.4042
0.9880 0.8294 0.8783 0.9601 0.2557 0.1172 0.0221 0.5007 0.7905 0.2132 0.1694 0.0099
0.9257 0.5319 0.6204 0.7990 0.0827 0.0266 0.0049 0.2107 0.5009 0.0652 0.0386 0.0016
0.9979 0.9471 0.9686 0.9917 0.5211 0.3529 0.1479 0.7879 0.9353 0.5007 0.4560 0.0904
0.9992 0.9726 0.9831 0.9965 0.6108 0.3827 0.1355 0.8315 0.9617 0.5453 0.5007 0.0703
1.0000 0.9993 0.9997 1.0000 0.9286 0.8543 0.5970 0.9902 0.9984 0.9101 0.9302 0.5006
Saturating Uniform, 10
Saturating Uniform, 30
Saturating Uniform, 60
Saturating Uniform, 100
Peaking Uniform, 10
Peaking Uniform, 30
Peaking Uniform, 60
Peaking Uniform, 100
Weighted Uniform, 10
Weighted Uniform, 30
Weighted Uniform, 60
Weighted Uniform, 100

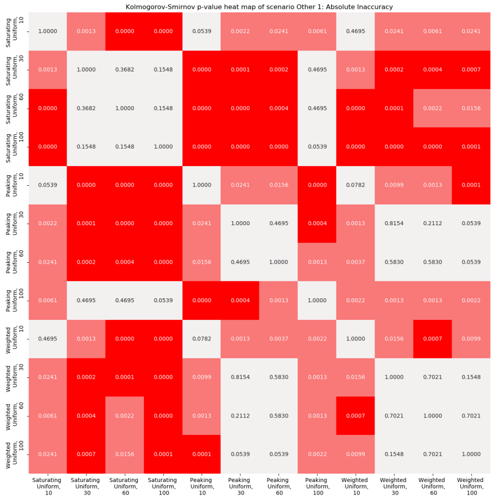
Kolmogorov-Smirnov p-value heat map of scenario Other 1: Absolute Inaccuracy
Saturating Uniform, 10
Saturating Uniform, 30
Saturating Uniform, 60
Saturating Uniform, 100
Peaking Uniform, 10
Peaking Uniform, 30
Peaking Uniform, 60
Peaking Uniform, 100
Weighted Uniform, 10
Weighted Uniform, 30
Weighted Uniform, 60
Weighted Uniform, 100
1.0000 0.0013 0.0000 0.0000 0.0539 0.0022 0.0241 0.0061 0.4695 0.0241 0.0061 0.0241
0.0013 1.0000 0.3682 0.1548 0.0000 0.0001 0.0002 0.4695 0.0013 0.0002 0.0004 0.0007
0.0000 0.3682 1.0000 0.1548 0.0000 0.0000 0.0004 0.4695 0.0000 0.0001 0.0022 0.0156
0.0000 0.1548 0.1548 1.0000 0.0000 0.0000 0.0000 0.0539 0.0000 0.0000 0.0000 0.0001
0.0539 0.0000 0.0000 0.0000 1.0000 0.0241 0.0156 0.0000 0.0782 0.0099 0.0013 0.0001
0.0022 0.0001 0.0000 0.0000 0.0241 1.0000 0.4695 0.0004 0.0013 0.8154 0.2112 0.0539
0.0241 0.0002 0.0004 0.0000 0.0156 0.4695 1.0000 0.0013 0.0037 0.5830 0.5830 0.0539
0.0061 0.4695 0.4695 0.0539 0.0000 0.0004 0.0013 1.0000 0.0022 0.0013 0.0013 0.0022
0.4695 0.0013 0.0000 0.0000 0.0782 0.0013 0.0037 0.0022 1.0000 0.0156 0.0007 0.0099
0.0241 0.0002 0.0001 0.0000 0.0099 0.8154 0.5830 0.0013 0.0156 1.0000 0.7021 0.1548
0.0061 0.0004 0.0022 0.0000 0.0013 0.2112 0.5830 0.0013 0.0007 0.7021 1.0000 0.7021
0.0241 0.0007 0.0156 0.0001 0.0001 0.0539 0.0539 0.0022 0.0099 0.1548 0.7021 1.0000
Saturating Uniform, 10
Saturating Uniform, 30
Saturating Uniform, 60
Saturating Uniform, 100
Peaking Uniform, 10
Peaking Uniform, 30
Peaking Uniform, 60
Peaking Uniform, 100
Weighted Uniform, 10
Weighted Uniform, 30
Weighted Uniform, 60
Weighted Uniform, 100

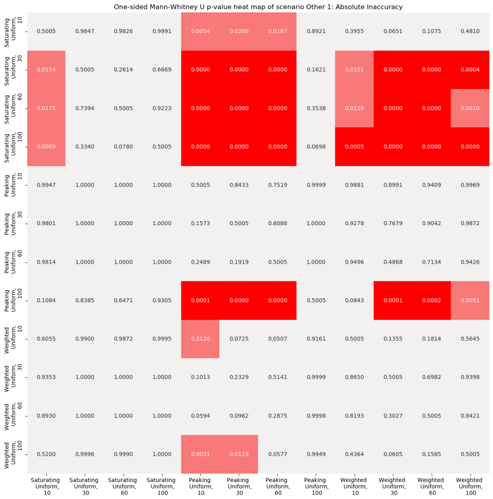
One-sided Mann-Whitney U p-value heat map of scenario Other 1: Absolute Inaccuracy
Saturating Uniform, 10
Saturating Uniform, 30
Saturating Uniform, 60
Saturating Uniform, 100
Peaking Uniform, 10
Peaking Uniform, 30
Peaking Uniform, 60
Peaking Uniform, 100
Weighted Uniform, 10
Weighted Uniform, 30
Weighted Uniform, 60
Weighted Uniform, 100
0.5005 0.9847 0.9826 0.9991 0.0054 0.0200 0.0187 0.8921 0.3955 0.0651 0.1075 0.4810
0.0154 0.5005 0.2614 0.6669 0.0000 0.0000 0.0000 0.1621 0.0101 0.0000 0.0000 0.0004
0.0175 0.7394 0.5005 0.9223 0.0000 0.0000 0.0000 0.3538 0.0129 0.0000 0.0000 0.0010
0.0009 0.3340 0.0780 0.5005 0.0000 0.0000 0.0000 0.0698 0.0005 0.0000 0.0000 0.0000
0.9947 1.0000 1.0000 1.0000 0.5005 0.8433 0.7519 0.9999 0.9881 0.8991 0.9409 0.9969
0.9801 1.0000 1.0000 1.0000 0.1573 0.5005 0.8088 1.0000 0.9278 0.7679 0.9042 0.9872
0.9814 1.0000 1.0000 1.0000 0.2489 0.1919 0.5005 1.0000 0.9496 0.4868 0.7134 0.9426
0.1084 0.8385 0.6471 0.9305 0.0001 0.0000 0.0000 0.5005 0.0843 0.0001 0.0002 0.0051
0.6055 0.9900 0.9872 0.9995 0.0120 0.0725 0.0507 0.9161 0.5005 0.1355 0.1814 0.5645
0.9353 1.0000 1.0000 1.0000 0.1013 0.2329 0.5141 0.9999 0.8650 0.5005 0.6982 0.9398
0.8930 1.0000 1.0000 1.0000 0.0594 0.0962 0.2875 0.9998 0.8193 0.3027 0.5005 0.8421
0.5200 0.9996 0.9990 1.0000 0.0031 0.0129 0.0577 0.9949 0.4364 0.0605 0.1585 0.5005
Saturating Uniform, 10
Saturating Uniform, 30
Saturating Uniform, 60
Saturating Uniform, 100
Peaking Uniform, 10
Peaking Uniform, 30
Peaking Uniform, 60
Peaking Uniform, 100
Weighted Uniform, 10
Weighted Uniform, 30
Weighted Uniform, 60
Weighted Uniform, 100

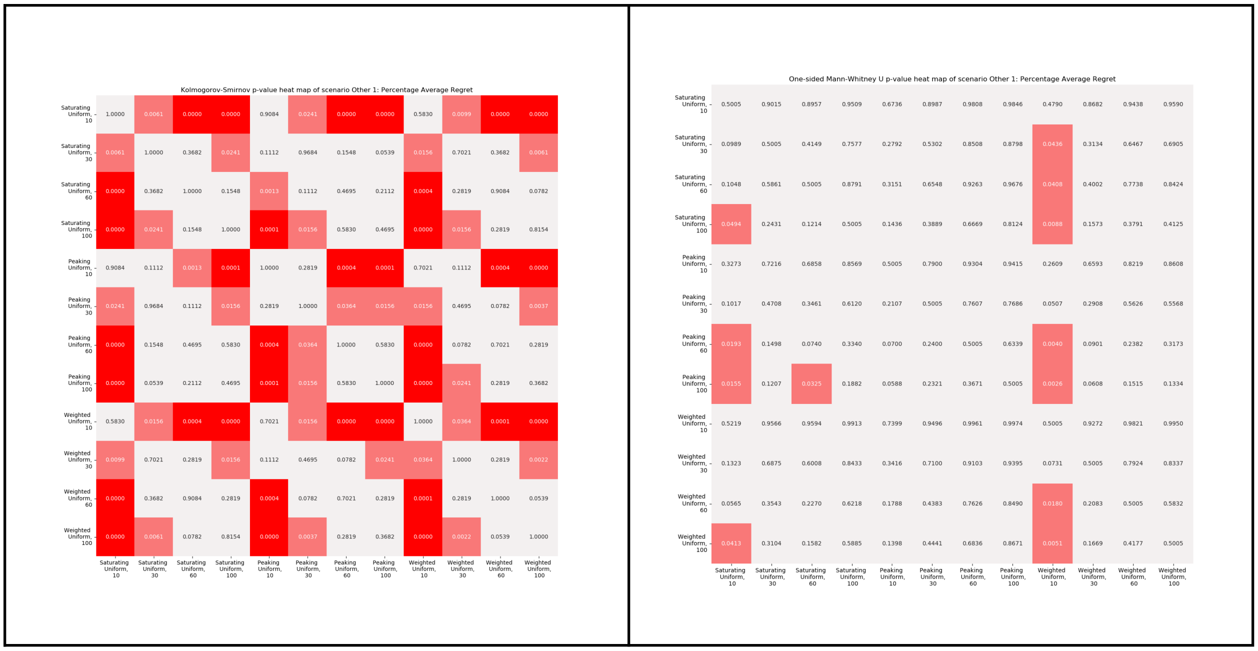

Figure Supplementary.Statistical.Obj1.X1. Kolmogorov–Smirnov (left) and Mann-Whitney U (right) heatmaps of p-values for objective 1, Scenario Other 1. These are for the metrics of PSR (top), Absolute Inaccuracy (middle) and PAR (bottom). Cells with a light pink hue represent the test statistic for that comparison would be significant under the threshold p<0.05, cells with a red hue represent the test statistic for that comparison would be significant under the threshold p<0.05 with Bonferri multiple comparison correction.

Scenario Other 2

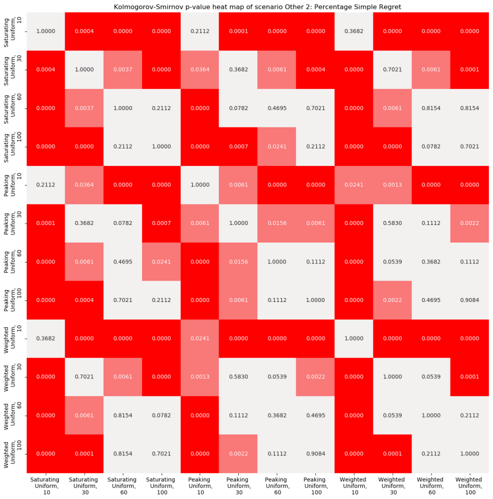
Kolmogorov-Smirnov p-value heat map of scenario Other 2: Percentage Simple Regret
Saturating Uniform, 10
Saturating Uniform, 30
Saturating Uniform, 60
Saturating Uniform, 100
Peaking Uniform, 10
Peaking Uniform, 30
Peaking Uniform, 60
Peaking Uniform, 100
Weighted Uniform, 10
Weighted Uniform, 30
Weighted Uniform, 60
Weighted Uniform, 100
1.0000 0.0004 0.0000 0.0000 0.2112 0.0001 0.0000 0.0000 0.3682 0.0000 0.0000 0.0000
0.0004 1.0000 0.0037 0.0000 0.0364 0.3682 0.0061 0.0004 0.0000 0.7021 0.0061 0.0001
0.0000 0.0037 1.0000 0.2112 0.0000 0.0782 0.4695 0.7021 0.0000 0.0061 0.8154 0.8154
0.0000 0.0000 0.2112 1.0000 0.0000 0.0007 0.0241 0.2112 0.0000 0.0000 0.0782 0.7021
0.2112 0.0364 0.0000 0.0000 1.0000 0.0061 0.0000 0.0000 0.0241 0.0013 0.0000 0.0000
0.0001 0.3682 0.0782 0.0007 0.0061 1.0000 0.0156 0.0061 0.0000 0.5830 0.1112 0.0022
0.0000 0.0061 0.4695 0.0241 0.0000 0.0156 1.0000 0.1112 0.0000 0.0539 0.3682 0.1112
0.0000 0.0004 0.7021 0.2112 0.0000 0.0061 0.1112 1.0000 0.0000 0.0022 0.4695 0.9084
0.3682 0.0000 0.0000 0.0000 0.0241 0.0000 0.0000 0.0000 1.0000 0.0000 0.0000 0.0000
0.0000 0.7021 0.0061 0.0000 0.0013 0.5830 0.0539 0.0022 0.0000 1.0000 0.0539 0.0001
0.0000 0.0061 0.8154 0.0782 0.0000 0.1112 0.3682 0.4695 0.0000 0.0539 1.0000 0.2112
0.0000 0.0001 0.8154 0.7021 0.0000 0.0022 0.1112 0.9084 0.0000 0.0001 0.2112 1.0000

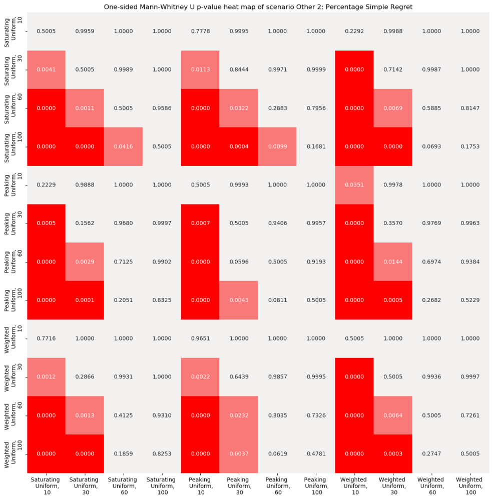
One-sided Mann-Whitney U p-value heat map of scenario Other 2: Percentage Simple Regret
Saturating Uniform, 10
Saturating Uniform, 30
Saturating Uniform, 60
Saturating Uniform, 100
Peaking Uniform, 10
Peaking Uniform, 30
Peaking Uniform, 60
Peaking Uniform, 100
Weighted Uniform, 10
Weighted Uniform, 30
Weighted Uniform, 60
Weighted Uniform, 100
0.5005 0.9959 1.0000 1.0000 0.7778 0.9995 1.0000 1.0000 0.2292 0.9988 1.0000 1.0000
0.0041 0.5005 0.9989 1.0000 0.0113 0.8444 0.9971 0.9999 0.0000 0.7142 0.9987 1.0000
0.0000 0.0011 0.5005 0.9586 0.0000 0.0322 0.2883 0.7956 0.0000 0.0069 0.5885 0.8147
0.0000 0.0000 0.0416 0.5005 0.0000 0.0004 0.0099 0.1681 0.0000 0.0000 0.0693 0.1753
0.2229 0.9888 1.0000 1.0000 0.5005 0.9993 1.0000 1.0000 0.0351 0.9978 1.0000 1.0000
0.0005 0.1562 0.9680 0.9997 0.0007 0.5005 0.9406 0.9957 0.0000 0.3570 0.9769 0.9963
0.0000 0.0029 0.7125 0.9902 0.0000 0.0596 0.5005 0.9193 0.0000 0.0144 0.6974 0.9384
0.0000 0.0001 0.2051 0.8325 0.0000 0.0043 0.0811 0.5005 0.0000 0.0005 0.2682 0.5229
0.7716 1.0000 1.0000 1.0000 0.9651 1.0000 1.0000 1.0000 0.5005 1.0000 1.0000 1.0000
0.0012 0.2866 0.9931 1.0000 0.0022 0.6439 0.9857 0.9995 0.0000 0.5005 0.9936 0.9997
0.0000 0.0013 0.4125 0.9310 0.0000 0.0232 0.3035 0.7326 0.0000 0.0064 0.5005 0.7261
0.0000 0.0000 0.1859 0.8253 0.0000 0.0037 0.0619 0.4781 0.0000 0.0003 0.2747 0.5005

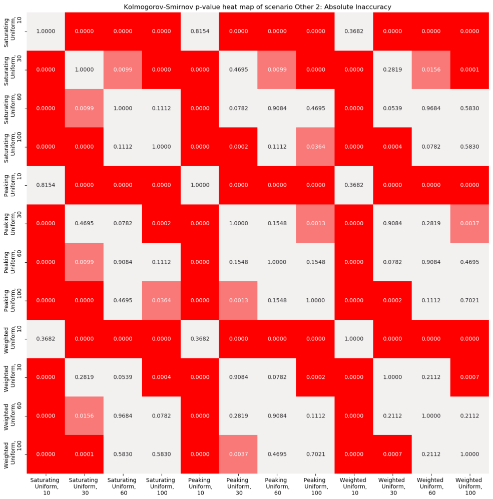
Kolmogorov-Smirnov p-value heat map of scenario Other 2: Absolute Inaccuracy
Saturating Uniform, 10
Saturating Uniform, 30
Saturating Uniform, 60
Saturating Uniform, 100
Peaking Uniform, 10
Peaking Uniform, 30
Peaking Uniform, 60
Peaking Uniform, 100
Weighted Uniform, 10
Weighted Uniform, 30
Weighted Uniform, 60
Weighted Uniform, 100
1.0000 0.0000 0.0000 0.0000 0.8154 0.0000 0.0000 0.0000 0.3682 0.0000 0.0000 0.0000
0.0000 1.0000 0.0099 0.0000 0.0000 0.4695 0.0099 0.0000 0.0000 0.2819 0.0156 0.0001
0.0000 0.0099 1.0000 0.1112 0.0000 0.0782 0.9084 0.4695 0.0000 0.0539 0.9684 0.5830
0.0000 0.0000 0.1112 1.0000 0.0000 0.0002 0.1112 0.0364 0.0000 0.0004 0.0782 0.5830
0.8154 0.0000 0.0000 0.0000 1.0000 0.0000 0.0000 0.0000 0.3682 0.0000 0.0000 0.0000
0.0000 0.4695 0.0782 0.0002 0.0000 1.0000 0.1548 0.0013 0.0000 0.9084 0.2819 0.0037
0.0000 0.0099 0.9084 0.1112 0.0000 0.1548 1.0000 0.1548 0.0000 0.0782 0.9084 0.4695
0.0000 0.0000 0.4695 0.0364 0.0000 0.0013 0.1548 1.0000 0.0000 0.0002 0.1112 0.7021
0.3682 0.0000 0.0000 0.0000 0.3682 0.0000 0.0000 0.0000 1.0000 0.0000 0.0000 0.0000
0.0000 0.2819 0.0539 0.0004 0.0000 0.9084 0.0782 0.0002 0.0000 1.0000 0.2112 0.0007
0.0000 0.0156 0.9684 0.0782 0.0000 0.2819 0.9084 0.1112 0.0000 0.2112 1.0000 0.2112
0.0000 0.0001 0.5830 0.5830 0.0000 0.0037 0.4695 0.7021 0.0000 0.0007 0.2112 1.0000

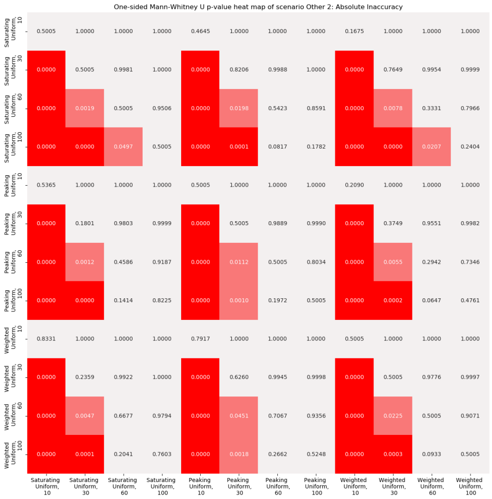
One-sided Mann-Whitney U p-value heat map of scenario Other 2: Absolute Inaccuracy
Saturating Uniform, 10
Saturating Uniform, 30
Saturating Uniform, 60
Saturating Uniform, 100
Peaking Uniform, 10
Peaking Uniform, 30
Peaking Uniform, 60
Peaking Uniform, 100
Weighted Uniform, 10
Weighted Uniform, 30
Weighted Uniform, 60
Weighted Uniform, 100
0.5005 1.0000 1.0000 1.0000 0.4645 1.0000 1.0000 1.0000 0.1675 1.0000 1.0000 1.0000
0.0000 0.5005 0.9981 1.0000 0.0000 0.8206 0.9988 1.0000 0.0000 0.7649 0.9954 0.9999
0.0000 0.0019 0.5005 0.9506 0.0000 0.0198 0.5423 0.8591 0.0000 0.0078 0.3331 0.7966
0.0000 0.0000 0.0497 0.5005 0.0000 0.0001 0.0817 0.1782 0.0000 0.0000 0.0207 0.2404
0.5365 1.0000 1.0000 1.0000 0.5005 1.0000 1.0000 1.0000 0.2090 1.0000 1.0000 1.0000
0.0000 0.1801 0.9803 0.9999 0.0000 0.5005 0.9889 0.9990 0.0000 0.3749 0.9551 0.9982
0.0000 0.0012 0.4586 0.9187 0.0000 0.0112 0.5005 0.8034 0.0000 0.0055 0.2942 0.7346
0.0000 0.0000 0.1414 0.8225 0.0000 0.0010 0.1972 0.5005 0.0000 0.0002 0.0647 0.4761
0.8331 1.0000 1.0000 1.0000 0.7917 1.0000 1.0000 1.0000 0.5005 1.0000 1.0000 1.0000
0.0000 0.2359 0.9922 1.0000 0.0000 0.6260 0.9945 0.9998 0.0000 0.5005 0.9776 0.9997
0.0000 0.0047 0.6677 0.9794 0.0000 0.0451 0.7067 0.9356 0.0000 0.0225 0.5005 0.9071
0.0000 0.0001 0.2041 0.7603 0.0000 0.0018 0.2662 0.5248 0.0000 0.0003 0.0933 0.5005

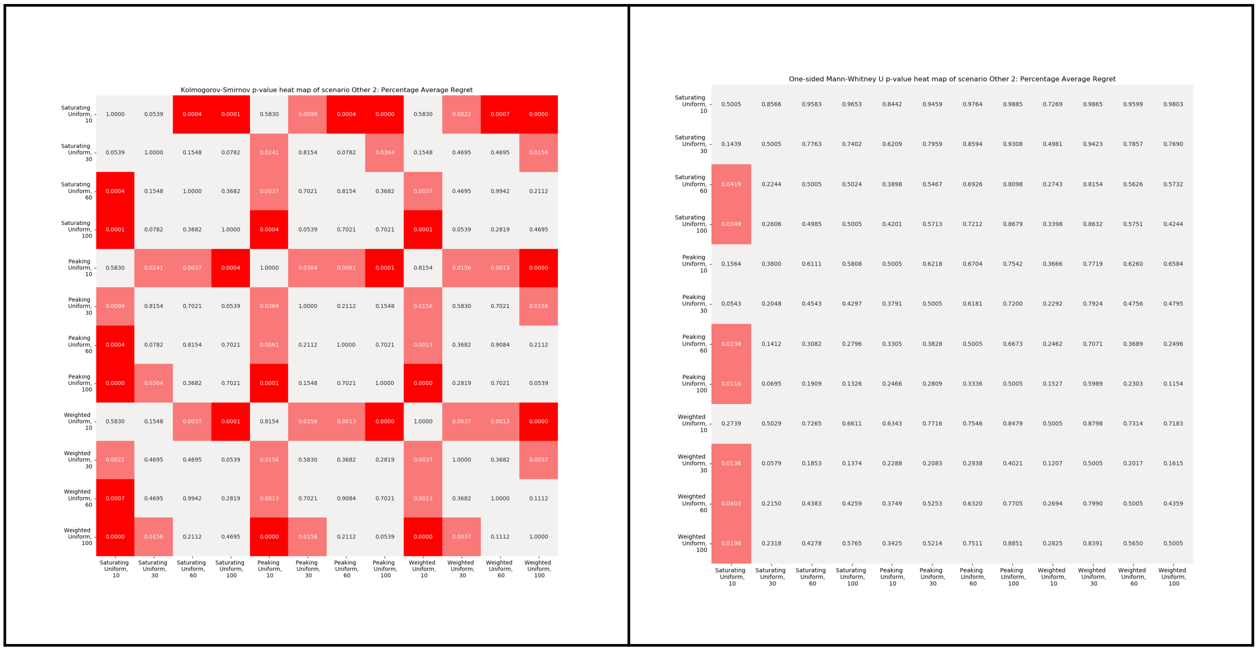

Figure Supplementary.Statistical.Obj1.X2. Kolmogorov–Smirnov (left) and Mann-Whitney U (right) heatmaps of p-values for objective 1, Scenario Other 2. These are for the metrics of PSR (top), Absolute Inaccuracy (middle) and PAR (bottom). Cells with a light pink hue represent the test statistic for that comparison would be significant under the threshold p<0.05, cells with a red hue represent the test statistic for that comparison would be significant under the threshold p<0.05 with Bonferri multiple comparison correction.

<u>Scenario Other 3</u>

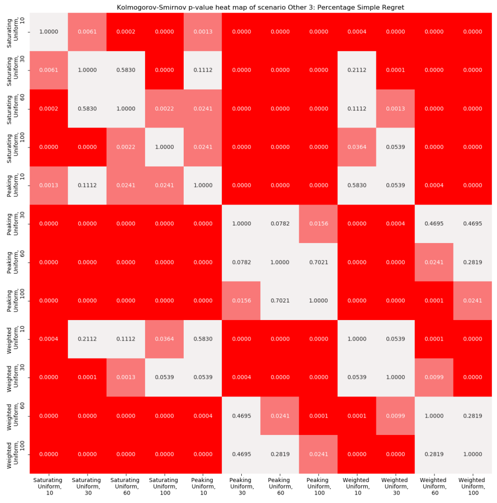
Kolmogorov-Smirnov p-value heat map of scenario Other 3: Percentage Simple Regret
Saturating Uniform, 10
Saturating Uniform, 30
Saturating Uniform, 60
Saturating Uniform, 100
Peaking Uniform, 10
Peaking Uniform, 30
Peaking Uniform, 60
Peaking Uniform, 100
Weighted Uniform, 10
Weighted Uniform, 30
Weighted Uniform, 60
Weighted Uniform, 100

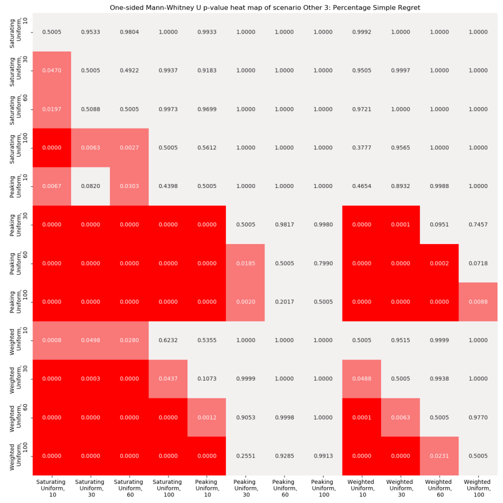
One-sided Mann-Whitney U p-value heat map of scenario Other 3: Percentage Simple Regret
Saturating Uniform, 10
Saturating Uniform, 30
Saturating Uniform, 60
Saturating Uniform, 100
Peaking Uniform, 10
Peaking Uniform, 30
Peaking Uniform, 60
Peaking Uniform, 100
Weighted Uniform, 10
Weighted Uniform, 30
Weighted Uniform, 60
Weighted Uniform, 100

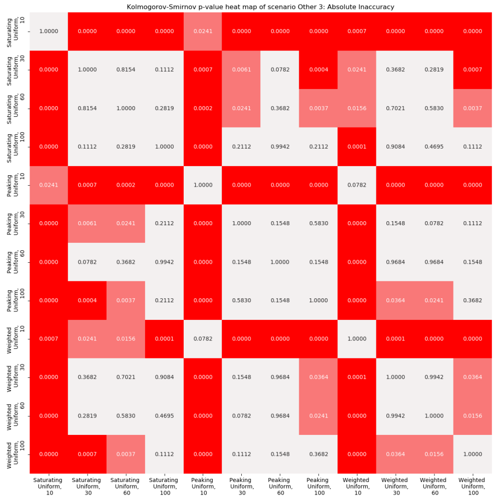
Kolmogorov-Smirnov p-value heat map of scenario Other 3: Absolute Inaccuracy
Saturating Uniform, 10
Saturating Uniform, 30
Saturating Uniform, 60
Saturating Uniform, 100
Peaking Uniform, 10
Peaking Uniform, 30
Peaking Uniform, 60
Peaking Uniform, 100
Weighted Uniform, 10
Weighted Uniform, 30
Weighted Uniform, 60
Weighted Uniform, 100

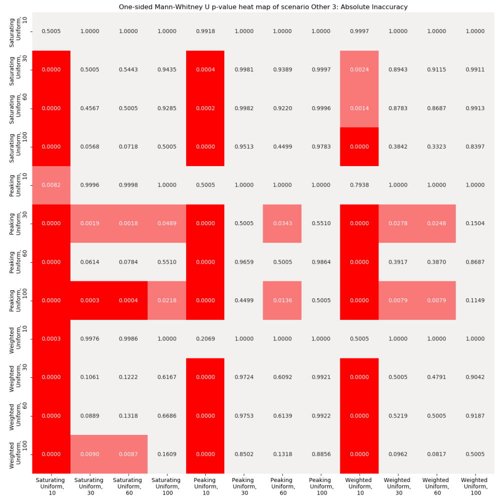
One-sided Mann-Whitney U p-value heat map of scenario Other 3: Absolute Inaccuracy
Saturating Uniform, 10
Saturating Uniform, 30
Saturating Uniform, 60
Saturating Uniform, 100
Peaking Uniform, 10
Peaking Uniform, 30
Peaking Uniform, 60
Peaking Uniform, 100
Weighted Uniform, 10
Weighted Uniform, 30
Weighted Uniform, 60
Weighted Uniform, 100

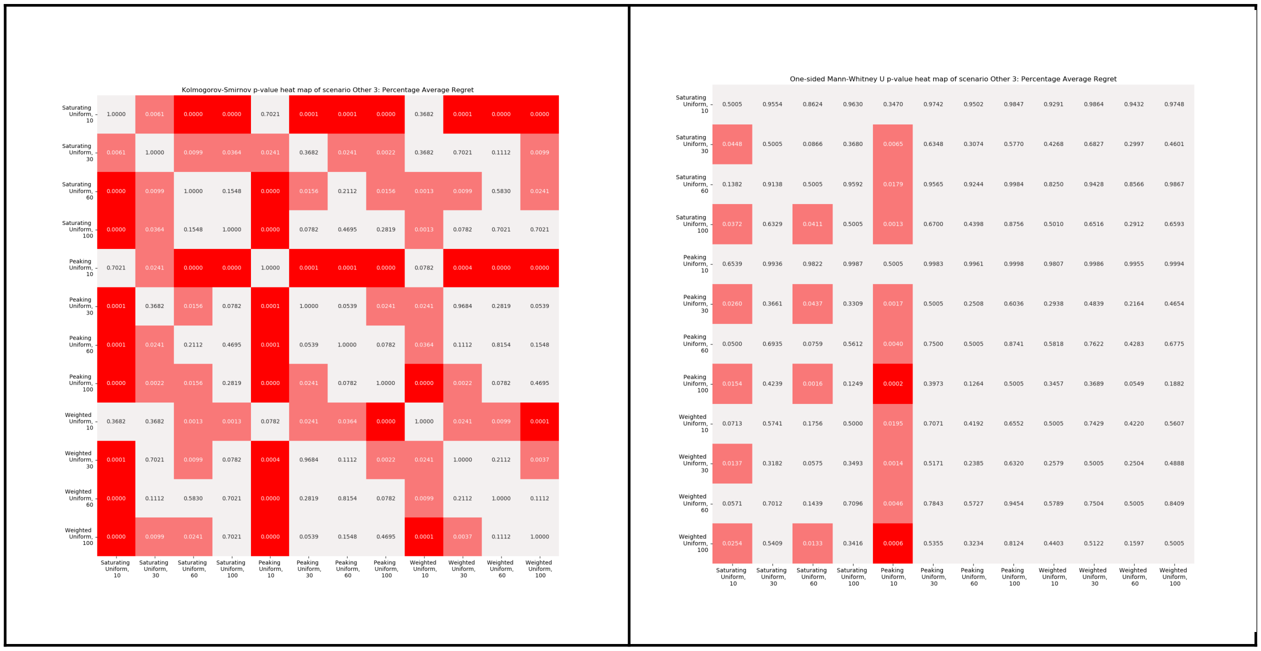

Figure Supplementary.Statistical.Obj1.X3. Kolmogorov–Smirnov (left) and Mann-Whitney U (right) heatmaps of p-values for objective 1, Scenario Other 3. These are for the metrics of PSR (top), Absolute Inaccuracy (middle) and PAR (bottom). Cells with a light pink hue represent the test statistic for that comparison would be significant under the threshold p<0.05, cells with a red hue represent the test statistic for that comparison would be significant under the threshold p<0.05 with Bonferri multiple comparison correction.

Scenario Other 4

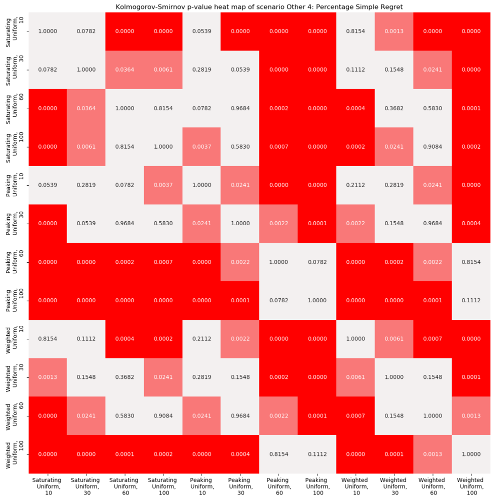
Kolmogorov-Smirnov p-value heat map of scenario Other 4: Percentage Simple Regret
Saturating Uniform, 10
Saturating Uniform, 30
Saturating Uniform, 60
Saturating Uniform, 100
Peaking Uniform, 10
Peaking Uniform, 30
Peaking Uniform, 60
Peaking Uniform, 100
Weighted Uniform, 10
Weighted Uniform, 30
Weighted Uniform, 60
Weighted Uniform, 100

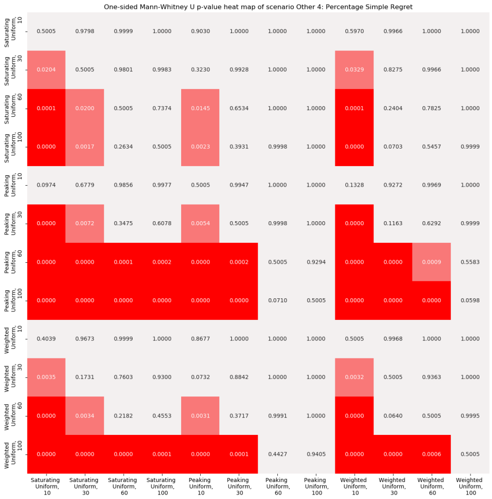
One-sided Mann-Whitney U p-value heat map of scenario Other 4: Percentage Simple Regret
Saturating Uniform, 10
Saturating Uniform, 30
Saturating Uniform, 60
Saturating Uniform, 100
Peaking Uniform, 10
Peaking Uniform, 30
Peaking Uniform, 60
Peaking Uniform, 100
Weighted Uniform, 10
Weighted Uniform, 30
Weighted Uniform, 60
Weighted Uniform, 100

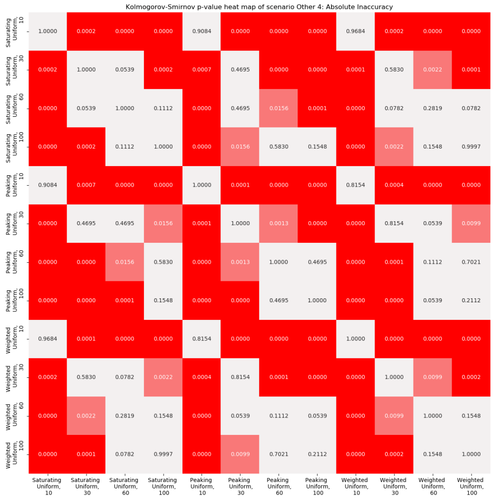
Kolmogorov-Smirnov p-value heat map of scenario Other 4: Absolute Inaccuracy
Saturating Uniform, 10
Saturating Uniform, 30
Saturating Uniform, 60
Saturating Uniform, 100
Peaking Uniform, 10
Peaking Uniform, 30
Peaking Uniform, 60
Peaking Uniform, 100
Weighted Uniform, 10
Weighted Uniform, 30
Weighted Uniform, 60
Weighted Uniform, 100

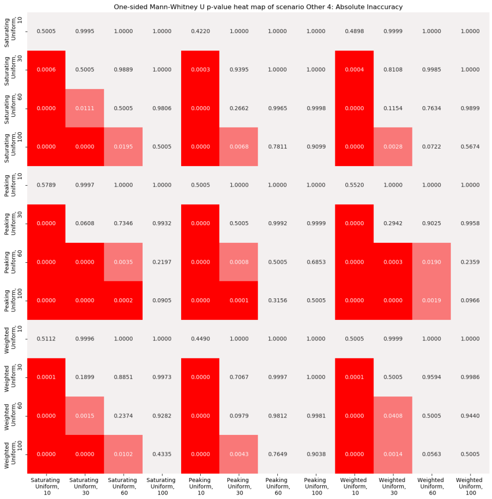
One-sided Mann-Whitney U p-value heat map of scenario Other 4: Absolute Inaccuracy
Saturating Uniform, 10
Saturating Uniform, 30
Saturating Uniform, 60
Saturating Uniform, 100
Peaking Uniform, 10
Peaking Uniform, 30
Peaking Uniform, 60
Peaking Uniform, 100
Weighted Uniform, 10
Weighted Uniform, 30
Weighted Uniform, 60
Weighted Uniform, 100

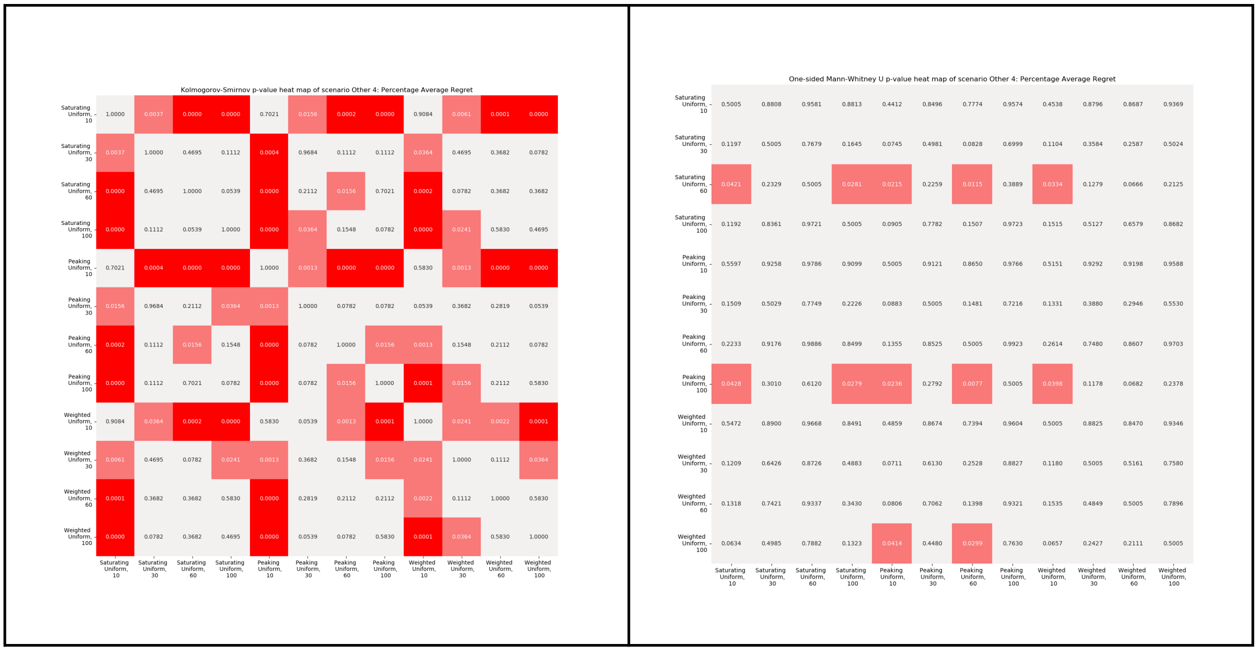

Figure Supplementary.Statistical.Obj1.X4. Kolmogorov–Smirnov (left) and Mann-Whitney U (right) heatmaps of p-values for objective 1, Scenario Other 4. These are for the metrics of PSR (top), Absolute Inaccuracy (middle) and PAR (bottom). Cells with a light pink hue represent the test statistic for that comparison would be significant under the threshold p<0.05, cells with a red hue represent the test statistic for that comparison would be significant under the threshold p<0.05 with Bonferri multiple comparison correction.

Objective 2 Total Analysis

Here we show the p-values for objective 2 for the metrics of PSR, Absolute Inaccuracy, and PAR. These are the metrics for the combined data of all scenarios. This is the data in figures 10, 11b and 12 respectively.

For interpretation, the Kolmogorov–Smirnov heatmaps are symmetric, with significance representing evidence that the true distribution for the approach-scenario test metrics of PSR, Absolute Inaccuracy, and PAR differ between the two dose-optimisation approaches across all scenarios. The One-sided Mann-Whitney U test heatmaps are not symmetric, with significance for the cell in row A and column B representing ‘statistically significant’ evidence that approach

A was preferable to approach B with regards to that metric (eg. lower PSR, lower Absolute Inaccuracy, Lower PAR).

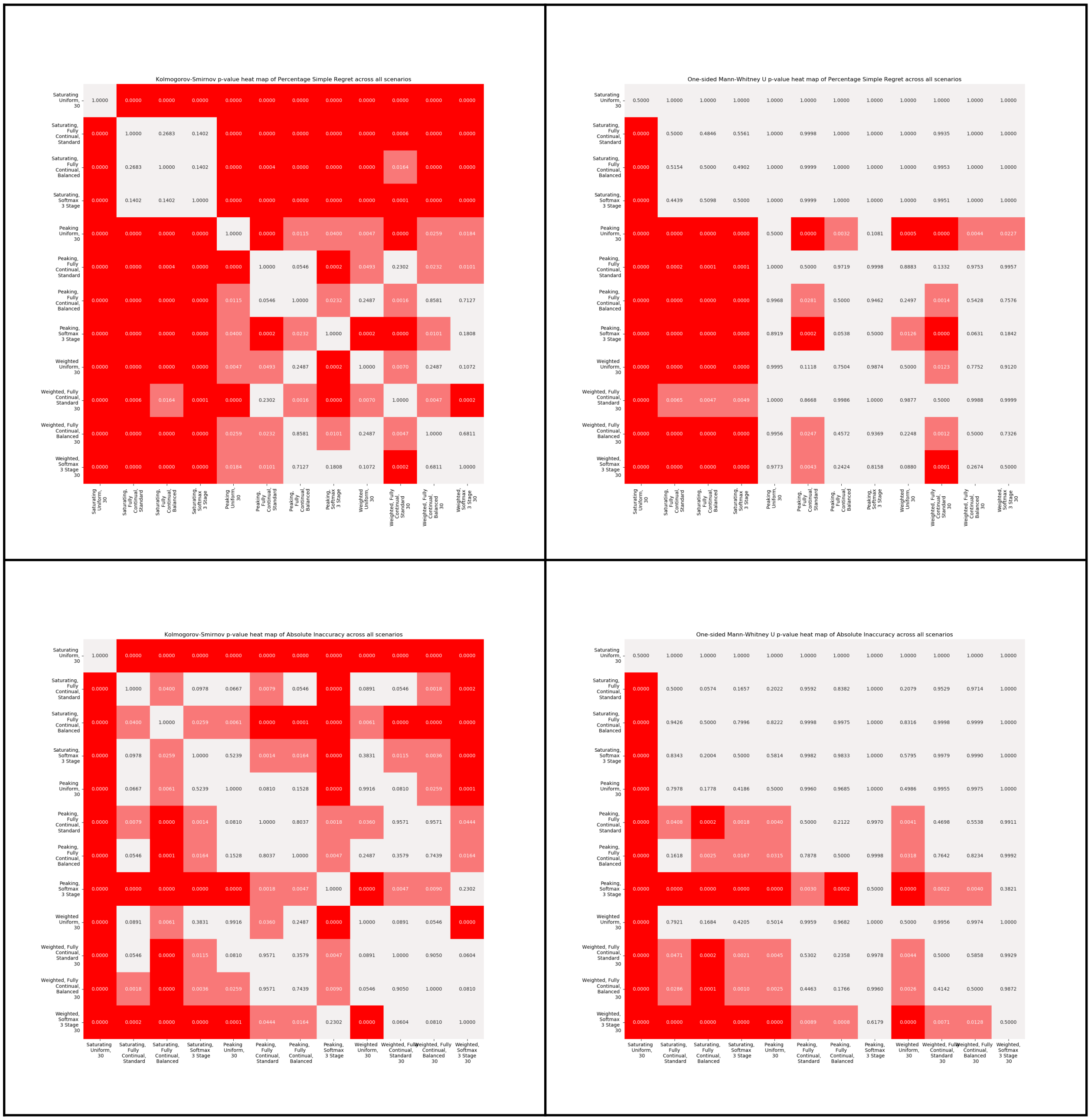

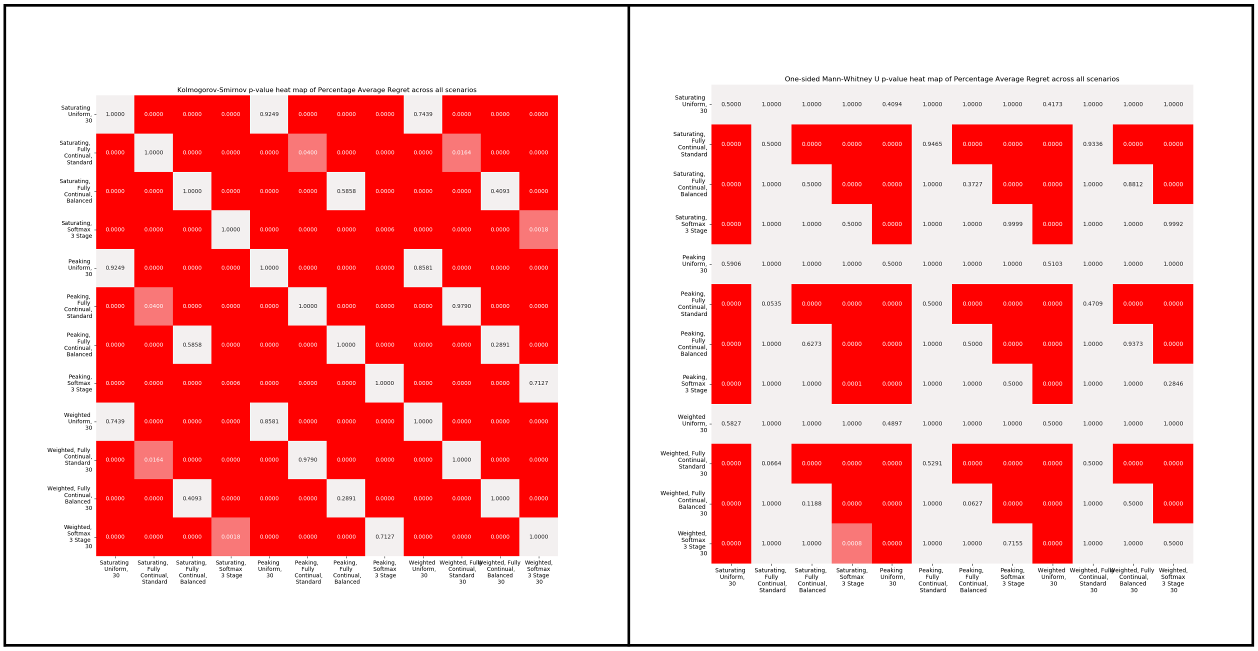

Figure Supplementary.Statistical.Obj2.Total. Kolmogorov–Smirnov (left) and Mann-Whitney U (right) heatmaps of p-values for objective 2 across all scenarios. These are for the metrics of PSR (top), Absolute Inaccuracy (middle) and PAR (bottom). Cells with a light pink hue represent the test statistic for that comparison would be significant under the threshold p<0.05, cells with a red hue represent the test statistic for that comparison would be significant under the threshold p<0.05 with Bonferri multiple comparison correction.

Objective 2 Scenario Specific

Here we show the p-values for objective 2 for the metrics of PSR, Absolute Inaccuracy, and PAR. These are the metrics for the data stratified on scenario. This is the data in Supplementary 12.

For interpretation, the Kolmogorov–Smirnov heatmaps are symmetric, with significance representing evidence that the true distribution for the approach-scenario test metrics of PSR, Absolute Inaccuracy, and PAR differ between the two dose-optimisation approaches across all scenarios. The One-sided Mann-Whitney U test heatmaps are not symmetric, with significance for the cell in row A and column B representing 'statistically significant' evidence that approach A was preferable to approach B with regards to that metric (eg. lower PSR, lower Absolute Inaccuracy, Lower PAR).

Scenario Saturating 1

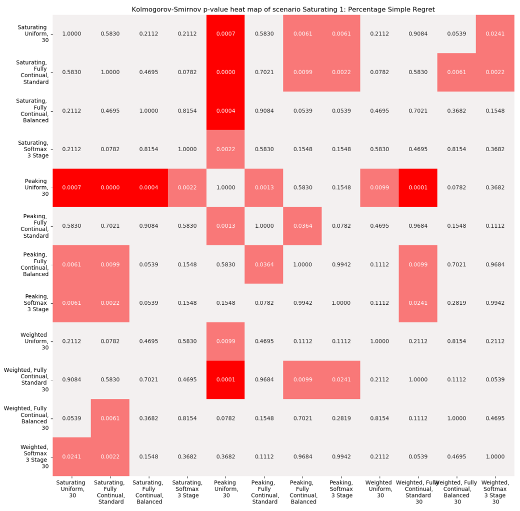

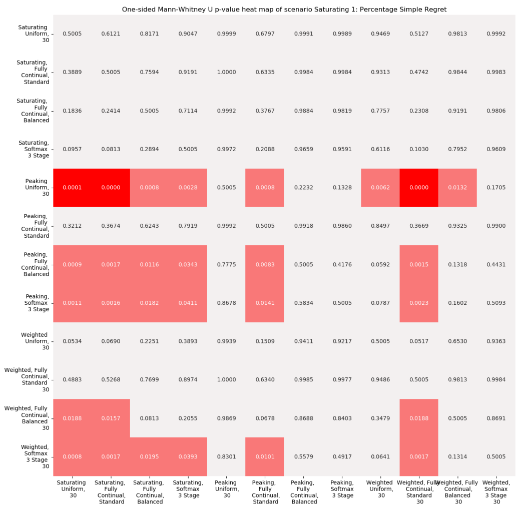

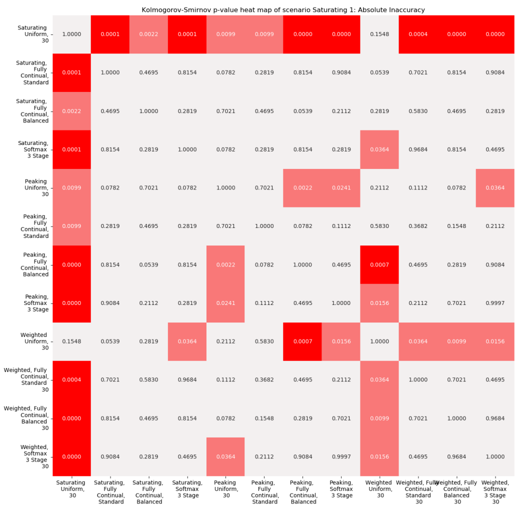

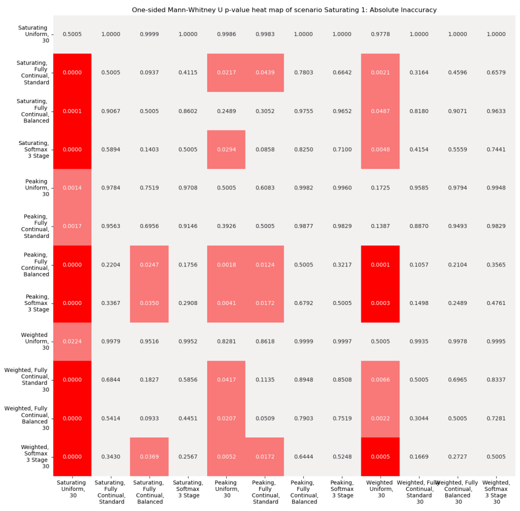

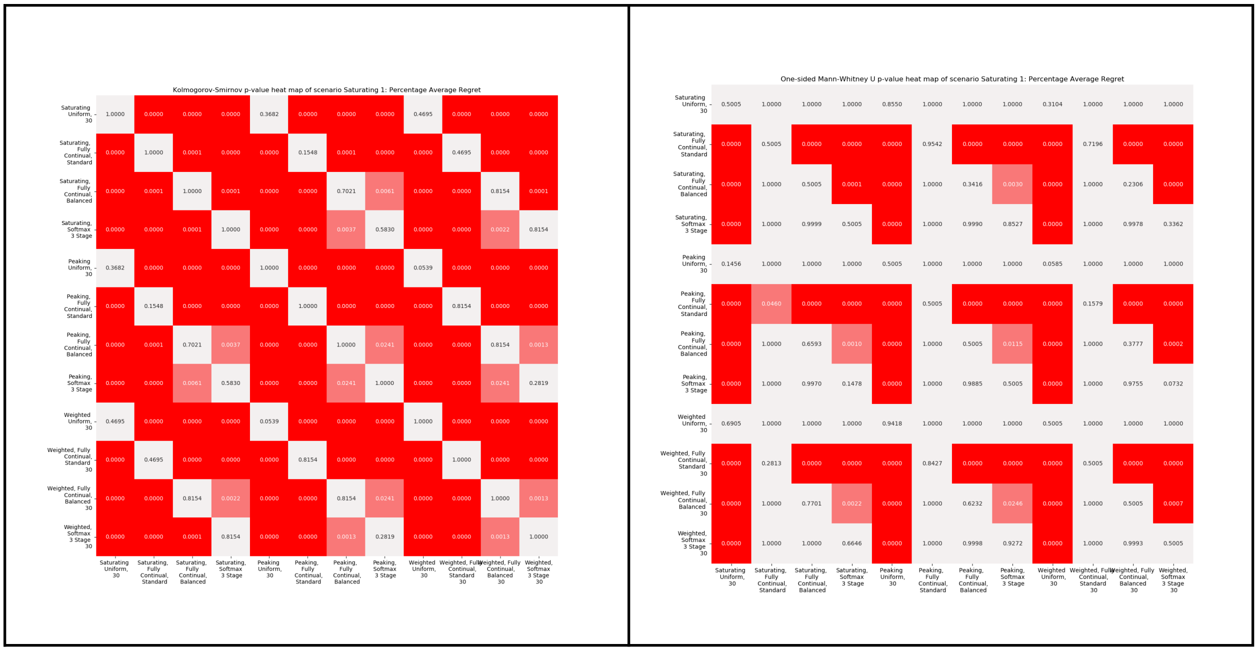

Figure Supplementary.Statistical.Obj2.S1. Kolmogorov–Smirnov (left) and Mann-Whitney U (right) heatmaps of p-values for objective 1, Scenario Saturating 1. These are for the metrics of PSR (top), Absolute Inaccuracy (middle) and PAR (bottom). Cells with a light pink hue represent the test statistic for that comparison would be significant under the threshold p<0.05, cells with a red hue represent the test statistic for that comparison would be significant under the threshold p<0.05 with Bonferri multiple comparison correction.

Scenario Saturating 2

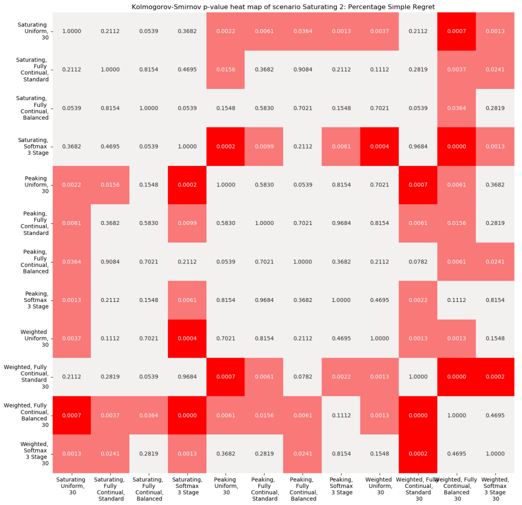
Kolmogorov-Smirnov p-value heat map of scenario Saturating 2: Percentage Simple Regret
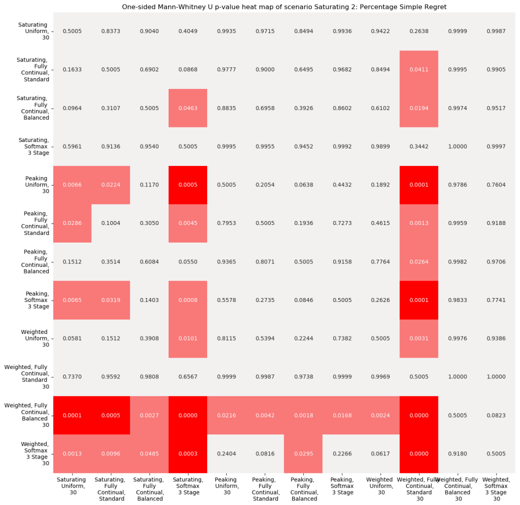
One-sided Mann-Whitney U p-value heat map of scenario Saturating 2: Percentage Simple Regret
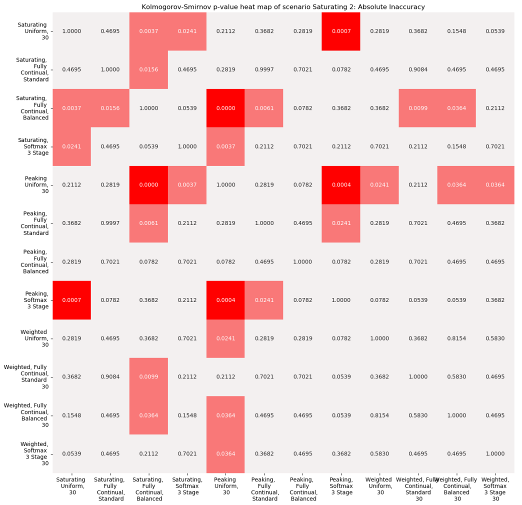
Kolmogorov-Smirnov p-value heat map of scenario Saturating 2: Absolute Inaccuracy
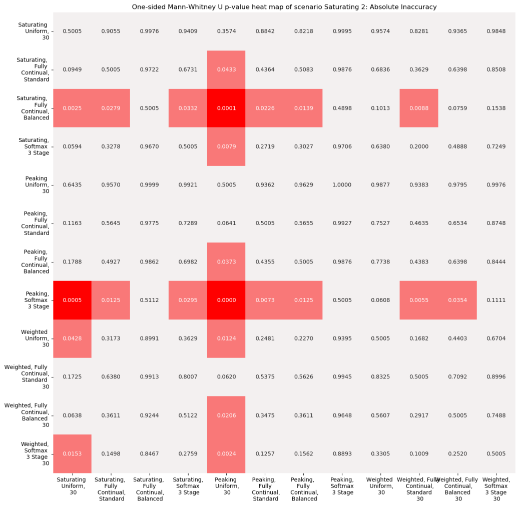
One-sided Mann-Whitney U p-value heat map of scenario Saturating 2: Absolute Inaccuracy

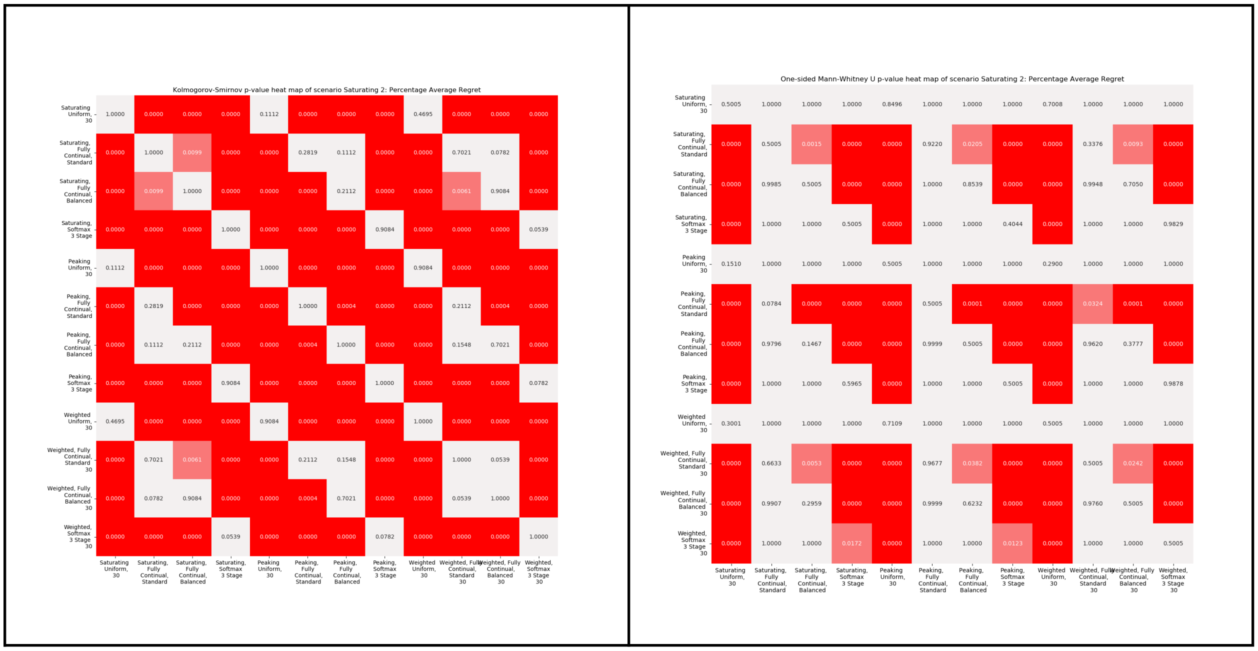

Figure Supplementary.Statistical.Obj2.S2. Kolmogorov–Smirnov (left) and Mann-Whitney U (right) heatmaps of p-values for objective 1, Scenario Saturating 2. These are for the metrics of PSR (top), Absolute Inaccuracy (middle) and PAR (bottom). Cells with a light pink hue represent the test statistic for that comparison would be significant under the threshold p<0.05, cells with a red hue represent the test statistic for that comparison would be significant under the threshold p<0.05 with Bonferri multiple comparison correction.

Scenario Saturating 3

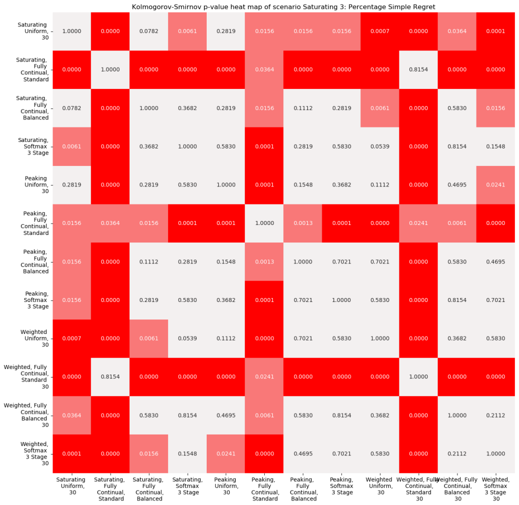
Kolmogorov-Smirnov p-value heat map of scenario Saturating 3: Percentage Simple Regret

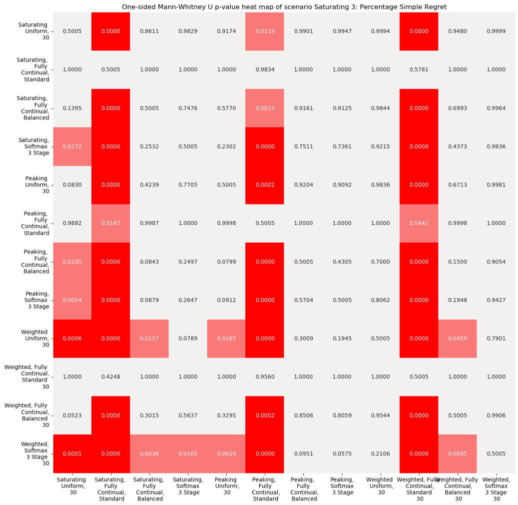
One-sided Mann-Whitney U p-value heat map of scenario Saturating 3: Percentage Simple Regret

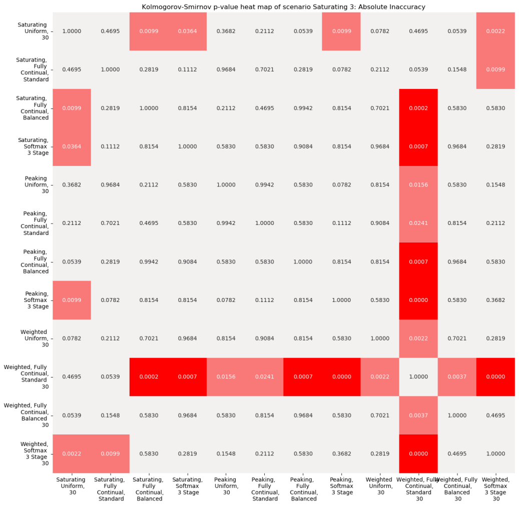
Kolmogorov-Smirnov p-value heat map of scenario Saturating 3: Absolute Inaccuracy

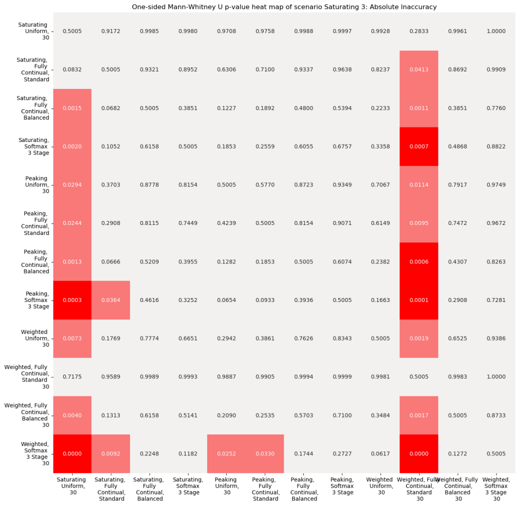
One-sided Mann-Whitney U p-value heat map of scenario Saturating 3: Absolute Inaccuracy

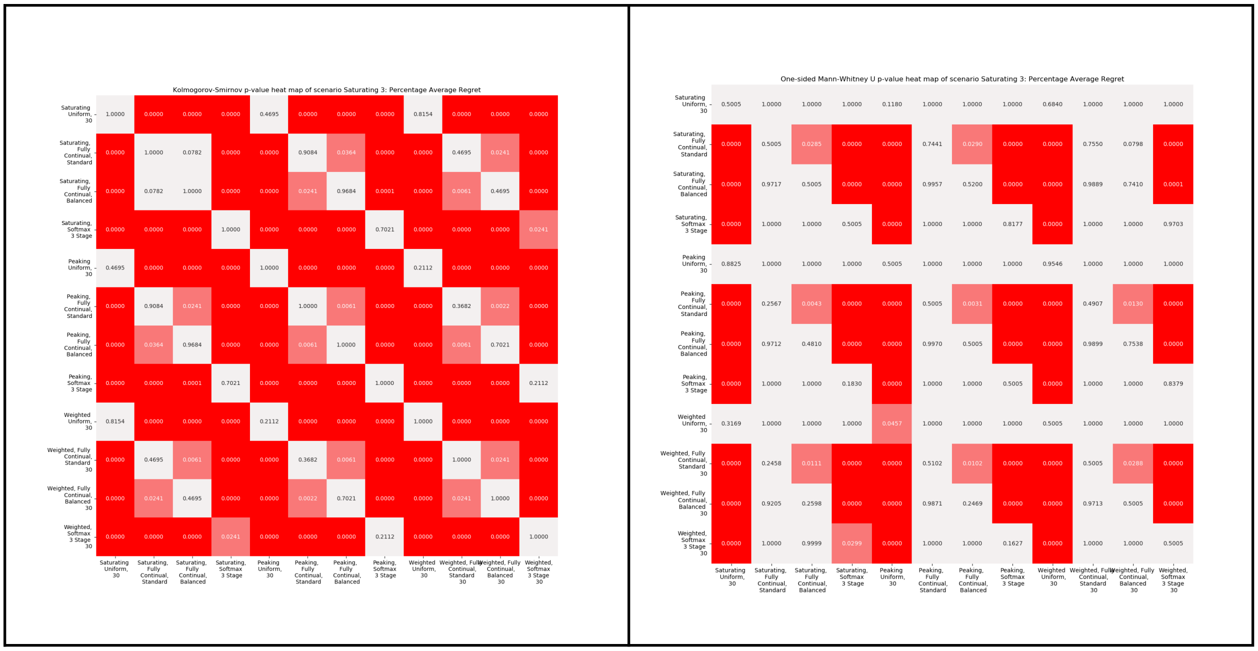

Figure Supplementary.Statistical.Obj2.S3. Kolmogorov–Smirnov (left) and Mann-Whitney U (right) heatmaps of p-values for objective 1, Scenario Saturating 3. These are for the metrics of PSR (top), Absolute Inaccuracy (middle) and PAR (bottom). Cells with a light pink hue represent the test statistic for that comparison would be significant under the threshold $p<0.05$, cells with a red hue represent the test statistic for that comparison would be significant under the threshold $p<0.05$ with Bonferri multiple comparison correction.

Scenario Saturating 4

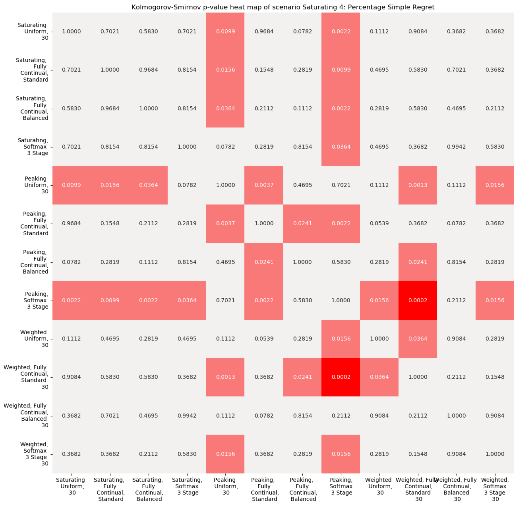
Kolmogorov-Smirnov p-value heat map of scenario Saturating 4: Percentage Simple Regret

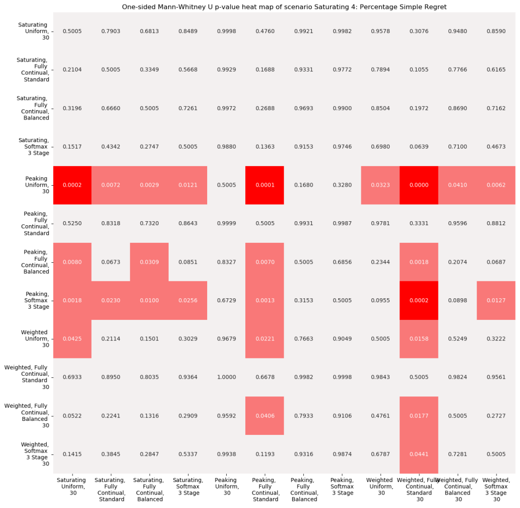
One-sided Mann-Whitney U p-value heat map of scenario Saturating 4: Percentage Simple Regret

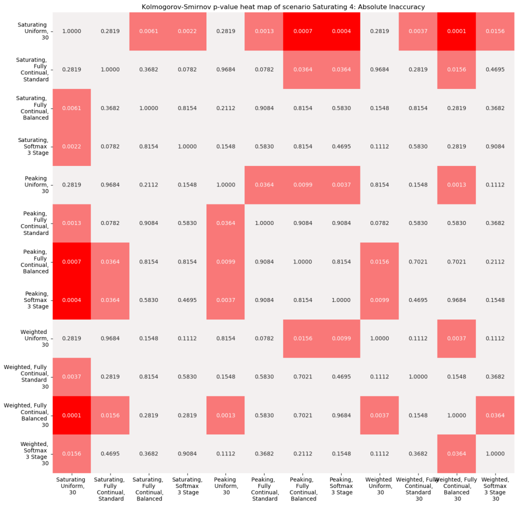
Kolmogorov-Smirnov p-value heat map of scenario Saturating 4: Absolute Inaccuracy

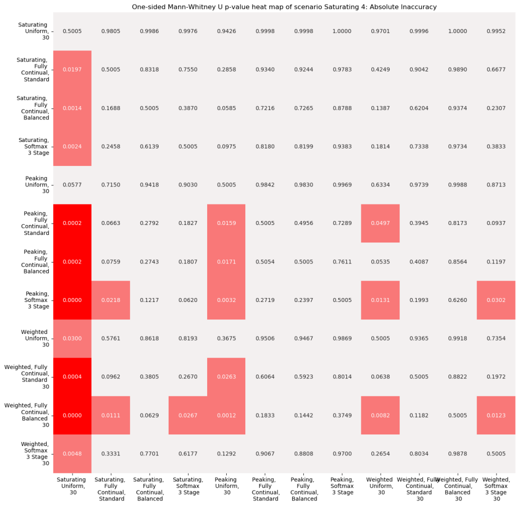
One-sided Mann-Whitney U p-value heat map of scenario Saturating 4: Absolute Inaccuracy

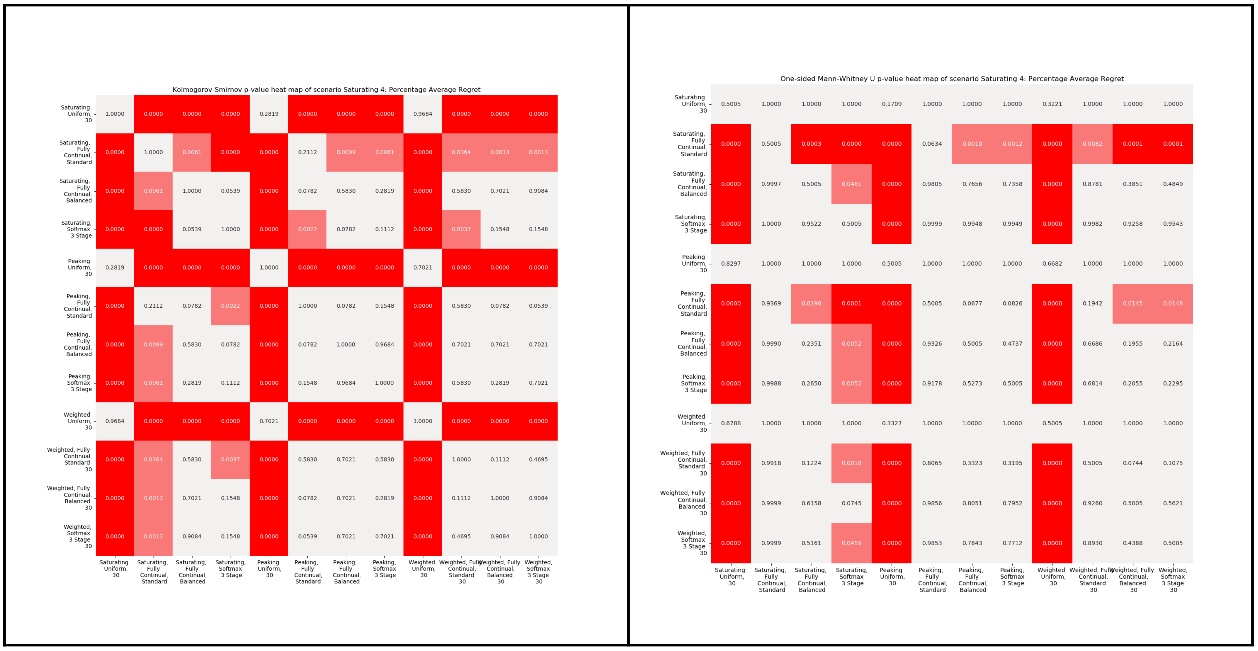

Figure Supplementary.Statistical.Obj2.S4. Kolmogorov–Smirnov (left) and Mann-Whitney U (right) heatmaps of p-values for objective 1, Scenario Saturating 4. These are for the metrics of PSR (top), Absolute Inaccuracy (middle) and PAR (bottom). Cells with a light pink hue represent the test statistic for that comparison would be significant under the threshold p<0.05, cells with a red hue represent the test statistic for that comparison would be significant under the threshold p<0.05 with Bonferri multiple comparison correction.

Scenario Saturating 5

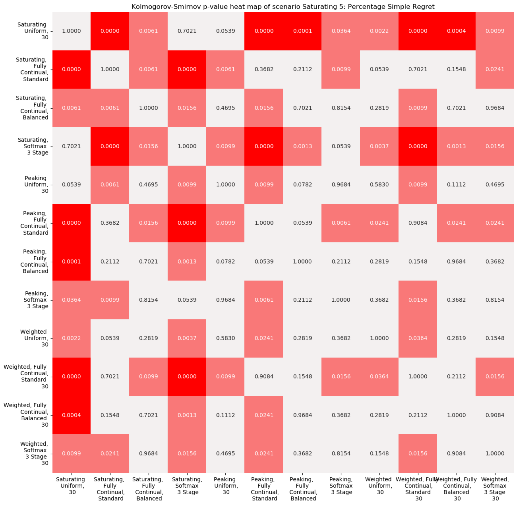
Kolmogorov-Smirnov p-value heat map of scenario Saturating 5: Percentage Simple Regret
Saturating Uniform, 30 – 1.0000 0.0000 0.0061 0.7021 0.0539 0.0000 0.0001 0.0364 0.0022 0.0000 0.0004 0.0099
Saturating, Fully Continual, Standard – 0.0000 1.0000 0.0061 0.0000 0.0061 0.3682 0.2112 0.0099 0.0539 0.7021 0.1548 0.0241
Saturating, Fully Continual, Balanced – 0.0061 0.0061 1.0000 0.0156 0.4695 0.0156 0.7021 0.8154 0.2819 0.0099 0.7021 0.9684
Saturating, Softmax 3 Stage – 0.7021 0.0000 0.0156 1.0000 0.0099 0.0000 0.0013 0.0539 0.0037 0.0000 0.0013 0.0156
Peaking Uniform, 30 – 0.0539 0.0061 0.4695 0.0099 1.0000 0.0099 0.0782 0.9684 0.5830 0.0099 0.1112 0.4695
Peaking, Fully Continual, Standard – 0.0000 0.3682 0.0156 0.0000 0.0099 1.0000 0.0539 0.0061 0.0241 0.9084 0.0241 0.0241
Peaking, Fully Continual, Balanced – 0.0001 0.2112 0.7021 0.0013 0.0782 0.0539 1.0000 0.2112 0.2819 0.1548 0.9684 0.3682
Peaking, Softmax 3 Stage – 0.0364 0.0099 0.8154 0.0539 0.9684 0.0061 0.2112 1.0000 0.3682 0.0156 0.3682 0.8154
Weighted Uniform, 30 – 0.0022 0.0539 0.2819 0.0037 0.5830 0.0241 0.2819 0.3682 1.0000 0.0364 0.2819 0.1548
Weighted, Fully Continual, Standard 30 – 0.0000 0.7021 0.0099 0.0000 0.0099 0.9084 0.1548 0.0156 0.0364 1.0000 0.2112 0.0156
Weighted, Fully Continual, Balanced 30 – 0.0004 0.1548 0.7021 0.0013 0.1112 0.0241 0.9684 0.3682 0.2819 0.2112 1.0000 0.9084
Weighted, Softmax 3 Stage 30 – 0.0099 0.0241 0.9684 0.0156 0.4695 0.0241 0.3682 0.8154 0.1548 0.0156 0.9084 1.0000
Saturating Uniform, 30
Saturating, Fully Continual, Standard
Saturating, Fully Continual, Balanced
Saturating, Softmax 3 Stage
Peaking Uniform, 30
Peaking, Fully Continual, Standard
Peaking, Fully Continual, Balanced
Peaking, Softmax 3 Stage
Weighted Uniform, 30
Weighted, Fully Continual, Standard 30
Weighted, Fully Continual, Balanced 30
Weighted, Softmax 3 Stage 30

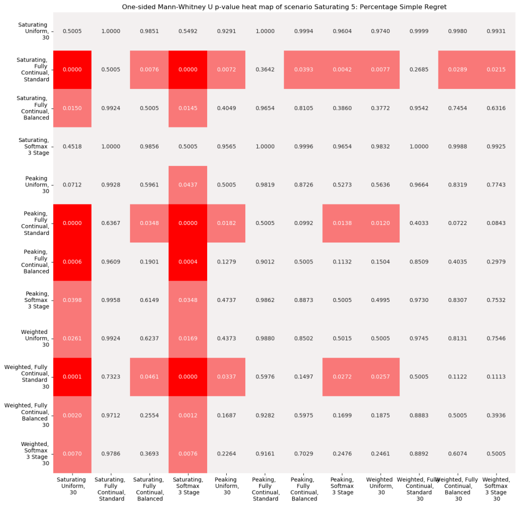
One-sided Mann-Whitney U p-value heat map of scenario Saturating 5: Percentage Simple Regret
Saturating Uniform, 30 – 0.5005 1.0000 0.9851 0.5492 0.9291 1.0000 0.9994 0.9604 0.9740 0.9999 0.9980 0.9931
Saturating, Fully Continual, Standard – 0.0000 0.5005 0.0076 0.0000 0.0072 0.3642 0.0393 0.0042 0.0077 0.2685 0.0289 0.0215
Saturating, Fully Continual, Balanced – 0.0150 0.9924 0.5005 0.0145 0.4049 0.9654 0.8105 0.3860 0.3772 0.9542 0.7454 0.6316
Saturating, Softmax 3 Stage – 0.4518 1.0000 0.9856 0.5005 0.9565 1.0000 0.9996 0.9654 0.9832 1.0000 0.9988 0.9925
Peaking Uniform, 30 – 0.0712 0.9928 0.5961 0.0437 0.5005 0.9819 0.8726 0.5273 0.5636 0.9664 0.8319 0.7743
Peaking, Fully Continual, Standard – 0.0000 0.6367 0.0348 0.0000 0.0182 0.5005 0.0992 0.0138 0.0120 0.4033 0.0722 0.0843
Peaking, Fully Continual, Balanced – 0.0006 0.9609 0.1901 0.0004 0.1279 0.9012 0.5005 0.1132 0.1504 0.8509 0.4035 0.2979
Peaking, Softmax 3 Stage – 0.0398 0.9958 0.6149 0.0348 0.4737 0.9862 0.8873 0.5005 0.4995 0.9730 0.8307 0.7532
Weighted Uniform, 30 – 0.0261 0.9924 0.6237 0.0169 0.4373 0.9880 0.8502 0.5015 0.5005 0.9745 0.8131 0.7546
Weighted, Fully Continual, Standard 30 – 0.0001 0.7323 0.0461 0.0000 0.0337 0.5976 0.1497 0.0272 0.0257 0.5005 0.1122 0.1113
Weighted, Fully Continual, Balanced 30 – 0.0020 0.9712 0.2554 0.0012 0.1687 0.9282 0.5975 0.1699 0.1875 0.8883 0.5005 0.3936
Weighted, Softmax 3 Stage 30 – 0.0070 0.9786 0.3693 0.0076 0.2264 0.9161 0.7029 0.2476 0.2461 0.8892 0.6074 0.5005
Saturating Uniform, 30
Saturating, Fully Continual, Standard
Saturating, Fully Continual, Balanced
Saturating, Softmax 3 Stage
Peaking Uniform, 30
Peaking, Fully Continual, Standard
Peaking, Fully Continual, Balanced
Peaking, Softmax 3 Stage
Weighted Uniform, 30
Weighted, Fully Continual, Standard 30
Weighted, Fully Continual, Balanced 30
Weighted, Softmax 3 Stage 30

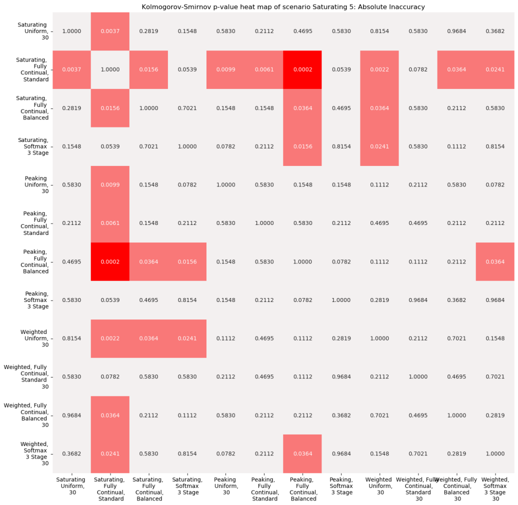
Kolmogorov-Smirnov p-value heat map of scenario Saturating 5: Absolute Inaccuracy
Saturating Uniform, 30 – 1.0000 0.0037 0.2819 0.1548 0.5830 0.2112 0.4695 0.5830 0.8154 0.5830 0.9684 0.3682
Saturating, Fully Continual, Standard – 0.0037 1.0000 0.0156 0.0539 0.0099 0.0061 0.0002 0.0539 0.0022 0.0782 0.0364 0.0241
Saturating, Fully Continual, Balanced – 0.2819 0.0156 1.0000 0.7021 0.1548 0.1548 0.0364 0.4695 0.0364 0.5830 0.2112 0.5830
Saturating, Softmax 3 Stage – 0.1548 0.0539 0.7021 1.0000 0.0782 0.2112 0.0156 0.8154 0.0241 0.5830 0.1112 0.8154
Peaking Uniform, 30 – 0.5830 0.0099 0.1548 0.0782 1.0000 0.5830 0.1548 0.1548 0.1112 0.2112 0.5830 0.0782
Peaking, Fully Continual, Standard – 0.2112 0.0061 0.1548 0.2112 0.5830 1.0000 0.5830 0.2112 0.4695 0.4695 0.2112 0.2112
Peaking, Fully Continual, Balanced – 0.4695 0.0002 0.0364 0.0156 0.1548 0.5830 1.0000 0.0782 0.1112 0.1112 0.2112 0.0364
Peaking, Softmax 3 Stage – 0.5830 0.0539 0.4695 0.8154 0.1548 0.2112 0.0782 1.0000 0.2819 0.9684 0.3682 0.9684
Weighted Uniform, 30 – 0.8154 0.0022 0.0364 0.0241 0.1112 0.4695 0.1112 0.2819 1.0000 0.2112 0.7021 0.1548
Weighted, Fully Continual, Standard 30 – 0.5830 0.0782 0.5830 0.5830 0.2112 0.4695 0.1112 0.9684 0.2112 1.0000 0.4695 0.7021
Weighted, Fully Continual, Balanced 30 – 0.9684 0.0364 0.2112 0.1112 0.5830 0.2112 0.2112 0.3682 0.7021 0.4695 1.0000 0.2819
Weighted, Softmax 3 Stage 30 – 0.3682 0.0241 0.5830 0.8154 0.0782 0.2112 0.0364 0.9684 0.1548 0.7021 0.2819 1.0000
Saturating Uniform, 30
Saturating, Fully Continual, Standard
Saturating, Fully Continual, Balanced
Saturating, Softmax 3 Stage
Peaking Uniform, 30
Peaking, Fully Continual, Standard
Peaking, Fully Continual, Balanced
Peaking, Softmax 3 Stage
Weighted Uniform, 30
Weighted, Fully Continual, Standard 30
Weighted, Fully Continual, Balanced 30
Weighted, Softmax 3 Stage 30

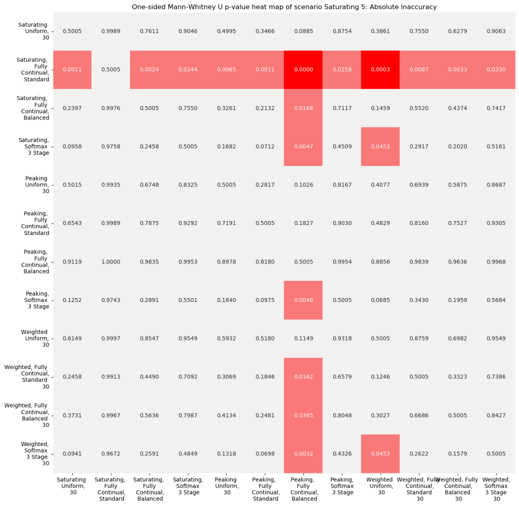
One-sided Mann-Whitney U p-value heat map of scenario Saturating 5: Absolute Inaccuracy
Saturating Uniform, 30 – 0.5005 0.9989 0.7611 0.9046 0.4995 0.3466 0.0885 0.8754 0.3861 0.7550 0.6279 0.9063
Saturating, Fully Continual, Standard – 0.0011 0.5005 0.0024 0.0244 0.0065 0.0011 0.0000 0.0258 0.0003 0.0087 0.0033 0.0330
Saturating, Fully Continual, Balanced – 0.2397 0.9976 0.5005 0.7550 0.3261 0.2132 0.0166 0.7117 0.1459 0.5520 0.4374 0.7417
Saturating, Softmax 3 Stage – 0.0958 0.9758 0.2458 0.5005 0.1682 0.0712 0.0047 0.4509 0.0453 0.2917 0.2020 0.5161
Peaking Uniform, 30 – 0.5015 0.9935 0.6748 0.8325 0.5005 0.2817 0.1026 0.8167 0.4077 0.6939 0.5875 0.8687
Peaking, Fully Continual, Standard – 0.6543 0.9989 0.7875 0.9292 0.7191 0.5005 0.1827 0.9030 0.4829 0.8160 0.7527 0.9305
Peaking, Fully Continual, Balanced – 0.9119 1.0000 0.9835 0.9953 0.8978 0.8180 0.5005 0.9954 0.8856 0.9839 0.9636 0.9968
Peaking, Softmax 3 Stage – 0.1252 0.9743 0.2891 0.5501 0.1840 0.0975 0.0046 0.5005 0.0685 0.3430 0.1959 0.5684
Weighted Uniform, 30 – 0.6149 0.9997 0.8547 0.9549 0.5932 0.5180 0.1149 0.9318 0.5005 0.8759 0.6982 0.9549
Weighted, Fully Continual, Standard 30 – 0.2458 0.9913 0.4490 0.7092 0.3069 0.1846 0.0162 0.6579 0.1246 0.5005 0.3323 0.7386
Weighted, Fully Continual, Balanced 30 – 0.3731 0.9967 0.5636 0.7987 0.4134 0.2481 0.0365 0.8048 0.3027 0.6686 0.5005 0.8427
Weighted, Softmax 3 Stage 30 – 0.0941 0.9672 0.2591 0.4849 0.1318 0.0698 0.0032 0.4326 0.0453 0.2622 0.1579 0.5005
Saturating Uniform, 30
Saturating, Fully Continual, Standard
Saturating, Fully Continual, Balanced
Saturating, Softmax 3 Stage
Peaking Uniform, 30
Peaking, Fully Continual, Standard
Peaking, Fully Continual, Balanced
Peaking, Softmax 3 Stage
Weighted Uniform, 30
Weighted, Fully Continual, Standard 30
Weighted, Fully Continual, Balanced 30
Weighted, Softmax 3 Stage 30

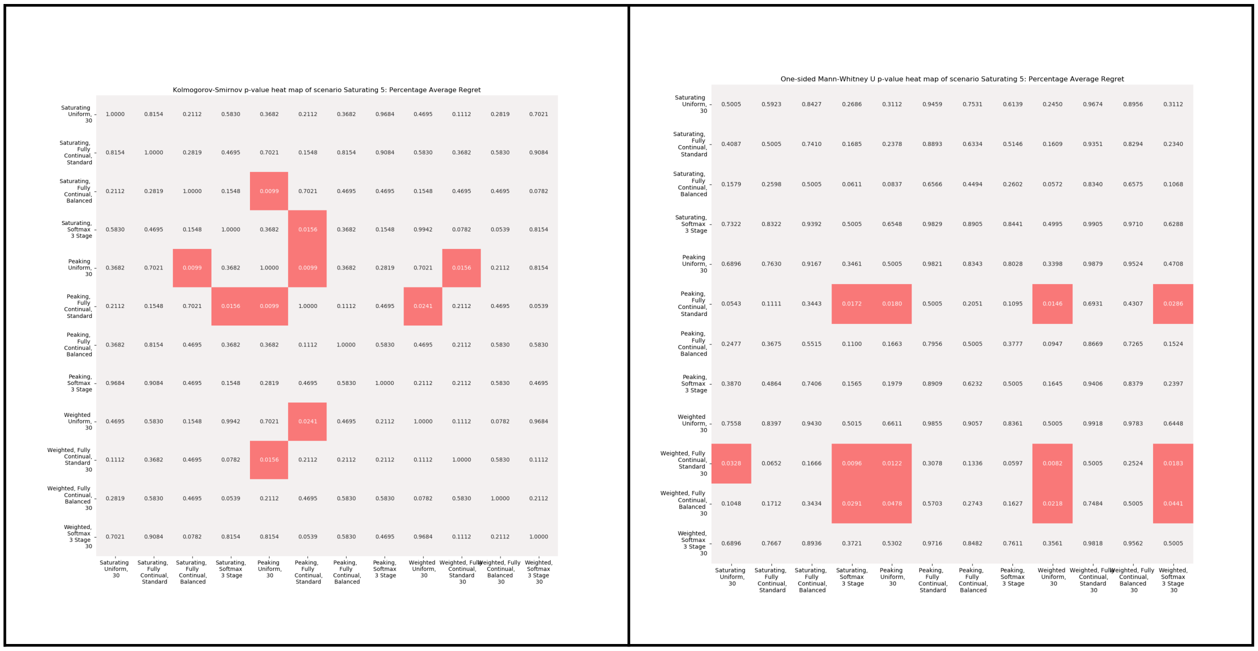

Figure Supplementary.Statistical.Obj2.S5. Kolmogorov–Smirnov (left) and Mann-Whitney U (right) heatmaps of p-values for objective 1, Scenario Saturating 5. These are for the metrics of PSR (top), Absolute Inaccuracy (middle) and PAR (bottom). Cells with a light pink hue represent the test statistic for that comparison would be significant under the threshold p<0.05, cells with a red hue represent the test statistic for that comparison would be significant under the threshold p<0.05 with Bonferri multiple comparison correction.

<u>Scenario Peaking 1</u>

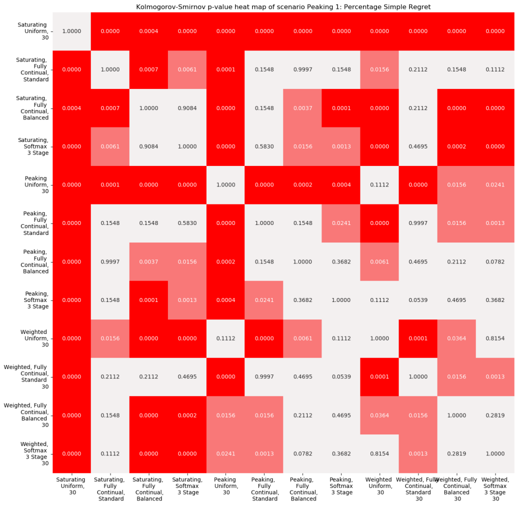
Kolmogorov-Smirnov p-value heat map of scenario Peaking 1: Percentage Simple Regret
Saturating Uniform, 30: 1.0000 0.0000 0.0004 0.0000 0.0000 0.0000 0.0000 0.0000 0.0000 0.0000 0.0000 0.0000
Saturating, Fully Continual, Standard: 0.0000 1.0000 0.0007 0.0061 0.0001 0.1548 0.9997 0.1548 0.0156 0.2112 0.1548 0.1112
Saturating, Fully Continual, Balanced: 0.0004 0.0007 1.0000 0.9084 0.0000 0.1548 0.0037 0.0001 0.0000 0.2112 0.0000 0.0000
Saturating, Softmax 3 Stage: 0.0000 0.0061 0.9084 1.0000 0.0000 0.5830 0.0156 0.0013 0.0000 0.4695 0.0002 0.0000
Peaking Uniform, 30: 0.0000 0.0001 0.0000 0.0000 1.0000 0.0000 0.0002 0.0004 0.1112 0.0000 0.0156 0.0241
Peaking, Fully Continual, Standard: 0.0000 0.1548 0.1548 0.5830 0.0000 1.0000 0.1548 0.0241 0.0000 0.9997 0.0156 0.0013
Peaking, Fully Continual, Balanced: 0.0000 0.9997 0.0037 0.0156 0.0002 0.1548 1.0000 0.3682 0.0061 0.4695 0.2112 0.0782
Peaking, Softmax 3 Stage: 0.0000 0.1548 0.0001 0.0013 0.0004 0.0241 0.3682 1.0000 0.1112 0.0539 0.4695 0.3682
Weighted Uniform, 30: 0.0000 0.0156 0.0000 0.0000 0.1112 0.0000 0.0061 0.1112 1.0000 0.0001 0.0364 0.8154
Weighted, Fully Continual, Standard 30: 0.0000 0.2112 0.2112 0.4695 0.0000 0.9997 0.4695 0.0539 0.0001 1.0000 0.0156 0.0013
Weighted, Fully Continual, Balanced 30: 0.0000 0.1548 0.0000 0.0002 0.0156 0.0156 0.2112 0.4695 0.0364 0.0156 1.0000 0.2819
Weighted, Softmax 3 Stage 30: 0.0000 0.1112 0.0000 0.0000 0.0241 0.0013 0.0782 0.3682 0.8154 0.0013 0.2819 1.0000
Saturating Uniform, 30 | Saturating, Fully Continual, Standard | Saturating, Fully Continual, Balanced | Saturating, Softmax 3 Stage | Peaking Uniform, 30 | Peaking, Fully Continual, Standard | Peaking, Fully Continual, Balanced | Peaking, Softmax 3 Stage | Weighted Uniform, 30 | Weighted, Fully Continual, Standard 30 | Weighted, Fully Continual, Balanced 30 | Weighted, Softmax 3 Stage 30

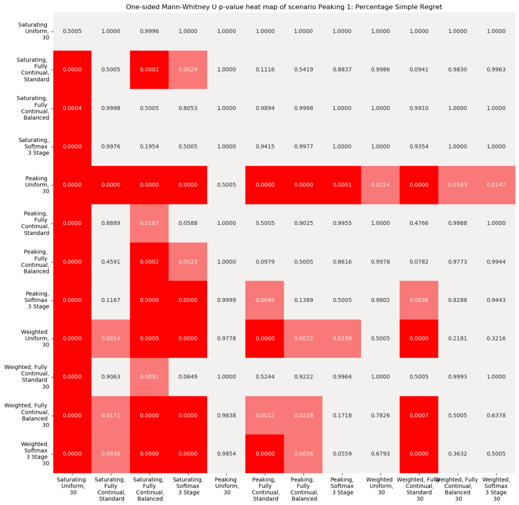
One-sided Mann-Whitney U p-value heat map of scenario Peaking 1: Percentage Simple Regret
Saturating Uniform, 30: 0.5005 1.0000 0.9996 1.0000 1.0000 1.0000 1.0000 1.0000 1.0000 1.0000 1.0000 1.0000
Saturating, Fully Continual, Standard: 0.0000 0.5005 0.0002 0.0024 1.0000 0.1116 0.5419 0.8837 0.9986 0.0941 0.9830 0.9963
Saturating, Fully Continual, Balanced: 0.0004 0.9998 0.5005 0.8053 1.0000 0.9894 0.9998 1.0000 1.0000 0.9910 1.0000 1.0000
Saturating, Softmax 3 Stage: 0.0000 0.9976 0.1954 0.5005 1.0000 0.9415 0.9977 1.0000 1.0000 0.9354 1.0000 1.0000
Peaking Uniform, 30: 0.0000 0.0000 0.0000 0.0000 0.5005 0.0000 0.0000 0.0001 0.0224 0.0000 0.0163 0.0147
Peaking, Fully Continual, Standard: 0.0000 0.8889 0.0107 0.0588 1.0000 0.5005 0.9025 0.9955 1.0000 0.4766 0.9988 1.0000
Peaking, Fully Continual, Balanced: 0.0000 0.4591 0.0002 0.0023 1.0000 0.0979 0.5005 0.8616 0.9978 0.0782 0.9773 0.9944
Peaking, Softmax 3 Stage: 0.0000 0.1167 0.0000 0.0000 0.9999 0.0045 0.1389 0.5005 0.9802 0.0036 0.8288 0.9443
Weighted Uniform, 30: 0.0000 0.0014 0.0000 0.0000 0.9778 0.0000 0.0022 0.0199 0.5005 0.0000 0.2181 0.3216
Weighted, Fully Continual, Standard 30: 0.0000 0.9063 0.0091 0.0649 1.0000 0.5244 0.9222 0.9964 1.0000 0.5005 0.9993 1.0000
Weighted, Fully Continual, Balanced 30: 0.0000 0.0171 0.0000 0.0000 0.9838 0.0012 0.0228 0.1718 0.7826 0.0007 0.5005 0.6378
Weighted, Softmax 3 Stage 30: 0.0000 0.0038 0.0000 0.0000 0.9854 0.0000 0.0056 0.0559 0.6793 0.0000 0.3632 0.5005
Saturating Uniform, 30 | Saturating, Fully Continual, Standard | Saturating, Fully Continual, Balanced | Saturating, Softmax 3 Stage | Peaking Uniform, 30 | Peaking, Fully Continual, Standard | Peaking, Fully Continual, Balanced | Peaking, Softmax 3 Stage | Weighted Uniform, 30 | Weighted, Fully Continual, Standard 30 | Weighted, Fully Continual, Balanced 30 | Weighted, Softmax 3 Stage 30

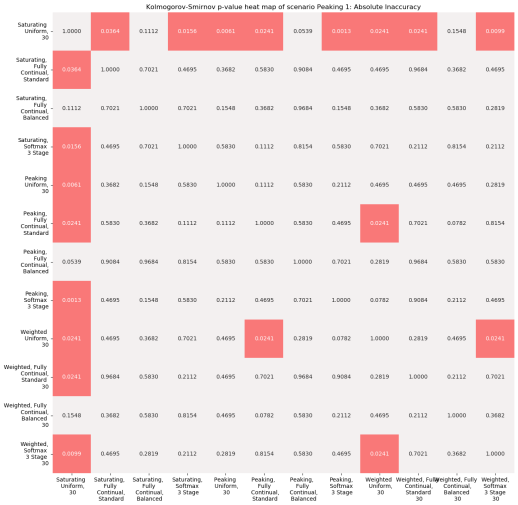
Kolmogorov-Smirnov p-value heat map of scenario Peaking 1: Absolute Inaccuracy
Saturating Uniform, 30: 1.0000 0.0364 0.1112 0.0156 0.0061 0.0241 0.0539 0.0013 0.0241 0.0241 0.1548 0.0099
Saturating, Fully Continual, Standard: 0.0364 1.0000 0.7021 0.4695 0.3682 0.5830 0.9084 0.4695 0.4695 0.9684 0.3682 0.4695
Saturating, Fully Continual, Balanced: 0.1112 0.7021 1.0000 0.7021 0.1548 0.3682 0.9684 0.1548 0.3682 0.5830 0.5830 0.2819
Saturating, Softmax 3 Stage: 0.0156 0.4695 0.7021 1.0000 0.5830 0.1112 0.8154 0.5830 0.7021 0.2112 0.8154 0.2112
Peaking Uniform, 30: 0.0061 0.3682 0.1548 0.5830 1.0000 0.1112 0.5830 0.2112 0.4695 0.4695 0.4695 0.2819
Peaking, Fully Continual, Standard: 0.0241 0.5830 0.3682 0.1112 0.1112 1.0000 0.5830 0.4695 0.0241 0.7021 0.0782 0.8154
Peaking, Fully Continual, Balanced: 0.0539 0.9084 0.9684 0.8154 0.5830 0.5830 1.0000 0.7021 0.2819 0.9684 0.5830 0.5830
Peaking, Softmax 3 Stage: 0.0013 0.4695 0.1548 0.5830 0.2112 0.4695 0.7021 1.0000 0.0782 0.9084 0.2112 0.4695
Weighted Uniform, 30: 0.0241 0.4695 0.3682 0.7021 0.4695 0.0241 0.2819 0.0782 1.0000 0.2819 0.4695 0.0241
Weighted, Fully Continual, Standard 30: 0.0241 0.9684 0.5830 0.2112 0.4695 0.7021 0.9684 0.9084 0.2819 1.0000 0.2112 0.7021
Weighted, Fully Continual, Balanced 30: 0.1548 0.3682 0.5830 0.8154 0.4695 0.0782 0.5830 0.2112 0.4695 0.2112 1.0000 0.3682
Weighted, Softmax 3 Stage 30: 0.0099 0.4695 0.2819 0.2112 0.2819 0.8154 0.5830 0.4695 0.0241 0.7021 0.3682 1.0000
Saturating Uniform, 30 | Saturating, Fully Continual, Standard | Saturating, Fully Continual, Balanced | Saturating, Softmax 3 Stage | Peaking Uniform, 30 | Peaking, Fully Continual, Standard | Peaking, Fully Continual, Balanced | Peaking, Softmax 3 Stage | Weighted Uniform, 30 | Weighted, Fully Continual, Standard 30 | Weighted, Fully Continual, Balanced 30 | Weighted, Softmax 3 Stage 30

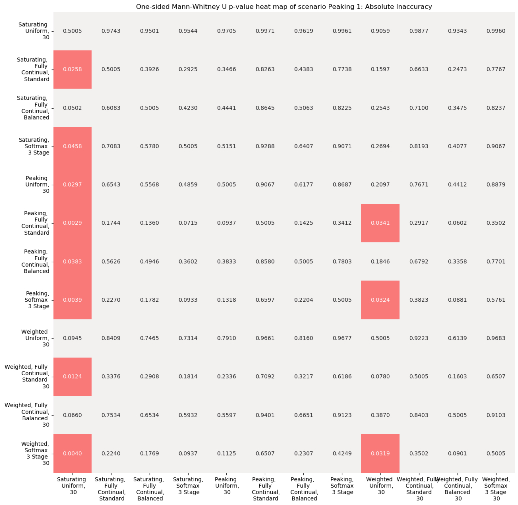
One-sided Mann-Whitney U p-value heat map of scenario Peaking 1: Absolute Inaccuracy
Saturating Uniform, 30: 0.5005 0.9743 0.9501 0.9544 0.9705 0.9971 0.9619 0.9961 0.9059 0.9877 0.9343 0.9960
Saturating, Fully Continual, Standard: 0.0258 0.5005 0.3926 0.2925 0.3466 0.8263 0.4383 0.7738 0.1597 0.6633 0.2473 0.7767
Saturating, Fully Continual, Balanced: 0.0502 0.6083 0.5005 0.4230 0.4441 0.8645 0.5063 0.8225 0.2543 0.7100 0.3475 0.8237
Saturating, Softmax 3 Stage: 0.0458 0.7083 0.5780 0.5005 0.5151 0.9288 0.6407 0.9071 0.2694 0.8193 0.4077 0.9067
Peaking Uniform, 30: 0.0297 0.6543 0.5568 0.4859 0.5005 0.9067 0.6177 0.8687 0.2097 0.7671 0.4412 0.8879
Peaking, Fully Continual, Standard: 0.0029 0.1744 0.1360 0.0715 0.0937 0.5005 0.1425 0.3412 0.0341 0.2917 0.0602 0.3502
Peaking, Fully Continual, Balanced: 0.0383 0.5626 0.4946 0.3602 0.3833 0.8580 0.5005 0.7803 0.1846 0.6792 0.3358 0.7701
Peaking, Softmax 3 Stage: 0.0039 0.2270 0.1782 0.0933 0.1318 0.6597 0.2204 0.5005 0.0324 0.3823 0.0881 0.5761
Weighted Uniform, 30: 0.0945 0.8409 0.7465 0.7314 0.7910 0.9661 0.8160 0.9677 0.5005 0.9223 0.6139 0.9683
Weighted, Fully Continual, Standard 30: 0.0124 0.3376 0.2908 0.1814 0.2336 0.7092 0.3217 0.6186 0.0780 0.5005 0.1603 0.6507
Weighted, Fully Continual, Balanced 30: 0.0660 0.7534 0.6534 0.5932 0.5597 0.9401 0.6651 0.9123 0.3870 0.8403 0.5005 0.9103
Weighted, Softmax 3 Stage 30: 0.0040 0.2240 0.1769 0.0937 0.1125 0.6507 0.2307 0.4249 0.0319 0.3502 0.0901 0.5005
Saturating Uniform, 30 | Saturating, Fully Continual, Standard | Saturating, Fully Continual, Balanced | Saturating, Softmax 3 Stage | Peaking Uniform, 30 | Peaking, Fully Continual, Standard | Peaking, Fully Continual, Balanced | Peaking, Softmax 3 Stage | Weighted Uniform, 30 | Weighted, Fully Continual, Standard 30 | Weighted, Fully Continual, Balanced 30 | Weighted, Softmax 3 Stage 30

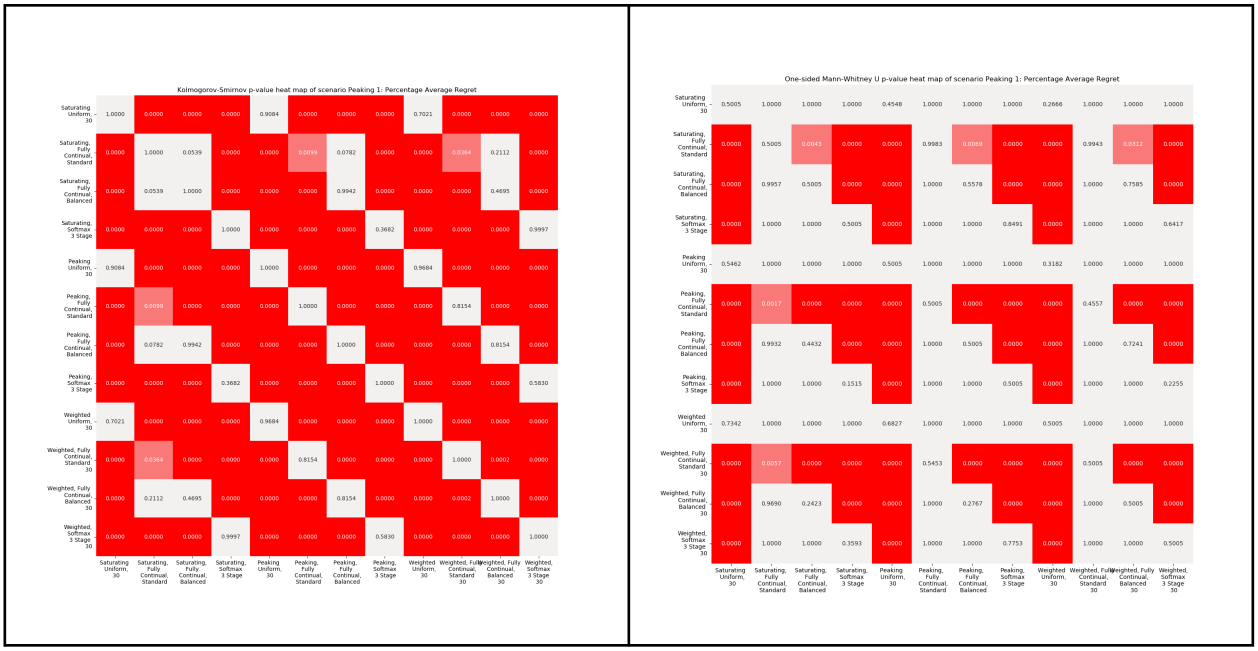

Figure Supplementary.Statistical.Obj2.P1. Kolmogorov–Smirnov (left) and Mann-Whitney U (right) heatmaps of p-values for objective 1, Scenario Peaking 1. These are for the metrics of PSR (top), Absolute Inaccuracy (middle) and PAR (bottom). Cells with a light pink hue represent the test statistic for that comparison would be significant under the threshold p<0.05, cells with a red hue represent the test statistic for that comparison would be significant under the threshold p<0.05 with Bonferri multiple comparison correction.

Scenario Peaking 2

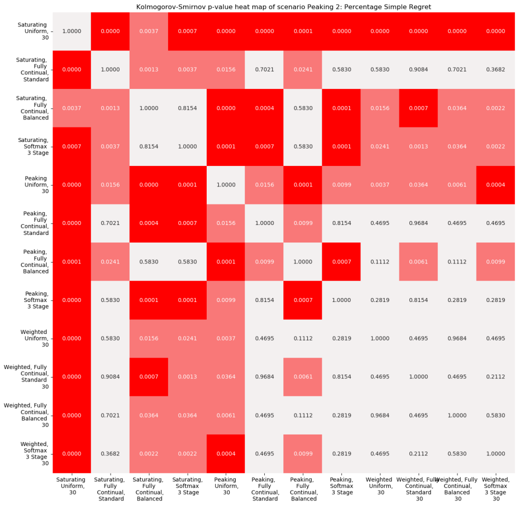
Kolmogorov-Smirnov p-value heat map of scenario Peaking 2: Percentage Simple Regret
Saturating Uniform, 30
Saturating, Fully Continual, Standard
Saturating, Fully Continual, Balanced
Saturating, Softmax 3 Stage
Peaking Uniform, 30
Peaking, Fully Continual, Standard
Peaking, Fully Continual, Balanced
Peaking, Softmax 3 Stage
Weighted Uniform, 30
Weighted, Fully Continual, Standard 30
Weighted, Fully Continual, Balanced 30
Weighted, Softmax 3 Stage 30

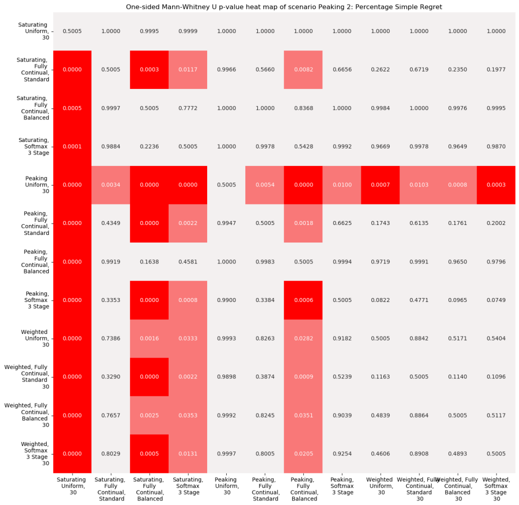
One-sided Mann-Whitney U p-value heat map of scenario Peaking 2: Percentage Simple Regret
Saturating Uniform, 30
Saturating, Fully Continual, Standard
Saturating, Fully Continual, Balanced
Saturating, Softmax 3 Stage
Peaking Uniform, 30
Peaking, Fully Continual, Standard
Peaking, Fully Continual, Balanced
Peaking, Softmax 3 Stage
Weighted Uniform, 30
Weighted, Fully Continual, Standard 30
Weighted, Fully Continual, Balanced 30
Weighted, Softmax 3 Stage 30

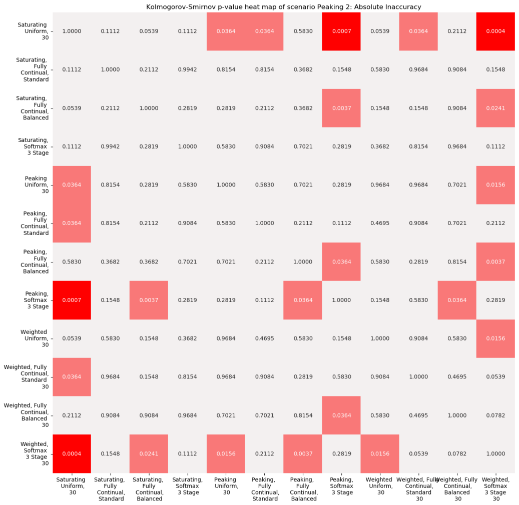
Kolmogorov-Smirnov p-value heat map of scenario Peaking 2: Absolute Inaccuracy
Saturating Uniform, 30
Saturating, Fully Continual, Standard
Saturating, Fully Continual, Balanced
Saturating, Softmax 3 Stage
Peaking Uniform, 30
Peaking, Fully Continual, Standard
Peaking, Fully Continual, Balanced
Peaking, Softmax 3 Stage
Weighted Uniform, 30
Weighted, Fully Continual, Standard 30
Weighted, Fully Continual, Balanced 30
Weighted, Softmax 3 Stage 30

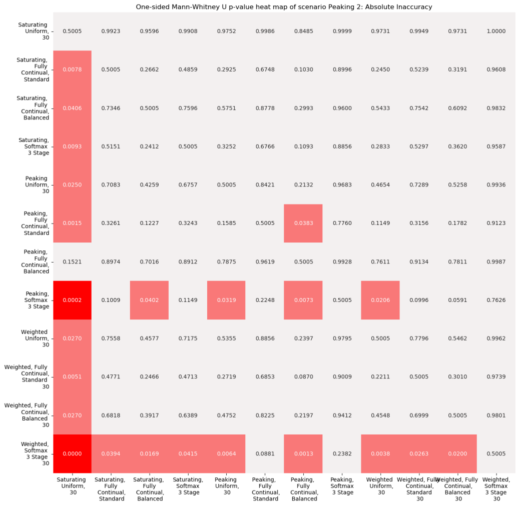
One-sided Mann-Whitney U p-value heat map of scenario Peaking 2: Absolute Inaccuracy
Saturating Uniform, 30
Saturating, Fully Continual, Standard
Saturating, Fully Continual, Balanced
Saturating, Softmax 3 Stage
Peaking Uniform, 30
Peaking, Fully Continual, Standard
Peaking, Fully Continual, Balanced
Peaking, Softmax 3 Stage
Weighted Uniform, 30
Weighted, Fully Continual, Standard 30
Weighted, Fully Continual, Balanced 30
Weighted, Softmax 3 Stage 30

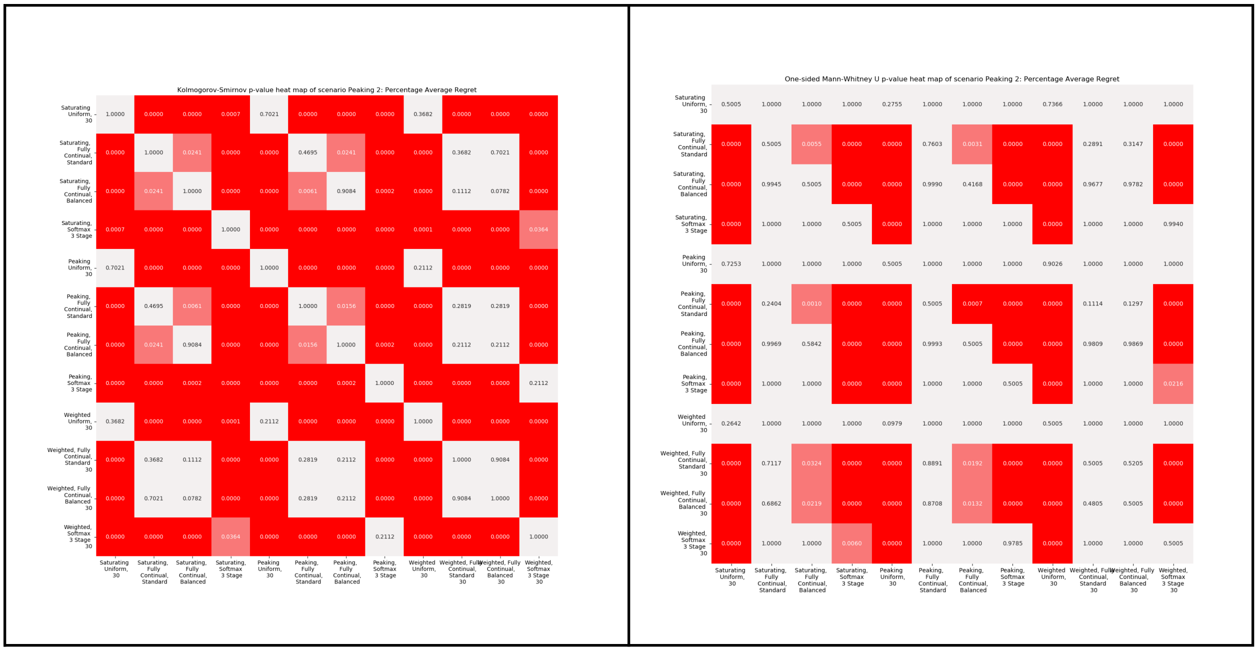

Figure Supplementary.Statistical.Obj2.P2. Kolmogorov–Smirnov (left) and Mann-Whitney U (right) heatmaps of p-values for objective 1, Scenario Peaking 2. These are for the metrics of PSR (top), Absolute Inaccuracy (middle) and PAR (bottom). Cells with a light pink hue represent the test statistic for that comparison would be significant under the threshold p<0.05, cells with a red hue represent the test statistic for that comparison would be significant under the threshold p<0.05 with Bonferri multiple comparison correction.

Scenario Peaking 3

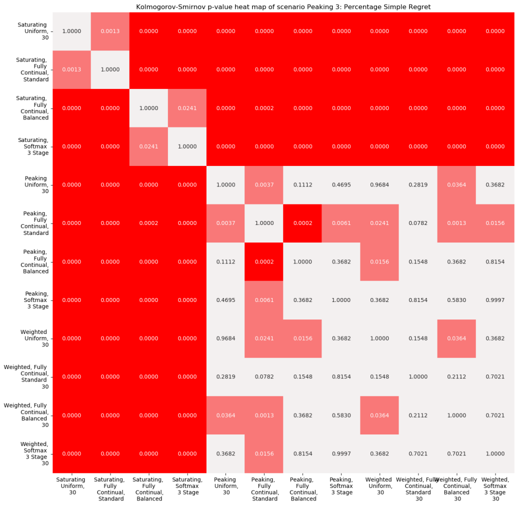
Kolmogorov-Smirnov p-value heat map of scenario Peaking 3: Percentage Simple Regret
Saturating Uniform, 30
Saturating, Fully Continual, Standard
Saturating, Fully Continual, Balanced
Saturating, Softmax 3 Stage
Peaking Uniform, 30
Peaking, Fully Continual, Standard
Peaking, Fully Continual, Balanced
Peaking, Softmax 3 Stage
Weighted Uniform, 30
Weighted, Fully Continual, Standard 30
Weighted, Fully Continual, Balanced 30
Weighted, Softmax 3 Stage 30

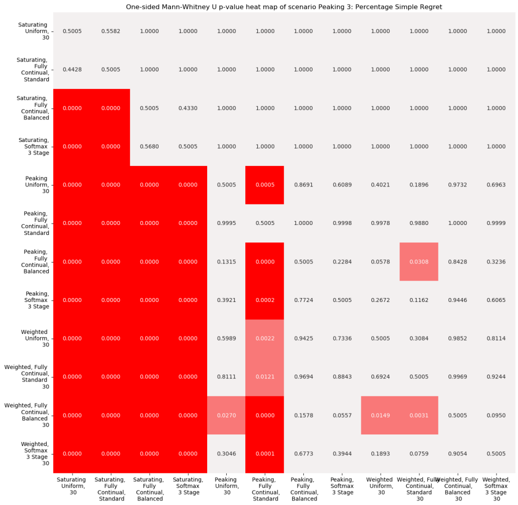
One-sided Mann-Whitney U p-value heat map of scenario Peaking 3: Percentage Simple Regret
Saturating Uniform, 30
Saturating, Fully Continual, Standard
Saturating, Fully Continual, Balanced
Saturating, Softmax 3 Stage
Peaking Uniform, 30
Peaking, Fully Continual, Standard
Peaking, Fully Continual, Balanced
Peaking, Softmax 3 Stage
Weighted Uniform, 30
Weighted, Fully Continual, Standard 30
Weighted, Fully Continual, Balanced 30
Weighted, Softmax 3 Stage 30

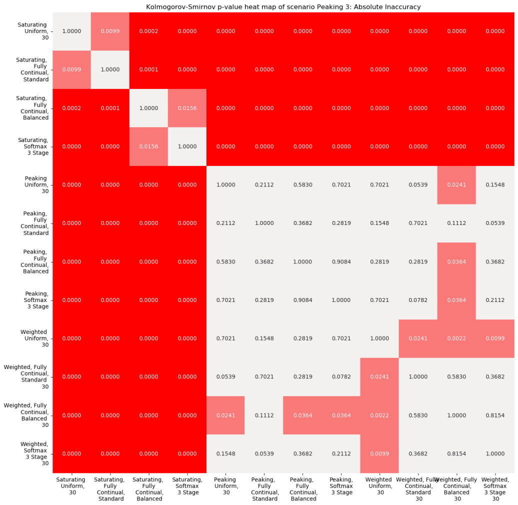
Kolmogorov-Smirnov p-value heat map of scenario Peaking 3: Absolute Inaccuracy
Saturating Uniform, 30
Saturating, Fully Continual, Standard
Saturating, Fully Continual, Balanced
Saturating, Softmax 3 Stage
Peaking Uniform, 30
Peaking, Fully Continual, Standard
Peaking, Fully Continual, Balanced
Peaking, Softmax 3 Stage
Weighted Uniform, 30
Weighted, Fully Continual, Standard 30
Weighted, Fully Continual, Balanced 30
Weighted, Softmax 3 Stage 30

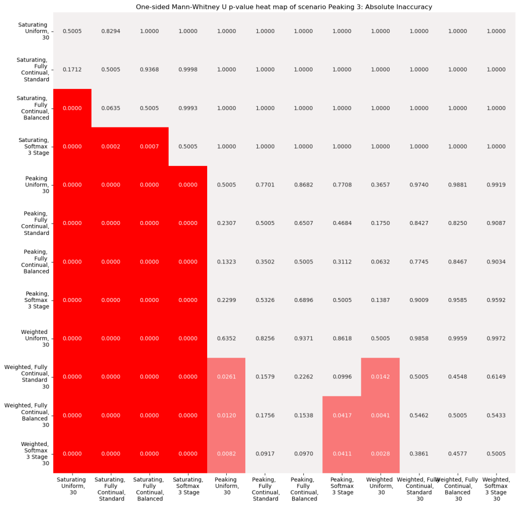
One-sided Mann-Whitney U p-value heat map of scenario Peaking 3: Absolute Inaccuracy
Saturating Uniform, 30
Saturating, Fully Continual, Standard
Saturating, Fully Continual, Balanced
Saturating, Softmax 3 Stage
Peaking Uniform, 30
Peaking, Fully Continual, Standard
Peaking, Fully Continual, Balanced
Peaking, Softmax 3 Stage
Weighted Uniform, 30
Weighted, Fully Continual, Standard 30
Weighted, Fully Continual, Balanced 30
Weighted, Softmax 3 Stage 30

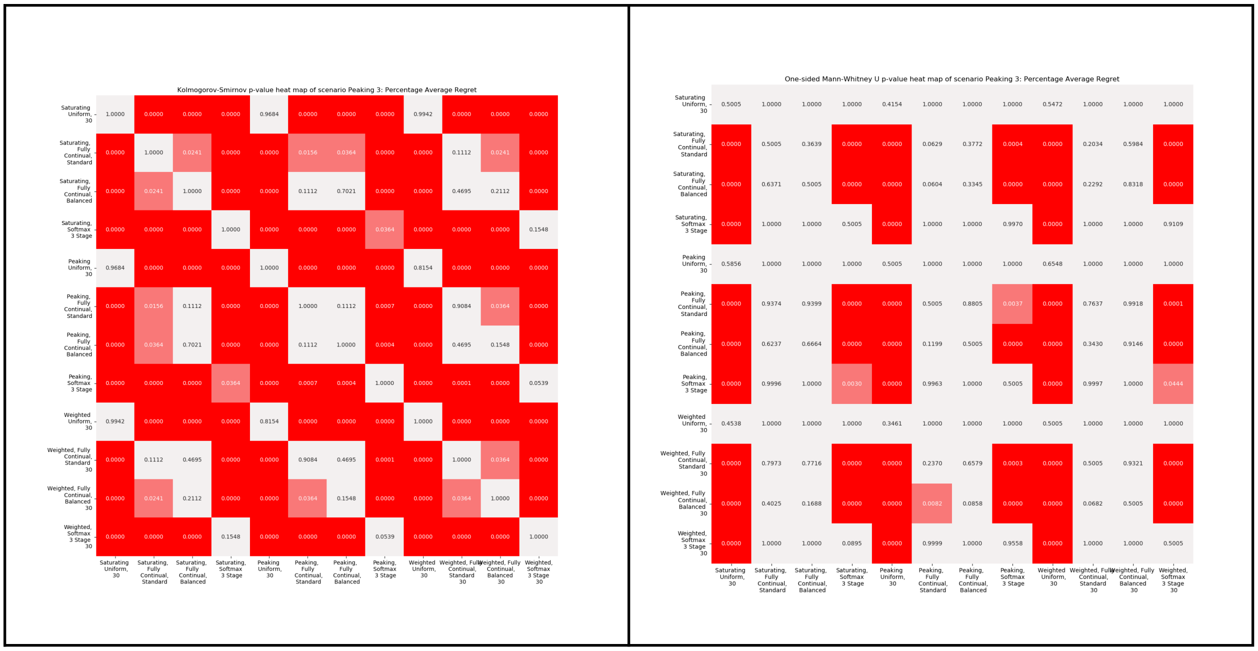

Figure Supplementary.Statistical.Obj2.P3. Kolmogorov–Smirnov (left) and Mann-Whitney U (right) heatmaps of p-values for objective 1, Scenario Peaking 3. These are for the metrics of PSR (top), Absolute Inaccuracy (middle) and PAR (bottom). Cells with a light pink hue represent the test statistic for that comparison would be significant under the threshold p<0.05, cells with a red hue represent the test statistic for that comparison would be significant under the threshold p<0.05 with Bonferri multiple comparison correction.

Scenario Peaking 4

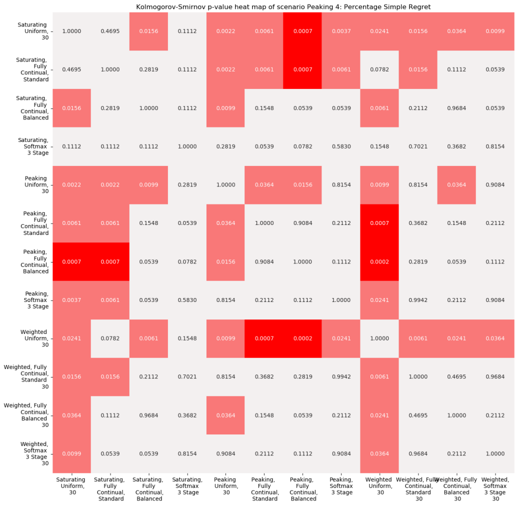
Kolmogorov-Smirnov p-value heat map of scenario Peaking 4: Percentage Simple Regret
Saturating Uniform, 30
Saturating, Fully Continual, Standard
Saturating, Fully Continual, Balanced
Saturating, Softmax 3 Stage
Peaking Uniform, 30
Peaking, Fully Continual, Standard
Peaking, Fully Continual, Balanced
Peaking, Softmax 3 Stage
Weighted Uniform, 30
Weighted, Fully Continual, Standard 30
Weighted, Fully Continual, Balanced 30
Weighted, Softmax 3 Stage 30

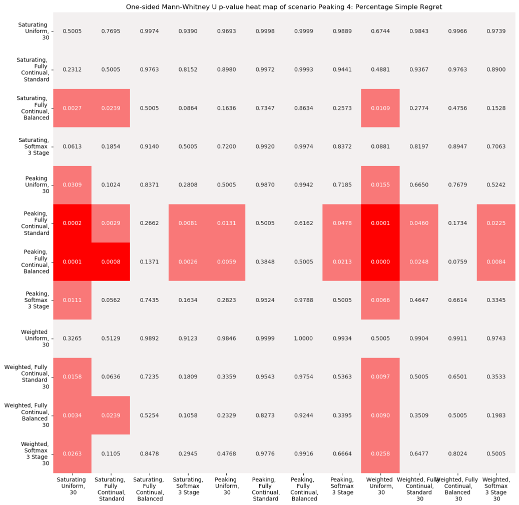
One-sided Mann-Whitney U p-value heat map of scenario Peaking 4: Percentage Simple Regret
Saturating Uniform, 30
Saturating, Fully Continual, Standard
Saturating, Fully Continual, Balanced
Saturating, Softmax 3 Stage
Peaking Uniform, 30
Peaking, Fully Continual, Standard
Peaking, Fully Continual, Balanced
Peaking, Softmax 3 Stage
Weighted Uniform, 30
Weighted, Fully Continual, Standard 30
Weighted, Fully Continual, Balanced 30
Weighted, Softmax 3 Stage 30

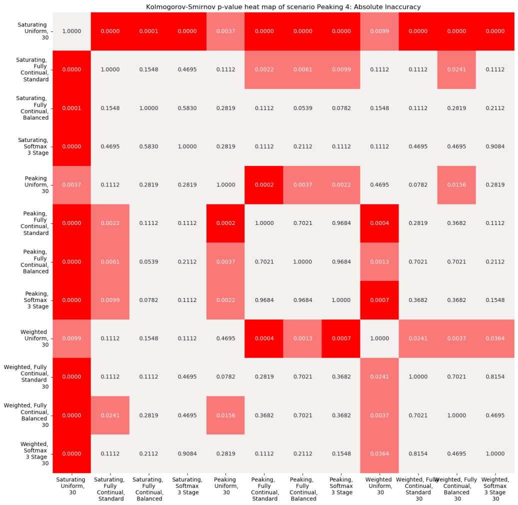
Kolmogorov-Smirnov p-value heat map of scenario Peaking 4: Absolute Inaccuracy
Saturating Uniform, 30
Saturating, Fully Continual, Standard
Saturating, Fully Continual, Balanced
Saturating, Softmax 3 Stage
Peaking Uniform, 30
Peaking, Fully Continual, Standard
Peaking, Fully Continual, Balanced
Peaking, Softmax 3 Stage
Weighted Uniform, 30
Weighted, Fully Continual, Standard 30
Weighted, Fully Continual, Balanced 30
Weighted, Softmax 3 Stage 30

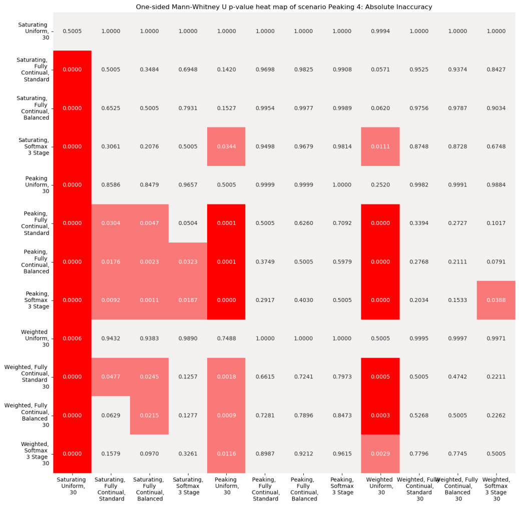
One-sided Mann-Whitney U p-value heat map of scenario Peaking 4: Absolute Inaccuracy
Saturating Uniform, 30
Saturating, Fully Continual, Standard
Saturating, Fully Continual, Balanced
Saturating, Softmax 3 Stage
Peaking Uniform, 30
Peaking, Fully Continual, Standard
Peaking, Fully Continual, Balanced
Peaking, Softmax 3 Stage
Weighted Uniform, 30
Weighted, Fully Continual, Standard 30
Weighted, Fully Continual, Balanced 30
Weighted, Softmax 3 Stage 30

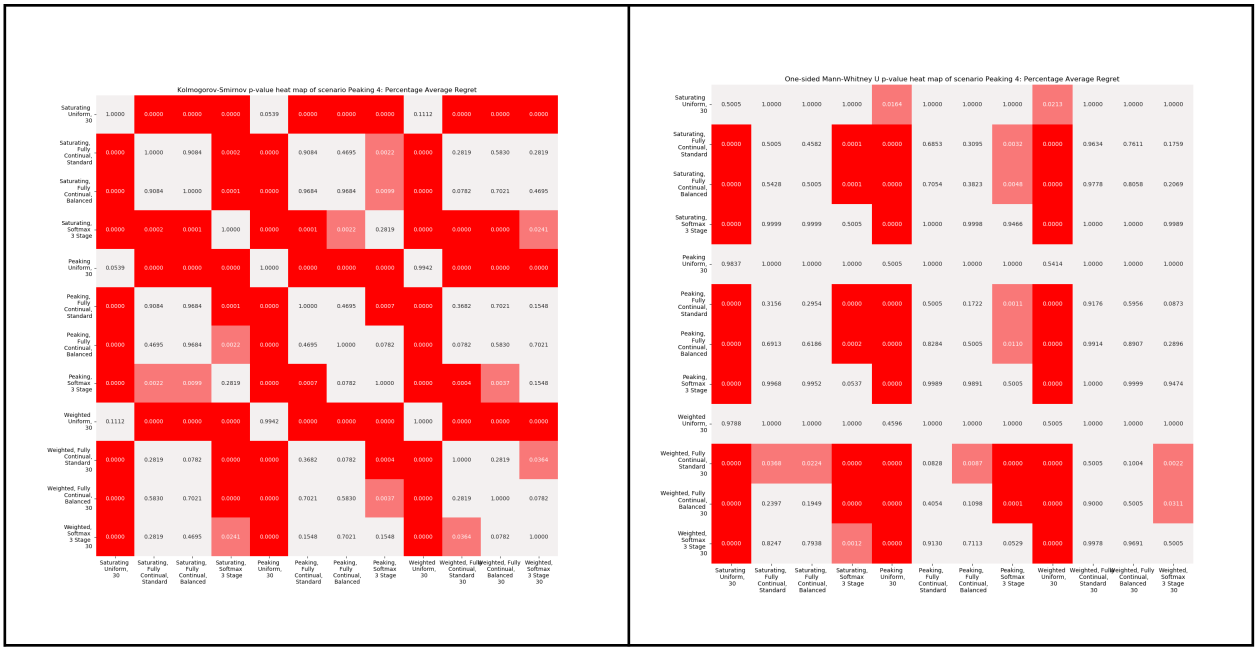

Figure Supplementary.Statistical.Obj2.P4. Kolmogorov–Smirnov (left) and Mann-Whitney U (right) heatmaps of p-values for objective 1, Scenario Peaking 4. These are for the metrics of PSR (top), Absolute Inaccuracy (middle) and PAR (bottom). Cells with a light pink hue represent the test statistic for that comparison would be significant under the threshold p<0.05, cells with a red hue represent the test statistic for that comparison would be significant under the threshold p<0.05 with Bonferri multiple comparison correction.

Scenario Peaking 5

Kolmogorov-Smirnov p-value heat map of scenario Peaking 5: Percentage Simple Regret

	Saturating Uniform, 30	Saturating, Fully Continual, Standard	Saturating, Fully Continual, Balanced	Saturating, Softmax 3 Stage	Peaking Uniform, 30	Peaking, Fully Continual, Standard	Peaking, Fully Continual, Balanced	Peaking, Softmax 3 Stage	Weighted Uniform, 30	Weighted, Fully Continual, Standard 30	Weighted, Fully Continual, Balanced 30	Weighted, Softmax 3 Stage 30
Saturating Uniform, 30	1.0000	0.0539	0.7021	0.2112	0.0539	0.0013	0.2112	0.0007	0.1548	0.0007	0.3682	0.0539
Saturating, Fully Continual, Standard	0.0539	1.0000	0.2819	0.3682	0.0241	0.0156	0.4695	0.0013	0.0782	0.0539	0.5830	0.1112
Saturating, Fully Continual, Balanced	0.7021	0.2819	1.0000	0.9084	0.0782	0.0241	0.7021	0.0539	0.5830	0.0241	0.9084	0.5830
Saturating, Softmax 3 Stage	0.2112	0.3682	0.9084	1.0000	0.3682	0.0539	0.8154	0.2112	0.9684	0.0241	0.5830	0.7021
Peaking Uniform, 30	0.0539	0.0241	0.0782	0.3682	1.0000	0.0156	0.1112	0.0364	0.5830	0.0037	0.0539	0.1548
Peaking, Fully Continual, Standard	0.0013	0.0156	0.0241	0.0539	0.0156	1.0000	0.1112	0.7021	0.0539	0.7021	0.0539	0.4695
Peaking, Fully Continual, Balanced	0.2112	0.4695	0.7021	0.8154	0.1112	0.1112	1.0000	0.2112	0.2819	0.0782	0.9684	0.9684
Peaking, Softmax 3 Stage	0.0007	0.0013	0.0539	0.2112	0.0364	0.7021	0.2112	1.0000	0.1548	0.0539	0.0364	0.3682
Weighted Uniform, 30	0.1548	0.0782	0.5830	0.9684	0.5830	0.0539	0.2819	0.1548	1.0000	0.0156	0.1548	0.4695
Weighted, Fully Continual, Standard 30	0.0007	0.0539	0.0241	0.0241	0.0037	0.7021	0.0782	0.0539	0.0156	1.0000	0.0241	0.2819
Weighted, Fully Continual, Balanced 30	0.3682	0.5830	0.9084	0.5830	0.0539	0.0539	0.9684	0.0364	0.1548	0.0241	1.0000	0.5830
Weighted, Softmax 3 Stage 30	0.0539	0.1112	0.5830	0.7021	0.1548	0.4695	0.9684	0.3682	0.4695	0.2819	0.5830	1.0000

One-sided Mann-Whitney U p-value heat map of scenario Peaking 5: Percentage Simple Regret

	Saturating Uniform, 30	Saturating, Fully Continual, Standard	Saturating, Fully Continual, Balanced	Saturating, Softmax 3 Stage	Peaking Uniform, 30	Peaking, Fully Continual, Standard	Peaking, Fully Continual, Balanced	Peaking, Softmax 3 Stage	Weighted Uniform, 30	Weighted, Fully Continual, Standard 30	Weighted, Fully Continual, Balanced 30	Weighted, Softmax 3 Stage 30
Saturating Uniform, 30	0.5005	0.9024	0.8597	0.9234	0.8624	0.9992	0.9360	0.9981	0.9512	0.9946	0.8830	0.9689
Saturating, Fully Continual, Standard	0.0980	0.5005	0.4354	0.6530	0.5646	0.9921	0.6325	0.9885	0.7029	0.9598	0.4335	0.8001
Saturating, Fully Continual, Balanced	0.1409	0.5655	0.5005	0.6642	0.5544	0.9859	0.6526	0.9592	0.7573	0.9224	0.4640	0.7130
Saturating, Softmax 3 Stage	0.0769	0.3479	0.3367	0.5005	0.4393	0.9296	0.4790	0.9020	0.5880	0.7612	0.2946	0.5355
Peaking Uniform, 30	0.1381	0.4364	0.4465	0.5617	0.5005	0.9437	0.5573	0.8937	0.6731	0.8165	0.4030	0.5665
Peaking, Fully Continual, Standard	0.0008	0.0080	0.0142	0.0707	0.0566	0.5005	0.0358	0.4995	0.1102	0.1661	0.0088	0.0691
Peaking, Fully Continual, Balanced	0.0643	0.3684	0.3483	0.5219	0.4436	0.9644	0.5005	0.9495	0.6102	0.8323	0.3022	0.5894
Peaking, Softmax 3 Stage	0.0020	0.0115	0.0410	0.0984	0.1067	0.5015	0.0508	0.5005	0.1826	0.1286	0.0214	0.0753
Weighted Uniform, 30	0.0490	0.2980	0.2435	0.4130	0.3277	0.8903	0.3908	0.8180	0.5005	0.7016	0.2295	0.4249
Weighted, Fully Continual, Standard 30	0.0055	0.0404	0.0779	0.2396	0.1842	0.8345	0.1683	0.8719	0.2992	0.5005	0.0522	0.2992
Weighted, Fully Continual, Balanced 30	0.1175	0.5674	0.5370	0.7063	0.5980	0.9913	0.6987	0.9787	0.7712	0.9481	0.5005	0.7779
Weighted, Softmax 3 Stage 30	0.0313	0.2006	0.2878	0.4654	0.4345	0.9312	0.4115	0.9250	0.5761	0.7017	0.2229	0.5005

Kolmogorov-Smirnov p-value heat map of scenario Peaking 5: Absolute Inaccuracy

	Saturating Uniform, 30	Saturating, Fully Continual, Standard	Saturating, Fully Continual, Balanced	Saturating, Softmax 3 Stage	Peaking Uniform, 30	Peaking, Fully Continual, Standard	Peaking, Fully Continual, Balanced	Peaking, Softmax 3 Stage	Weighted Uniform, 30	Weighted, Fully Continual, Standard 30	Weighted, Fully Continual, Balanced 30	Weighted, Softmax 3 Stage 30
Saturating Uniform, 30	1.0000	0.5830	0.5830	0.8154	0.4695	0.3682	0.9997	0.7021	0.7021	0.2112	0.8154	0.0782
Saturating, Fully Continual, Standard	0.5830	1.0000	0.9684	0.2819	0.0364	0.2112	0.4695	0.7021	0.4695	0.7021	0.2819	0.3682
Saturating, Fully Continual, Balanced	0.5830	0.9684	1.0000	0.7021	0.1548	0.5830	0.5830	0.7021	0.5830	0.7021	0.3682	0.7021
Saturating, Softmax 3 Stage	0.8154	0.2819	0.7021	1.0000	0.1112	0.5830	0.9084	0.8154	0.8154	0.7021	0.8154	0.2112
Peaking Uniform, 30	0.4695	0.0364	0.1548	0.1112	1.0000	0.0241	0.3682	0.1548	0.1548	0.0156	0.1548	0.0241
Peaking, Fully Continual, Standard	0.3682	0.2112	0.5830	0.5830	0.0241	1.0000	0.3682	0.5830	0.7021	0.4695	0.8154	0.1548
Peaking, Fully Continual, Balanced	0.9997	0.4695	0.5830	0.9084	0.3682	0.3682	1.0000	0.7021	0.8154	0.2112	0.7021	0.0782
Peaking, Softmax 3 Stage	0.7021	0.7021	0.7021	0.8154	0.1548	0.5830	0.7021	1.0000	0.9997	0.5830	0.4695	0.4695
Weighted Uniform, 30	0.7021	0.4695	0.5830	0.8154	0.1548	0.7021	0.8154	0.9997	1.0000	0.4695	0.4695	0.3682
Weighted, Fully Continual, Standard 30	0.2112	0.7021	0.7021	0.7021	0.0156	0.4695	0.2112	0.5830	0.4695	1.0000	0.2112	0.5830
Weighted, Fully Continual, Balanced 30	0.8154	0.2819	0.3682	0.8154	0.1548	0.8154	0.7021	0.4695	0.4695	0.2112	1.0000	0.0364
Weighted, Softmax 3 Stage 30	0.0782	0.3682	0.7021	0.2112	0.0241	0.1548	0.0782	0.4695	0.3682	0.5830	0.0364	1.0000

One-sided Mann-Whitney U p-value heat map of scenario Peaking 5: Absolute Inaccuracy

	Saturating Uniform, 30	Saturating, Fully Continual, Standard	Saturating, Fully Continual, Balanced	Saturating, Softmax 3 Stage	Peaking Uniform, 30	Peaking, Fully Continual, Standard	Peaking, Fully Continual, Balanced	Peaking, Softmax 3 Stage	Weighted Uniform, 30	Weighted, Fully Continual, Standard 30	Weighted, Fully Continual, Balanced 30	Weighted, Softmax 3 Stage 30
Saturating Uniform, 30	0.5005	0.8682	0.8288	0.4742	0.0949	0.6214	0.4345	0.6783	0.5932	0.8586	0.4548	0.9775
Saturating, Fully Continual, Standard	0.1323	0.5005	0.4606	0.1098	0.0081	0.2111	0.1052	0.2233	0.1651	0.4654	0.1130	0.8134
Saturating, Fully Continual, Balanced	0.1719	0.5404	0.5005	0.1344	0.0190	0.2146	0.1568	0.2984	0.2233	0.4800	0.1425	0.8607
Saturating, Softmax 3 Stage	0.5268	0.8907	0.8661	0.5005	0.1323	0.6543	0.5063	0.7265	0.6534	0.8827	0.4976	0.9855
Peaking Uniform, 30	0.9055	0.9919	0.9812	0.8682	0.5005	0.9324	0.8842	0.9528	0.9272	0.9865	0.8813	0.9986
Peaking, Fully Continual, Standard	0.3796	0.7896	0.7861	0.3466	0.0679	0.5005	0.3376	0.6008	0.5209	0.8263	0.3466	0.9711
Peaking, Fully Continual, Balanced	0.5665	0.8952	0.8438	0.4946	0.1163	0.6633	0.5005	0.7100	0.6195	0.8803	0.5190	0.9794
Peaking, Softmax 3 Stage	0.3226	0.7774	0.7024	0.2743	0.0475	0.4002	0.2908	0.5005	0.4077	0.7249	0.2654	0.9389
Weighted Uniform, 30	0.4077	0.8355	0.7774	0.3475	0.0732	0.4800	0.3814	0.5932	0.5005	0.7861	0.3385	0.9608
Weighted, Fully Continual, Standard 30	0.1420	0.5355	0.5209	0.1178	0.0136	0.1744	0.1202	0.2759	0.2146	0.5005	0.1111	0.8634
Weighted, Fully Continual, Balanced 30	0.5462	0.8875	0.8580	0.5034	0.1192	0.6543	0.4820	0.7354	0.6624	0.8893	0.5005	0.9853
Weighted, Softmax 3 Stage 30	0.0226	0.1872	0.1398	0.0146	0.0014	0.0291	0.0207	0.0614	0.0394	0.1371	0.0148	0.5005

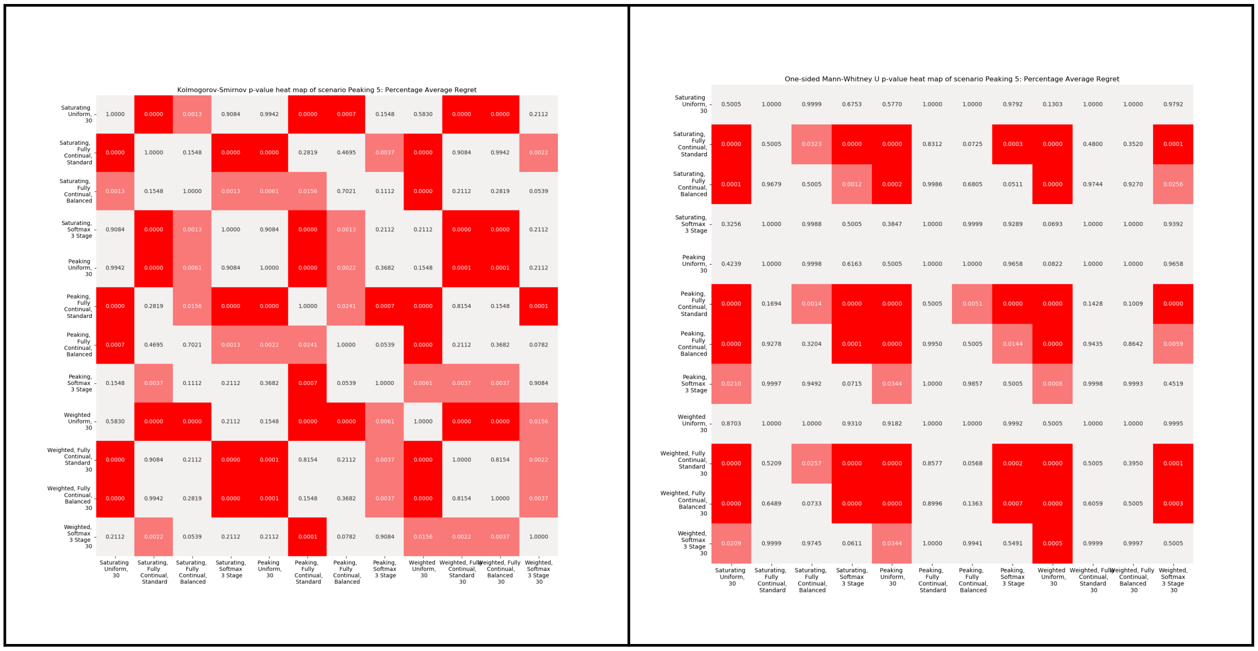

Figure Supplementary.Statistical.Obj2.S5. Kolmogorov–Smirnov (left) and Mann-Whitney U (right) heatmaps of p-values for objective 1, Scenario Peaking 5. These are for the metrics of PSR (top), Absolute Inaccuracy (middle) and PAR (bottom). Cells with a light pink hue represent the test statistic for that comparison would be significant under the threshold $p<0.05$, cells with a red hue represent the test statistic for that comparison would be significant under the threshold $p<0.05$ with Bonferri multiple comparison correction.

Scenario Other 1

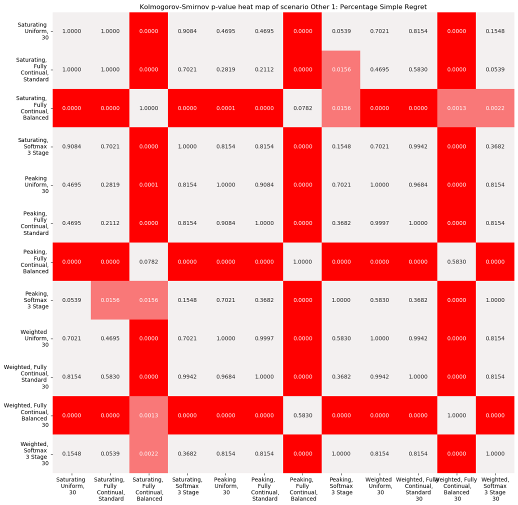
Kolmogorov-Smirnov p-value heat map of scenario Other 1: Percentage Simple Regret
Saturating Uniform, 30: 1.0000 1.0000 0.0000 0.9084 0.4695 0.4695 0.0000 0.0539 0.7021 0.8154 0.0000 0.1548
Saturating, Fully Continual, Standard: 1.0000 1.0000 0.0000 0.7021 0.2819 0.2112 0.0000 0.0156 0.4695 0.5830 0.0000 0.0539
Saturating, Fully Continual, Balanced: 0.0000 0.0000 1.0000 0.0000 0.0001 0.0000 0.0782 0.0156 0.0000 0.0000 0.0013 0.0022
Saturating, Softmax 3 Stage: 0.9084 0.7021 0.0000 1.0000 0.8154 0.8154 0.0000 0.1548 0.7021 0.9942 0.0000 0.3682
Peaking Uniform, 30: 0.4695 0.2819 0.0001 0.8154 1.0000 0.9084 0.0000 0.7021 1.0000 0.9684 0.0000 0.8154
Peaking, Fully Continual, Standard: 0.4695 0.2112 0.0000 0.8154 0.9084 1.0000 0.0000 0.3682 0.9997 1.0000 0.0000 0.8154
Peaking, Fully Continual, Balanced: 0.0000 0.0000 0.0782 0.0000 0.0000 0.0000 1.0000 0.0000 0.0000 0.0000 0.5830 0.0000
Peaking, Softmax 3 Stage: 0.0539 0.0156 0.0156 0.1548 0.7021 0.3682 0.0000 1.0000 0.5830 0.3682 0.0000 1.0000
Weighted Uniform, 30: 0.7021 0.4695 0.0000 0.7021 1.0000 0.9997 0.0000 0.5830 1.0000 0.9942 0.0000 0.8154
Weighted, Fully Continual, Standard 30: 0.8154 0.5830 0.0000 0.9942 0.9684 1.0000 0.0000 0.3682 0.9942 1.0000 0.0000 0.8154
Weighted, Fully Continual, Balanced 30: 0.0000 0.0000 0.0013 0.0000 0.0000 0.0000 0.5830 0.0000 0.0000 0.0000 1.0000 0.0000
Weighted, Softmax 3 Stage 30: 0.1548 0.0539 0.0022 0.3682 0.8154 0.8154 0.0000 1.0000 0.8154 0.8154 0.0000 1.0000
Saturating Uniform, 30
Saturating, Fully Continual, Standard
Saturating, Fully Continual, Balanced
Saturating, Softmax 3 Stage
Peaking Uniform, 30
Peaking, Fully Continual, Standard
Peaking, Fully Continual, Balanced
Peaking, Softmax 3 Stage
Weighted Uniform, 30
Weighted, Fully Continual, Standard 30
Weighted, Fully Continual, Balanced 30
Weighted, Softmax 3 Stage 30

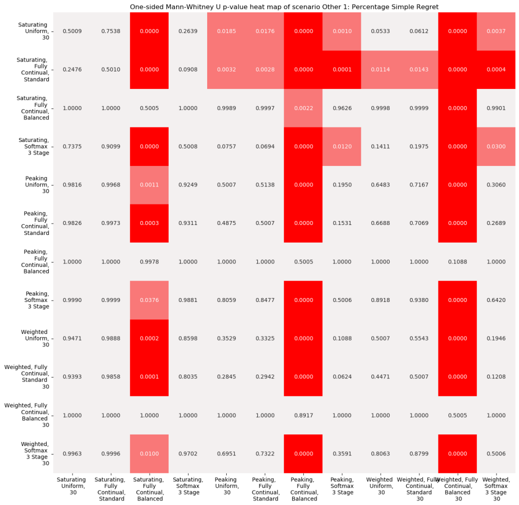
One-sided Mann-Whitney U p-value heat map of scenario Other 1: Percentage Simple Regret
Saturating Uniform, 30: 0.5009 0.7538 0.0000 0.2639 0.0185 0.0176 0.0000 0.0010 0.0533 0.0612 0.0000 0.0037
Saturating, Fully Continual, Standard: 0.2476 0.5010 0.0000 0.0908 0.0032 0.0028 0.0000 0.0001 0.0114 0.0143 0.0000 0.0004
Saturating, Fully Continual, Balanced: 1.0000 1.0000 0.5005 1.0000 0.9989 0.9997 0.0022 0.9626 0.9998 0.9999 0.0000 0.9901
Saturating, Softmax 3 Stage: 0.7375 0.9099 0.0000 0.5008 0.0757 0.0694 0.0000 0.0120 0.1411 0.1975 0.0000 0.0300
Peaking Uniform, 30: 0.9816 0.9968 0.0011 0.9249 0.5007 0.5138 0.0000 0.1950 0.6483 0.7167 0.0000 0.3060
Peaking, Fully Continual, Standard: 0.9826 0.9973 0.0003 0.9311 0.4875 0.5007 0.0000 0.1531 0.6688 0.7069 0.0000 0.2689
Peaking, Fully Continual, Balanced: 1.0000 1.0000 0.9978 1.0000 1.0000 1.0000 0.5005 1.0000 1.0000 1.0000 0.1088 1.0000
Peaking, Softmax 3 Stage: 0.9990 0.9999 0.0376 0.9881 0.8059 0.8477 0.0000 0.5006 0.8918 0.9380 0.0000 0.6420
Weighted Uniform, 30: 0.9471 0.9888 0.0002 0.8598 0.3529 0.3325 0.0000 0.1088 0.5007 0.5543 0.0000 0.1946
Weighted, Fully Continual, Standard 30: 0.9393 0.9858 0.0001 0.8035 0.2845 0.2942 0.0000 0.0624 0.4471 0.5007 0.0000 0.1208
Weighted, Fully Continual, Balanced 30: 1.0000 1.0000 1.0000 1.0000 1.0000 1.0000 0.8917 1.0000 1.0000 1.0000 0.5005 1.0000
Weighted, Softmax 3 Stage 30: 0.9963 0.9996 0.0100 0.9702 0.6951 0.7322 0.0000 0.3591 0.8063 0.8799 0.0000 0.5006
Saturating Uniform, 30
Saturating, Fully Continual, Standard
Saturating, Fully Continual, Balanced
Saturating, Softmax 3 Stage
Peaking Uniform, 30
Peaking, Fully Continual, Standard
Peaking, Fully Continual, Balanced
Peaking, Softmax 3 Stage
Weighted Uniform, 30
Weighted, Fully Continual, Standard 30
Weighted, Fully Continual, Balanced 30
Weighted, Softmax 3 Stage 30

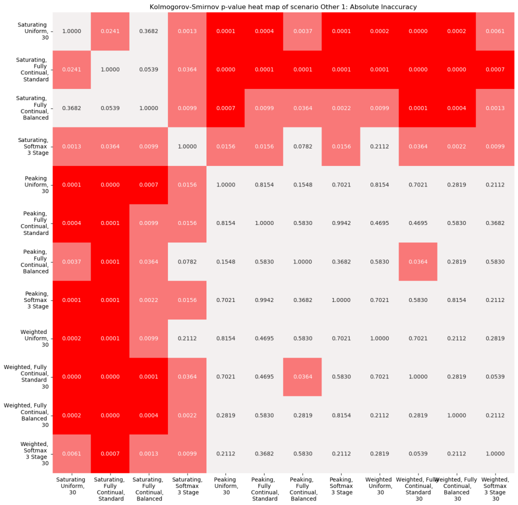
Kolmogorov-Smirnov p-value heat map of scenario Other 1: Absolute Inaccuracy
Saturating Uniform, 30: 1.0000 0.0241 0.3682 0.0013 0.0001 0.0004 0.0037 0.0001 0.0002 0.0000 0.0002 0.0061
Saturating, Fully Continual, Standard: 0.0241 1.0000 0.0539 0.0364 0.0000 0.0001 0.0001 0.0001 0.0001 0.0000 0.0000 0.0007
Saturating, Fully Continual, Balanced: 0.3682 0.0539 1.0000 0.0099 0.0007 0.0099 0.0364 0.0022 0.0099 0.0001 0.0004 0.0013
Saturating, Softmax 3 Stage: 0.0013 0.0364 0.0099 1.0000 0.0156 0.0156 0.0782 0.0156 0.2112 0.0364 0.0022 0.0099
Peaking Uniform, 30: 0.0001 0.0000 0.0007 0.0156 1.0000 0.8154 0.1548 0.7021 0.8154 0.7021 0.2819 0.2112
Peaking, Fully Continual, Standard: 0.0004 0.0001 0.0099 0.0156 0.8154 1.0000 0.5830 0.9942 0.4695 0.4695 0.5830 0.3682
Peaking, Fully Continual, Balanced: 0.0037 0.0001 0.0364 0.0782 0.1548 0.5830 1.0000 0.3682 0.5830 0.0364 0.2819 0.5830
Peaking, Softmax 3 Stage: 0.0001 0.0001 0.0022 0.0156 0.7021 0.9942 0.3682 1.0000 0.7021 0.5830 0.8154 0.2112
Weighted Uniform, 30: 0.0002 0.0001 0.0099 0.2112 0.8154 0.4695 0.5830 0.7021 1.0000 0.7021 0.2112 0.2819
Weighted, Fully Continual, Standard 30: 0.0000 0.0000 0.0001 0.0364 0.7021 0.4695 0.0364 0.5830 0.7021 1.0000 0.2819 0.0539
Weighted, Fully Continual, Balanced 30: 0.0002 0.0000 0.0004 0.0022 0.2819 0.5830 0.2819 0.8154 0.2112 0.2819 1.0000 0.2112
Weighted, Softmax 3 Stage 30: 0.0061 0.0007 0.0013 0.0099 0.2112 0.3682 0.5830 0.2112 0.2819 0.0539 0.2112 1.0000
Saturating Uniform, 30
Saturating, Fully Continual, Standard
Saturating, Fully Continual, Balanced
Saturating, Softmax 3 Stage
Peaking Uniform, 30
Peaking, Fully Continual, Standard
Peaking, Fully Continual, Balanced
Peaking, Softmax 3 Stage
Weighted Uniform, 30
Weighted, Fully Continual, Standard 30
Weighted, Fully Continual, Balanced 30
Weighted, Softmax 3 Stage 30

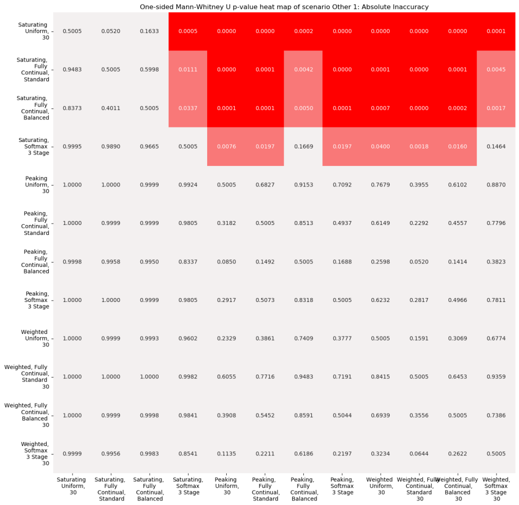
One-sided Mann-Whitney U p-value heat map of scenario Other 1: Absolute Inaccuracy
Saturating Uniform, 30: 0.5005 0.0520 0.1633 0.0005 0.0000 0.0000 0.0002 0.0000 0.0000 0.0000 0.0000 0.0001
Saturating, Fully Continual, Standard: 0.9483 0.5005 0.5998 0.0111 0.0000 0.0001 0.0042 0.0000 0.0001 0.0000 0.0001 0.0045
Saturating, Fully Continual, Balanced: 0.8373 0.4011 0.5005 0.0337 0.0001 0.0001 0.0050 0.0001 0.0007 0.0000 0.0002 0.0017
Saturating, Softmax 3 Stage: 0.9995 0.9890 0.9665 0.5005 0.0076 0.0197 0.1669 0.0197 0.0400 0.0018 0.0160 0.1464
Peaking Uniform, 30: 1.0000 1.0000 0.9999 0.9924 0.5005 0.6827 0.9153 0.7092 0.7679 0.3955 0.6102 0.8870
Peaking, Fully Continual, Standard: 1.0000 0.9999 0.9999 0.9805 0.3182 0.5005 0.8513 0.4937 0.6149 0.2292 0.4557 0.7796
Peaking, Fully Continual, Balanced: 0.9998 0.9958 0.9950 0.8337 0.0850 0.1492 0.5005 0.1688 0.2598 0.0520 0.1414 0.3823
Peaking, Softmax 3 Stage: 1.0000 1.0000 0.9999 0.9805 0.2917 0.5073 0.8318 0.5005 0.6232 0.2817 0.4966 0.7811
Weighted Uniform, 30: 1.0000 0.9999 0.9993 0.9602 0.2329 0.3861 0.7409 0.3777 0.5005 0.1591 0.3069 0.6774
Weighted, Fully Continual, Standard 30: 1.0000 1.0000 1.0000 0.9982 0.6055 0.7716 0.9483 0.7191 0.8415 0.5005 0.6453 0.9359
Weighted, Fully Continual, Balanced 30: 1.0000 0.9999 0.9998 0.9841 0.3908 0.5452 0.8591 0.5044 0.6939 0.3556 0.5005 0.7386
Weighted, Softmax 3 Stage 30: 0.9999 0.9956 0.9983 0.8541 0.1135 0.2211 0.6186 0.2197 0.3234 0.0644 0.2622 0.5005
Saturating Uniform, 30
Saturating, Fully Continual, Standard
Saturating, Fully Continual, Balanced
Saturating, Softmax 3 Stage
Peaking Uniform, 30
Peaking, Fully Continual, Standard
Peaking, Fully Continual, Balanced
Peaking, Softmax 3 Stage
Weighted Uniform, 30
Weighted, Fully Continual, Standard 30
Weighted, Fully Continual, Balanced 30
Weighted, Softmax 3 Stage 30

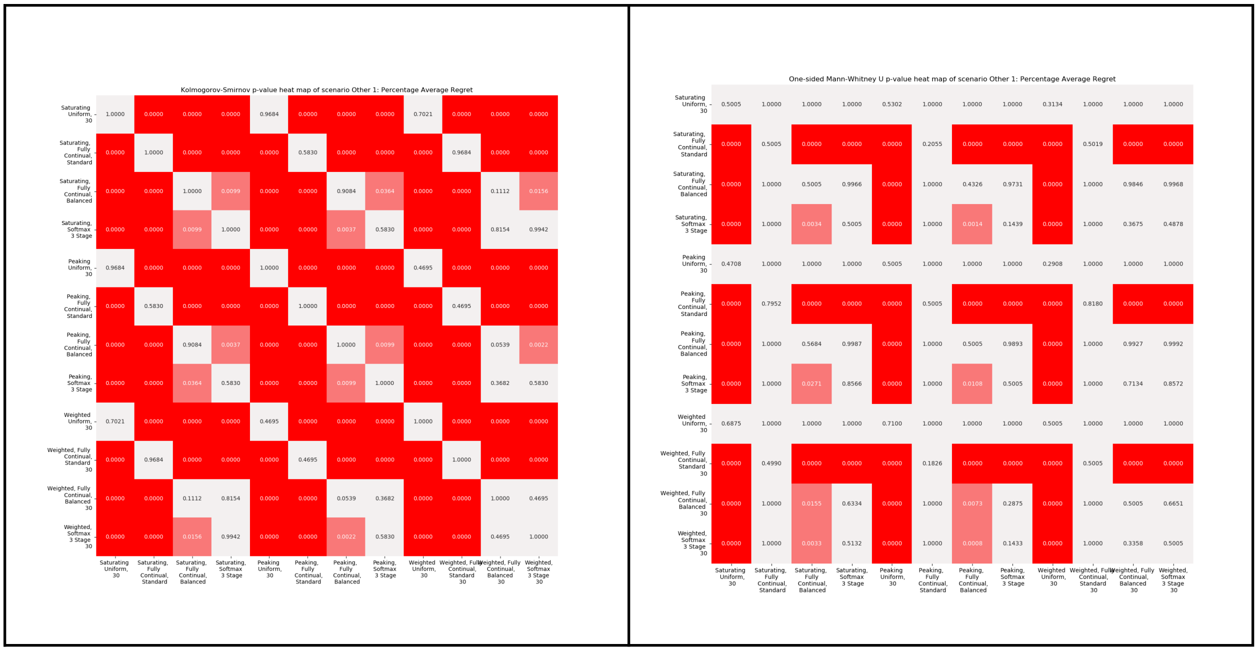

Figure Supplementary.Statistical.Obj2.X1. Kolmogorov–Smirnov (left) and Mann-Whitney U (right) heatmaps of p-values for objective 1, Scenario Other 1. These are for the metrics of PSR (top), Absolute Inaccuracy (middle) and PAR (bottom). Cells with a light pink hue represent the test statistic for that comparison would be significant under the threshold p<0.05, cells with a red hue represent the test statistic for that comparison would be significant under the threshold p<0.05 with Bonferri multiple comparison correction.

Scenario Other 2

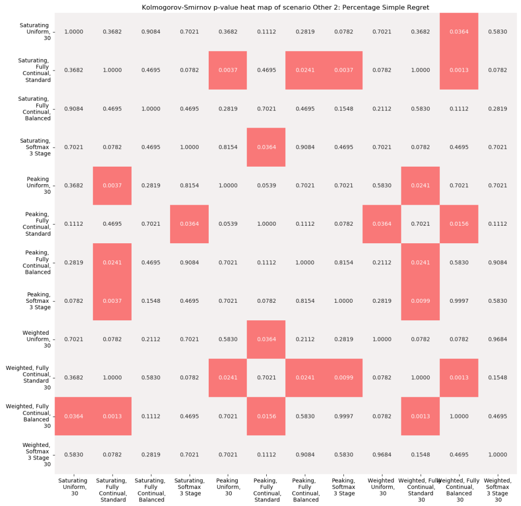
Kolmogorov-Smirnov p-value heat map of scenario Other 2: Percentage Simple Regret

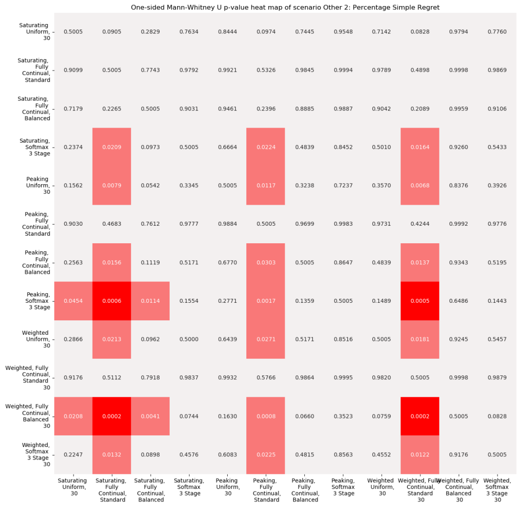
One-sided Mann-Whitney U p-value heat map of scenario Other 2: Percentage Simple Regret

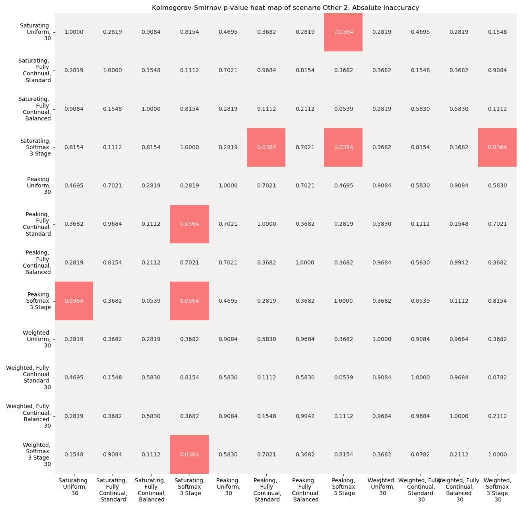
Kolmogorov-Smirnov p-value heat map of scenario Other 2: Absolute Inaccuracy

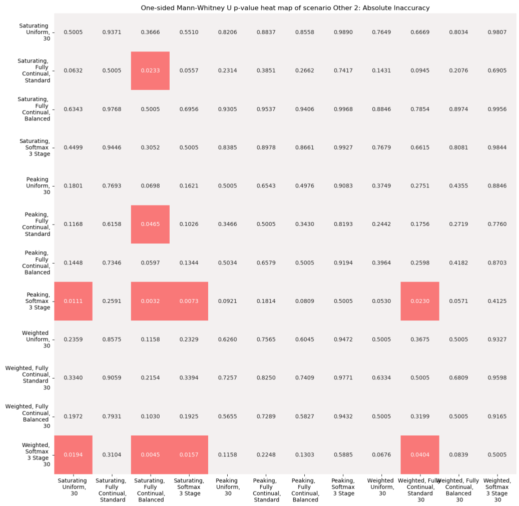
One-sided Mann-Whitney U p-value heat map of scenario Other 2: Absolute Inaccuracy

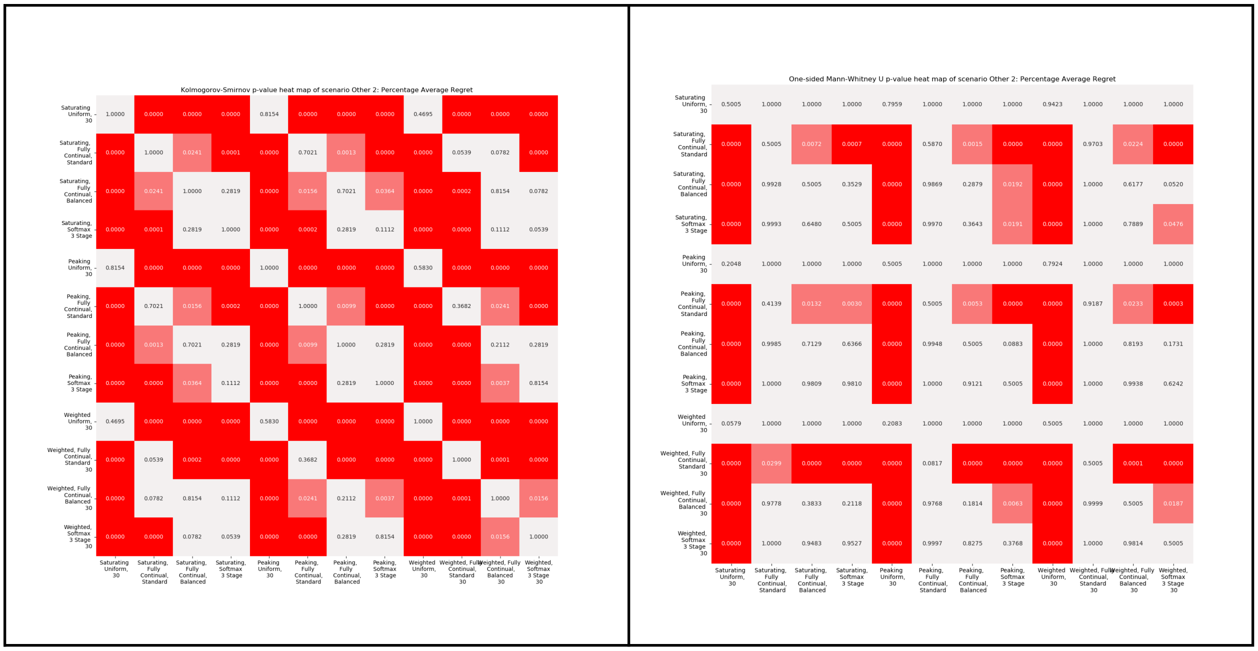

Figure Supplementary.Statistical.Obj2.X2. Kolmogorov–Smirnov (left) and Mann-Whitney U (right) heatmaps of p-values for objective 1, Scenario Other 2. These are for the metrics of PSR (top), Absolute Inaccuracy (middle) and PAR (bottom). Cells with a light pink hue represent the test statistic for that comparison would be significant under the threshold p<0.05, cells with a red hue represent the test statistic for that comparison would be significant under the threshold p<0.05 with Bonferri multiple comparison correction.

Scenario Other 3

Kolmogorov-Smirnov p-value heat map of scenario Other 3: Percentage Simple Regret

	Saturating Uniform, 30	Saturating, Fully Continual, Standard	Saturating, Fully Continual, Balanced	Saturating, Softmax 3 Stage	Peaking Uniform, 30	Peaking, Fully Continual, Standard	Peaking, Fully Continual, Balanced	Peaking, Softmax 3 Stage	Weighted Uniform, 30	Weighted, Fully Continual, Standard 30	Weighted, Fully Continual, Balanced 30	Weighted, Softmax 3 Stage 30
Saturating Uniform, 30	1.0000	0.0004	0.0000	0.0364	0.0000	0.0000	0.0000	0.0000	0.0001	0.0000	0.0000	0.0000
Saturating, Fully Continual, Standard	0.0004	1.0000	0.4695	0.0364	0.0007	0.3682	0.0004	0.0782	0.0782	0.0241	0.1548	0.2819
Saturating, Fully Continual, Balanced	0.0000	0.4695	1.0000	0.2819	0.0099	0.7021	0.0004	0.2112	0.2819	0.0061	0.1548	0.7021
Saturating, Softmax 3 Stage	0.0364	0.0364	0.2819	1.0000	0.0000	0.0782	0.0000	0.0022	0.2112	0.0001	0.0156	0.0539
Peaking Uniform, 30	0.0000	0.0007	0.0099	0.0000	1.0000	0.0061	0.0241	0.5830	0.0004	0.1548	0.0364	0.0156
Peaking, Fully Continual, Standard	0.0000	0.3682	0.7021	0.0782	0.0061	1.0000	0.0037	0.3682	0.1548	0.0364	0.8154	0.9084
Peaking, Fully Continual, Balanced	0.0000	0.0004	0.0004	0.0000	0.0241	0.0037	1.0000	0.1548	0.0000	0.3682	0.0364	0.0022
Peaking, Softmax 3 Stage	0.0000	0.0782	0.2112	0.0022	0.5830	0.3682	0.1548	1.0000	0.0061	0.2819	0.7021	0.3682
Weighted Uniform, 30	0.0001	0.0782	0.2819	0.2112	0.0004	0.1548	0.0000	0.0061	1.0000	0.0001	0.0156	0.3682
Weighted, Fully Continual, Standard 30	0.0000	0.0241	0.0061	0.0001	0.1548	0.0364	0.3682	0.2819	0.0001	1.0000	0.3682	0.0241
Weighted, Fully Continual, Balanced 30	0.0000	0.1548	0.1548	0.0156	0.0364	0.8154	0.0364	0.7021	0.0156	0.3682	1.0000	0.3682
Weighted, Softmax 3 Stage 30	0.0000	0.2819	0.7021	0.0539	0.0156	0.9084	0.0022	0.3682	0.3682	0.0241	0.3682	1.0000

One-sided Mann-Whitney U p-value heat map of scenario Other 3: Percentage Simple Regret

	Saturating Uniform, 30	Saturating, Fully Continual, Standard	Saturating, Fully Continual, Balanced	Saturating, Softmax 3 Stage	Peaking Uniform, 30	Peaking, Fully Continual, Standard	Peaking, Fully Continual, Balanced	Peaking, Softmax 3 Stage	Weighted Uniform, 30	Weighted, Fully Continual, Standard 30	Weighted, Fully Continual, Balanced 30	Weighted, Softmax 3 Stage 30
Saturating Uniform, 30	0.5005	0.9997	0.9999	0.9846	1.0000	1.0000	1.0000	1.0000	0.9997	1.0000	1.0000	1.0000
Saturating, Fully Continual, Standard	0.0003	0.5005	0.4863	0.0352	0.9886	0.7459	0.9993	0.9453	0.2582	0.9893	0.9456	0.6692
Saturating, Fully Continual, Balanced	0.0001	0.5146	0.5005	0.0385	0.9963	0.7643	0.9999	0.9704	0.2203	0.9977	0.9621	0.6750
Saturating, Softmax 3 Stage	0.0155	0.9650	0.9617	0.5005	1.0000	0.9944	1.0000	0.9998	0.8847	1.0000	0.9998	0.9904
Peaking Uniform, 30	0.0000	0.0115	0.0037	0.0000	0.5005	0.0230	0.9298	0.2411	0.0001	0.6801	0.2030	0.0071
Peaking, Fully Continual, Standard	0.0000	0.2549	0.2365	0.0057	0.9771	0.5005	0.9989	0.8921	0.0701	0.9813	0.8731	0.4325
Peaking, Fully Continual, Balanced	0.0000	0.0007	0.0001	0.0000	0.0706	0.0011	0.5005	0.0182	0.0000	0.2040	0.0272	0.0002
Peaking, Softmax 3 Stage	0.0000	0.0550	0.0297	0.0002	0.7596	0.1084	0.9819	0.5005	0.0026	0.8629	0.4761	0.0654
Weighted Uniform, 30	0.0003	0.7426	0.7804	0.1158	0.9999	0.9302	1.0000	0.9974	0.5005	0.9998	0.9946	0.8977
Weighted, Fully Continual, Standard 30	0.0000	0.0108	0.0023	0.0000	0.3207	0.0188	0.7967	0.1376	0.0002	0.5005	0.1889	0.0092
Weighted, Fully Continual, Balanced 30	0.0000	0.0547	0.0382	0.0003	0.7977	0.1275	0.9729	0.5249	0.0055	0.8118	0.5005	0.0959
Weighted, Softmax 3 Stage 30	0.0000	0.3317	0.3259	0.0096	0.9930	0.5684	0.9998	0.9349	0.1028	0.9909	0.9045	0.5005

Kolmogorov-Smirnov p-value heat map of scenario Other 3: Absolute Inaccuracy

	Saturating Uniform, 30	Saturating, Fully Continual, Standard	Saturating, Fully Continual, Balanced	Saturating, Softmax 3 Stage	Peaking Uniform, 30	Peaking, Fully Continual, Standard	Peaking, Fully Continual, Balanced	Peaking, Softmax 3 Stage	Weighted Uniform, 30	Weighted, Fully Continual, Standard 30	Weighted, Fully Continual, Balanced 30	Weighted, Softmax 3 Stage 30
Saturating Uniform, 30	1.0000	0.2112	0.7021	0.5830	0.0061	0.0782	0.0004	0.0022	0.3682	0.0156	0.2112	0.0061
Saturating, Fully Continual, Standard	0.2112	1.0000	0.2819	0.5830	0.0241	0.2112	0.0022	0.0099	0.4695	0.0539	0.3682	0.0156
Saturating, Fully Continual, Balanced	0.7021	0.2819	1.0000	0.9084	0.1112	0.5830	0.0156	0.0539	0.9084	0.2112	0.9084	0.0782
Saturating, Softmax 3 Stage	0.5830	0.5830	0.9084	1.0000	0.1548	0.9084	0.0241	0.0539	0.4695	0.1548	0.5830	0.0782
Peaking Uniform, 30	0.0061	0.0241	0.1112	0.1548	1.0000	0.3682	0.3682	0.4695	0.1548	0.8154	0.4695	0.3682
Peaking, Fully Continual, Standard	0.0782	0.2112	0.5830	0.9084	0.3682	1.0000	0.2112	0.1548	0.4695	0.3682	0.7021	0.2819
Peaking, Fully Continual, Balanced	0.0004	0.0022	0.0156	0.0241	0.3682	0.2112	1.0000	0.7021	0.0782	0.5830	0.2819	0.4695
Peaking, Softmax 3 Stage	0.0022	0.0099	0.0539	0.0539	0.4695	0.1548	0.7021	1.0000	0.0539	0.5830	0.1548	0.1548
Weighted Uniform, 30	0.3682	0.4695	0.9084	0.4695	0.1548	0.4695	0.0782	0.0539	1.0000	0.1548	0.9942	0.2819
Weighted, Fully Continual, Standard 30	0.0156	0.0539	0.2112	0.1548	0.8154	0.3682	0.5830	0.5830	0.1548	1.0000	0.4695	0.2112
Weighted, Fully Continual, Balanced 30	0.2112	0.3682	0.9084	0.5830	0.4695	0.7021	0.2819	0.1548	0.9942	0.4695	1.0000	0.5830
Weighted, Softmax 3 Stage 30	0.0061	0.0156	0.0782	0.0782	0.3682	0.2819	0.4695	0.1548	0.2819	0.2112	0.5830	1.0000

One-sided Mann-Whitney U p-value heat map of scenario Other 3: Absolute Inaccuracy

	Saturating Uniform, 30	Saturating, Fully Continual, Standard	Saturating, Fully Continual, Balanced	Saturating, Softmax 3 Stage	Peaking Uniform, 30	Peaking, Fully Continual, Standard	Peaking, Fully Continual, Balanced	Peaking, Softmax 3 Stage	Weighted Uniform, 30	Weighted, Fully Continual, Standard 30	Weighted, Fully Continual, Balanced 30	Weighted, Softmax 3 Stage 30
Saturating Uniform, 30	0.5005	0.7973	0.8121	0.7945	0.9981	0.9265	0.9978	0.9981	0.8943	0.9974	0.9327	0.9836
Saturating, Fully Continual, Standard	0.2034	0.5005	0.4791	0.4182	0.9642	0.6642	0.9615	0.9659	0.6045	0.9321	0.6999	0.8842
Saturating, Fully Continual, Balanced	0.1885	0.5219	0.5005	0.4364	0.9821	0.7488	0.9684	0.9814	0.5904	0.9709	0.7142	0.8650
Saturating, Softmax 3 Stage	0.2062	0.5827	0.5645	0.5005	0.9832	0.7142	0.9719	0.9770	0.6624	0.9766	0.7634	0.9075
Peaking Uniform, 30	0.0019	0.0360	0.0180	0.0169	0.5005	0.0705	0.3565	0.4287	0.0278	0.3898	0.0638	0.1470
Peaking, Fully Continual, Standard	0.0738	0.3367	0.2520	0.2866	0.9298	0.5005	0.8607	0.8925	0.3466	0.9009	0.4625	0.6827
Peaking, Fully Continual, Balanced	0.0023	0.0388	0.0317	0.0283	0.6444	0.1398	0.5005	0.6167	0.0333	0.5818	0.0870	0.1820
Peaking, Softmax 3 Stage	0.0019	0.0343	0.0187	0.0232	0.5722	0.1079	0.3842	0.5005	0.0241	0.5171	0.0651	0.1287
Weighted Uniform, 30	0.1061	0.3964	0.4106	0.3385	0.9724	0.6543	0.9668	0.9760	0.5005	0.9578	0.6398	0.8508
Weighted, Fully Continual, Standard 30	0.0026	0.0682	0.0292	0.0236	0.6111	0.0996	0.4192	0.4839	0.0424	0.5005	0.0897	0.1775
Weighted, Fully Continual, Balanced 30	0.0676	0.3010	0.2866	0.2374	0.9365	0.5384	0.9134	0.9353	0.3611	0.9107	0.5005	0.7183
Weighted, Softmax 3 Stage 30	0.0165	0.1163	0.1355	0.0929	0.8536	0.3182	0.8186	0.8718	0.1498	0.8231	0.2825	0.5005

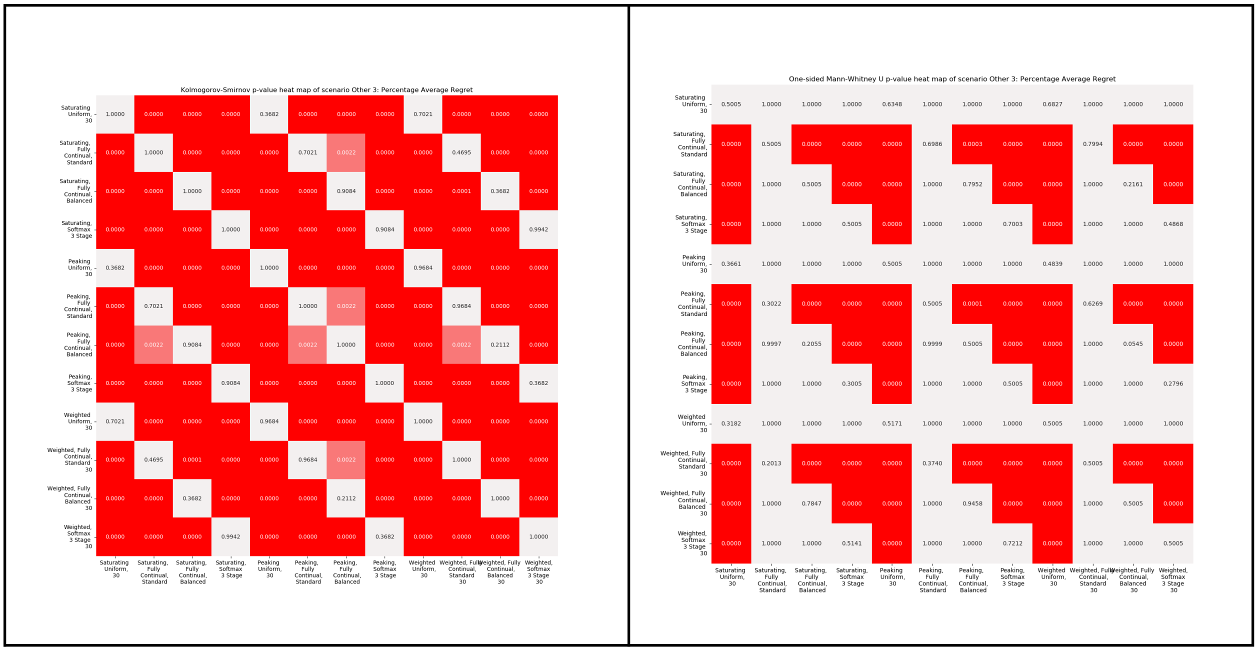

Figure Supplementary.Statistical.Obj2.X3. Kolmogorov–Smirnov (left) and Mann-Whitney U (right) heatmaps of p-values for objective 1, Scenario Other 3. These are for the metrics of PSR (top), Absolute Inaccuracy (middle) and PAR (bottom). Cells with a light pink hue represent the test statistic for that comparison would be significant under the threshold $p<0.05$, cells with a red hue represent the test statistic for that comparison would be significant under the threshold $p<0.05$ with Bonferri multiple comparison correction.

Scenario Other 4

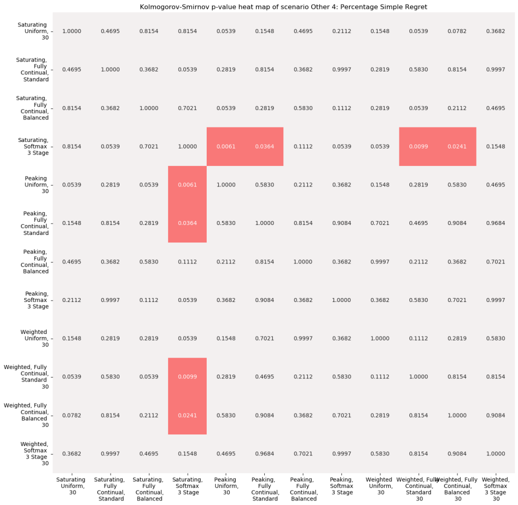
Kolmogorov-Smirnov p-value heat map of scenario Other 4: Percentage Simple Regret
Saturating Uniform, 30 1.0000 0.4695 0.8154 0.8154 0.0539 0.1548 0.4695 0.2112 0.1548 0.0539 0.0782 0.3682
Saturating, Fully Continual, Standard 0.4695 1.0000 0.3682 0.0539 0.2819 0.8154 0.3682 0.9997 0.2819 0.5830 0.8154 0.9997
Saturating, Fully Continual, Balanced 0.8154 0.3682 1.0000 0.7021 0.0539 0.2819 0.5830 0.1112 0.2819 0.0539 0.2112 0.4695
Saturating, Softmax 3 Stage 0.8154 0.0539 0.7021 1.0000 0.0061 0.0364 0.1112 0.0539 0.0539 0.0099 0.0241 0.1548
Peaking Uniform, 30 0.0539 0.2819 0.0539 0.0061 1.0000 0.5830 0.2112 0.3682 0.1548 0.2819 0.5830 0.4695
Peaking, Fully Continual, Standard 0.1548 0.8154 0.2819 0.0364 0.5830 1.0000 0.8154 0.9084 0.7021 0.4695 0.9084 0.9684
Peaking, Fully Continual, Balanced 0.4695 0.3682 0.5830 0.1112 0.2112 0.8154 1.0000 0.3682 0.9997 0.2112 0.3682 0.7021
Peaking, Softmax 3 Stage 0.2112 0.9997 0.1112 0.0539 0.3682 0.9084 0.3682 1.0000 0.3682 0.5830 0.7021 0.9997
Weighted Uniform, 30 0.1548 0.2819 0.2819 0.0539 0.1548 0.7021 0.9997 0.3682 1.0000 0.1112 0.2819 0.5830
Weighted, Fully Continual, Standard 30 0.0539 0.5830 0.0539 0.0099 0.2819 0.4695 0.2112 0.5830 0.1112 1.0000 0.8154 0.8154
Weighted, Fully Continual, Balanced 30 0.0782 0.8154 0.2112 0.0241 0.5830 0.9084 0.3682 0.7021 0.2819 0.8154 1.0000 0.9084
Weighted, Softmax 3 Stage 30 0.3682 0.9997 0.4695 0.1548 0.4695 0.9684 0.7021 0.9997 0.5830 0.8154 0.9084 1.0000
Saturating Uniform, 30
Saturating, Fully Continual, Standard
Saturating, Fully Continual, Balanced
Saturating, Softmax 3 Stage
Peaking Uniform, 30
Peaking, Fully Continual, Standard
Peaking, Fully Continual, Balanced
Peaking, Softmax 3 Stage
Weighted Uniform, 30
Weighted, Fully Continual, Standard 30
Weighted, Fully Continual, Balanced 30
Weighted, Softmax 3 Stage 30

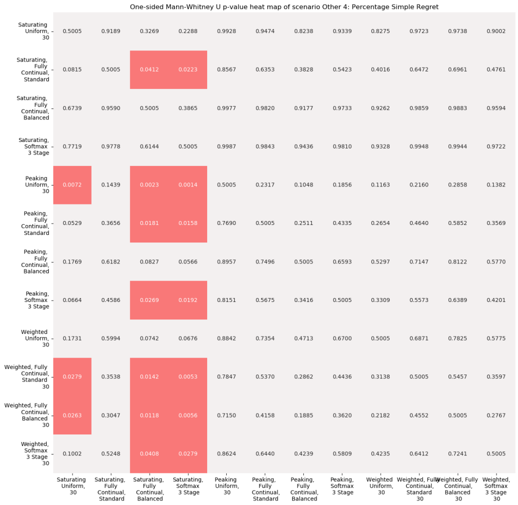
One-sided Mann-Whitney U p-value heat map of scenario Other 4: Percentage Simple Regret
Saturating Uniform, 30 0.5005 0.9189 0.3269 0.2288 0.9928 0.9474 0.8238 0.9339 0.8275 0.9723 0.9738 0.9002
Saturating, Fully Continual, Standard 0.0815 0.5005 0.0412 0.0223 0.8567 0.6353 0.3828 0.5423 0.4016 0.6472 0.6961 0.4761
Saturating, Fully Continual, Balanced 0.6739 0.9590 0.5005 0.3865 0.9977 0.9820 0.9177 0.9733 0.9262 0.9859 0.9883 0.9594
Saturating, Softmax 3 Stage 0.7719 0.9778 0.6144 0.5005 0.9987 0.9843 0.9436 0.9810 0.9328 0.9948 0.9944 0.9722
Peaking Uniform, 30 0.0072 0.1439 0.0023 0.0014 0.5005 0.2317 0.1048 0.1856 0.1163 0.2160 0.2858 0.1382
Peaking, Fully Continual, Standard 0.0529 0.3656 0.0181 0.0158 0.7690 0.5005 0.2511 0.4335 0.2654 0.4640 0.5852 0.3569
Peaking, Fully Continual, Balanced 0.1769 0.6182 0.0827 0.0566 0.8957 0.7496 0.5005 0.6593 0.5297 0.7147 0.8122 0.5770
Peaking, Softmax 3 Stage 0.0664 0.4586 0.0269 0.0192 0.8151 0.5675 0.3416 0.5005 0.3309 0.5573 0.6389 0.4201
Weighted Uniform, 30 0.1731 0.5994 0.0742 0.0676 0.8842 0.7354 0.4713 0.6700 0.5005 0.6871 0.7825 0.5775
Weighted, Fully Continual, Standard 30 0.0279 0.3538 0.0142 0.0053 0.7847 0.5370 0.2862 0.4436 0.3138 0.5005 0.5457 0.3597
Weighted, Fully Continual, Balanced 30 0.0263 0.3047 0.0118 0.0056 0.7150 0.4158 0.1885 0.3620 0.2182 0.4552 0.5005 0.2767
Weighted, Softmax 3 Stage 30 0.1002 0.5248 0.0408 0.0279 0.8624 0.6440 0.4239 0.5809 0.4235 0.6412 0.7241 0.5005
Saturating Uniform, 30
Saturating, Fully Continual, Standard
Saturating, Fully Continual, Balanced
Saturating, Softmax 3 Stage
Peaking Uniform, 30
Peaking, Fully Continual, Standard
Peaking, Fully Continual, Balanced
Peaking, Softmax 3 Stage
Weighted Uniform, 30
Weighted, Fully Continual, Standard 30
Weighted, Fully Continual, Balanced 30
Weighted, Softmax 3 Stage 30

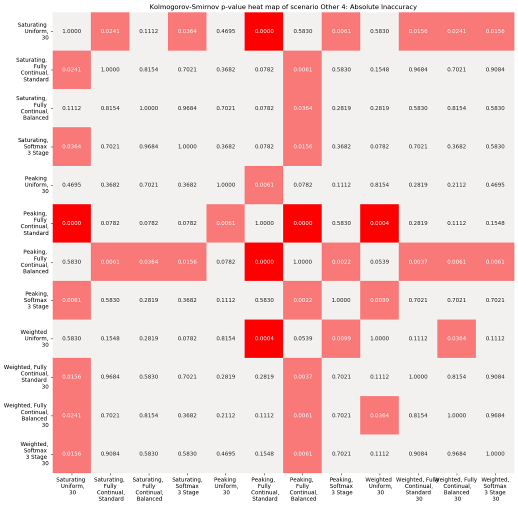
Kolmogorov-Smirnov p-value heat map of scenario Other 4: Absolute Inaccuracy
Saturating Uniform, 30 1.0000 0.0241 0.1112 0.0364 0.4695 0.0000 0.5830 0.0061 0.5830 0.0156 0.0241 0.0156
Saturating, Fully Continual, Standard 0.0241 1.0000 0.8154 0.7021 0.3682 0.0782 0.0061 0.5830 0.1548 0.9684 0.7021 0.9084
Saturating, Fully Continual, Balanced 0.1112 0.8154 1.0000 0.9684 0.7021 0.0782 0.0364 0.2819 0.2819 0.5830 0.8154 0.5830
Saturating, Softmax 3 Stage 0.0364 0.7021 0.9684 1.0000 0.3682 0.0782 0.0156 0.3682 0.0782 0.7021 0.3682 0.5830
Peaking Uniform, 30 0.4695 0.3682 0.7021 0.3682 1.0000 0.0061 0.0782 0.1112 0.8154 0.2819 0.2112 0.4695
Peaking, Fully Continual, Standard 0.0000 0.0782 0.0782 0.0782 0.0061 1.0000 0.0000 0.5830 0.0004 0.2819 0.1112 0.1548
Peaking, Fully Continual, Balanced 0.5830 0.0061 0.0364 0.0156 0.0782 0.0000 1.0000 0.0022 0.0539 0.0037 0.0061 0.0061
Peaking, Softmax 3 Stage 0.0061 0.5830 0.2819 0.3682 0.1112 0.5830 0.0022 1.0000 0.0099 0.7021 0.7021 0.7021
Weighted Uniform, 30 0.5830 0.1548 0.2819 0.0782 0.8154 0.0004 0.0539 0.0099 1.0000 0.1112 0.0364 0.1112
Weighted, Fully Continual, Standard 30 0.0156 0.9684 0.5830 0.7021 0.2819 0.2819 0.0037 0.7021 0.1112 1.0000 0.8154 0.9084
Weighted, Fully Continual, Balanced 30 0.0241 0.7021 0.8154 0.3682 0.2112 0.1112 0.0061 0.7021 0.0364 0.8154 1.0000 0.9684
Weighted, Softmax 3 Stage 30 0.0156 0.9084 0.5830 0.5830 0.4695 0.1548 0.0061 0.7021 0.1112 0.9084 0.9684 1.0000
Saturating Uniform, 30
Saturating, Fully Continual, Standard
Saturating, Fully Continual, Balanced
Saturating, Softmax 3 Stage
Peaking Uniform, 30
Peaking, Fully Continual, Standard
Peaking, Fully Continual, Balanced
Peaking, Softmax 3 Stage
Weighted Uniform, 30
Weighted, Fully Continual, Standard 30
Weighted, Fully Continual, Balanced 30
Weighted, Softmax 3 Stage 30

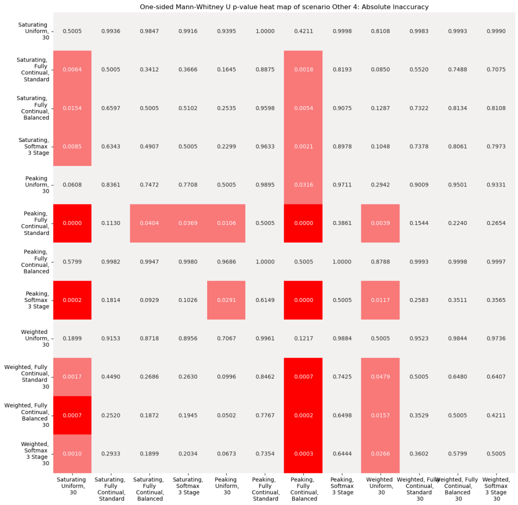
One-sided Mann-Whitney U p-value heat map of scenario Other 4: Absolute Inaccuracy
Saturating Uniform, 30 0.5005 0.9936 0.9847 0.9916 0.9395 1.0000 0.4211 0.9998 0.8108 0.9983 0.9993 0.9990
Saturating, Fully Continual, Standard 0.0064 0.5005 0.3412 0.3666 0.1645 0.8875 0.0018 0.8193 0.0850 0.5520 0.7488 0.7075
Saturating, Fully Continual, Balanced 0.0154 0.6597 0.5005 0.5102 0.2535 0.9598 0.0054 0.9075 0.1287 0.7322 0.8134 0.8108
Saturating, Softmax 3 Stage 0.0085 0.6343 0.4907 0.5005 0.2299 0.9633 0.0021 0.8978 0.1048 0.7378 0.8061 0.7973
Peaking Uniform, 30 0.0608 0.8361 0.7472 0.7708 0.5005 0.9895 0.0316 0.9711 0.2942 0.9009 0.9501 0.9331
Peaking, Fully Continual, Standard 0.0000 0.1130 0.0404 0.0369 0.0106 0.5005 0.0000 0.3861 0.0039 0.1544 0.2240 0.2654
Peaking, Fully Continual, Balanced 0.5799 0.9982 0.9947 0.9980 0.9686 1.0000 0.5005 1.0000 0.8788 0.9993 0.9998 0.9997
Peaking, Softmax 3 Stage 0.0002 0.1814 0.0929 0.1026 0.0291 0.6149 0.0000 0.5005 0.0117 0.2583 0.3511 0.3565
Weighted Uniform, 30 0.1899 0.9153 0.8718 0.8956 0.7067 0.9961 0.1217 0.9884 0.5005 0.9523 0.9844 0.9736
Weighted, Fully Continual, Standard 30 0.0017 0.4490 0.2686 0.2630 0.0996 0.8462 0.0007 0.7425 0.0479 0.5005 0.6480 0.6407
Weighted, Fully Continual, Balanced 30 0.0007 0.2520 0.1872 0.1945 0.0502 0.7767 0.0002 0.6498 0.0157 0.3529 0.5005 0.4211
Weighted, Softmax 3 Stage 30 0.0010 0.2933 0.1899 0.2034 0.0673 0.7354 0.0003 0.6444 0.0266 0.3602 0.5799 0.5005
Saturating Uniform, 30
Saturating, Fully Continual, Standard
Saturating, Fully Continual, Balanced
Saturating, Softmax 3 Stage
Peaking Uniform, 30
Peaking, Fully Continual, Standard
Peaking, Fully Continual, Balanced
Peaking, Softmax 3 Stage
Weighted Uniform, 30
Weighted, Fully Continual, Standard 30
Weighted, Fully Continual, Balanced 30
Weighted, Softmax 3 Stage 30

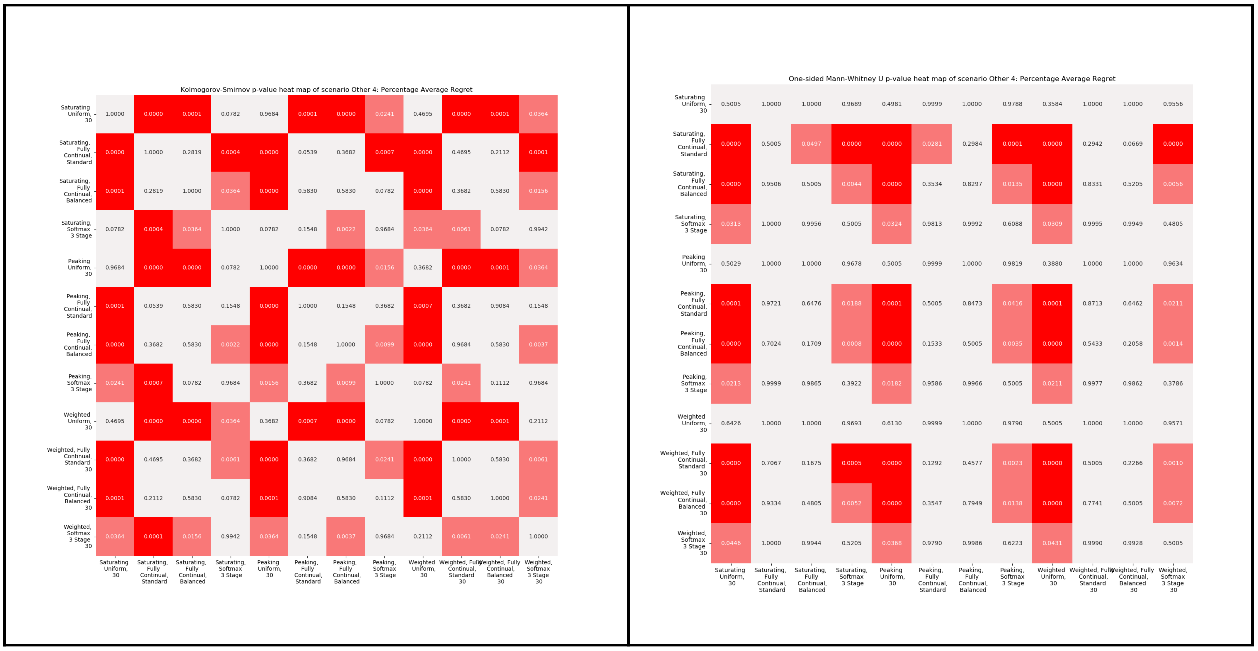

Figure Supplementary.Statistical.Obj2.X4. Kolmogorov–Smirnov (left) and Mann-Whitney U (right) heatmaps of p-values for objective 1, Scenario Other 4. These are for the metrics of PSR (top), Absolute Inaccuracy (middle) and PAR (bottom). Cells with a light pink hue represent the test statistic for that comparison would be significant under the threshold p<0.05, cells with a red hue represent the test statistic for that comparison would be significant under the threshold p<0.05 with Bonferri multiple comparison correction.

Supplementary 13. Objective 2 Plots

Scenario Saturating 1

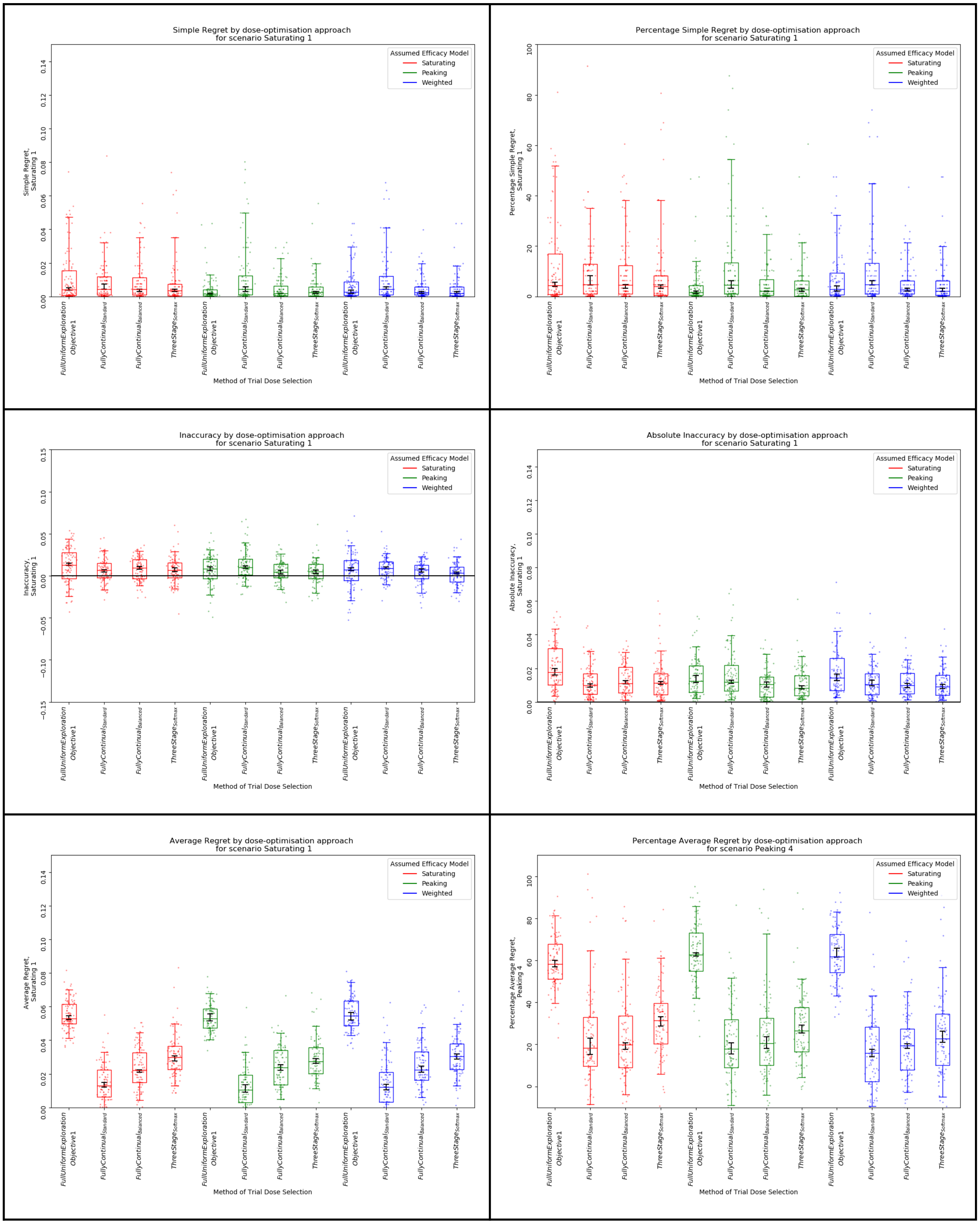

Figure Supplementary.Obj2_Plots.Saturating 1. Plots of the metrics from simulations for dose-optimisation approaches in objective 2 for scenario Saturating 1. The shown metrics are simple regret (top left), percentage simple regret (top right), inaccuracy (middle left), absolute

inaccuracy (middle right), average regret (bottom left), and percentage average regret (bottom right).

Scenario Saturating 2

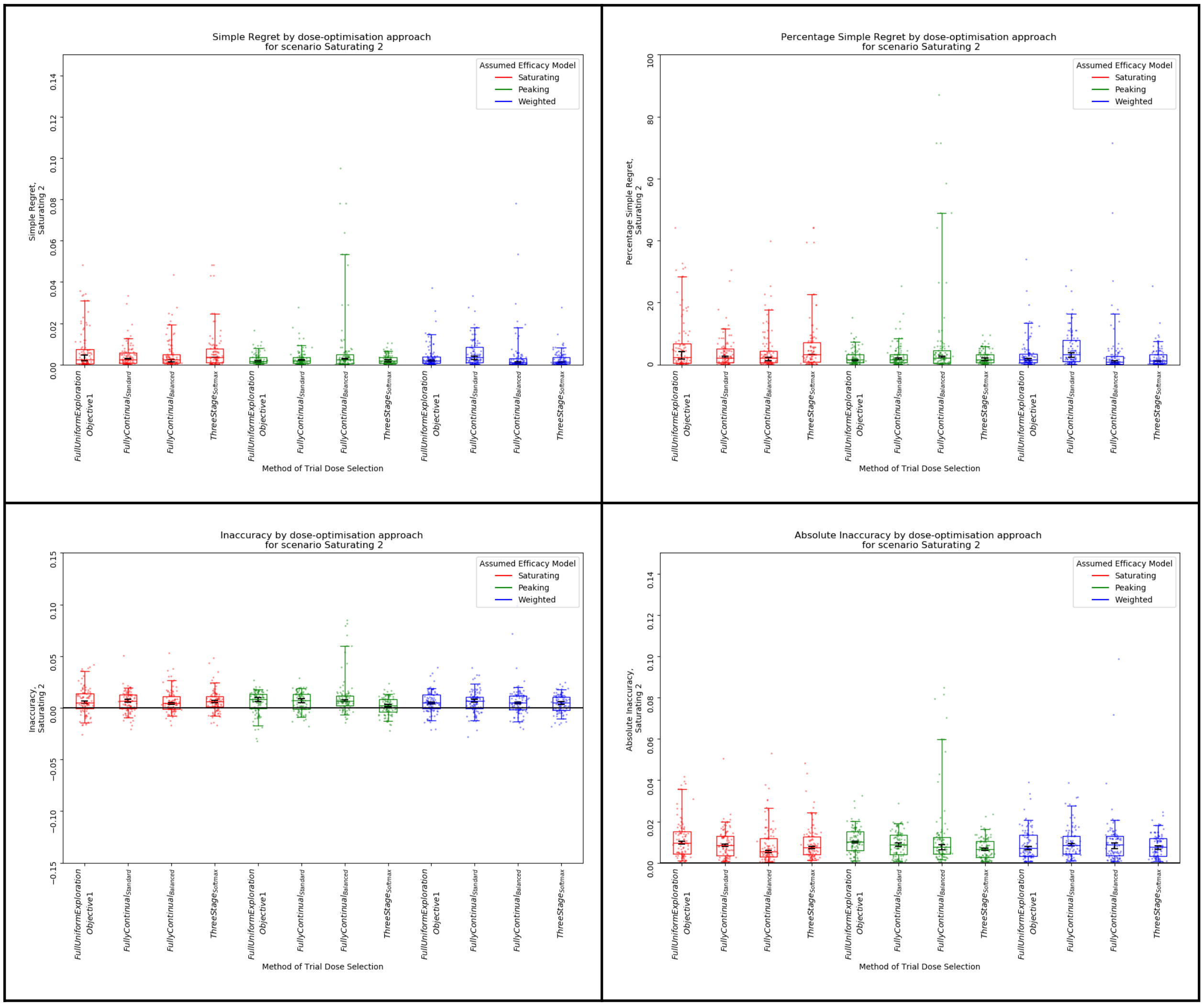

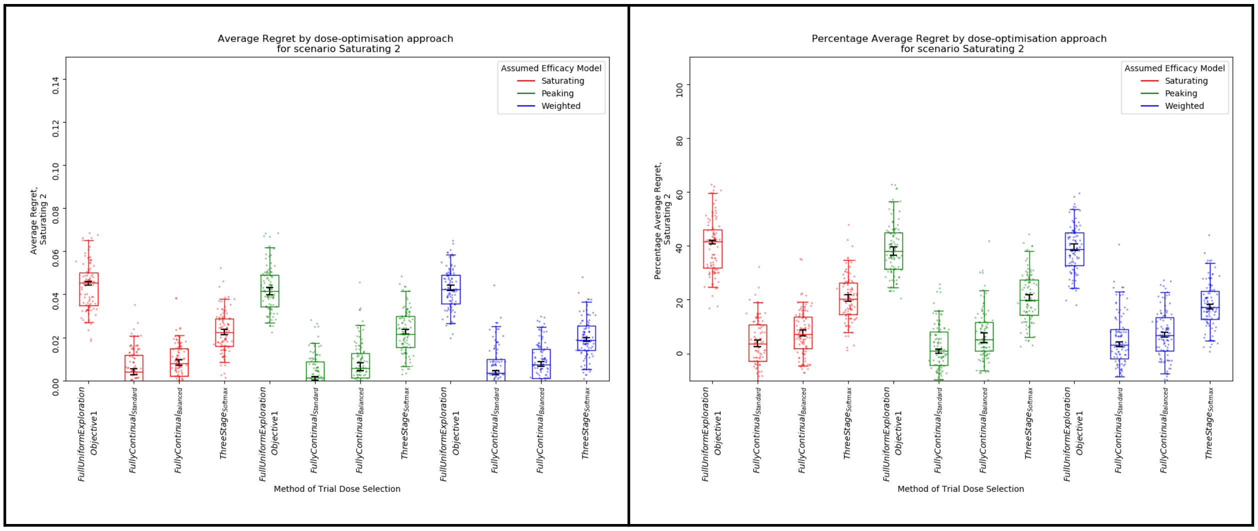

Figure Supplementary.Obj2_Plots.Saturating 2. Plots of the metrics from simulations for dose-optimisation approaches in objective 2 for scenario Saturating 2. The shown metrics are simple regret (top left), percentage simple regret (top right), inaccuracy (middle left), absolute inaccuracy (middle right), average regret (bottom left), and percentage average regret (bottom right).

Scenario Saturating 3

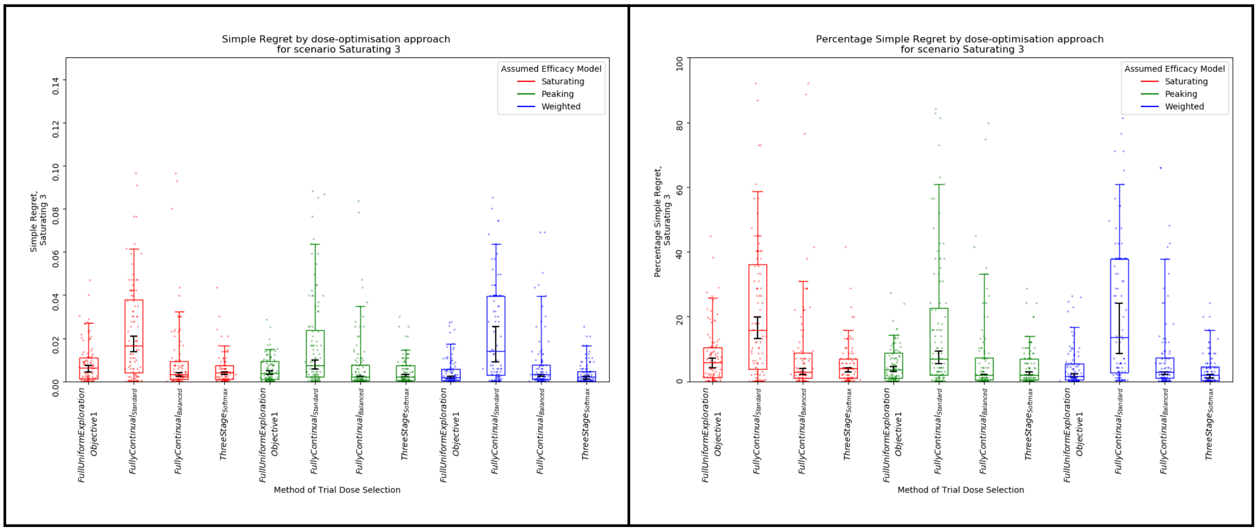

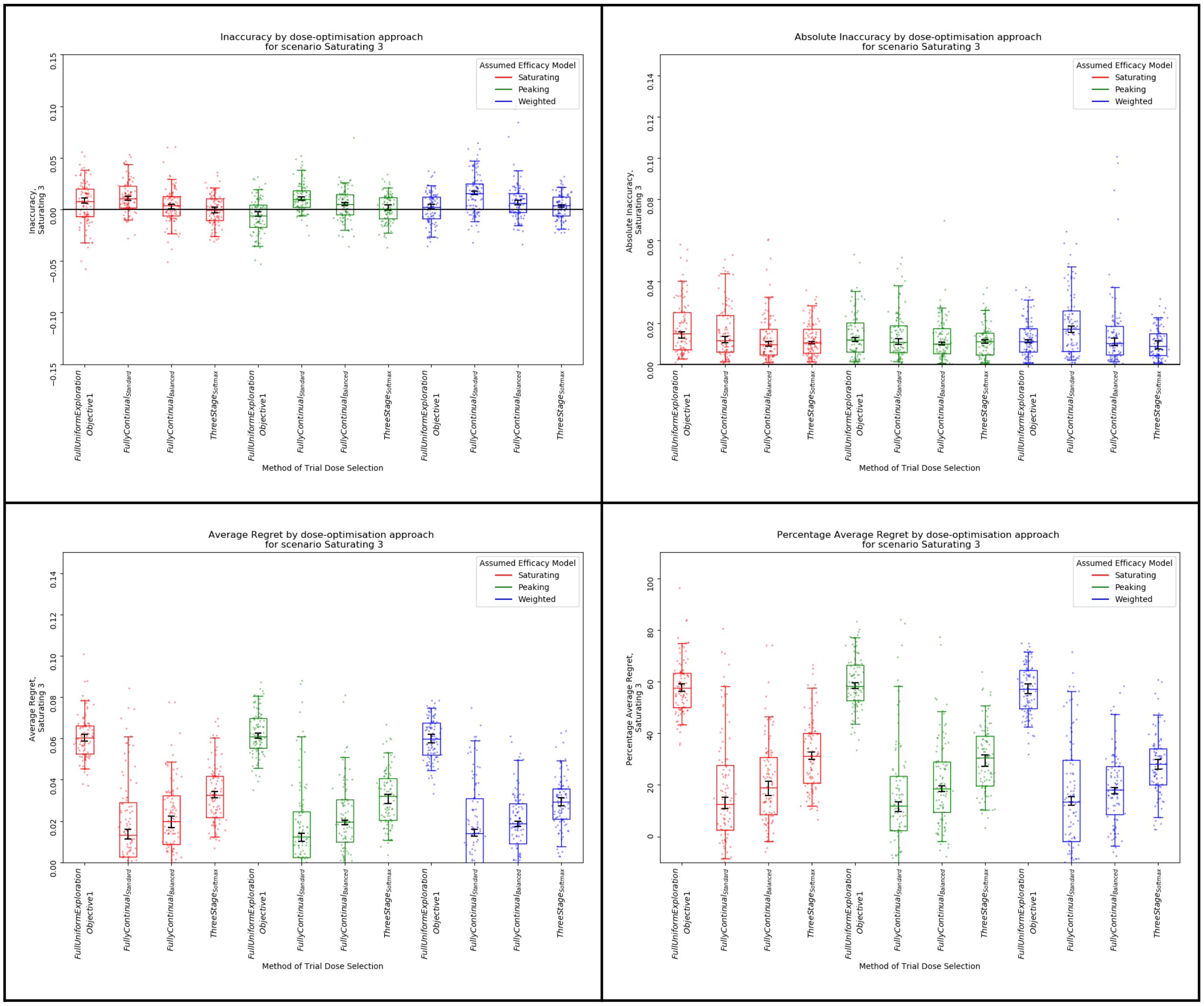

Figure Supplementary.Obj2_Plots.Saturating 3. Plots of the metrics from simulations for dose-optimisation approaches in objective 2 for scenario Saturating 3. The shown metrics are simple regret (top left), percentage simple regret (top right), inaccuracy (middle left), absolute inaccuracy (middle right), average regret (bottom left), and percentage average regret (bottom right).

Scenario Saturating 4

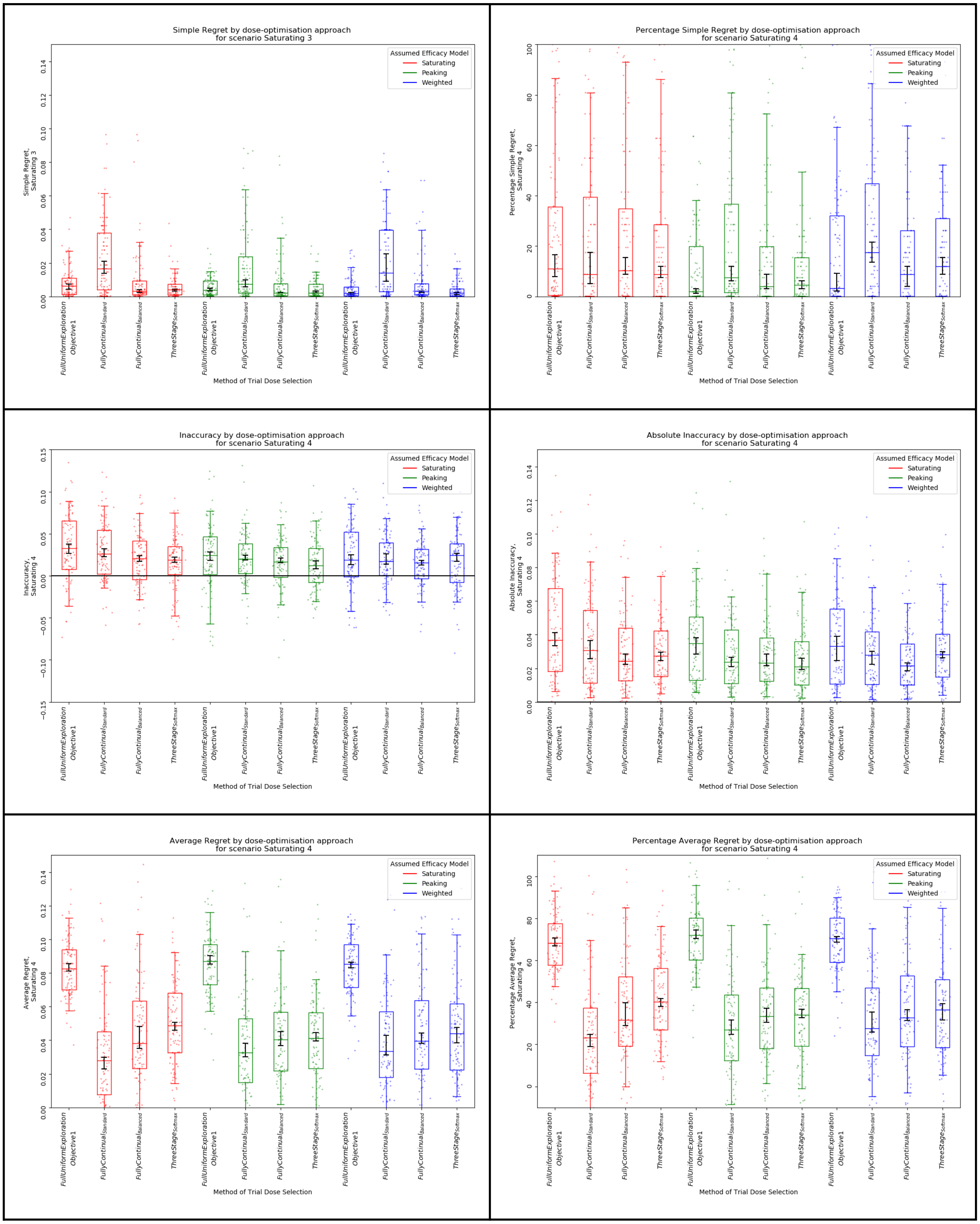

Figure Supplementary.Obj2_Plots.Saturating 4. Plots of the metrics from simulations for dose-optimisation approaches in objective 2 for scenario Saturating 4. The shown metrics are simple regret (top left), percentage simple regret (top right), inaccuracy (middle left), absolute

inaccuracy (middle right), average regret (bottom left), and percentage average regret (bottom right).

Scenario Saturating 5

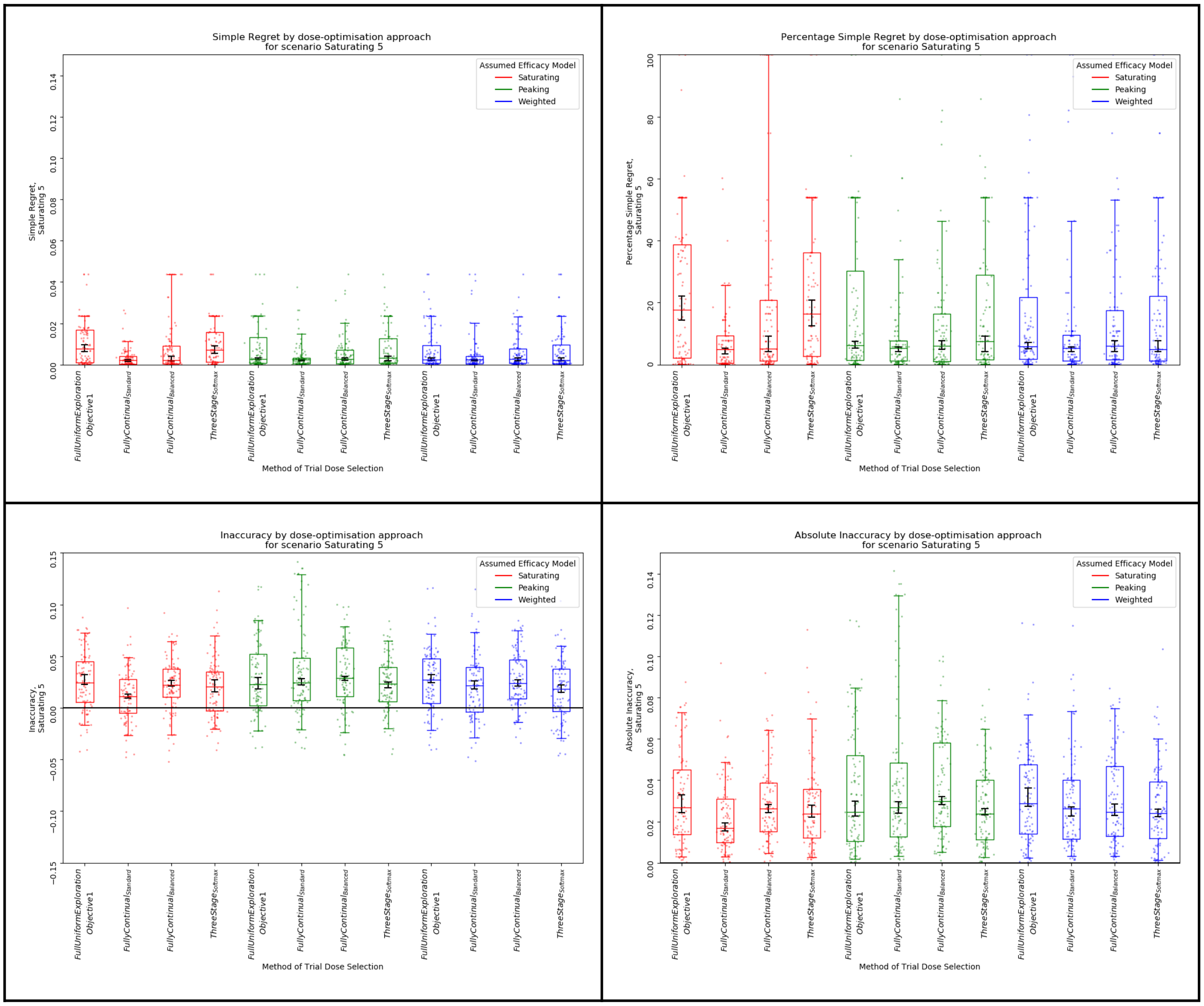

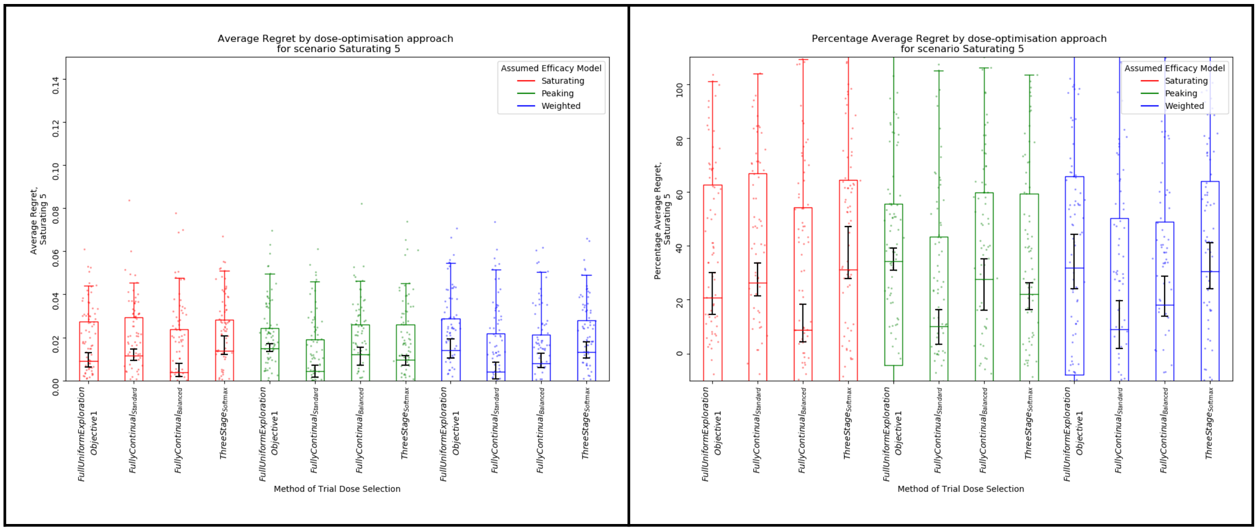

Figure Supplementary.Obj2_Plots.Saturating 5. Plots of the metrics from simulations for dose-optimisation approaches in objective 2 for scenario Saturating 5. The shown metrics are simple regret (top left), percentage simple regret (top right), inaccuracy (middle left), absolute inaccuracy (middle right), average regret (bottom left), and percentage average regret (bottom right).

Scenario Peaking 1

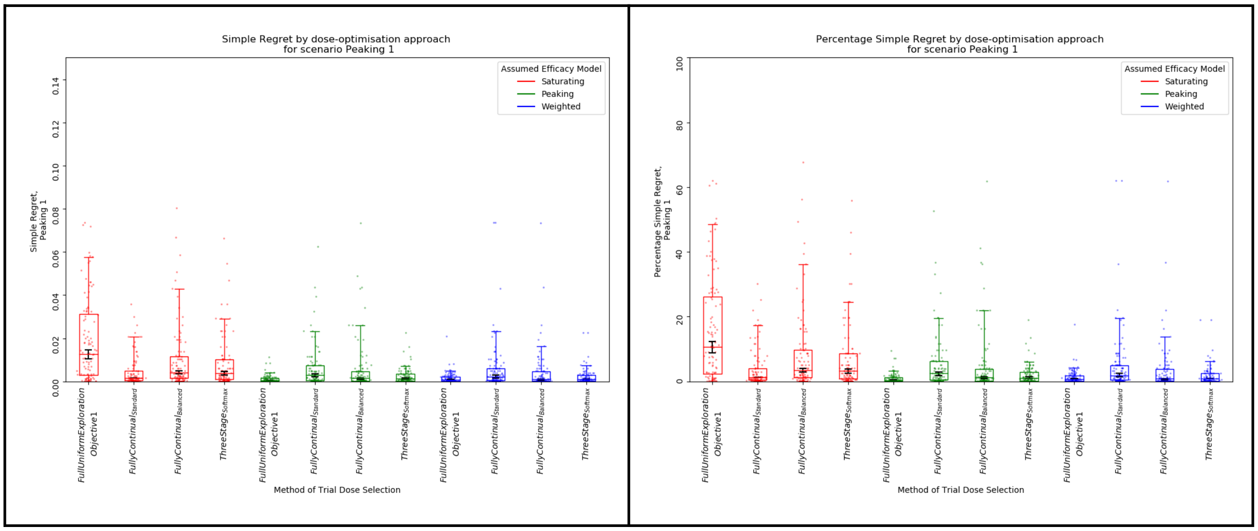

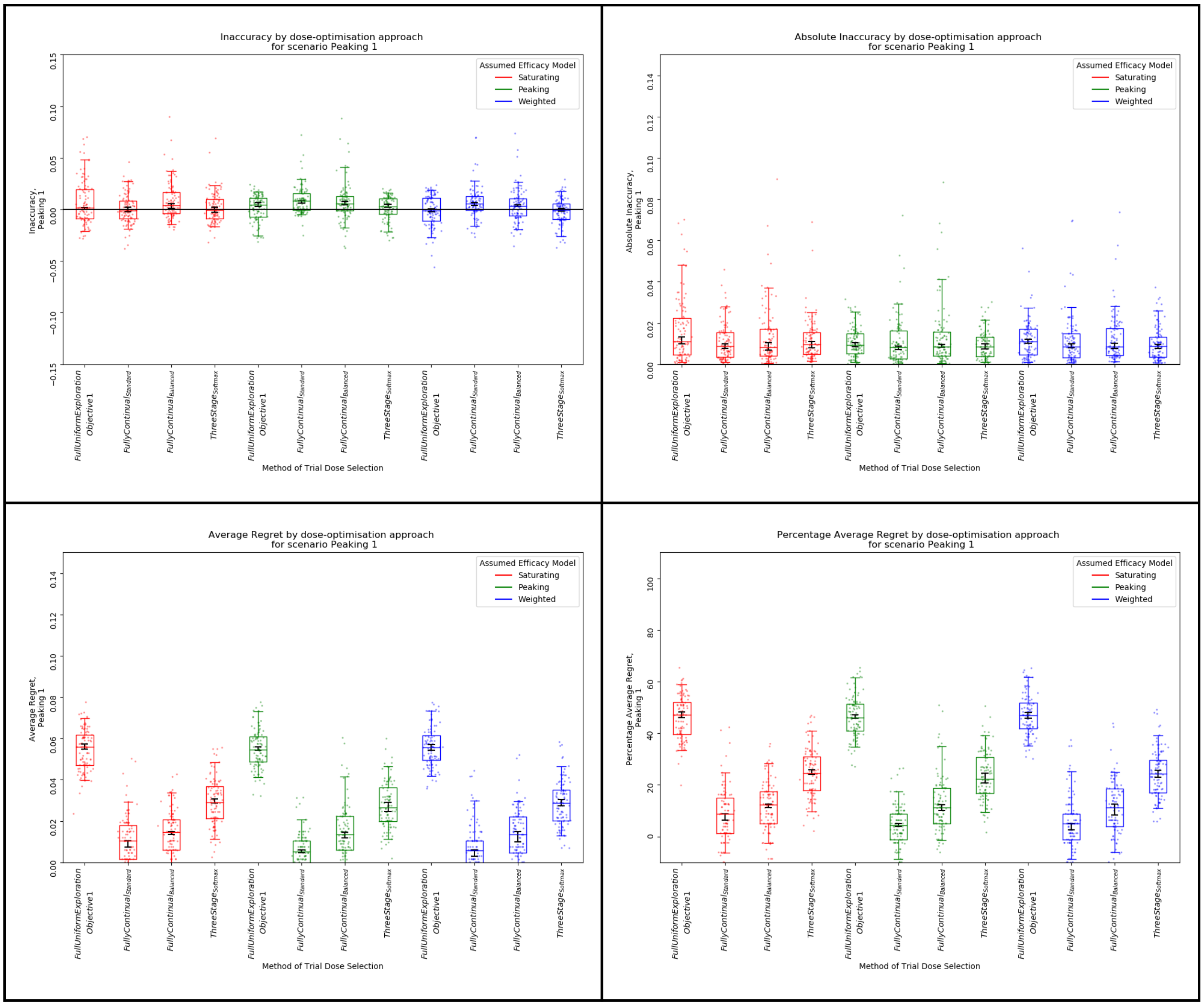

Figure Supplementary.Obj2_Plots.Peaking 1. Plots of the metrics from simulations for dose-optimisation approaches in objective 2 for scenario Peaking 1. The shown metrics are simple regret (top left), percentage simple regret (top right), inaccuracy (middle left), absolute inaccuracy (middle right), average regret (bottom left), and percentage average regret (bottom right).

Scenario Peaking 2

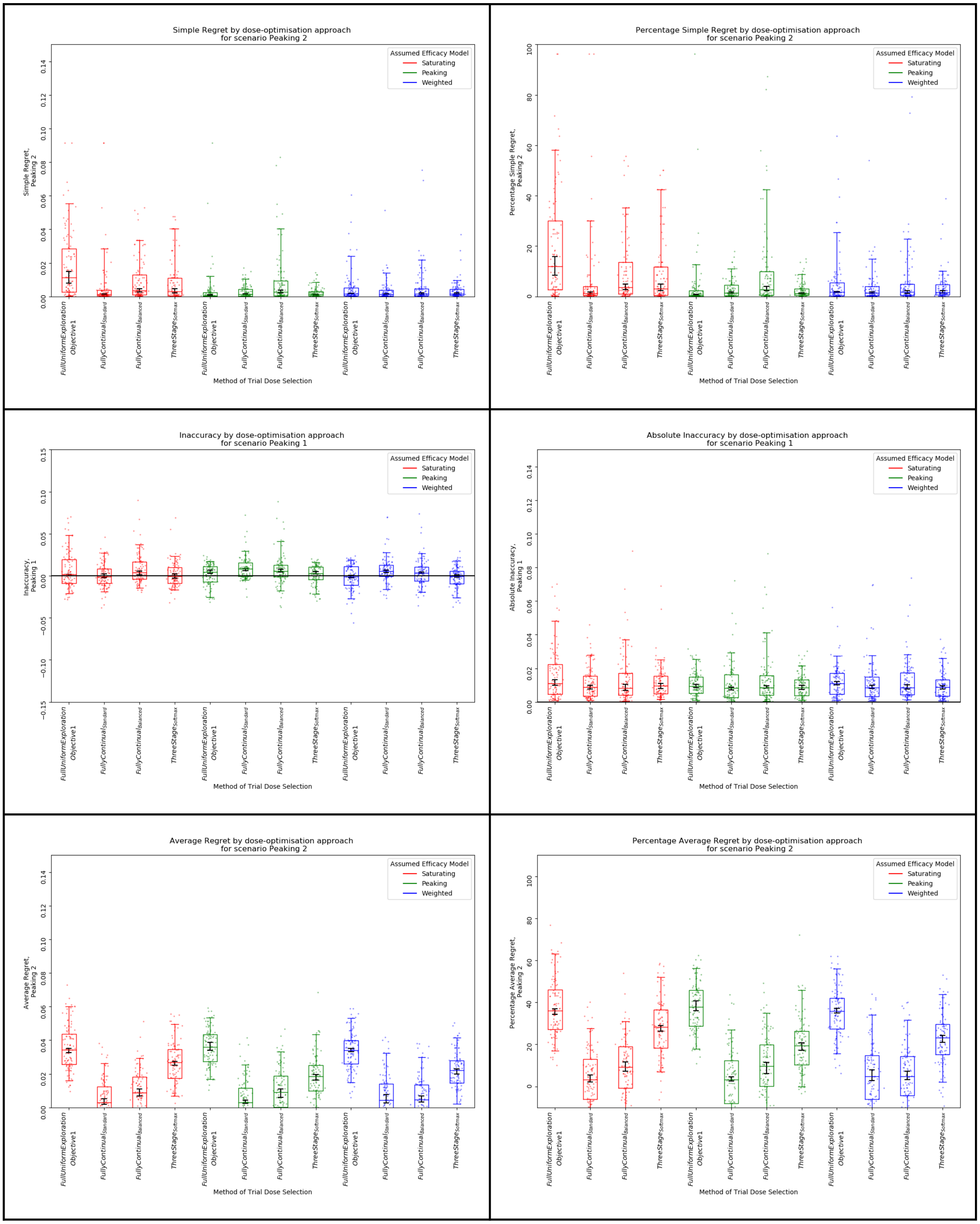

Figure Supplementary.Obj2_Plots.Peaking 2. Plots of the metrics from simulations for dose-optimisation approaches in objective 2 for scenario Peaking 2. The shown metrics are simple regret (top left), percentage simple regret (top right), inaccuracy (middle left), absolute

inaccuracy (middle right), average regret (bottom left), and percentage average regret (bottom right).

Scenario Peaking 3

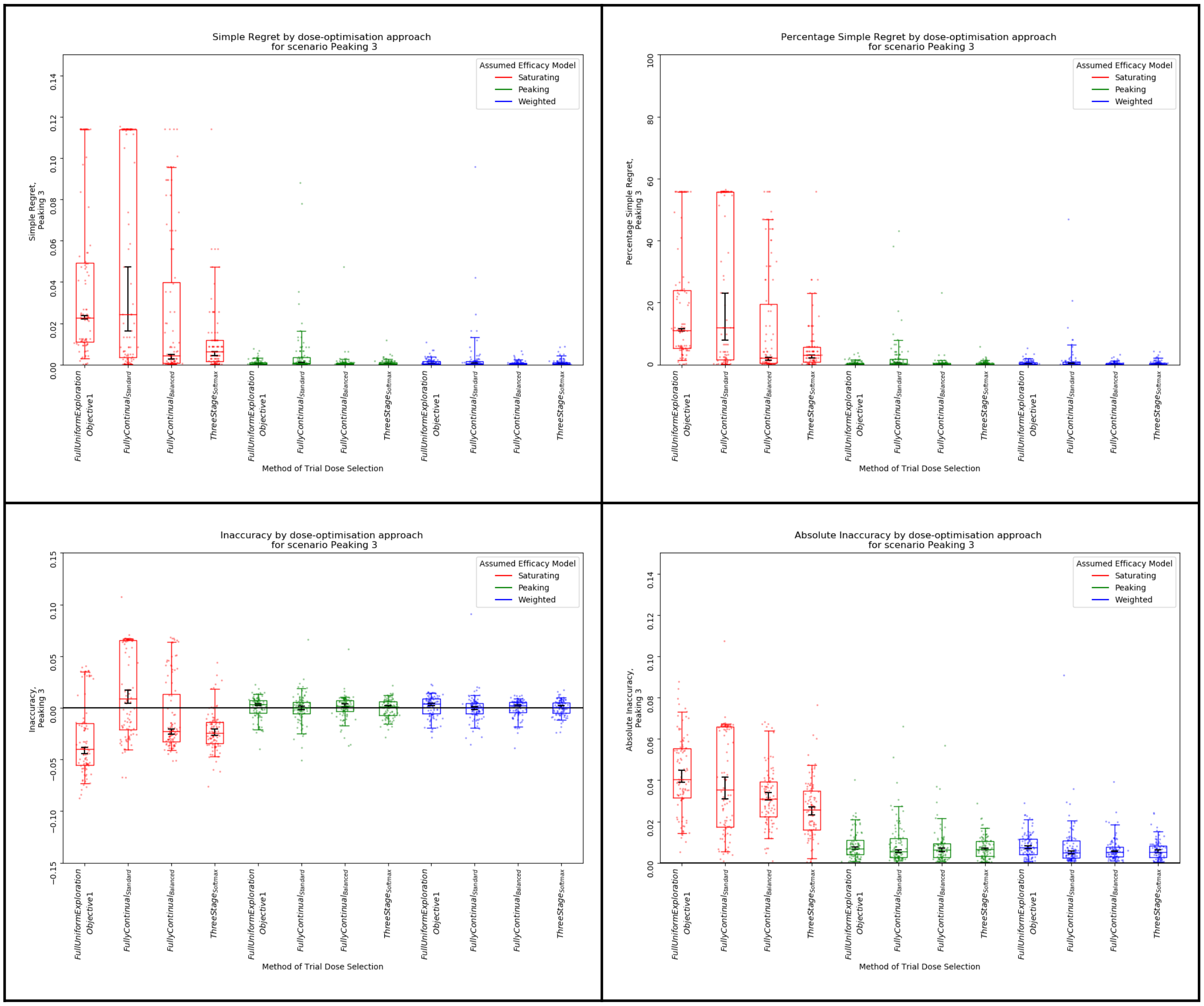

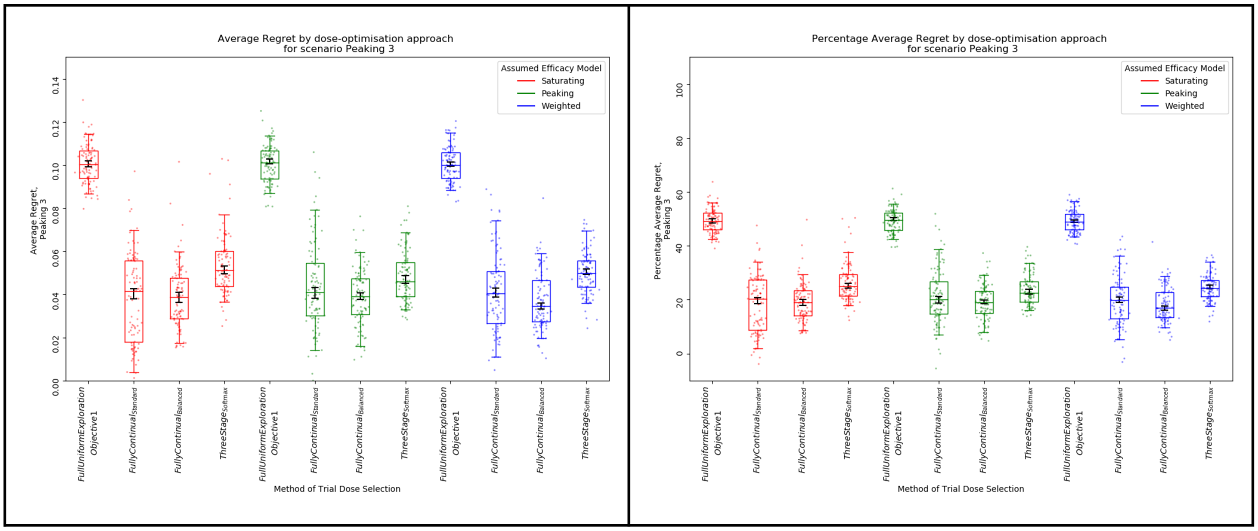

Figure Supplementary.Obj2_Plots.Peaking 3. Plots of the metrics from simulations for dose-optimisation approaches in objective 2 for scenario Peaking 3. The shown metrics are simple regret (top left), percentage simple regret (top right), inaccuracy (middle left), absolute inaccuracy (middle right), average regret (bottom left), and percentage average regret (bottom right).

Scenario Peaking 4

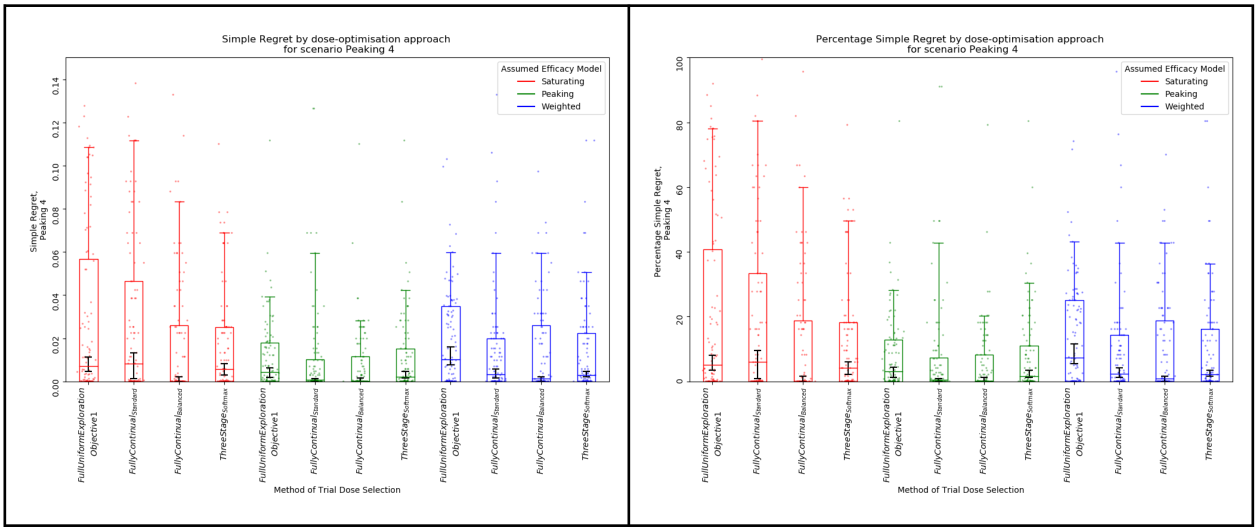

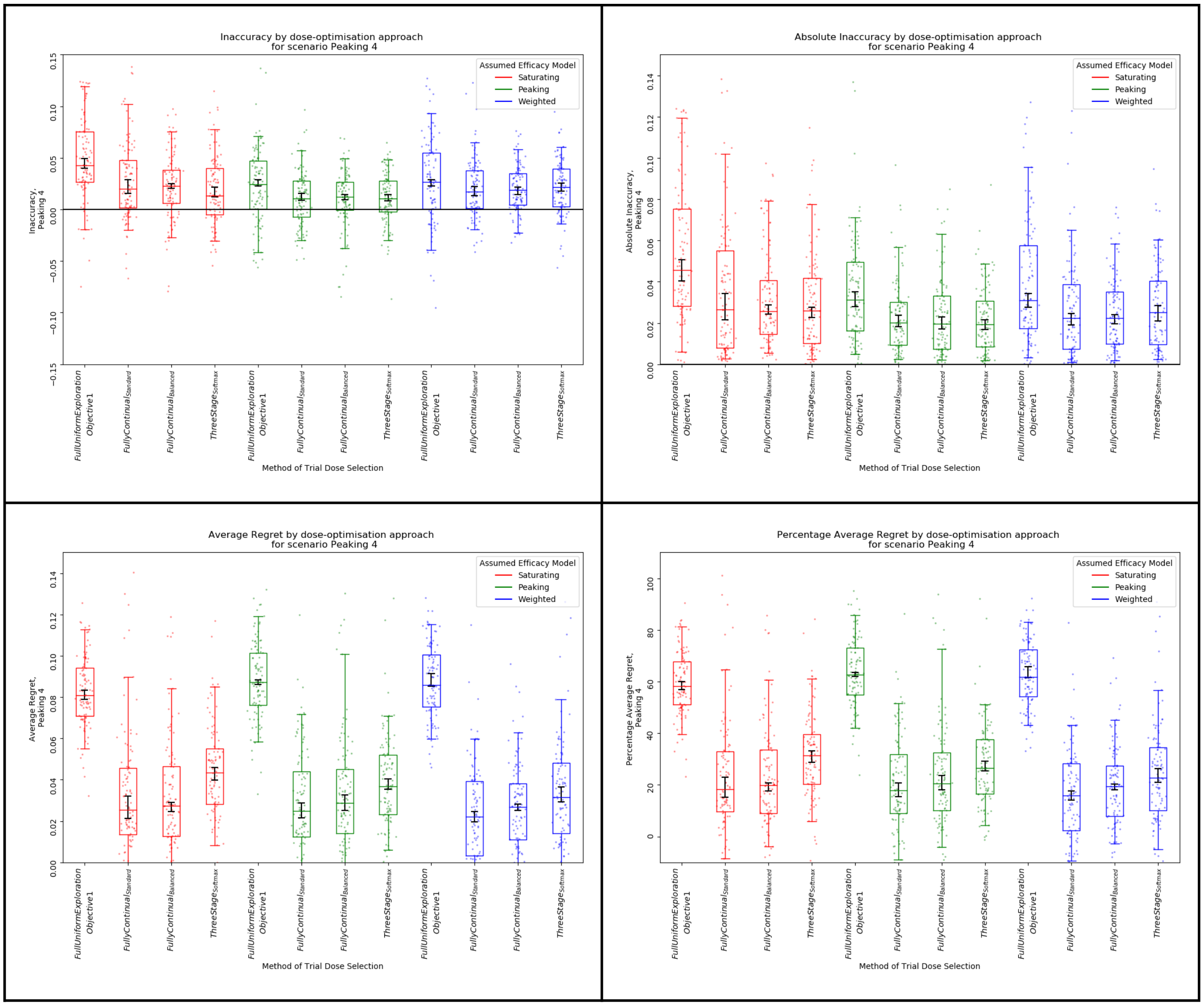

Figure Supplementary.Obj2_Plots.Peaking 4. Plots of the metrics from simulations for dose-optimisation approaches in objective 2 for scenario Peaking 4. The shown metrics are simple regret (top left), percentage simple regret (top right), inaccuracy (middle left), absolute inaccuracy (middle right), average regret (bottom left), and percentage average regret (bottom right).

Scenario Peaking 5

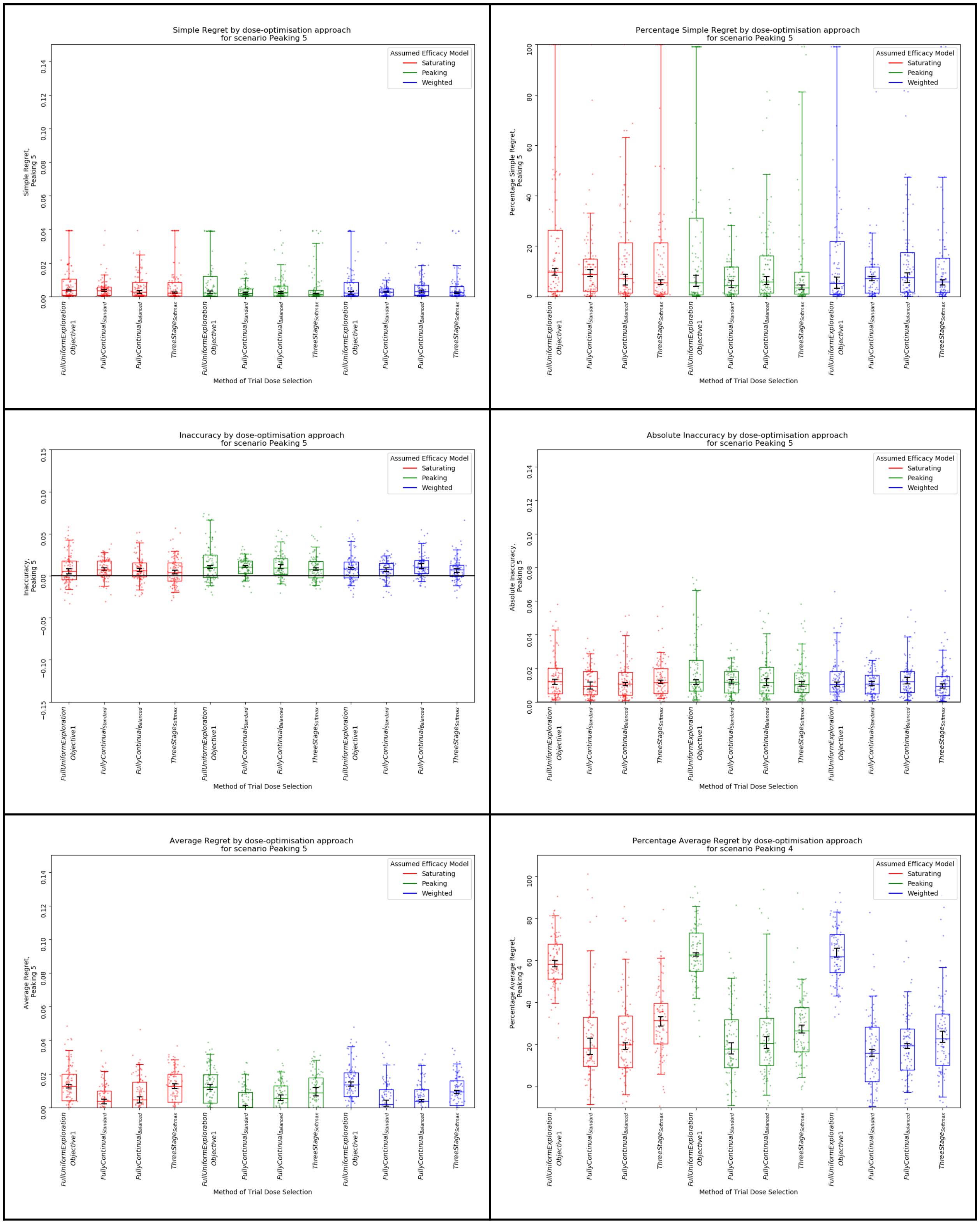

Figure Supplementary.Obj2_Plots.Peaking 5. Plots of the metrics from simulations for dose-optimisation approaches in objective 2 for scenario Peaking 5. The shown metrics are simple regret (top left), percentage simple regret (top right), inaccuracy (middle left), absolute

inaccuracy (middle right), average regret (bottom left), and percentage average regret (bottom right).

Scenario Other 1

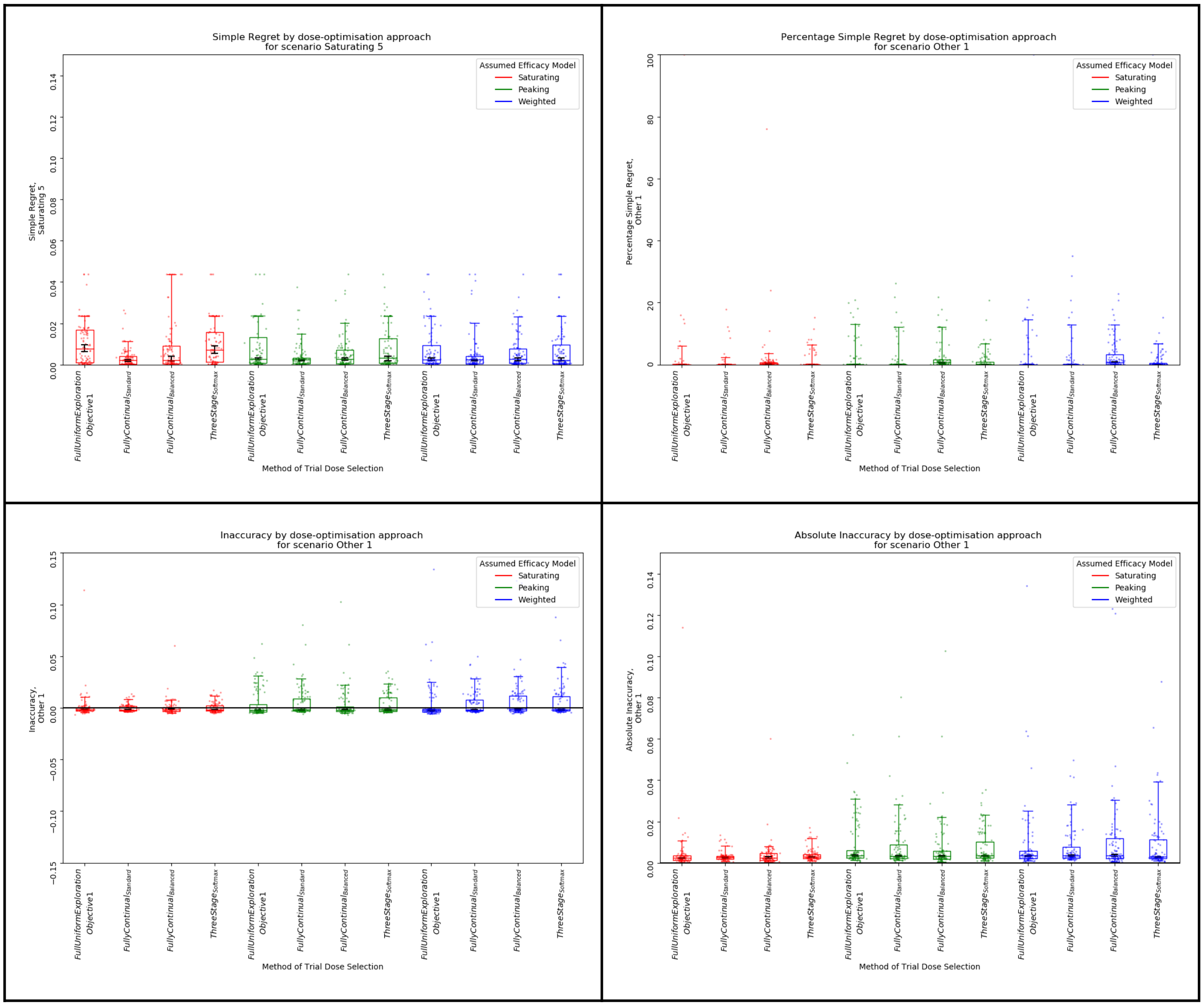

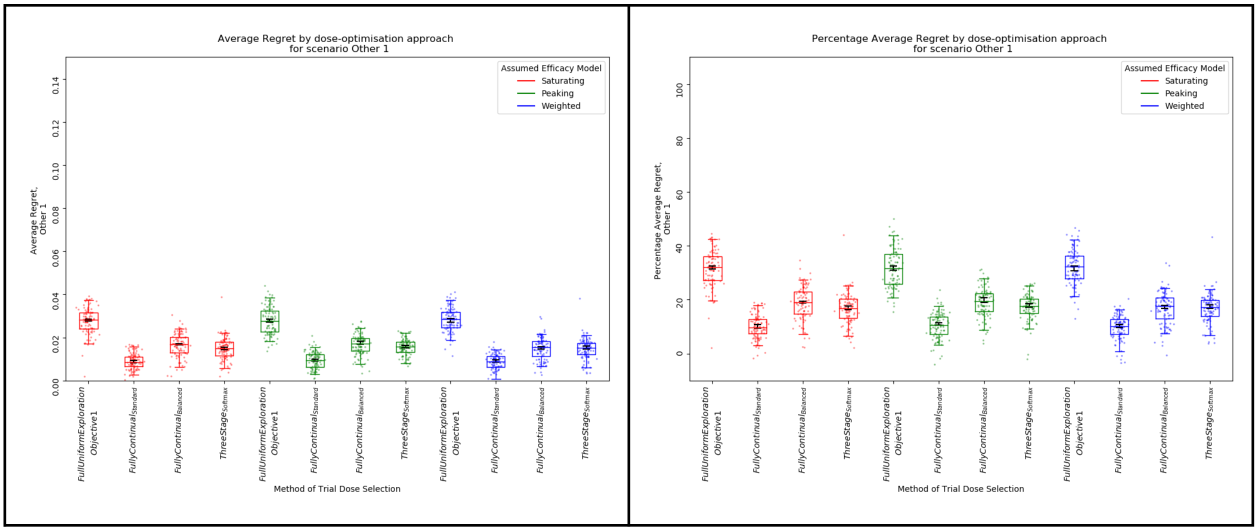

Figure Supplementary.Obj2_Plots.Other 1. Plots of the metrics from simulations for dose-optimisation approaches in objective 2 for scenario Other 1. The shown metrics are simple regret (top left), percentage simple regret (top right), inaccuracy (middle left), absolute inaccuracy (middle right), average regret (bottom left), and percentage average regret (bottom right).

Scenario Other 2

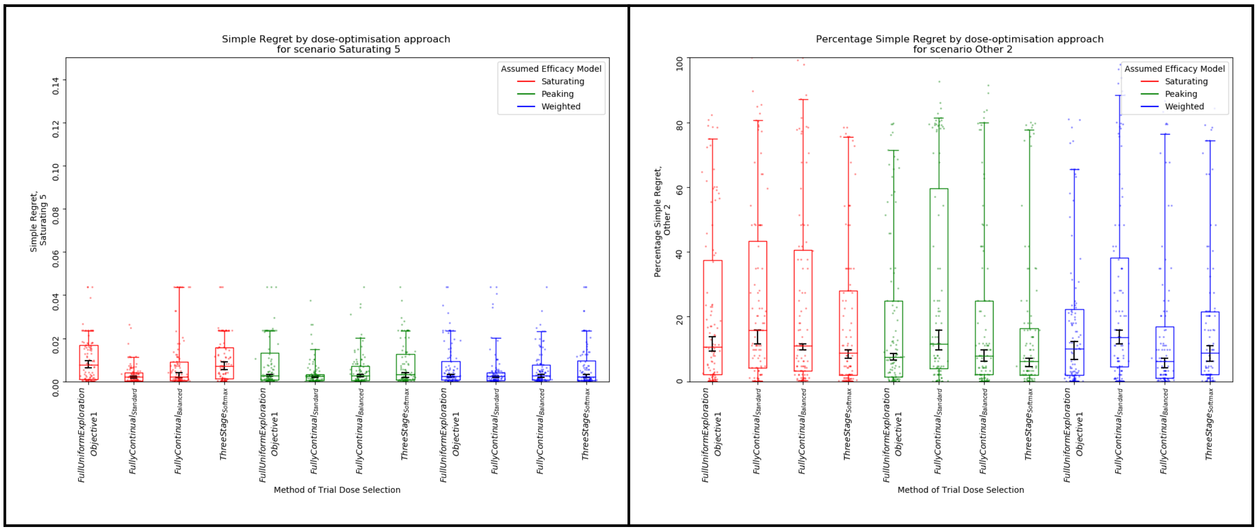

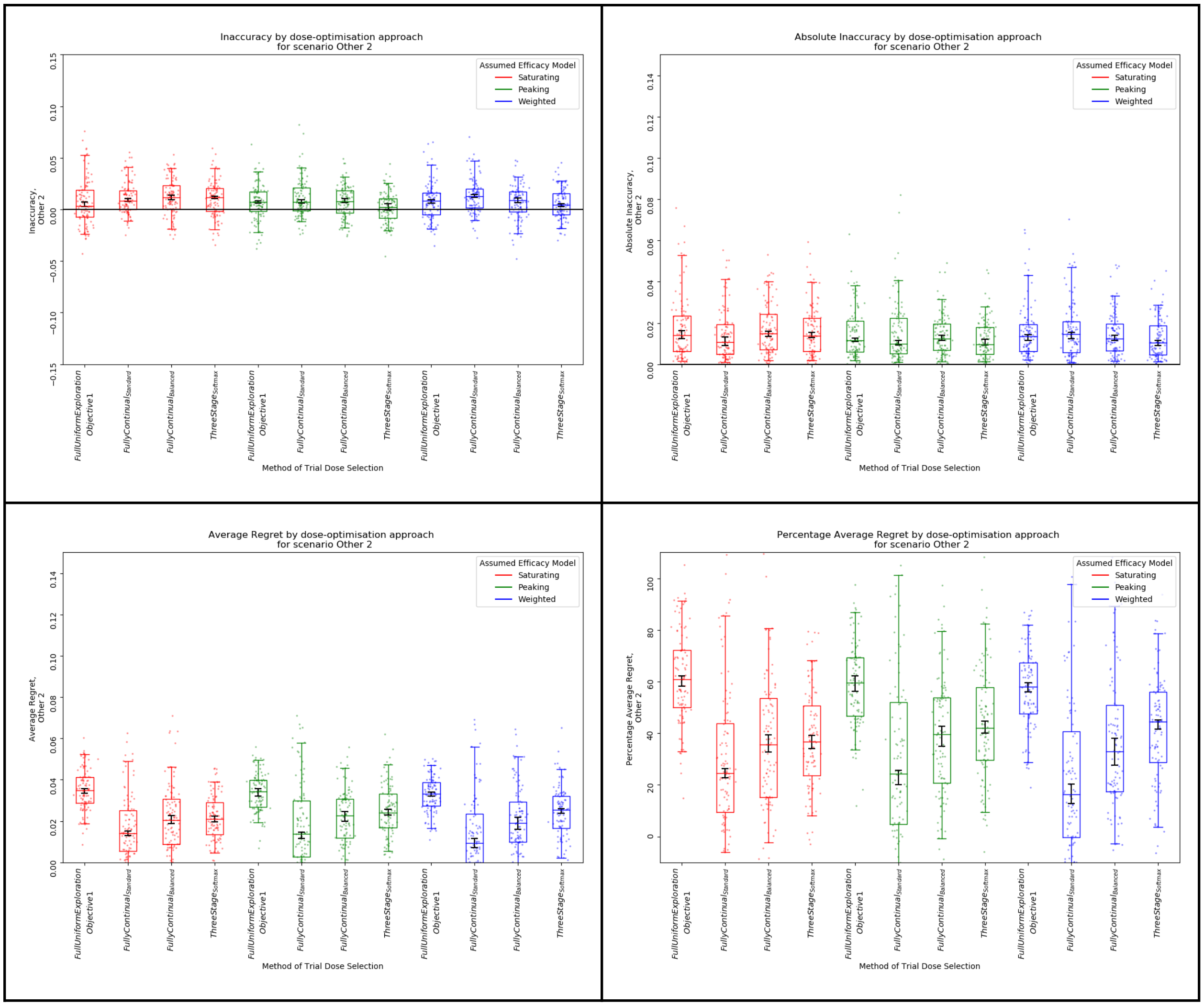

Figure Supplementary.Obj2_Plots.Other 2. Plots of the metrics from simulations for dose-optimisation approaches in objective 2 for scenario Other 2. The shown metrics are simple regret (top left), percentage simple regret (top right), inaccuracy (middle left), absolute inaccuracy (middle right), average regret (bottom left), and percentage average regret (bottom right).

Scenario Other 3

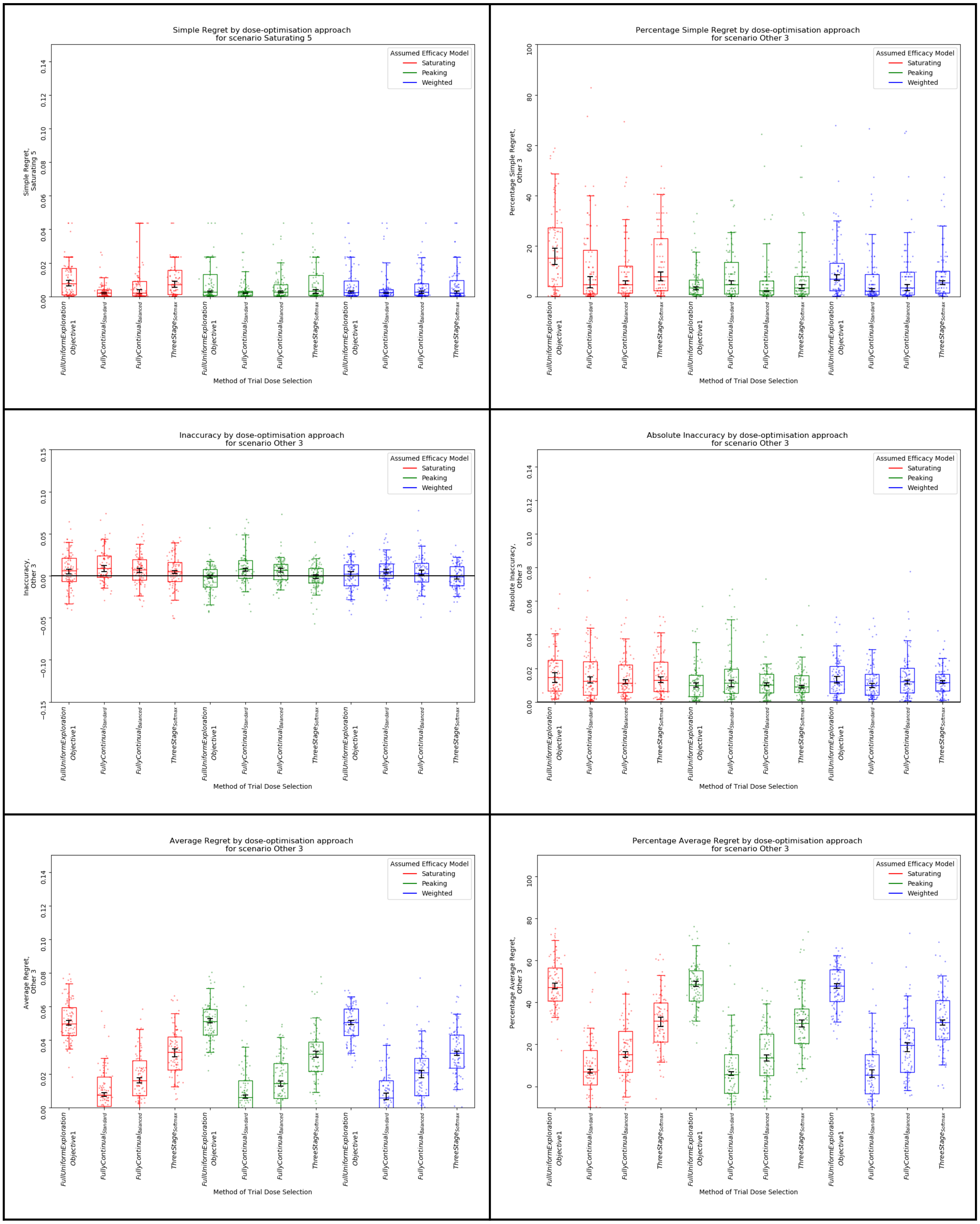

Figure Supplementary.Obj2_Plots.Other 3. Plots of the metrics from simulations for dose-optimisation approaches in objective 2 for scenario Other 3. The shown metrics are simple regret (top left), percentage simple regret (top right), inaccuracy (middle left), absolute

inaccuracy (middle right), average regret (bottom left), and percentage average regret (bottom right).

Scenario Other 4

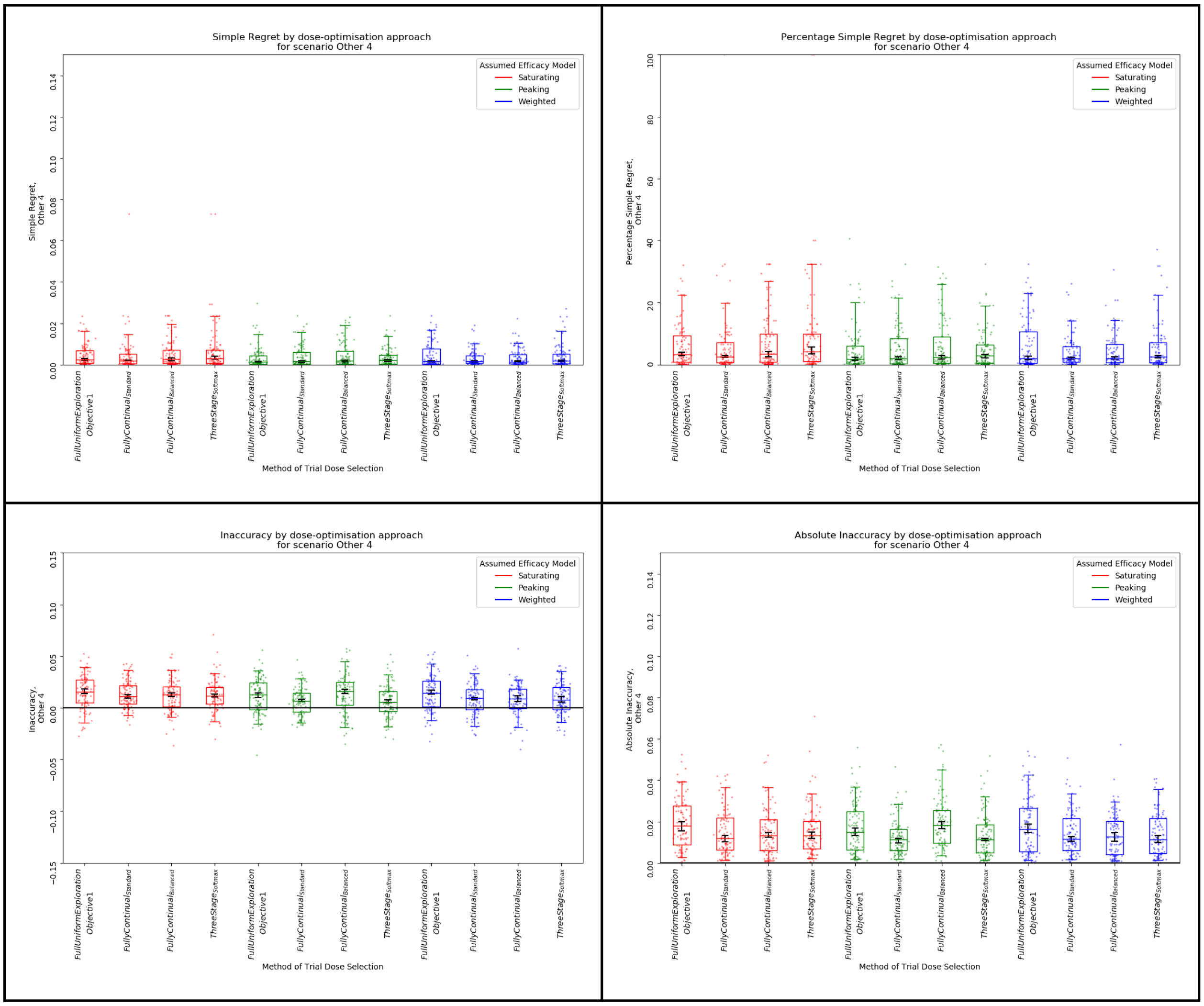

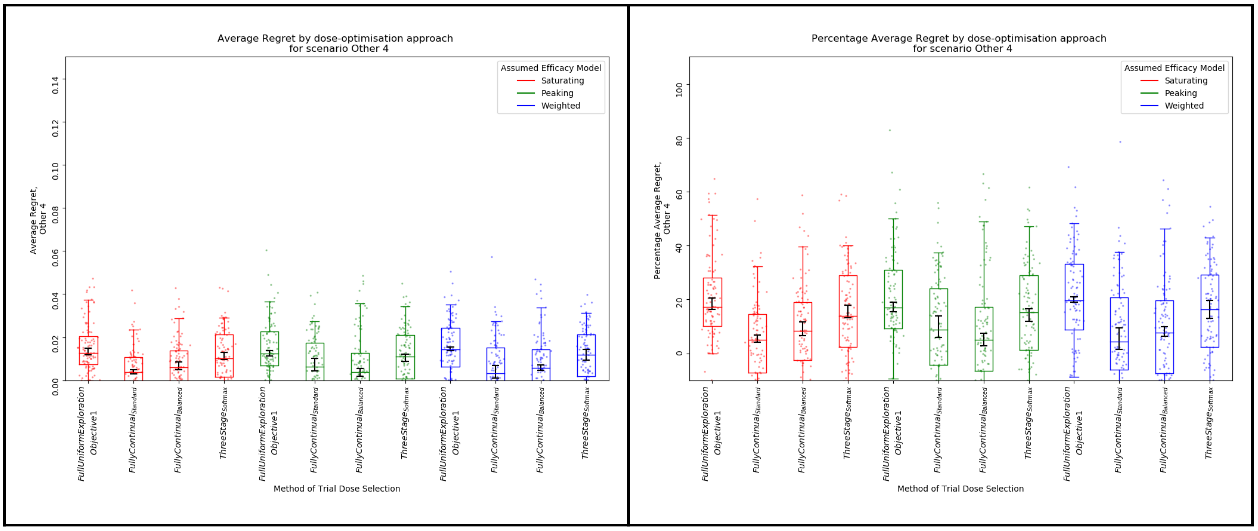

Figure Supplementary.Obj2_Plots.Other 4. Plots of the metrics from simulations for dose-optimisation approaches in objective 2 for scenario Other 4. The shown metrics are simple regret (top left), percentage simple regret (top right), inaccuracy (middle left), absolute inaccuracy (middle right), average regret (bottom left), and percentage average regret (bottom right).

Supplementary 14. Copeland Tables

Scenario Saturating 1

Rank	Approach	Score
**1**	Peaking CRM, Fully Continual, Standard	0.81
**2**	Weighted CRM, Fully Continual, Standard	0.772
**3**	Saturating CRM, Fully Continual, Standard	0.768
**4**	Saturating CRM, Fully Continual, Balanced	0.617
**5**	Peaking CRM, Fully Continual, Balanced	0.602
**6**	Weighted CRM, Fully Continual, Balanced	0.587
**7**	Peaking, Softmax 3 Step	0.523

**8**	Saturating, Softmax 3 Step	0.491
**9**	Weighted, Softmax 3 Step	0.482
**10**	Peaking Uniform, 30	0.128
**11**	Saturating Uniform, 30	0.113
**12**	Weighted Uniform, 30	0.107

Table S.Copelands.Saturating 1.Average Regret.

Rank	Approach	Score
**1**	Peaking CRM, Fully Continual, Balanced	0.582
**2**	Peaking, Softmax 3 Stage	0.57
**3**	Weighted, Softmax 3 Stage	0.558
**4**	Saturating CRM, Fully Continual, Standard	0.549
**5**	Saturating, Softmax 3 Stage	0.539
**6**	Weighted CRM, Fully Continual, Standard	0.528
**7**	Weighted CRM, Fully Continual, Balanced	0.514
**8**	Saturating CRM, Fully Continual, Balanced	0.492
**9**	Peaking CRM, Fully Continual, Standard	0.462
**10**	Peaking Uniform, 30	0.457
**11**	Weighted Uniform, 30	0.419
**12**	Saturating Uniform, 30	0.33

Table S.Copelands.Saturating 1.Absolute Inaccuracy.

Rank	Approach	Score
**1**	Peaking Uniform, 30	0.605
**2**	Peaking CRM, Fully Continual, Balanced	0.566
**3**	Weighted, Softmax 3 Step	0.562
**4**	Peaking, Softmax 3 Step	0.559
**5**	Weighted CRM, Fully Continual, Balanced	0.518

**6**	Weighted Uniform, 30	0.498
**7**	Saturating, Softmax 3 Step	0.486
**8**	Saturating CRM, Fully Continual, Balanced	0.464
**9**	Peaking CRM, Fully Continual, Standard	0.453
**10**	Saturating CRM, Fully Continual, Standard	0.431
**11**	Weighted CRM, Fully Continual, Standard	0.43
**12**	Saturating Uniform, 30	0.426

Table S.Copelands.Saturating 1.SR.

Scenario Saturating 2

Rank	Approach	Score
**1**	Peaking CRM, Fully Continual, Standard	0.806
**2**	Saturating CRM, Fully Continual, Standard	0.751
**3**	Weighted CRM, Fully Continual, Standard	0.741
**4**	Peaking CRM, Fully Continual, Balanced	0.708
**5**	Weighted CRM, Fully Continual, Balanced	0.692
**6**	Saturating CRM, Fully Continual, Balanced	0.681
**7**	Weighted, Softmax 3 Step	0.46
**8**	Saturating, Softmax 3 Step	0.406
**9**	Peaking, Softmax 3 Step	0.403
**10**	Peaking Uniform, 30	0.123
**11**	Weighted Uniform, 30	0.116
**12**	Saturating Uniform, 30	0.111

Table S.Copelands.Saturating 2.Average Regret.

Rank	Approach	Score
**1**	Peaking, Softmax 3 Stage	0.591
**2**	Saturating CRM, Fully Continual, Balanced	0.58
**3**	Weighted, Softmax 3 Stage	0.537
**4**	Weighted Uniform, 30	0.519
**5**	Saturating, Softmax 3 Stage	0.51
**6**	Weighted CRM, Fully Continual, Balanced	0.508
**7**	Peaking CRM, Fully Continual, Balanced	0.489
**8**	Weighted CRM, Fully Continual, Standard	0.479
**9**	Peaking CRM, Fully Continual, Standard	0.475
**10**	Saturating CRM, Fully Continual, Standard	0.471
**11**	Saturating Uniform, 30	0.44
**12**	Peaking Uniform, 30	0.403

Table S.Copelands.Saturating 2.Absolute Inaccuracy.

Rank	Approach	Score
**1**	Weighted CRM, Fully Continual, Balanced	0.622
**2**	Weighted, Softmax 3 Step	0.573
**3**	Peaking Uniform, 30	0.55
**4**	Peaking, Softmax 3 Step	0.544
**5**	Peaking CRM, Fully Continual, Standard	0.518
**6**	Weighted Uniform, 30	0.51
**7**	Saturating CRM, Fully Continual, Balanced	0.494
**8**	Peaking CRM, Fully Continual, Balanced	0.483

**9**	Saturating CRM, Fully Continual, Standard	0.468
**10**	Saturating Uniform, 30	0.434
**11**	Saturating, Softmax 3 Step	0.412
**12**	Weighted CRM, Fully Continual, Standard	0.393

Table S.Copelands.Saturating 2.SR.

Scenario Saturating 3

Rank	Approach	Score
**1**	Weighted, Softmax 3 Stage	0.58
**2**	Peaking, Softmax 3 Stage	0.543
**3**	Saturating CRM, Fully Continual, Balanced	0.541
**4**	Saturating, Softmax 3 Stage	0.53
**5**	Peaking CRM, Fully Continual, Balanced	0.529
**6**	Weighted CRM, Fully Continual, Balanced	0.527
**7**	Weighted Uniform, 30	0.512
**8**	Peaking CRM, Fully Continual, Standard	0.498
**9**	Peaking Uniform, 30	0.49
**10**	Saturating CRM, Fully Continual, Standard	0.462
**11**	Saturating Uniform, 30	0.407
**12**	Weighted CRM, Fully Continual, Standard	0.38

Table S.Copelands.Saturating 3.Average Regret.

Rank	Approach	Score
**1**	Weighted, Softmax 3 Step	0.58
**2**	Peaking, Softmax 3 Step	0.543

**3**	Saturating CRM, Fully Continual, Balanced	0.541
**4**	Saturating, Softmax 3 Step	0.53
**5**	Peaking CRM, Fully Continual, Balanced	0.529
**6**	Weighted CRM, Fully Continual, Balanced	0.527
**7**	Weighted Uniform, 30	0.512
**8**	Peaking CRM, Fully Continual, Standard	0.498
**9**	Peaking Uniform, 30	0.49
**10**	Saturating CRM, Fully Continual, Standard	0.462
**11**	Saturating Uniform, 30	0.407
**12**	Weighted CRM, Fully Continual, Standard	0.38

Table S.Copelands.Saturating 3.Absolute Inaccuracy.

Rank	Approach	Score
**1**	Weighted, Softmax 3 Step	0.637
**2**	Weighted Uniform, 30	0.608
**3**	Peaking, Softmax 3 Step	0.577
**4**	Peaking CRM, Fully Continual, Balanced	0.576
**5**	Saturating, Softmax 3 Step	0.553
**6**	Weighted CRM, Fully Continual, Balanced	0.537
**7**	Peaking Uniform, 30	0.525
**8**	Saturating CRM, Fully Continual, Balanced	0.517
**9**	Saturating Uniform, 30	0.473
**10**	Peaking CRM, Fully Continual, Standard	0.383
**11**	Weighted CRM, Fully Continual, Standard	0.317
**12**	Saturating CRM, Fully	0.297

	Continual, Standard	

Table S.Copelands.Saturating 3.SR.

Scenario Saturating 4

Rank	Approach	Score
**1**	Saturating CRM, Fully Continual, Standard	0.71
**2**	Peaking CRM, Fully Continual, Standard	0.654
**3**	Weighted CRM, Fully Continual, Standard	0.623
**4**	Peaking, Softmax 3 Step	0.614
**5**	Peaking CRM, Fully Continual, Balanced	0.61
**6**	Weighted, Softmax 3 Step	0.575
**7**	Saturating CRM, Fully Continual, Balanced	0.574
**8**	Weighted CRM, Fully Continual, Balanced	0.565
**9**	Saturating, Softmax 3 Step	0.516
**10**	Saturating Uniform, 30	0.192
**11**	Weighted Uniform, 30	0.189
**12**	Peaking Uniform, 30	0.178

Table S.Copelands.Saturating 4.Average Regret.

Rank	Approach	Score
**1**	Weighted CRM, Fully Continual, Balanced	0.58
**2**	Peaking, Softmax 3 Stage	0.566
**3**	Peaking CRM, Fully Continual, Standard	0.54
**4**	Weighted CRM, Fully Continual, Standard	0.528

**5**	Peaking CRM, Fully Continual, Balanced	0.527
**6**	Saturating CRM, Fully Continual, Balanced	0.514
**7**	Saturating, Softmax 3 Stage	0.5
**8**	Weighted, Softmax 3 Stage	0.487
**9**	Saturating CRM, Fully Continual, Standard	0.472
**10**	Weighted Uniform, 30	0.464
**11**	Peaking Uniform, 30	0.444
**12**	Saturating Uniform, 30	0.379

Table S.Copelands.Saturating 4.Absolute Inaccuracy.

Rank	Approach	Score
**1**	Peaking Uniform, 30	0.595
**2**	Peaking, Softmax 3 Step	0.576
**3**	Peaking CRM, Fully Continual, Balanced	0.552
**4**	Weighted Uniform, 30	0.518
**5**	Weighted CRM, Fully Continual, Balanced	0.517
**6**	Saturating, Softmax 3 Step	0.493
**7**	Weighted, Softmax 3 Step	0.491
**8**	Saturating CRM, Fully Continual, Standard	0.484
**9**	Saturating CRM, Fully Continual, Balanced	0.467
**10**	Saturating Uniform, 30	0.445
**11**	Peaking CRM, Fully Continual, Standard	0.439
**12**	Weighted CRM, Fully Continual, Standard	0.423

Table S.Copelands.Saturating 4.SR.

Scenario Saturating 5

Rank	Approach	Score
**1**	Peaking Uniform, 30	0.595
**2**	Peaking, Softmax 3 Stage	0.576
**3**	Peaking CRM, Fully Continual, Balanced	0.552
**4**	Weighted Uniform, 30	0.518
**5**	Weighted CRM, Fully Continual, Balanced	0.517
**6**	Saturating, Softmax 3 Stage	0.493
**7**	Weighted, Softmax 3 Stage	0.491
**8**	Saturating CRM, Fully Continual, Standard	0.484
**9**	Saturating CRM, Fully Continual, Balanced	0.467
**10**	Saturating Uniform, 30	0.445
**11**	Peaking CRM, Fully Continual, Standard	0.439
**12**	Weighted CRM, Fully Continual, Standard	0.423

Table S.Copelands.Saturating 5.Average Regret.

Rank	Approach	Score
**1**	Saturating CRM, Fully Continual, Standard	0.614
**2**	Saturating, Softmax 3 Stage	0.532
**3**	Weighted, Softmax 3 Stage	0.524
**4**	Peaking, Softmax 3 Stage	0.517
**5**	Weighted CRM, Fully Continual, Standard	0.509
**6**	Saturating CRM, Fully Continual, Balanced	0.504
**7**	Weighted CRM, Fully Continual, Balanced	0.493
**8**	Peaking Uniform, 30	0.485
**9**	Saturating Uniform, 30	0.478
**10**	Peaking CRM, Fully	0.466

	Continual, Standard	
**11**	Weighted Uniform, 30	0.463
**12**	Peaking CRM, Fully Continual, Balanced	0.415

Table S.Copelands.Saturating 5.Absolute Inaccuracy.

Rank	Approach	Score
**1**	Saturating CRM, Fully Continual, Standard	0.596
**2**	Peaking CRM, Fully Continual, Standard	0.578
**3**	Weighted CRM, Fully Continual, Standard	0.567
**4**	Peaking CRM, Fully Continual, Balanced	0.528
**5**	Weighted CRM, Fully Continual, Balanced	0.518
**6**	Weighted, Softmax 3 Step	0.507
**7**	Saturating CRM, Fully Continual, Balanced	0.49
**8**	Weighted Uniform, 30	0.48
**9**	Peaking, Softmax 3 Step	0.477
**10**	Peaking Uniform, 30	0.476
**11**	Saturating Uniform, 30	0.394
**12**	Saturating, Softmax 3 Step	0.389

Table S.Copelands.Saturating 5.SR.

Scenario Peaking 1

Rank	Approach	Score
**1**	Peaking CRM, Fully Continual, Standard	0.816
**2**	Weighted CRM, Fully Continual, Standard	0.765
**3**	Saturating CRM, Fully Continual, Standard	0.727

**4**	Weighted CRM, Fully Continual, Balanced	0.686
**5**	Saturating CRM, Fully Continual, Balanced	0.676
**6**	Peaking CRM, Fully Continual, Balanced	0.676
**7**	Peaking, Softmax 3 Step	0.451
**8**	Weighted, Softmax 3 Step	0.433
**9**	Saturating, Softmax 3 Step	0.427
**10**	Saturating Uniform, 30	0.119
**11**	Peaking Uniform, 30	0.115
**12**	Weighted Uniform, 30	0.108

Table S.Copelands.Peaking 1.Average Regret.

Rank	Approach	Score
**1**	Weighted, Softmax 3 Stage	0.546
**2**	Peaking, Softmax 3 Stage	0.544
**3**	Peaking CRM, Fully Continual, Standard	0.522
**4**	Saturating CRM, Fully Continual, Standard	0.513
**5**	Weighted CRM, Fully Continual, Standard	0.508
**6**	Peaking CRM, Fully Continual, Balanced	0.508
**7**	Saturating CRM, Fully Continual, Balanced	0.503
**8**	Peaking Uniform, 30	0.497
**9**	Saturating, Softmax 3 Stage	0.492
**10**	Weighted CRM, Fully Continual, Balanced	0.486
**11**	Weighted Uniform, 30	0.469
**12**	Saturating Uniform, 30	0.412

Table S.Copelands.Peaking 1.Absolute Inaccuracy.

Rank	Approach	Score

**1**	Peaking Uniform, 30	0.701
**2**	Weighted Uniform, 30	0.636
**3**	Weighted, Softmax 3 Step	0.618
**4**	Weighted CRM, Fully Continual, Balanced	0.592
**5**	Peaking, Softmax 3 Step	0.558
**6**	Peaking CRM, Fully Continual, Balanced	0.508
**7**	Saturating CRM, Fully Continual, Standard	0.505
**8**	Peaking CRM, Fully Continual, Standard	0.452
**9**	Weighted CRM, Fully Continual, Standard	0.449
**10**	Saturating, Softmax 3 Step	0.384
**11**	Saturating CRM, Fully Continual, Balanced	0.352
**12**	Saturating Uniform, 30	0.244

Table S.Copelands.Peaking 1.SR.

Scenario Peaking 2

Rank	Approach	Score
**1**	Saturating CRM, Fully Continual, Standard	0.739
**2**	Peaking CRM, Fully Continual, Standard	0.733
**3**	Weighted CRM, Fully Continual, Balanced	0.721
**4**	Weighted CRM, Fully Continual, Standard	0.706
**5**	Saturating CRM, Fully Continual, Balanced	0.652
**6**	Peaking CRM, Fully Continual, Balanced	0.644

**7**	Peaking, Softmax 3 Step	0.486
**8**	Weighted, Softmax 3 Step	0.42
**9**	Saturating, Softmax 3 Step	0.338
**10**	Weighted Uniform, 30	0.203
**11**	Saturating Uniform, 30	0.19
**12**	Peaking Uniform, 30	0.169

Table S.Copelands.Peaking 2.Average Regret.

Rank	Approach	Score
**1**	Weighted, Softmax 3 Stage	0.595
**2**	Peaking, Softmax 3 Stage	0.569
**3**	Weighted CRM, Fully Continual, Standard	0.516
**4**	Saturating CRM, Fully Continual, Standard	0.515
**5**	Peaking CRM, Fully Continual, Standard	0.515
**6**	Saturating, Softmax 3 Stage	0.514
**7**	Weighted Uniform, 30	0.485
**8**	Weighted CRM, Fully Continual, Balanced	0.484
**9**	Peaking Uniform, 30	0.48
**10**	Saturating CRM, Fully Continual, Balanced	0.475
**11**	Peaking CRM, Fully Continual, Balanced	0.447
**12**	Saturating Uniform, 30	0.406

Table S.Copelands.Peaking 2.Absolute Inaccuracy.

Rank	Approach	Score
**1**	Peaking Uniform, 30	0.655
**2**	Peaking, Softmax 3 Step	0.576
**3**	Weighted CRM, Fully Continual, Standard	0.569
**4**	Peaking CRM, Fully Continual, Standard	0.559

**5**	Saturating CRM, Fully Continual, Standard	0.546
**6**	Weighted, Softmax 3 Step	0.522
**7**	Weighted Uniform, 30	0.518
**8**	Weighted CRM, Fully Continual, Balanced	0.517
**9**	Saturating, Softmax 3 Step	0.437
**10**	Peaking CRM, Fully Continual, Balanced	0.437
**11**	Saturating CRM, Fully Continual, Balanced	0.393
**12**	Saturating Uniform, 30	0.269

Table S.Copelands.Peaking 2.SR.

Scenario Peaking 3

Rank	Approach	Score
**1**	Weighted CRM, Fully Continual, Balanced	0.738
**2**	Saturating CRM, Fully Continual, Balanced	0.708
**3**	Peaking CRM, Fully Continual, Balanced	0.699
**4**	Saturating CRM, Fully Continual, Standard	0.693
**5**	Weighted CRM, Fully Continual, Standard	0.675
**6**	Peaking CRM, Fully Continual, Standard	0.646
**7**	Peaking, Softmax 3 Step	0.559
**8**	Weighted, Softmax 3 Step	0.516
**9**	Saturating, Softmax 3 Step	0.482
**10**	Weighted Uniform, 30	0.097
**11**	Saturating Uniform, 30	0.095
**12**	Peaking Uniform, 30	0.092

Table S.Copelands.Peaking 3.Average Regret.

Rank	Approach	Score
**1**	Weighted, Softmax 3 Stage	0.703
**2**	Weighted CRM, Fully Continual, Balanced	0.695
**3**	Weighted CRM, Fully Continual, Standard	0.682
**4**	Peaking CRM, Fully Continual, Balanced	0.647
**5**	Peaking CRM, Fully Continual, Standard	0.64
**6**	Peaking, Softmax 3 Stage	0.637
**7**	Peaking Uniform, 30	0.622
**8**	Weighted Uniform, 30	0.603
**9**	Saturating, Softmax 3 Stage	0.272
**10**	Saturating CRM, Fully Continual, Standard	0.194
**11**	Saturating CRM, Fully Continual, Balanced	0.188
**12**	Saturating Uniform, 30	0.117

Table S.Copelands.Peaking 3.Absolute Inaccuracy.

Rank	Approach	Score
**1**	Weighted CRM, Fully Continual, Balanced	0.7
**2**	Peaking CRM, Fully Continual, Balanced	0.671
**3**	Weighted, Softmax 3 Step	0.654
**4**	Peaking, Softmax 3 Step	0.647
**5**	Peaking Uniform, 30	0.636
**6**	Weighted Uniform, 30	0.623
**7**	Weighted CRM, Fully Continual, Standard	0.597
**8**	Peaking CRM, Fully Continual, Standard	0.511

**9**	Saturating CRM, Fully Continual, Balanced	0.339
**10**	Saturating, Softmax 3 Step	0.298
**11**	Saturating CRM, Fully Continual, Standard	0.206
**12**	Saturating Uniform, 30	0.118

Table S.Copelands.Peaking 3.SR.

Scenario Peaking 4

Rank	Approach	Score
**1**	Weighted CRM, Fully Continual, Standard	0.711
**2**	Weighted CRM, Fully Continual, Balanced	0.677
**3**	Peaking CRM, Fully Continual, Standard	0.658
**4**	Saturating CRM, Fully Continual, Balanced	0.633
**5**	Saturating CRM, Fully Continual, Standard	0.633
**6**	Peaking CRM, Fully Continual, Balanced	0.62
**7**	Weighted, Softmax 3 Step	0.605
**8**	Peaking, Softmax 3 Step	0.552
**9**	Saturating, Softmax 3 Step	0.5
**10**	Saturating Uniform, 30	0.159
**11**	Peaking Uniform, 30	0.127
**12**	Weighted Uniform, 30	0.127

Table S.Copelands.Peaking 4.Average Regret.

Rank	Approach	Score
**1**	Peaking, Softmax 3 Stage	0.605
**2**	Peaking CRM, Fully Continual, Balanced	0.593
**3**	Peaking CRM, Fully	0.585

	Continual, Standard	
**4**	Weighted CRM, Fully Continual, Standard	0.562
**5**	Weighted CRM, Fully Continual, Balanced	0.562
**6**	Weighted, Softmax 3 Stage	0.519
**7**	Saturating, Softmax 3 Stage	0.5
**8**	Saturating CRM, Fully Continual, Standard	0.488
**9**	Saturating CRM, Fully Continual, Balanced	0.474
**10**	Peaking Uniform, 30	0.431
**11**	Weighted Uniform, 30	0.41
**12**	Saturating Uniform, 30	0.27

Table S.Copelands.Peaking 4.Absolute Inaccuracy.

Rank	Approach	Score
**1**	Peaking CRM, Fully Continual, Balanced	0.593
**2**	Peaking CRM, Fully Continual, Standard	0.577
**3**	Saturating CRM, Fully Continual, Balanced	0.536
**4**	Weighted CRM, Fully Continual, Balanced	0.53
**5**	Peaking, Softmax 3 Step	0.514
**6**	Weighted CRM, Fully Continual, Standard	0.511
**7**	Weighted, Softmax 3 Step	0.495
**8**	Peaking Uniform, 30	0.494
**9**	Saturating, Softmax 3 Step	0.475
**10**	Saturating CRM, Fully Continual, Standard	0.444
**11**	Weighted Uniform, 30	0.418
**12**	Saturating Uniform, 30	0.413

Table S.Copelands.Peaking 4.SR.

Scenario Peaking 5

Rank	Approach	Score
**1**	Peaking CRM, Fully Continual, Standard	0.65
**2**	Weighted CRM, Fully Continual, Standard	0.615
**3**	Saturating CRM, Fully Continual, Standard	0.596
**4**	Weighted CRM, Fully Continual, Balanced	0.571
**5**	Peaking CRM, Fully Continual, Balanced	0.564
**6**	Saturating CRM, Fully Continual, Balanced	0.546
**7**	Peaking, Softmax 3 Step	0.47
**8**	Weighted, Softmax 3 Step	0.464
**9**	Peaking Uniform, 30	0.405
**10**	Saturating, Softmax 3 Step	0.396
**11**	Saturating Uniform, 30	0.389
**12**	Weighted Uniform, 30	0.334

Table S.Copelands.Peaking 5.Average Regret.

Rank	Approach	Score
**1**	Weighted, Softmax 3 Stage	0.556
**2**	Weighted CRM, Fully Continual, Standard	0.534
**3**	Saturating CRM, Fully Continual, Balanced	0.531
**4**	Saturating CRM, Fully Continual, Standard	0.516
**5**	Peaking, Softmax 3 Stage	0.508
**6**	Weighted Uniform, 30	0.498
**7**	Peaking CRM, Fully Continual, Standard	0.498
**8**	Saturating Uniform, 30	0.488

**9**	Weighted CRM, Fully Continual, Balanced	0.482
**10**	Saturating, Softmax 3 Stage	0.482
**11**	Peaking CRM, Fully Continual, Balanced	0.472
**12**	Peaking Uniform, 30	0.434

Table S.Copelands.Peaking 5.Absolute Inaccuracy.

Rank	Approach	Score
**1**	Peaking CRM, Fully Continual, Standard	0.57
**2**	Peaking, Softmax 3 Step	0.562
**3**	Weighted CRM, Fully Continual, Standard	0.533
**4**	Weighted Uniform, 30	0.508
**5**	Weighted, Softmax 3 Step	0.503
**6**	Saturating, Softmax 3 Step	0.497
**7**	Peaking CRM, Fully Continual, Balanced	0.492
**8**	Peaking Uniform, 30	0.489
**9**	Saturating CRM, Fully Continual, Balanced	0.476
**10**	Saturating CRM, Fully Continual, Standard	0.474
**11**	Weighted CRM, Fully Continual, Balanced	0.471
**12**	Saturating Uniform, 30	0.427

Table S.Copelands.Peaking 5.SR.

Scenario Other 1

Rank	Approach	Score
**1**	Weighted CRM, Fully Continual, Standard	0.835
**2**	Saturating CRM, Fully Continual, Standard	0.83

**3**	Peaking CRM, Fully Continual, Standard	0.81
**4**	Saturating, Softmax 3 Stage	0.562
**5**	Weighted, Softmax 3 Stage	0.561
**6**	Weighted CRM, Fully Continual, Balanced	0.556
**7**	Peaking, Softmax 3 Stage	0.53
**8**	Saturating CRM, Fully Continual, Balanced	0.481
**9**	Peaking CRM, Fully Continual, Balanced	0.469
**10**	Saturating Uniform, 30	0.129
**11**	Peaking Uniform, 30	0.124
**12**	Weighted Uniform, 30	0.113

Table S.Copelands.Other 1.Average Regret.

Rank	Approach	Score
**1**	Saturating Uniform, 30	0.658
**2**	Saturating CRM, Fully Continual, Standard	0.613
**3**	Saturating CRM, Fully Continual, Balanced	0.598
**4**	Saturating, Softmax 3 Stage	0.531
**5**	Weighted, Softmax 3 Stage	0.482
**6**	Peaking CRM, Fully Continual, Balanced	0.481
**7**	Weighted Uniform, 30	0.46
**8**	Peaking CRM, Fully Continual, Standard	0.446
**9**	Peaking, Softmax 3 Stage	0.445
**10**	Weighted CRM, Fully Continual, Balanced	0.445
**11**	Peaking Uniform, 30	0.427
**12**	Weighted CRM, Fully Continual, Standard	0.413

Table S.Copelands.Other 1.Absolute Inaccuracy.

Rank	Approach	Score
**1**	Weighted CRM, Fully Continual, Balanced	0.7
**2**	Peaking CRM, Fully Continual, Balanced	0.675
**3**	Saturating CRM, Fully Continual, Balanced	0.584
**4**	Peaking, Softmax 3 Step	0.503
**5**	Weighted, Softmax 3 Step	0.488
**6**	Peaking Uniform, 30	0.47
**7**	Peaking CRM, Fully Continual, Standard	0.465
**8**	Weighted Uniform, 30	0.456
**9**	Weighted CRM, Fully Continual, Standard	0.449
**10**	Saturating, Softmax 3 Step	0.424
**11**	Saturating Uniform, 30	0.402
**12**	Saturating CRM, Fully Continual, Standard	0.385

Table S.Copelands.Other 1.SR.

Scenario Other 2

Rank	Approach	Score
**1**	Weighted CRM, Fully Continual, Standard	0.697
**2**	Saturating CRM, Fully Continual, Standard	0.657
**3**	Peaking CRM, Fully Continual, Standard	0.634
**4**	Weighted CRM, Fully Continual, Balanced	0.563
**5**	Saturating, Softmax 3 Stage	0.557
**6**	Saturating CRM, Fully Continual, Balanced	0.551
**7**	Peaking CRM, Fully	0.539

	Continual, Balanced	
**8**	Weighted, Softmax 3 Stage	0.5
**9**	Peaking, Softmax 3 Stage	0.481
**10**	Weighted Uniform, 30	0.294
**11**	Peaking Uniform, 30	0.274
**12**	Saturating Uniform, 30	0.253

Table S.Copelands.Other 2.Average Regret.

Rank	Approach	Score
**1**	Peaking, Softmax 3 Stage	0.568
**2**	Weighted, Softmax 3 Stage	0.549
**3**	Saturating CRM, Fully Continual, Standard	0.526
**4**	Peaking CRM, Fully Continual, Standard	0.524
**5**	Peaking CRM, Fully Continual, Balanced	0.509
**6**	Peaking Uniform, 30	0.508
**7**	Weighted CRM, Fully Continual, Balanced	0.489
**8**	Weighted Uniform, 30	0.484
**9**	Weighted CRM, Fully Continual, Standard	0.47
**10**	Saturating Uniform, 30	0.464
**11**	Saturating, Softmax 3 Stage	0.464
**12**	Saturating CRM, Fully Continual, Balanced	0.444

Table S.Copelands.Other 2.Absolute Inaccuracy.

Rank	Approach	Score
**1**	Weighted CRM, Fully Continual, Balanced	0.582
**2**	Peaking, Softmax 3 Step	0.566
**3**	Peaking Uniform, 30	0.536
**4**	Weighted, Softmax 3 Step	0.523
**5**	Saturating, Softmax 3 Step	0.519

**6**	Peaking CRM, Fully Continual, Balanced	0.519
**7**	Weighted Uniform, 30	0.518
**8**	Saturating Uniform, 30	0.489
**9**	Saturating CRM, Fully Continual, Balanced	0.463
**10**	Peaking CRM, Fully Continual, Standard	0.433
**11**	Saturating CRM, Fully Continual, Standard	0.427
**12**	Weighted CRM, Fully Continual, Standard	0.424

Table S.Copelands.Other 2.SR.

Scenario Other 3

Rank	Approach	Score
**1**	Weighted CRM, Fully Continual, Standard	0.788
**2**	Peaking CRM, Fully Continual, Standard	0.78
**3**	Saturating CRM, Fully Continual, Standard	0.771
**4**	Peaking CRM, Fully Continual, Balanced	0.678
**5**	Saturating CRM, Fully Continual, Balanced	0.645
**6**	Weighted CRM, Fully Continual, Balanced	0.624
**7**	Peaking, Softmax 3 Stage	0.429
**8**	Saturating, Softmax 3 Stage	0.414
**9**	Weighted, Softmax 3 Stage	0.413
**10**	Weighted Uniform, 30	0.157
**11**	Peaking Uniform, 30	0.154
**12**	Saturating Uniform, 30	0.148

Table S.Copelands.Other 3.Average Regret.

Rank	Approach	Score
**1**	Peaking Uniform, 30	0.563
**2**	Weighted CRM, Fully Continual, Standard	0.554
**3**	Peaking, Softmax 3 Stage	0.548
**4**	Peaking CRM, Fully Continual, Balanced	0.541
**5**	Weighted, Softmax 3 Stage	0.519
**6**	Weighted CRM, Fully Continual, Balanced	0.494
**7**	Peaking CRM, Fully Continual, Standard	0.486
**8**	Weighted Uniform, 30	0.478
**9**	Saturating CRM, Fully Continual, Balanced	0.469
**10**	Saturating, Softmax 3 Stage	0.465
**11**	Saturating CRM, Fully Continual, Standard	0.452
**12**	Saturating Uniform, 30	0.429

Table S.Copelands.Other 3.Absolute Inaccuracy.

Rank	Approach	Score
**1**	Peaking CRM, Fully Continual, Balanced	0.636
**2**	Weighted CRM, Fully Continual, Standard	0.593
**3**	Peaking Uniform, 30	0.587
**4**	Peaking, Softmax 3 Step	0.553
**5**	Weighted CRM, Fully Continual, Balanced	0.549
**6**	Peaking CRM, Fully Continual, Standard	0.502
**7**	Weighted, Softmax 3 Step	0.491
**8**	Saturating CRM, Fully Continual, Standard	0.477

**9**	Saturating CRM, Fully Continual, Balanced	0.471
**10**	Weighted Uniform, 30	0.438
**11**	Saturating, Softmax 3 Step	0.393
**12**	Saturating Uniform, 30	0.31

Table S.Copelands.Other 3.SR.

Scenario Other 4

Rank	Approach	Score
**1**	Saturating CRM, Fully Continual, Standard	0.63
**2**	Peaking CRM, Fully Continual, Balanced	0.592
**3**	Weighted CRM, Fully Continual, Standard	0.591
**4**	Saturating CRM, Fully Continual, Balanced	0.571
**5**	Weighted CRM, Fully Continual, Balanced	0.564
**6**	Peaking CRM, Fully Continual, Standard	0.552
**7**	Peaking, Softmax 3 Stage	0.473
**8**	Saturating, Softmax 3 Stage	0.471
**9**	Weighted, Softmax 3 Stage	0.463
**10**	Weighted Uniform, 30	0.374
**11**	Peaking Uniform, 30	0.364
**12**	Saturating Uniform, 30	0.355

Table S.Copelands.Other 4.Average Regret.

Rank	Approach	Score
**1**	Peaking CRM, Fully Continual, Standard	0.575
**2**	Peaking, Softmax 3 Stage	0.568
**3**	Weighted CRM, Fully Continual, Balanced	0.553

**4**	Weighted, Softmax 3 Stage	0.538
**5**	Weighted CRM, Fully Continual, Standard	0.526
**6**	Saturating, Softmax 3 Stage	0.513
**7**	Saturating CRM, Fully Continual, Standard	0.504
**8**	Saturating CRM, Fully Continual, Balanced	0.499
**9**	Peaking Uniform, 30	0.481
**10**	Weighted Uniform, 30	0.448
**11**	Saturating Uniform, 30	0.399
**12**	Peaking CRM, Fully Continual, Balanced	0.394

Table S.Copelands.Other 4.Absolute Inaccuracy.

Rank	Approach	Score
**1**	Peaking Uniform, 30	0.559
**2**	Weighted CRM, Fully Continual, Balanced	0.536
**3**	Weighted CRM, Fully Continual, Standard	0.528
**4**	Peaking CRM, Fully Continual, Standard	0.527
**5**	Peaking, Softmax 3 Step	0.519
**6**	Saturating CRM, Fully Continual, Standard	0.512
**7**	Weighted, Softmax 3 Step	0.51
**8**	Weighted Uniform, 30	0.5
**9**	Peaking CRM, Fully Continual, Balanced	0.498
**10**	Saturating Uniform, 30	0.452
**11**	Saturating CRM, Fully Continual, Balanced	0.434
**12**	Saturating, Softmax 3 Step	0.424

Table S.Copelands.Other 4.SR.

Supplementary References

1. Glass, E.J. Genetic Variation and Responses to Vaccines. *Anim. Health Res. Rev.* **2004**, *5*, 197–208, doi:10.1079/ahr200469.
2. O'Quigley, J.; Iasonos, A.; Bornkamp, B. *Handbook of Methods for Designing and Monitoring Dose Finding Trials*; 2019; ISBN 978-0-367-33068-2.
3. Symonds, M.R.E.; Moussalli, A. A Brief Guide to Model Selection, Multimodel Inference and Model Averaging in Behavioural Ecology Using Akaike's Information Criterion. *Behav. Ecol. Sociobiol.* **2011**, *65*, 13–21, doi:10.1007/s00265-010-1037-6.
4. van Hasselt, H.; Guez, A.; Silver, D. Deep Reinforcement Learning with Double Q-Learning. *ArXiv150906461 Cs* **2015**.
5. Hodges, J.L. The Significance Probability of the Smirnov Two-Sample Test. Ark. För Mat. 1958, 3, 469–486, doi:10.1007/BF02589501.
6. Mann, H.B.; Whitney, D.R. On a Test of Whether One of Two Random Variables Is Stochastically Larger than the Other. Ann. Math. Stat. 1947, 18, 50–60, doi:10.1214/aoms/1177730491.
7. Weisstein, E.W. Bonferroni Correction Available online: https://mathworld.wolfram.com/ (accessed on 29 April 2022).
